# Selective Chiral Diamine-bisoxazoline Iron(II) Catalysts for Pyrrolidine Formation via Intramolecular C(sp^3^)–H Amination of Aliphatic Azides

**DOI:** 10.1021/jacs.5c14105

**Published:** 2025-11-18

**Authors:** Zhiyuan He, Stijn W. J. de Wit, Samuel M. van der Loo, Dorette S. Tromp, Alexis K. Bauer, Michael L. Neidig, Simon Mathew, Andreas W. Ehlers, Bas de Bruin, Jarl Ivar van der Vlugt

**Affiliations:** a Homogeneous, Supramolecular Catalysis, and Bio-Inspired Catalysis Group, van ’t Hoff Institute for Molecular Sciences (HIMS), 1234 University of Amsterdam, 1098 XH Amsterdam, The Netherlands; b Inorganic Chemistry Laboratory, Department of Chemistry, 6396 University of Oxford, Oxford OX1 3QR, United Kingdom; c Bioinspired Coordination Chemistry and Homogeneous Catalysis Group, Institute of Chemistry, School of Mathematics and Science, Carl von Ossietzky University Oldenburg, 26129 Oldenburg, Germany

## Abstract

The utilization of iron-catalyzed intramolecular C­(sp^3^)–H amination starting from organic aliphatic azides, which are easily available precursors, represents a particularly promising route to synthesize sought-after *N*-heterocyclic motifs. While significant advances in achiral iron catalysis have been made by the field in recent years, we introduce here a new, highly reactive chiral diamine-bisoxazoline iron­(II) system for asymmetric C–H amination with primary azides. The presence of two distinctly different chiral components, i.e., an atropisomeric binaphthyl-diamine backbone and two stereochemically identical oxazoline units, leads to the formation of four stereoisomers, which revealed distinctly different reactivity for the two sets of diastereomers available. Kinetic experiments and detailed computation studies point to a stepwise radical mechanism after the initial rate-limiting azide activation via a 1,5-HAT process at the quintet spin surface. Ligand modification (substitution at the oxazoline ring) significantly enhanced the reactivity, chemoselectivity, and enantioselectivity, thus enabling the synthesis of pyrrolidines in high yield with good stereocontrol under mild, additive-free conditions and with N_2_ as the sole byproduct.

## Graphical abstract

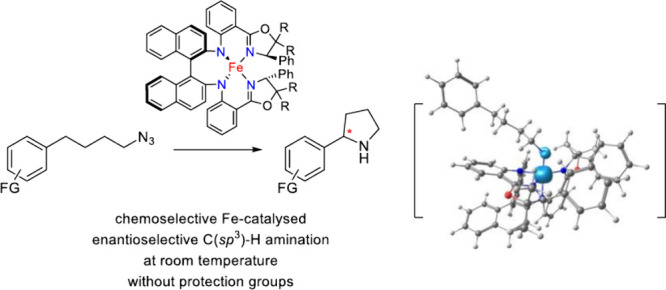

## Introduction

C–H amination has received significant attention in recent years as a powerful and versatile strategy to construct C–N bonds, in particular for the synthesis of pharmaceutically relevant *N*-heterocyclic compounds.^1^ Being essentially an *N*-group transfer process, this reaction revolves around the generation and activation of a nitrene precursor (e.g., haloamine, hydroxylamine, iminoiodinane, *N*-sulfonylazide, organoazide), predominantly in the coordination sphere of a transition metal.^2^ This methodology has been successfully implemented using a variety of metal catalysts, with iron emerging as a particularly promising candidate due to its low toxicity, abundance, and unique electronic properties. This includes a moderately high d-electron count and a compressed ligand field, thus favoring population of the iron nitrene *anti*-bonding orbitals and thereby enhancing the reactivity of the metal-nitrene intermediate toward C–H activation.^3^ Iron-catalyzed intramolecular C–H amination, particularly using readily accessible organic azides such as primary aliphatic azides, has unlocked new synthetic pathways for the generation of *N*-heterocyclic motifs which are the structures of immense value in pharmaceuticals.^4^ Despite these advancements, iron-catalyzed C–H amination still faces several important remaining challenges, as current Fe-based systems, such as those reported by the groups of Betley and Che as well as our own group (Figure 1A–C), require harsh reaction conditions (high temperatures) and the need for additives like Boc_2_O to prevent product inhibition. Moreover, most Fe-based system exhibit a limited substrate scope and still face chemoselectivity issues, whereby competing pathways, such as undesired 1,2-HAT (hydrogen atom transfer) reactions, lead to undesired (imine, nitrile) side product formation.^5^ Furthermore, none of the reported molecular Fe-based systems can achieve any level of chiral induction. To date, a few Fe systems have been reported to be catalytically active in the absence of a protecting group such as Boc_2_O, but only for *gem*-Me,Me-azides, where the 1,2-HAT reactionis not possible.^6^ Addressing these limitations thus represents a critical frontier in the development of more efficient and selective catalytic systems for C–H amination.

**Figure 1:**
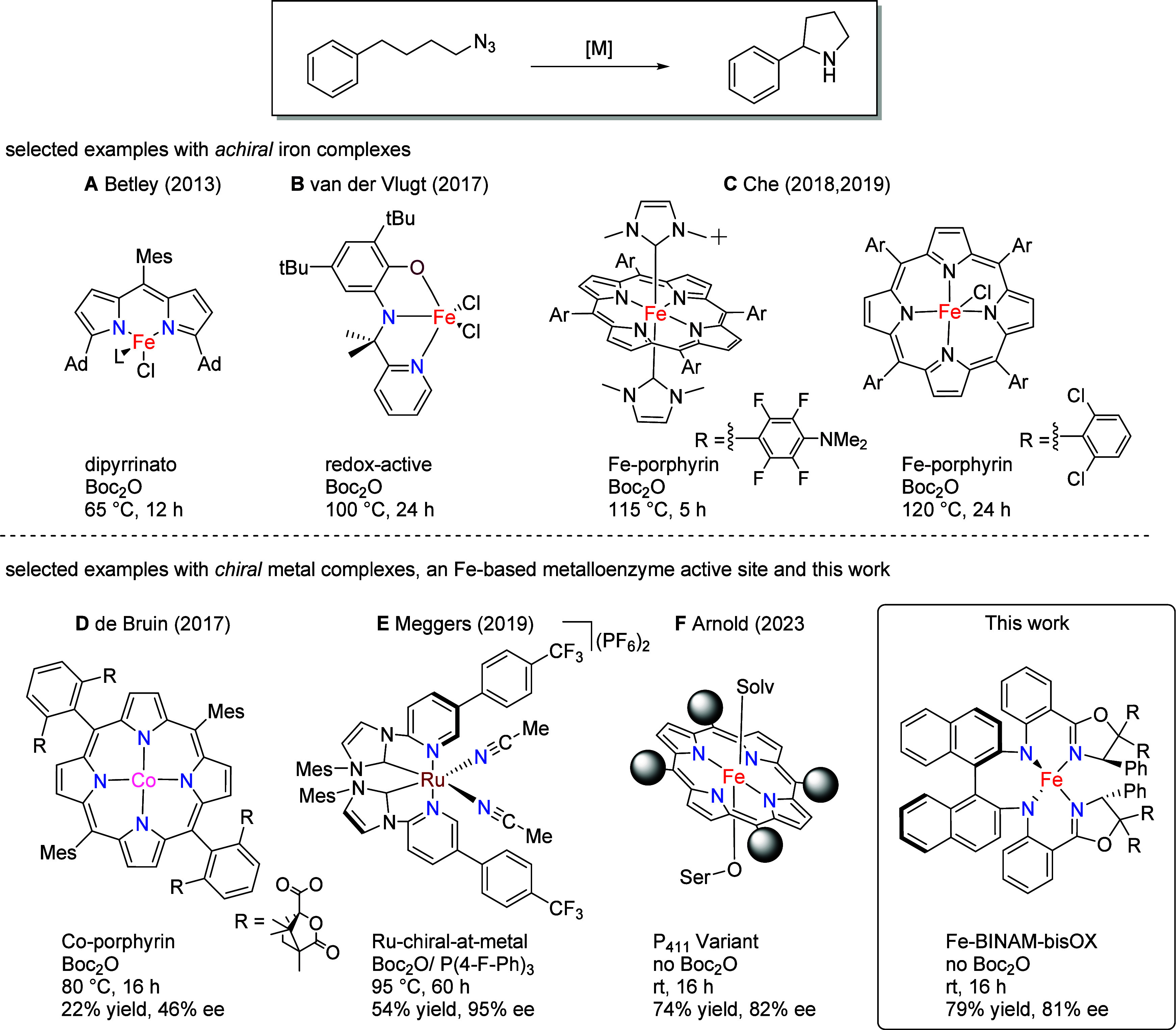
Selective examples of achiral (top) and chiral (bottom) C­(sp^3^)–H amination reactions.

P450-based enzymes have demonstrated significant potential in addressing the above-mentioned limitations associated with iron-catalyzed C–H amination. Through directed evolution, the Arnold group developed cytochrome P411 variants that efficiently catalyze the synthesis of pyrrolidines under mild conditions with notable enantioselectivity (Figure 1F).^7^ However, this transformation remains constrained by challenges such as scalability (currently limited to analytical-scale reactions), substrate dependency, and a lack of detailed mechanistic understanding. Despite these hurdles, the success of P411 variants underscores the promise of iron-based catalysis for this challenging transformation, offering a foundation for future advancements in molecular iron catalysis for C–H amination via (net, formal) nitrene insertion. In part inspired by the activity of enzymes, several advances with molecular iron catalysts for organoazide C–H amination have relied on porphyrin-based ligand systems (Figure 1C).^8^ Related (chiral) cobalt porphyrins have been studied in our own group (Figure 1D), providing the first example of *enantioselective* transition metal nitrene (radical) insertion into a C–H bond of the substrate for the synthesis of *N*-heterocycles from organic azides.^9^ However, these systems exhibit limited reactivity and low enantioselectivity, particularly for primary aliphatic azides. The Meggers group recently disclosed highly interesting developments with a chiral-at-Ru catalyst (Figure 1E).^10^ This catalyst achieves excellent enantioselectivity (ee), but it requires a high reaction temperature and the presence of Boc_2_O. Furthermore, the associated toxicity, low abundance, and high cost of such a 4d-metal catalyst place it at a disadvantage compared with iron-based catalysts, particularly in the synthesis of pharmaceutically relevant nitrogen-containing heterocyclic compounds. As such, all current approaches still face limitations in addressing these challenges. As a ligand design principle, we anticipated that higher reactivities might be achievable with rigid electron-rich and chiral tetradentate N_4_-ligands enforcing a strongly twisted, constrained geometry around the metal center compared to traditional porphyrin systems, concomitantly imposing a high-spin Fe^II^ configuration.

In the present paper, we introduce a series of diamine-bisoxazoline ligands that, when deprotonated and coordinated to Fe­(II), induce a (distorted) tetrahedral coordination geometry and a high spin configuration of the iron­(II) center. This ligand design aims to enhance reactivity and selectivity, in particular toward challenging substrates such as primary aliphatic azides. Furthermore, we anticipated chiral induction during the C–N forming step by including the “privileged” oxazoline motif,^11^ thus providing the first example of Fe-mediated asymmetric C­(sp^3^)–H-amination of primary organoazides with a *molecular* Fe-catalyst. Diamine-bisoxazoline ligands based on *tropos*-type 2,2′-diaminobiphenyl backbone were first reported by Doherty et al.^12^ Copper complexes with these ligands proved configurationally stable, thus highlighting their potential for precise stereochemical control. Additionally, Pan et al.^13^ demonstrated the ability of these tetradentate ligands to form mononuclear complexes with a variety of transition metals.

Yet the practical application of diamine-bisoxazoline ligands in metal-catalyzed asymmetric transformations remains underexplored. This gap in development has sparked our interest in leveraging diamine-bisoxazoline ligands for iron-based catalysis, particularly for challenging asymmetric reactions, where their structural and electronic properties could offer significant advantages in reactivity and enantioselectivity. We herein discuss ligand design and optimization strategies, Fe­(II) coordination chemistry and catalysis including kinetic and mechanistic investigations (using DFT) and showcase the applicability of these Fe­(II) complexes for the synthesis of selected *N*-heterocyclic building blocks for bioactive compounds.

## Results and Discussion

### Synthesis of Chiral Tetradentate Ligands and Corresponding Iron Complexes

The new ligand *gem*-H,H-(*R*,*S*,*S*)-**L1** (Scheme 1a) was synthesized via a Buchwald–Hartwig coupling between the commercially available *atropos*-diamine (*R*)-BINAM ([1,1′-binaphthalene]-2,2′-diamine) and (4*S*)-2-(2-bromophenyl)-4,5-dihydro-4-phenyloxazole (accessible via a one-step procedure using commercial precursors), utilizing the well-established second generation catalyst system comprised of Pd_2_(dba)_3_ and *rac*-BINAP [2,2′-bis­(diphenylphosphino)-1,1′-binaphthyl]; see Supporting Information for details). Subsequent treatment with Fe­(HMDS)_2_ resulted in clean formation of the red complex (*R*,*S*,*S*)-**1** (Scheme 1a). ^1^H NMR spectroscopic analysis confirmed the disappearance of signals associated with the free **L1** and the presence of new signals over a broad chemical shift range, consistent with the generation of a paramagnetic species.

**Scheme 1:**
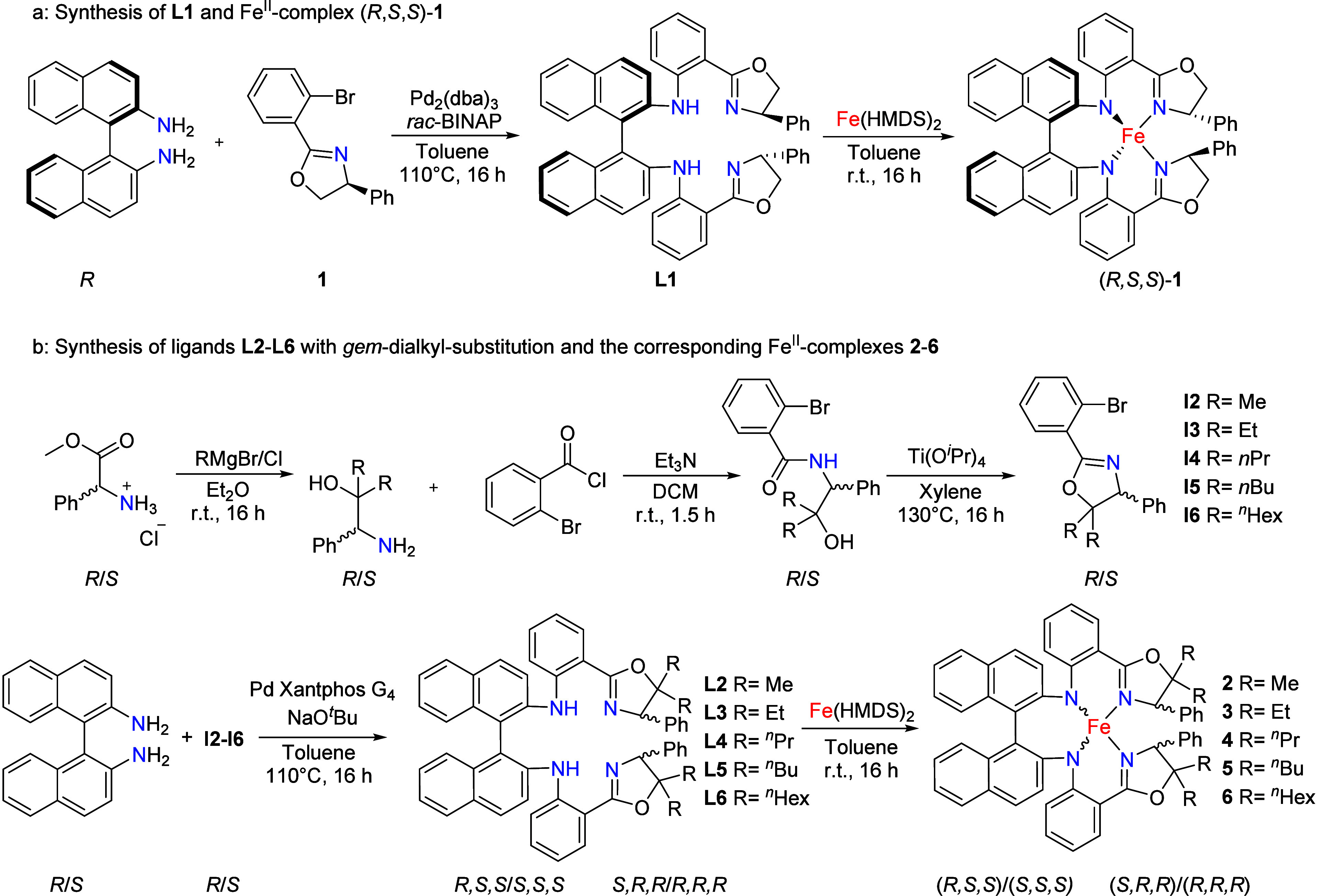
(a) Synthetic Protocol toward Ligand *gem*-H,H-(*R*,*S*,*S*)-**L1** and Fe Complex (*R*,*S*,*S*)-**1**. and (b) Improved Synthetic Protocol toward Ligands with *gem*-Dialkyl Substitution

Because the ligand *gem*-H,H-(*R,S,S*)-**L1** was expected (and found) to be sensitive to dehydrogenation of the dihydrooxazole fragment under the applied catalytic conditions (*vide infra*), we prepared a series of *gem*-dialkyl-substituted oxazoline analogs using a modified literature procedure.^14^ The required dialkyl-substituted amino alcohols were obtained by reacting d-phenylglycine methyl ester hydrochloride with different alkyl Grignard reagents, and then converted to the desired *gem*-dialkyl-substituted oxazoline fragments **I2**–**I6** (Scheme 1b) using a two-step approach involving 2-bromobenzoyl chloride and titanium isopropoxide. Subsequent Buchwald–Hartwig coupling reactions via the same protocol as that used for *gem*-H,H-(*R,S,S*)-**L1** suffered from low yields. However, reaction optimization via a catalyst screening process allowed us to identify XantphosG4 ^15^ as a highly effective catalyst for the final C–N bond forming step, leading to successful preparation of a series of *gem*-R,R-substituted oxazoline-based ligands **L2**–**L6** (Scheme 1b).

Coordination to Fe­(II) was explored in detail for ligand **L2**, including an investigation of the spectroscopic properties of the resulting iron complexes. By systematically varying the chirality of both the BINAM and oxazoline moieties, we obtained four distinct stereoisomers of *gem*-Me,Me-substituted ligand **L2**: (*R,S,S*), (*S,S,S*), (*S,R,R*), and (*R,R,R*). Subsequent reaction of each variant of **L2** with Fe­(HMDS)_2_ in toluene overnight yielded the corresponding Fe­(II) complexes. As expected, the ^1^H NMR spectra of the enantiomeric pairs (*R,S,S*)-**2** and (*S,R,R*)-**2** and (*S,S,S*)-**2** and (*R,R,R*)-**2** were identical, whereas (*R,S,S*)-**2** displayed significant peak shifts relative to its diastereomer (*R,R,R*)-**2** (Figure 2).

**Figure 2:**
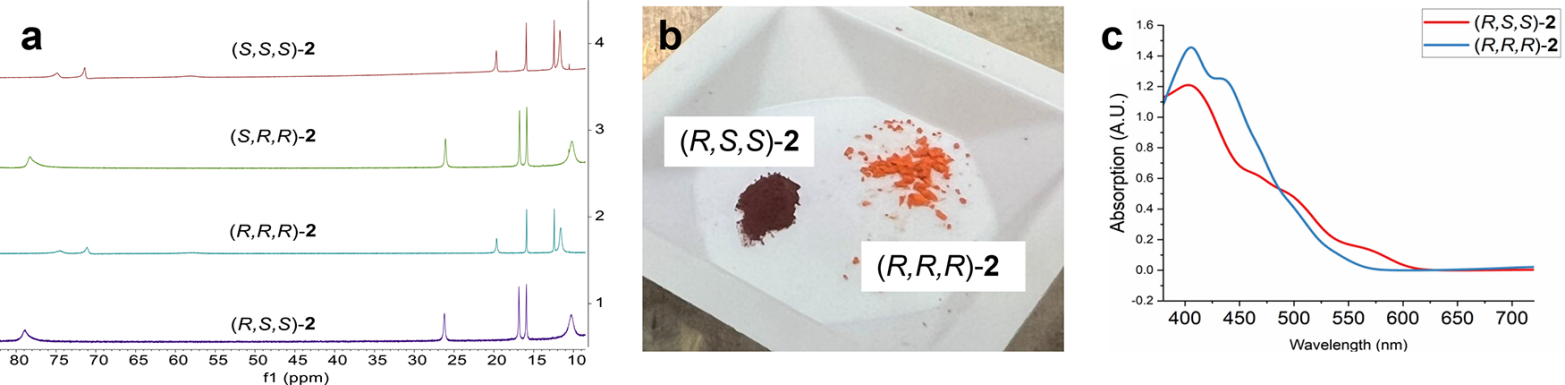
(a) Partial ^1^H NMR spectra of (*S,S,S*)-**2**, (*S,R,R*)-**2**, (*R,R,R*)-**2**, and (*R,S,S*)-**2** in C_6_D_6_. (b) Difference in color between (*R,S,S*)-**2** and (*R,R,R*)-**2**. (c) overlaid UV–vis spectra of (*R,S,S*) and (*R,R,R*)-**2.**

The diastereomer pair [(*R,S,S*)-**2** and (*S,S,S*)-**2**] also exhibited visibly distinct colors (Figure 2). UV–vis spectroscopic analysis of (*R,S,S*)-**2**, (*S,R,R*)-**2**, and (*R,R,R*)-**2** revealed that the latter showed more pronounced absorption bands between 500 and 600 nm, tentatively assigned to MLCT events (and somewhat lower intensity bands from 400 to 500 nm). This indicates that these diastereomers adopt different overall molecular structures, which may also impact the underlying electronic configurations as well as their reactivity.

Single crystals for both diastereomers of **2** were obtained from a saturated toluene (*R*,*S*,*S*)-**2** and benzene (*S*,*S*,*S*)-**2** solution, respectively. The enantiomer of the latter, (*R,R,R*)-**2**, was also obtained from a saturated benzene solution, while (*S*,*R*,*R*)-**2** could be crystallized from pentane solution with a minimum amount of toluene at −30 °C. This likely reflects small differences in the electric field gradients, potentially arising from the minor bond distance/angle variations observed in the respective molecular structures, which also feature solvent-filled voids in place of close packing, impacting the overall symmetry of the asymmetric unit. The N_ox_–Fe–N_ox_ bite angles and τ_4_ geometry^16^ indices are nearly identical for the two sets of enantiomers but substantially different *between* the sets of diastereomers: (*S*,*S*,*S*)-**2** (106.41°, τ_4_ = 0.64; (*R,R,R*)-**2** (102.49°, τ_4_ = 0.54; 102.75°, τ_4_ = 0.55) versus (*S*,*R*,*R*)-**2** (126.90°, τ_4_ = 0.74; 123.12°, τ_4_ = 0.75) and (*R*,*S*,*S*)-**2** (127.54°, τ_4_ = 0.74; 122.90°, τ_4_ = 0.75); see Figures 3, S21, and S22 for the respective molecular structures. Hence, comparing the (*S*,*S*,*S*)-**2**/(*R*,*S*,*S*)-**2** diastereomer pair, the chirality of the BINAM backbone seems to directly impact the overall geometry around the Fe center, which potentially also affects the structural flexibility of the respective conformers upon substrate binding.

**Figure 3:**
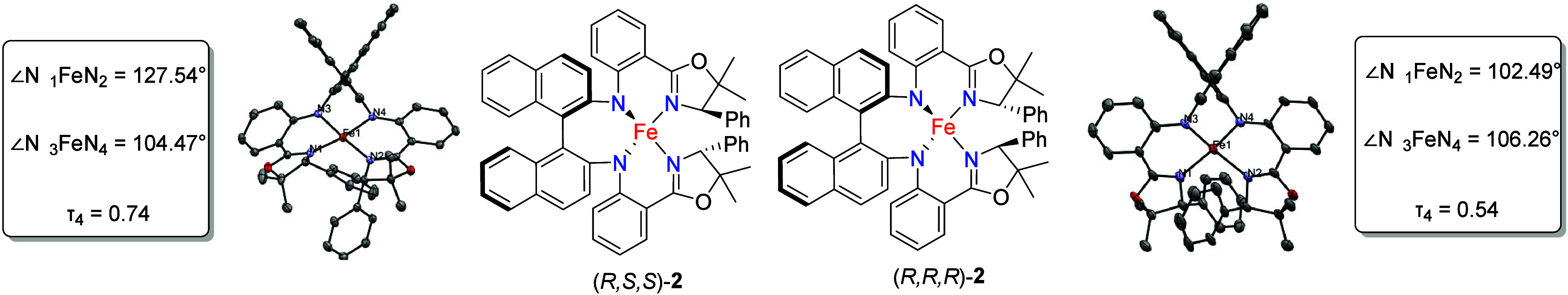
ORTEP plot (50% thermal ellipsoids) of the molecular structures of (*R,R,R*)-**2** and (*R,S,S*)-**2**. Only the independent molecules in the asymmetric unit cell are shown. Hydrogen atoms and solvent molecules have been omitted for the sake of clarity.

### Mössbauer Spectroscopy

To further probe potential variations in electronic structure and bonding in these two diastereomeric complexes, Mössbauer spectroscopy was performed on both (*S,S,S*)-**2** and (*R,S,S*)-**2** (Figure 4). The Mössbauer spectrum for the latter revealed an isomer shift δ of 0.79 mm s^–1^ and a quadrupole moment |Δ*E*_Q_| of 1.57 mm s^–1^.

**Figure 4:**
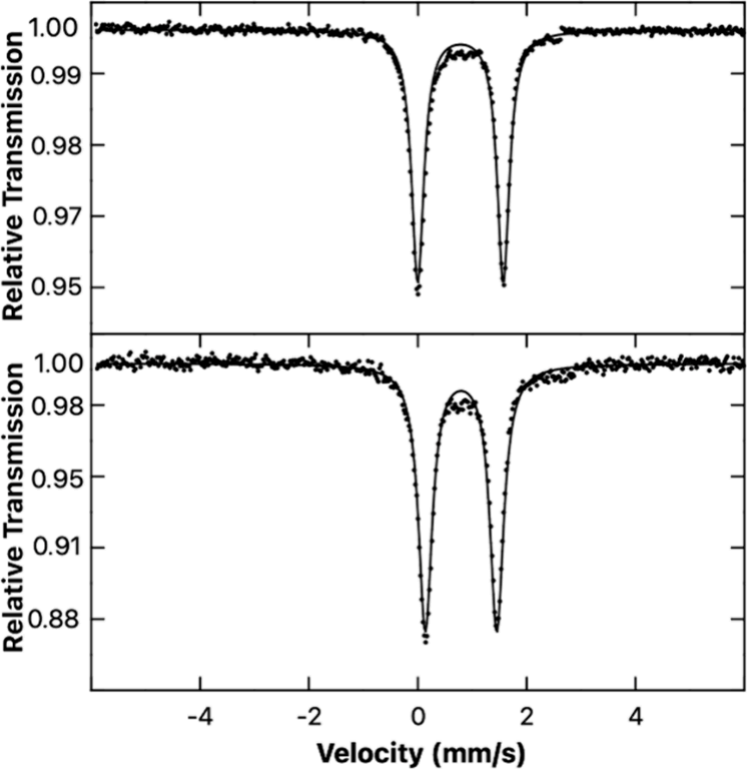
Mössbauer spectra of the diastereomers (*R*,*S*,*S*)-**2** (top) and (*S*,*S*,*S*)-**2** (bottom) measured at 80 K. For (*R,S,S*)-**2**: δ = 0.79 mm s^–1^, |Δ*E*_Q_| = 1.57 mm s^–1^. For (*S*,*S*,*S*)-**2**: δ = 0.79 mm s^–1^, |Δ*E*_Q_| = 1.31 mm s^–1^.

The spectrum of (*S,S,S*)-**2** provided the same isomer shift, as expected, but a lower quadrupole moment |Δ*E*_Q_| of 1.31 mm s^–1^. The isomer shift and quadrupole splitting values for the two diastereomers are consistent with high-spin Fe­(II) complexes, with the small differences in the observed quadrupole splitting values likely reflecting small differences in the electric field gradients caused by the somewhat different geometries of the diastereomers.^17^

### Intramolecular C­(sp^3^)–H Amination Catalysis

We first tested the catalytic activity of Fe­(II) complex (*R*,*S*,*S*)-**1** toward the benchmark substrate (4-azidobutyl)­benzene at room temperature (Table 1, entry 1). Interestingly, this substrate could be converted to the desired 2-phenylpyrrolidine product even *in the absence of* di-*tert*-butyl dicarbonate (Boc_2_O). This additive, which acts as *N*-protection group to overcome catalyst deactivation by competitive binding of the parent pyrrolidine to the metal center, is a necessity in all reported Fe-based systems capable of converting this particular benchmark substrate. However, the observed catalytic efficiency of complex (*R*,*S*,*S*)-**1** in terms of conversion (TON = 3) nevertheless proved to be relatively low. We believe this is caused by the relatively easy (formal) dehydrogenation of the unprotected oxazoline backbone under the applied catalytic reaction conditions, leading to significant structural reorganization (due to the formation of the planar oxazole ring) around the Fe center, which could induce catalyst inactivity. Indeed, LIFDI (liquid injection field desorption ionization) mass spectrometry of a sample from the reaction between (*R*,*S*,*S*)-**1** and 3,5-ditrifluoromethyl phenyl azide as a model substrate (incapable of undergoing follow-up C–H amination) revealed the loss of two mass units for the major species detected, which is interpreted to result from substantial dehydrogenation of the oxazoline moiety within the ligand (thereby generating the corresponding oxazole fragment). The hydrogen atoms are probably picked up by the metal-bound nitrene, as we observed the corresponding aniline after reaction. This unwanted side reaction should be suppressed by usage of the Fe-complexes (*R*,*S*,*S*)-**2**, (*R*,*S*,*S*)-**3**, (*R*,*S*,*S*)-**4**, (*R*,*S*,*S*)-**5**, and *(R,S,S)*-**6** containing *gem*-dialkyl-substituted oxazoline ligands, for which the alkyl-substituents at the 5-position of the oxazoline ring protect the ligands from dehydrogenation. Indeed, as anticipated, the corresponding Fe complex (*R*,*S*,*S*)-**2** did not undergo ligand dehydrogenation upon reaction with the aryl azide, as confirmed by LIFDI-MS.

**Table 1:**
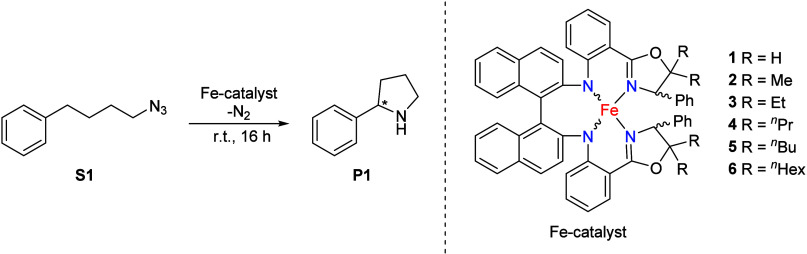
Optimization of Catalytic C­(sp^3^)–H Amination of the Benchmark Substrate (4-Azidobutyl)­benzene^a^

entry	catalyst	solvent	*T* (°C)	conversion (%)^b^	yield (%)^b^	ee (%)^c^
1​	(*R,S,S*)-**1**	pentane	rt	16	11	–33
2​^d^		pentane	rt	64	<5	nd
3​^e^		pentane	rt	95	47	58
4​	(*R,S,S*)-**2**	pentane	rt	79	62 (60)^f^	65 (−63)^f^
5​	(*R,S,S*)-**2**	benzene	rt	85	69	61
6​	(*R,S,S*)-**2**	diethyl ether	rt	93	67	63
7​	(*R,S,S*)-**2**	THF	rt	59	46	55
8​	(*R,R,R*)-**2**	pentane	rt	0		
9​	(*R,S,S*)-**3**	pentane	rt	>99	87	68
10​	(*R,S,S*)-**4**	pentane	rt	>99	86	78
11​	(*R,S,S*)-**5**	pentane	rt	>99	82	79
12​	(*R,S,S*)-**6**	pentane	rt	>99	79	81
13​	(*R,S,S*)-**6**	pentane	50	98	78	76
14​	(*R,S,S*)-**6**	pentane	–5	>99	67	82

a Conditions: substrate (0.15 mmol) and Fe-catalyst (5 mol %) were stirred in solvent (2.5 mL) for 16 h.

b Calculated via ^1^H NMR analysis with 1,3,5-trimethoxybenzene as internal standard.

c ee determined via chiral GC.

d Substrate (0.15 mmol) and Fe­(HMDS)_2_ (5 mol %) were stirred in solvent (2.5 mL) for 16 h.

e Substrate (0.15 mmol), Fe­(HMDS)_2_ (5 mol %), and ligand (*R*,*S*,*S*)-**L2** (5 mol %) were stirred in solvent (2.5 mL) for 16 h.

f ee recorded for reaction catalyzed by diastereomer (*S*,*R*,*R*)-**2**. Assignment of optical isomer formed was done using an authentic enantiopure sample and comparing chiral GC retention times.

To verify that the diamine-bisoxazoline ligand has a positive effect on the conversion of the benchmark substrate (4-azidobutyl)­benzene compared to the “ligand-free” iron precursor Fe­(HMDS)_2_, we performed a control experiment with the latter (Table 1, entry 2). An overnight run using this species generated only stoichiometric amounts of pyrrolidine at room temperature (entry 1), whereas simultaneous addition of (4-azidobutyl)­benzene (0.15 mmol), Fe­(HMDS)_2_ (5 mol %) and (*R,S*,*S*)-**L2** (5 mol %) afforded the unprotected pyrrolidine as the main product (47%, 58% ee; entry 3) after 16 h of reaction at room temperature. Notably, for the reaction in the presence of (*R,S*,*S*)-**L2**, the reaction proceeds without the need for any additive, protecting group, or heating, thus highlighting the importance of the ligand to boost catalytic activity. The isolated Fe-complex (*R*,*S*,*S*)-**2** exhibits comparable catalytic performance while demonstrating improved selectivity for the desired pyrrolidine product (reduced side product formation; entry 4). The higher conversion observed in entry 2 versus entry 3 is likely due to the enhanced solubility of both free Fe­(HMDS)_2_ and Fe­(HMDS)_2_ ligand adducts in pentane, which improves the reactivity (note that the isolated Fe-complex (*R*,*S*,*S*)-**2** is not completely soluble in pentane under the applied reaction conditions). Complex formation with Fe­(HMDS)_2_ and ligand, as monitored by NMR spectroscopy, requires 16 h to reach full conversion under these conditions (Figure S3).

The enantioselectivity and reactivity were both found to be sensitive to the solvent polarity. THF, the most polar solvent used, resulted in the lowest conversion and enantioselectivity (entry 6). In contrast, less polar solvents improved the enantioselectivity. The enantioselectivity was the highest in pentane (65% ee), albeit with a slightly reduced yield (62%; entry 4) when compared to benzene (69%; entry 5) or diethyl ether (67%; entry 6) due to lower solubility of the preformed Fe-complex (*R*,*S*,*S*)-**2** in pentane.

Remarkably, (*R*,*S*,*S*)-**1** generates the opposite enantiomer of 2-phenylpyrrolidine, compared to any of the *gem*-dialkyl based catalysts (*R*,*S*,*S*)-**2**-**6**, and with much lower enantioselectivity. Complex (*R,S*,*S*)-**3** (*gem*-Et,Et; entry 9) resulted in enhanced enantioselectivity (68% ee) and yield (87%). Further lengthening of the *gem*-alkyl,alkyl substituents to give complex (*R,S*,*S*)-**6** (entry 12) improved the ee to 81%. The enhanced enantioselectivity may be explained by the increased contact area and thus van der Waals (dispersion) interactions between the substrate and the elongated alkyl chains.

We observed that the reactivity displayed by the Fe-center is highly dependent on the specific chiral information stored in the ligand framework. While the (*R,S,S*)-**2** complex showed a high activity, the (*R,R,R*)-**2** complex proved to be completely inactive toward (4-azidobutyl)­benzene under the same reaction conditions, showcasing that the overall reactivity and subsequent enantioinduction are primarily governed by the oxazoline fragments. We attribute the large differences in reactivity to the dissimilar steric environment induced by the respective ligand coordination to the Fe-center, consistent with our initial hypothesis (*vide supra*). Performing the reaction with the enantiomer (*S,R,R*)-**2** (entry 4) afforded the corresponding pyrrolidine stereoisomer with comparable ee (for the opposite enantiomer) and yield (−63% ee, 60% yield), further supporting the ligand’s role in stereochemical control.

The reaction temperature affects the enantioselectivity to only some extent. Conducting the reaction at 50 °C using (*R,S*,*S*)-**6** led to a decrease in ee by 4% (entry 13), while cooling to −5 °C led to a modest increase in ee by 1% ee (entry 14). Given the practical challenges of inert cooling of the reaction mixture, room temperature was selected for subsequent scope investigations.

Varying the catalyst concentration or the catalyst loading did not significantly alter the chemoselectivity of the reaction (Supporting Information). Therefore, the initial reaction conditions (0.15 mmol substrate, 5 mol % Fe-complex in 2.5 mL pentane) were selected for further catalysis tests. Monitoring the catalytic reaction every 30 min for a total of two and a half h showed no change in the ee over this period, underscoring the robustness and stability of the tetradentate system (see Supporting Information).

Using various non-natural amino acids, accessible by the procedure reported by Meggers and co-workers,^18^ we prepared several derivatives of our ligand with different substituents at the carbon stereocenters in the oxazoline fragments. However, replacing the phenyl group with larger aromatic rings resulted in a lower enantiomeric excess (ee). Furthermore, no reactivity was obtained at room temperature when changing the phenyl substituent at the oxazoline to an alkyl group (see Suppporting Information for more details).

### Kinetic Investigations

To shed more light on the underlying mechanism of the intramolecular C­(sp^3^)–H amination using our Fe-catalyst, a kinetic analysis of the reaction progress was undertaken by monitoring the conversion of the (4-azidobutyl)­benzene substrate into the desired 2-phenylpyrrolidine with (*R,S,S*)-**2** as the catalyst and using paramagnetic ^1^H NMR spectroscopy (see Supporting Information) revealed clean first-order kinetics in both the [substrate] and [catalyst]. No product inhibition was observed, making it the first example of intramolecular C–H amination of this substrate for which the kinetics do not depend on the pyrrolidine concentration. This also explains why no Boc_2_O is needed to trap the product at the final stage of the catalytic cycle.

Remarkably, the reaction rate for the conversion of (4-azidobutyl)­benzene increased more than 30-fold with the isolated complex (*R*,*S*,*S*)-**2** as compared to the isolated complex (*R*,*S*,*S*)-**1** containing the unsubstituted ligand (Figure 5). Furthermore, the catalytic reaction rate was subtly affected by the alkyl chain substituents at the *gem*-dialkyl position, with an increase in rate realised on going fto in (*R*,*S*,*S*)-**3** from (*R*,*S*,*S*)-**2** to (*R*,*S*,*S*)-**3** (*gem*-Et,Et) and (*R*,*S*,*S*)-**4** (*gem*-nPr,nPr). Further elongation of the side chain to *n*-butyl or *n*-hexyl did not lead to further absolute improvements in terms of reaction rate.

**Figure 5:**
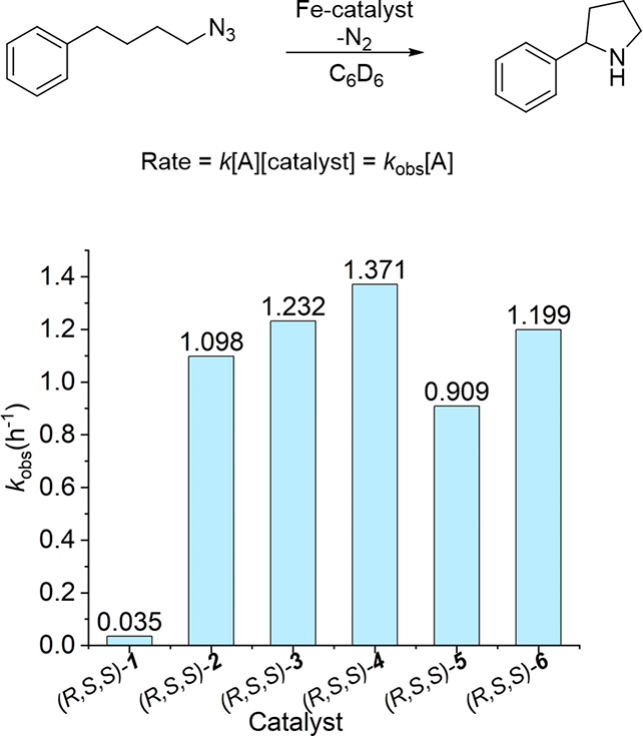
Comparison of the catalytic activity of the complexes (*R*,*S*,*S*)-**1**–(*R,S,S*)-**6** in the C­(sp^3^)-H amination of the benchmark substrate (4-azidobutyl)­benzene at room temperature with 14 mM catalyst and 0.008 mmol of substrate in 0.6 mL of C_6_D_6_.

To determine whether azide activation or C–H bond-activation is rate-limiting, we performed both an *intra-* and *inter*molecular kinetic isotope competition experiment, using either the monodeuterated analogue or a mixture of the normal and the bis-deuterated analogue of the (4-azidobutyl)­benzene substrate, respectively. In the *inter*molecular competition experiment, no kinetic isotope effect (KIE = 1) was observed, thus showing that the C–H bond-activation step occurs after rate-determining azide activation. The *intra*molecular kinetic isotope competition experiment did reveal a substantial kinetic isotope effect (KIE = 5.45), showing that the C–H activation step is not barrierless (Scheme 2). The magnitude of this KIE points to a radical-type hydrogen-atom transfer (HAT) process.^19^

**Scheme 2:**
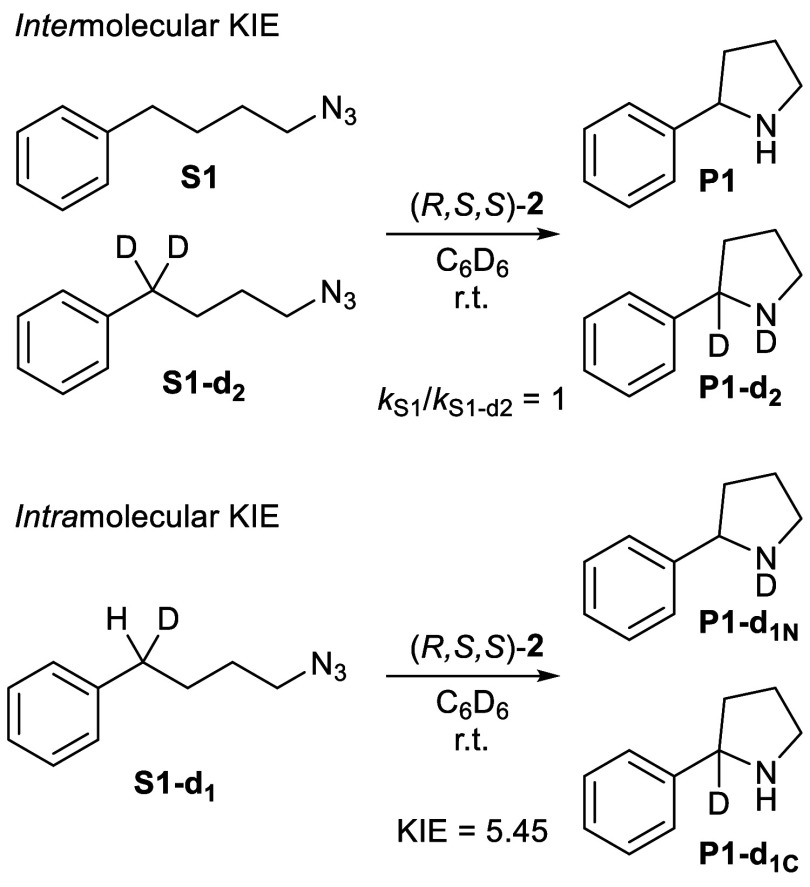
Experiments To Determine *Inter-* and *Intra*molecular Kinetic Isotope Effects for the C­(sp^3^)–H Amination of Benchmark Substrate (4-Azidobutyl)­benzene

The reaction rate was further evaluated at various temperatures (25–45 °C) to obtain the relevant activation parameters from the Arrhenius and Eyring equations (see Supporting Information for details). Catalyst (*R,S,S*)-**2** exhibits a remarkable low activation energy (*E*_a_ = 7.80 ± 0.12 kcal mol^–1^) and a correspondingly low free energy barrier Δ*G* ^⧧^_298K_ of 14.64 ± 0.17 kcal mol^–1^, with a low enthalpic contribution Δ*H* ^⧧^ of 7.16 ± 0.12 kcal mol^–1^ and a rather large entropic involvement (Δ*S* ^⧧^ = −25.08 ± 0.38 cal mol^–1^ K^–1^). When compared to the reference system Co­(TMP),^9^ the superior efficiency of (*R*,*S*,*S*)-**2** becomes evident: its activation energy is ∼11 kcal mol^–1^ lower, and its overall activation free energy is 9.9 kcal mol^–1^ lower. The enthalpic barrier of (*R,S,S*)-**2** is >10 kcal mol^–1^ smaller than that of Co­(TMP) (Table 2), resulting in a high catalytic activity that enables the reaction to proceed even at −5 °C. Overall, this catalytic system demonstrates the highest reactivity reported to date under these mild conditions.

**Table 2:** Activation Parameters for (*R,S,S*)-**2** vs Co­(TMP)

catalyst	*E*_a_ (kcal mol^–1^)	Δ*G* ^⧧^_298K_ (kcal mol^–1^)	Δ*H* ^⧧^_298K_ (kcal mol^–1^)	Δ*S* ^⧧^_298K_ (cal mol^–1^ K^–1^)
(*R,S,S*)-**2**	7.80 ± 0.12	14.64 ± 0.17	7.16 ± 0.12	–25.08 ± 0.38
Co(TMP)	18.7 ± 2.3	24.5 ± 3.0	+18.0 ± 2.3	–22.0 ± 6.2

To the best of our knowledge, these diamine-bisoxazoline based Fe­(II) systems represent the first class of molecular catalysts for the C­(sp^3^)–H amination of (4-azidobutyl)­benzene as a substrate that do not suffer from product inhibition during catalytic turnover. Several follow-up kinetic experiments were conducted to investigate this behavior (Figure 6). Addition of 1 molar equivalent of exogenous pyridine (relative to the catalyst) to a catalytic reaction mixture of (4-azidobutyl)­benzene and (*R,S,S*)-**2** did not affect the reaction rate. Furthermore, addition of 2 equiv of TEMPO (with respect to the substrate) or 1 equiv of pyrrolidine product (relative to the catalyst) did not lead to any erosion of catalytic activity. Reaction monitoring with the related substrate (4-azidobutyl)­pyridine showed no effect on the internal pyridine functionality (Figure S10). In line with data presented in Table 1, THF-*d*_8_ as a solvent led to lower rate, likely due to polarity effects, but the reaction is not inhibited, i.e., THF-*d*_8_ does not act as a poison (see Supporting Information).

**Figure 6:**
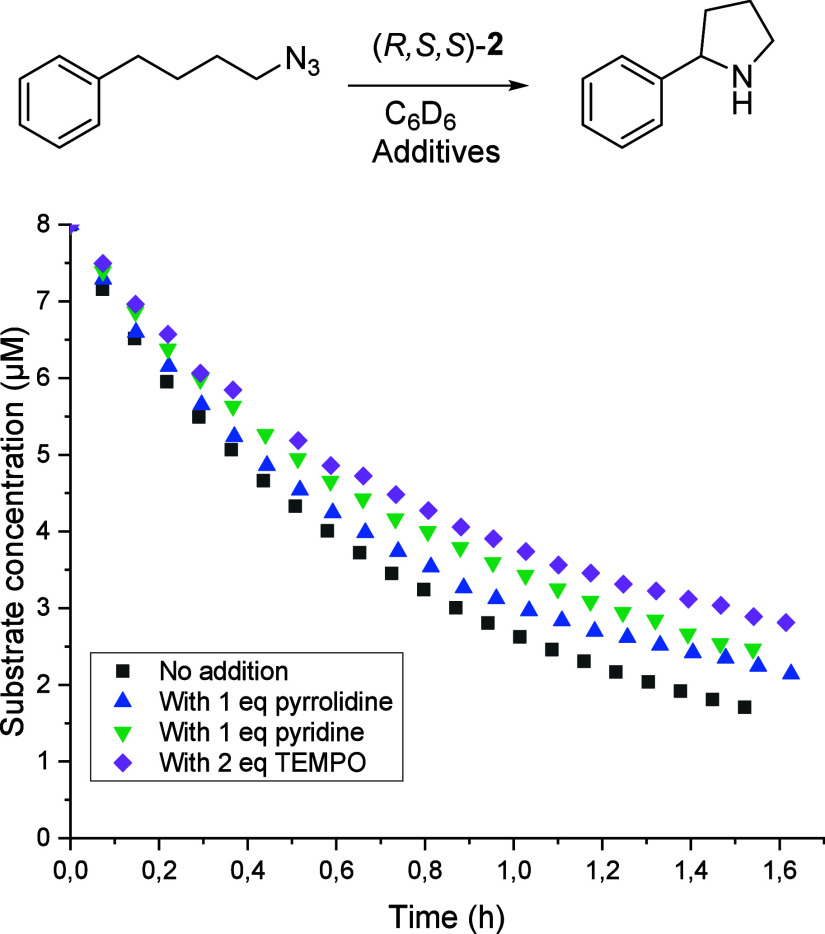
Effect of different solvents and additives on the C­(sp^3^)–H amination of the benchmark substrate (4-azidobutyl)­benzene at room temperature with 14 mM catalyst (*R,S,S*)-**2** and 0.008 mmol of substrate.

### DFT Calculations

To obtain more insight into the reaction mechanism of the asymmetric intramolecular Fe-mediated C­(sp^3^)–H amination, we investigated our catalyst system using density functional theory (DFT) calculations (OPBE-D3 ^26^/def2-TZVP) to elucidate a plausible reaction pathway and identify factors governing the enantioselectivity.

From the kinetic studies described above we already derived that the rate-determining step involves azide activation, which most logically should generate an iron nitrene intermediate, a mechanism consistent with prior reports.^5^ The quintet spin state was found to be the most stable for the (*R,S,S*)-**2** complex, with the triplet state lying 10.7 kcal mol^–1^ higher in energy and the open-shell singlet state being 18.0 kcal mol^–1^ higher. These computational results are consistent with the experimental data obtained from Evans’ method (μ_eff_ = 5.02). We were unable to optimize the geometry of the azide adduct. Any attempts to calculate this as a ground state structure led to spontaneous dissociation of the organic azide during the geometry optimization steps. Nevertheless, the transition state for concerted azide coordination and activation to generate **Int-1** could be located.

The DFT results thus point to concerted formation of the iron nitrene coordination bond concomitant with N_2_ dissociation upon approach of the organic azide to the Fe­(II) site. As could be expected for this process, DFT calculations using uncorrected gas phase entropy corrections overestimate the activation barrier (27 kcal mol^–1^) compared to the experimentally measured Gibbs free energy of activation (*vide supra*). However, upon correcting for the solution phase reference volume and additional entropy corrections applicable to catalytic reactions in solution (i.e., shifting from an ideal gas phase to a realistic solution phase with excess substrate),^27^ we arrive at an activation barrier of ∼20–21 kcal mol^–1^, which is closer to the experimental value (Δ*G* ^⧧^_298K_ = 14.64 ± 0.17 kcal mol^–1^). Irrespective of entropy, focusing on the activation enthalpies for nitrene formation, we note that the computed value (Δ*H* ^⧧^ = 12.04 kcal mol^–1^) is also a bit overestimated compared to the experimental value (Δ*H* ^⧧^ = 7.16 ± 0.12 kcal mol^–1^). The resulting iron nitrene complex **Int-1** has a quintet ground state (⟨*S* ^2^⟩ = 6.05) with a low lying (Δ*E* = +2.6 kcal mol^–1^) triplet state (⟨*S* ^2^⟩ = 2.05).

In both cases, the nitrene is positioned at the axial site of a trigonal bipyramidal complex with an Fe=N bond of 1.713 Å in the quintet state (*S* = 2; 1.659 Å in the triplet state). Spin density analysis of the quintet state iron nitrene complex reveals 58% spin population localized at the nitrene moiety. In the triplet state, the nitrene spin population is substantially lower (21%).

Both in the triplet and in the quintet spin state, hydrogen atom transfer (HAT) from the benzylic C–H bond to the iron nitrene moiety preferentially abstracts the pro-*S* benzylic hydrogen via **TS2-S** (see Figure 7). The **TS2-S** barrier is lowest on the quintet surface (Δ*G* ^⧧^ = 6.0 kcal mol^–1^, rC–H = 1.374 Å), favored by 4.3 kcal mol^–1^ over the pro-*R* pathway via **TS2-R** (Δ*G* ^⧧^ = 10.3 kcal mol^–1^, rC–H = 1.402 Å). The resulting benzylic radical intermediates could be optimized (with **Int2-S** at 4.1 kcal mol^–1^ being somewhat stabilized over **Int2-R** at 7.5 kcal mol^–1^). At the quintet surface, the subsequent radical rebound step is virtually barrierless and requires only a slight movement of the benzylic radical in the direction of the Fe-amide.

**Figure 7:**
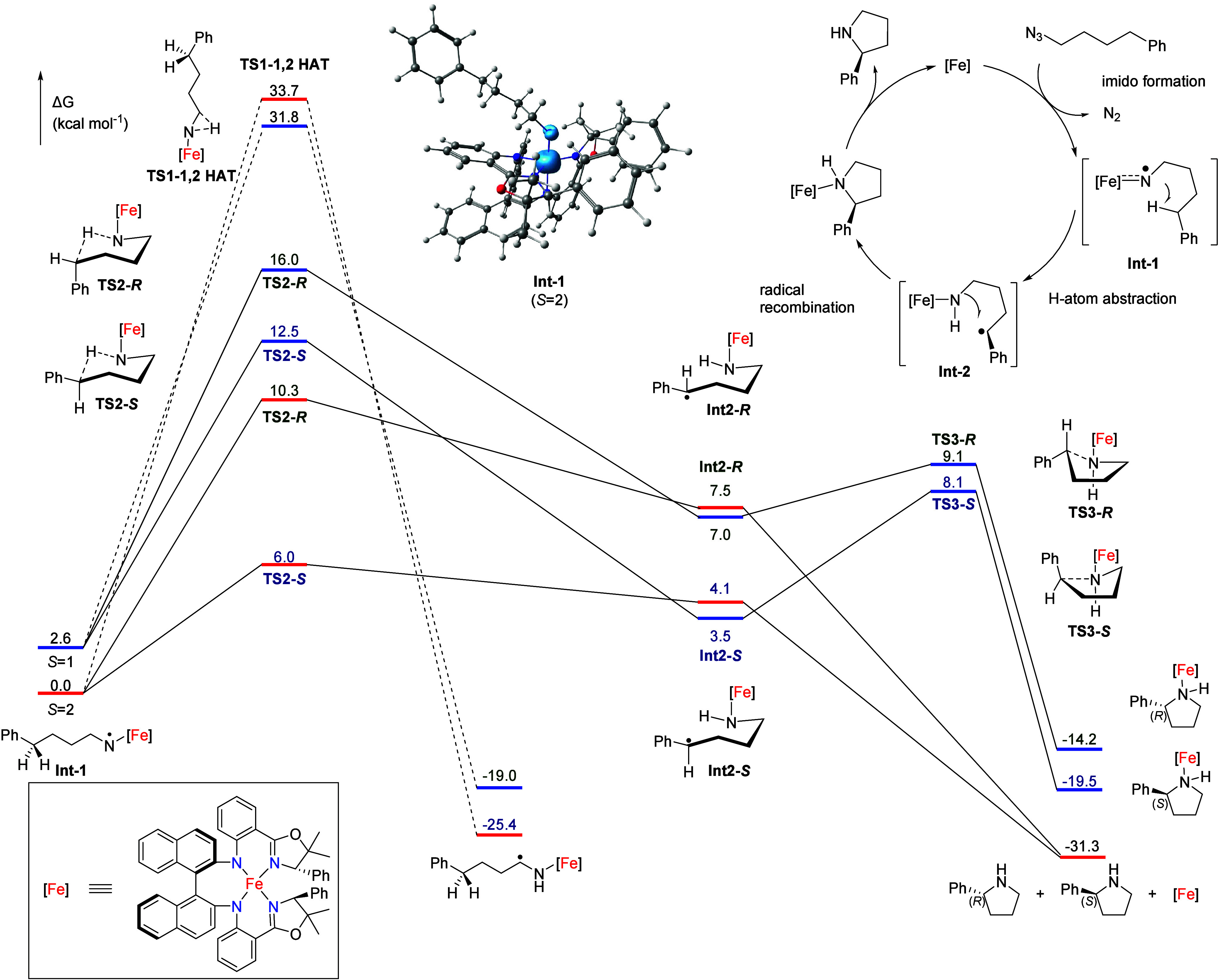
Computed reaction free energy profiles of the Fe-catalyzed asymmetric intramolecular nitrene transfer with (*R,S,S*)-**2** as catalyst (red for quintet and blue for triplet) and spin density plot of iron nitrene intermediate (top right).

Formation of the pyrrolidine product, which readily dissociates from the Fe catalyst (in line with the absence of any product inhibition), is highly exergonic (Δ*G* ^⧧^ = −31.3 kcal mol^–1^). These results explain the experimental observations that the catalysts do not suffer from product inhibition (and hence do not need Boc_2_O to trap the product) to sustain catalytic turnover.^28^ The enantio-determining step is likely the **TS2** HAT step, with a 4.3 kcal mol^–1^ energy difference between the transition states leading to the *S*- and *R*-enantiomers in favor of the former, which is also the experimentally preferred one (measured ee = 81%; the DFT calculated energy difference between **TS2-R** and **TS2-S** seems to be somewhat overestimated). This is supported by a linear transit calculation, whereby the Ph*C* ^•^H-*C*H_2_ bond was rotated in steps of 10° in either direction, resulting in high energy penalties (Figure S36), due to (i) the rigidity of the tetradentate ligand framework on the one side and (ii) the proximity of the N–H group on the other side. The high C–C bond rotational barrier indicates a stable intermediate conformation. As previously discussed, the 1,2-HAT of a metal nitrene to form an imine complex is typically the limiting factor preventing primary azides from undergoing C­(sp^3^)–H amination. We also examined this side product formation process computationally. The barriers for this process at both the quintet (Δ*G* ^⧧^ = 33.7 kcal mol^–1^) and triplet states (Δ*G* ^⧧^ = 29.2 kcal mol^–1^) are significantly higher than those for the 1,5-HAT step, leading to desired pyrrolidine formation, in support of the experimentally determined excellent chemoselectivity­(Figure 7).

### Substrate Scope

Given that catalyst (*R,S,S*)-**6** delivered the highest ee for the enantioselective intramolecular C­(sp^3^)–H amination of (4-azidobutyl)­benzene, we decided to examine its suitability to generate pyrrolidine building blocks present in various medicinally and biologically important molecules, such as larotrectinib, (*R*)-(+)-crispine A and a CDK_8_ inhibitor that is of interest for certain cancer treatments. Also, the precursors for an I_kur_ inhibitor (K-channel blocker), anticaprant (antidepressant) and nornicotine^20^ were prepared (Scheme 3).

**Scheme 3:**
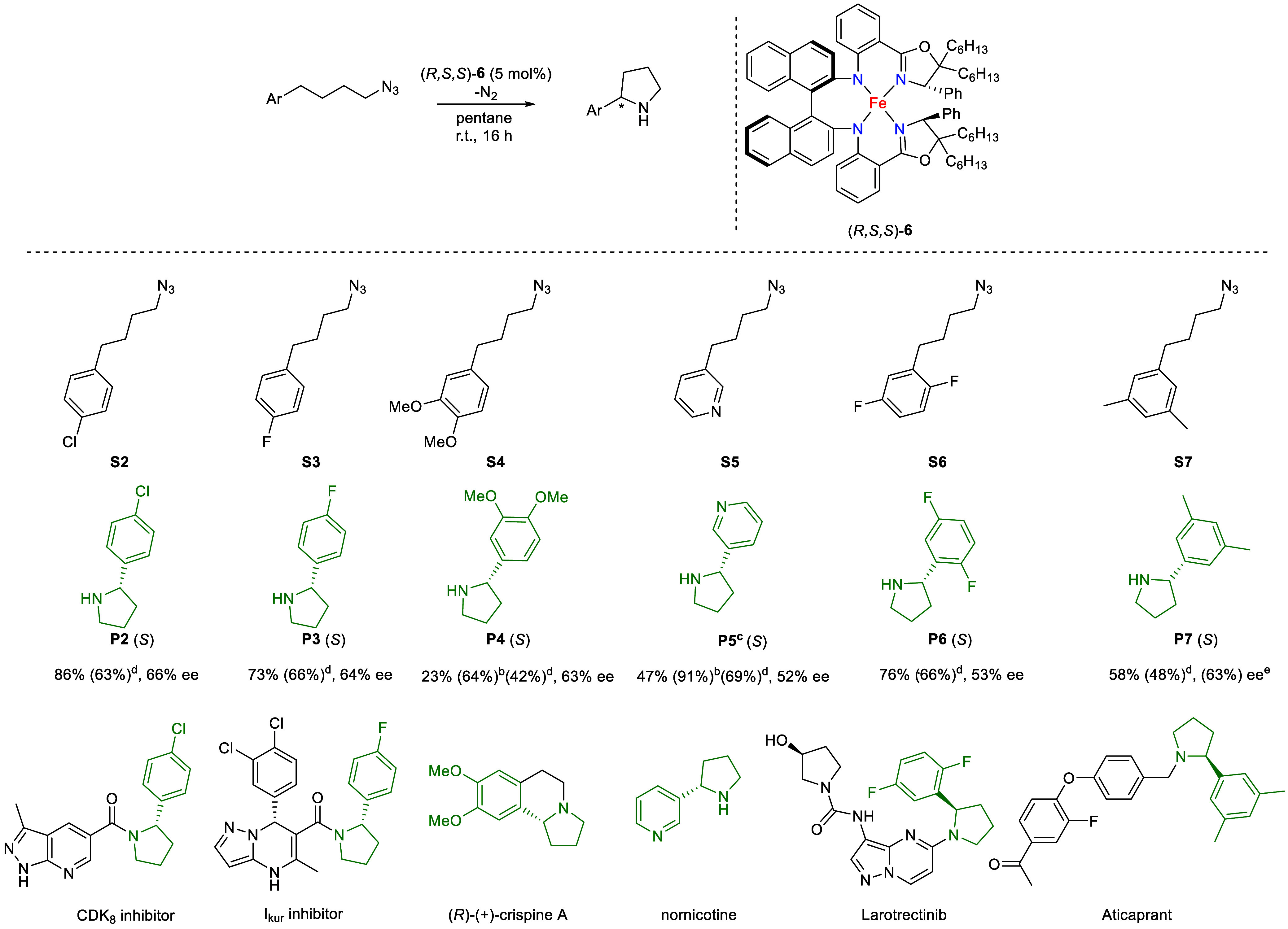
Catalytic C­(sp^3^)–H Amination of the Azide Substrates **S2**–**S7** ^a^

a Conditions: substrate (0.15 mmol) and catalyst (*R*,*S*,*S*)-**6** (5 mol %) were stirred in pentane (2.5 mL) at rt for 16 h unless stated otherwise. Determined ee values via chiral HPLC unless stated otherwise. Yields calculated via ^1^H NMR analysis with 1,3,5-trimethoxybenzene as internal standard. ^a^ee determined via chiral GC. ^b^Reaction performed with catalyst (*R*,*S*,*S*)-**6** (10 mol %) in benzene (0.6 mL) at rt for 16 h. ^c^ee obtained under standard conditions; n.d. means not determined. ^d^Isolated yield. ^e^Measured using the Mosher’s amide method.

Azides **S2**–**S5** were conveniently synthesized via a substitution reaction of the respective, commercially available halide or tosylate precursors with NaN_3_ in DMF at 80 °C over 16 h ^21^ (Figure S19). Access to the azides **S6** and **S7** either required a four-step synthesis under inert conditions^22^ or, alternatively, a two-step route involving a Grignard addition followed by a photochemical *anti*-Markovnikov hydroazidation, as developed by Carreira and co-workers.^23^ This latter step converts an alkene precursor into the desired azide by simple addition of NaN_3_ in the presence of FeCl_3_·6H_2_O under blue light irradiation (456 nm); the hydrate water molecules supply the hydrogen for this reaction.

These azides could be converted into the corresponding substituted pyrrolidine products **P2**-**P7**, although conversion of azides **S4**, **S5**, and **S7**, each featuring an electron-rich arene system, led to the respective pyrrolidine products in lower yields compared to the parent precursor und the standard reaction conditions, and with similar ee values as for the other examined azides, i.e., between 52–66% ee. The **S7** substrate was converted in 58% yield, but separation of the enantiomers proved problematic by both chiral GC and HPLC methods. For the **S4** and **S5** substrates, the lowest yields were observed, namely, 33% and 47%, respectively. Thereupon, alternative reaction conditions were devised, i.e., increasing both the concentration (0.6 mL of benzene solution) and loading (10 mol %) of the Fe-catalyst (*R,S,S*)-**6**. Satisfyingly, this improved the yields to 64% for the **S4** substrate and to 91% for the **S5** substrate.

Analysis of the crude reaction mixtures revealed that each of these catalytic reactions proceeded with high chemoselectivity as they cleanly afforded the corresponding pyrrolidines. Imine side-product, which could be formed via a 1,2-HAT-shift after initial azide activation and release of N_2_, was barely observed (trace amount) for **S2** to **S7**.^24^ This may be explained by polar effects induced by modifying the arene fragment, which can influence the bond dissociation free energy (BDFEs) of the benzylic C–H bonds that are relevant for the 1,5-HAT process.^25^

## Conclusions

In summary, we have unlocked the enantioselective iron catalyzed C­(sp^3^)–H amination of primary aliphatic azides under mild, protection-group-free conditions (at room temperature and without the requirement for Boc_2_O) utilizing a small family of new diamine-bisoxazoline tetradentate ligands based on the commercially available BINAM-backbone. The isolated Fe-complexes feature two different sites of chirality (stereochemistry of the two oxazoline fragments is the same in each complex), leading to a total of four different stereoisomeric Fe species for each ligand type. The resulting enantiomers exhibit comparable reactivity and structure, while the diastereomers show distinct geometric and physicochemical differences, as observed by NMR and UV–vis spectroscopy, X-ray diffraction, and Mössbauer spectroscopy. Only one of the enantiomeric pairs proved capable of catalytic C­(sp^3^)–H amination under the mild conditions noted above. Notably, geminal substitution at the 3-position of the oxazoline fragments plays a critical role: Switching from *gem*-H,H to *gem*-Me,Me led to a >30-fold increase in reactivity. Moreover, systematically extending the alkyl chain length at this geminal position led to significantly improved enantioselectivity (up to 81%) while maintaining high levels of chemoselectivity. A detailed kinetic study including kinetic isotope competition experiments suggested that azide activation to form an iron nitrene radical-like intermediate is the rate-determining step. DFT calculations support the formation of an iron nitrene radical intermediate following rate-limiting azide activation, with a stepwise hydrogen atom transfer (1,5-HAT) and radical rebound process at the quintet surface to form the chiral *N*-heterocyclic product, with the enantio-determining step likely involving a 1,5-HAT process. Moreover, the high-spin nature of the Fe­(II) species prevents product inhibition, thus enabling protection-group-free conditions, even in the presence of coordinating solvents as well as the conversion of substrates featuring donating functional groups (methoxy, pyridine). The 1,2-HAT pathway exhibits significantly higher energy barriers compared to 1,5-HAT, explaining the observed high chemoselectivity. Using our optimized azide synthesis protocol, this catalyst design allows for the first time the enantioselective C–H amination of a broader azide substrate scope, as highlighted by the conversion of six different synthetic precursors to bioactive molecules at room temperature, eliminating the requirement for Boc_2_O as an additive. We are convinced that these findings, based on conceptual ligand and catalyst development, will contribute to new directions in the field and stimulate more research into enantioselective nitrene transfer reactions using aliphatic azides.

## Supplementary Material



## Notes

Synthetic procedures, characterization details for all new compounds including NMR spectroscopic data, kinetic data, computational details, and crystallographic data (PDF)
