# Supplementary material for: Selective Chiral Diamine-bisoxazoline Iron(II) Catalysts for Pyrrolidine Formation via Intramolecular C(sp3)–H Amination of Aliphatic Azides
Source: J Am Chem Soc. 2025 Nov 18;147(48):44273–84. doi: 10.1021/jacs.5c14105 (PMC12679640; doi:10.1021/jacs.5c14105)
Supplement: Supplementary file 1 [file ja5c14105_si_001.pdf]

Supporting information for

Selective chiral diamine-bisoxazoline iron(II) catalysts for pyrrolidine formation via intramolecular C(sp^3)–H amination of aliphatic azides

Zhiyuan He[a], Stijn W. J. de Wit[a], Samuel M. van der Loo[a], Dorette S. Tromp[a], Alexis K. Bauer[b], Michael L. Neidig[b], Simon Mathew[a], Andreas W. Ehlers[a], Bas de Bruin,[a]* Jarl Ivar van der Vlugt[a,c]*

a Homogeneous, Supramolecular Catalysis, and Bio-Inspired Catalysis Group, van 't Hoff Institute for Molecular Sciences (HIMS), University of Amsterdam, 1098 XH Amsterdam, The Netherlands

b Inorganic Chemistry Laboratory, Department of Chemistry, University of Oxford, Oxford OX1 3QR, United Kingdom

c Bioinspired Coordination Chemistry and Homogeneous Catalysis Group, Institute of Chemistry, School of Mathematics and Science, Carl von Ossietzky University Oldenburg, 26129 Oldenburg, Germany

* Email: b.debruin@uva.nl; jarl.ivar.van.der.vlugt@uni-oldenburg.de

Contents

General information 1

General procedure for ligand synthesis 2

General procedure for the synthesis of the amino alcohols 2

General procedure for the synthesis of the alkyl-substituted benzamides 3

General procedure for the synthesis of the alkyl-substituted oxazolines 5

Synthesis of the substituted BINAM ligands 7

General procedure for the synthesis of the Fe-Complexes 13

Proposed dehydrogenation of oxazoline in complex 1 upon reaction with aryl azide 16

Indications for Fe-NR Species 17

In Situ Monitoring (NMR, UV-vis, IR-dip-probe) of Resting State 20

Kinetic study 22

VT-NMR Reaction Monitoring 24

Catalysis 37

General procedure of catalysis and characterization of products 37

Chiral GC spectra 46

Chiral HPLC spectra 63

NMR Spectra 69

Single-crystal X-ray diffraction (SC-XRD) 101

DFT calculations 104

References 143

General information

The aminoalcohols, amides, oxazolines[S1] and the ligands[S2] were initially synthesized following previously published procedures for 2,2‘-diaminobiphenyl derived backbones – subsequent modifications were undertaken with respect to catalyst optimization.

All other starting materials were purchased from commercial sources and were used as received. All reactions were performed under an inert atmosphere (N_2), using dry solvents, dried and freshly distilled prior to use. Et_2O and toluene were distilled from sodium wire. Dichloromethane was distilled from CaH_2. Automatic column chromatography was performed on a Büchi pure C-850 FlashPrep and fractions were detected by both UV-Vis & ELS. ^{1}H, ^{13}C and ^{19}F NMR spectra were recorded on a Bruker AVANCE NEO 300 MHz, Bruker AVANCE NEO 400 MHz, Bruker AVANCE NEO 500 MHz or a Bruker AVANCE III HD 300 MHz spectrometer and referenced to the solvent residual signal (5.32 ppm for CD_2Cl_2 and 7.26 ppm for $CDCl_3$) and converted to the TMS scale. High-resolution mass spectra (HRMS) were recorded with a TOF mass spectrometer of the type AccuTOF GC v 4g, JMST100GCV (JEOL, Japan). Solution magnetic susceptibilities were determined by the Evans method.[S3] For Mössbauer spectroscopy, all solid samples were prepared in an inert atmosphere glovebox equipped with a liquid nitrogen fill port to enable sample freezing to 77 K within the glovebox. Each solid sample was loaded into a Delrin Mössbauer sample cup for measurements and loaded under liquid nitrogen. Low temperature ^{57}Fe Mössbauer measurements were performed using a See Co. MS4 Mössbauer spectrometer integrated with a Janis SVT-400T He/N_2 cryostat for measurements at 80 K. Isomer shifts were determined relative to α-Fe at 298 K. All Mössbauer spectra were fit using the program WMoss (SeeCo). Errors of the fit analyses were the following: $\delta \pm 0.02$ mm/s and $\Delta E_Q \pm 3\%$. For multi-component fits the quantitation errors were ± 3% (e.g. 70 ± 3%).

General procedure for ligand synthesis

General procedure for the synthesis of the amino alcohols

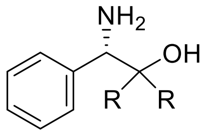

(*S*)-methyl-2-amino-2-phenylacetate hydrochloride (2.5 g, 12.40 mmol) was charged into a dry Schlenk flask and 20 mL of dry Et_2O was added, forming a white suspension. At 0 °C, the appropriate Grignard reagent (RMgBr) was added dropwise to the amino-ester (~1:6 ratio, 75 mmol, volume adjusted to the molarity of the Grignard reagent solution). After Grignard addition, the reaction mixture was warmed up to room temperature and stirred for 17 h. The reaction mixture was carefully neutralized with saturated NH_4Cl solution, poured in a beaker and 20 mL NaOH solution (2 M) was added. This mixture was stirred for 30 minutes and allowed to set. The Et_2O layer was separated from the salts and dried over $MgSO_4$ (part A). The mixture of salts, water and residue of Et_2O was diluted with some extra water and stirred to result in a milky suspension. This suspension was washed with Et_2O in a separation funnel (3×) and the Et_2O layers were collected and also dried over $MgSO_4$ (part B). Both Et_2O parts (A+B) were filtered, combined and evaporated to yield a yellow viscous oil. The formed amino alcohols were used without further purification.

(S)-1-amino-2-methyl-1-phenylpropan-2-ol (R=Me)
25 mL MeMgBr (3 M in Et_2O, 75 mmol). yellow oil
(S)-1-amino-2-ethyl-1-phenylbutan-2-ol (R=Et)
25 mL EtMgBr (3 M in Et_2O, 75 mmol). Yield: yellow oil 1.95 g 82%.
(S)-1-amino-2-propyl-1-phenylpentan-2-ol (R=Pr)
37 mL PrMgBr (2 M in Et_2O, 75 mmol). Yield: yellow oil 2.39 g 87%.
(S)-1-amino-2-butyl-1-phenylhexan-2-ol (R=Bu)
37 mL BuMgBr (2 M in Et_2O, 75 mmol). Yield: yellow oil 2.55 g 82.5%.
(S)-1-amino-2-hexyl-1-phenyloctan-2-ol (R=Hex)
37 mL HexMgBr (2 M in Et_2O, 75 mmol). Yield: yellow oil

General procedure for the synthesis of the alkyl-substituted benzamides

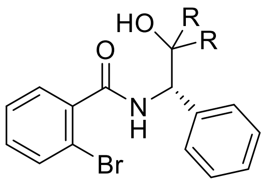

In a dry Schlenk, 2-bromobenzoyl chloride (1 molar equiv.) was dissolved in 20 mL dry CH_2Cl_2 and the mixture cooled to 0 °C. Dry NEt_3 (3.5 equiv.) and the required amino alcohol (1 molar equiv. dissolved in dry CH_2Cl_2) were added, the reaction mixture was slowly warmed up to room temperature and stirred for 17 h. The reaction mixture was extracted with sat. $NaHCO_3$ solution. The CH_2Cl_2 layers were combined and dried over $NaSO_4$. After filtration and evaporation, the residual yellow oil was purified via auto-column chromatography.

(S)-2-bromo-N-(2-hydroxy-2-methyl-1-phenylpropyl)benzamide (R=Me)
Synthesized according to the literature.[S1]

(S)-2-bromo-N-(2-hydroxy-2-ethyl-1-phenylbutyl)benzamide (R=Et)
1.95 g (10.09 mmol) (*S*)-1-amino-2-ethyl-1-phenylbutan-2-ol*;* 2.21 g (10.09 mmol) 2-bromobenzoyl chloride; 4.9 mL (3.58 g, 35.34 mmol) NEt_3 is used.

Column: Flash Silica, 12 g. Running scheme: t(min) 0: EtAc 0%, 5: EtAc 20%, 15: EtAc 40%, 5: EtAc 40%. Yield: 0.74 g 19.6%.

^{1}H NMR (CD_2Cl_2): δ 7.58 (dd, *J* = 7.8, 1.4 Hz, 1H, Ar*H*), 7.46 – 7.21 (m, 8H, Ar*H*), 7.11 (d, *J* = 9.5 Hz, 1H, N*H*), 5.06 (d, *J* = 8.9 Hz, 1H, C*H*), 1.77 (dh, *J* = 14.1, 7.3 Hz, 2H, CH_2), 1.35 (dq, *J* = 14.8, 7.4 Hz, 1H, CH_2), 1.11 (dq, *J* = 14.4, 7.3 Hz, 1H, CH_2), 0.96 (t, *J* = 7.5 Hz, 3H, CH_3), 0.82 (t, *J* = 7.4 Hz, 3H, CH_3).

^{13}C NMR (CD_2Cl_2): δ 166.58 (*C*O), 139.38 (Cq_{aryl}), 138.30 (Cq_{aryl}),133.22 (C_{aryl}H), 131.01(C_{aryl}H), 129.18 (C_{aryl}H), 128.57 (2×C_{aryl}H), 128.20 (2×C_{aryl}H), 127.47(C_{aryl}H), 127.46 (C_{aryl}H), 119.10 (Cq_{aryl}), 77.02 (*C*q), 58.33 (*C*H), 28.48 (CH_2), 27.41 (CH_2), 7.98 (CH_3), 7.19 (CH_3).

HRMS (ESI$^+$) *m/z* calcd for $C_{19}H_{22}BrNO_2$ $[M + H]^+$ 376.0912, found 376.0917.

(S)-2-bromo-N-(2-hydroxy-2-propyl-1-phenylpentyl)benzamide (R=Prop)
2.4 g (10.8 mmol) (*S*)-1-amino-2-propyl-1-phenylpentan-2-ol*;* 2.37 g (10.8 mmol) 2-bromobenzoyl chloride; 5.2 mL (3.80 g, 38.0 mmol) NEt_3 is used.

Column: Flash Silica, 12 g. Running scheme: t(min) 0: EtAc 0%, 5: EtAc 15%, 10: EtAc 15%,15: EtAc 20 %. Yield: 0,41 g white solid 9%.

^{1}H NMR (CD_2Cl_2): δ 7.63 (d, *J* = 7.6 Hz, 1H, Ar*H*), 7.48 – 7.27 (m, 8H, Ar*H*), 7.12 (d, *J* = 9.2 Hz, 1H, N*H*), 5.08 (d, *J* = 8.9 Hz, 1H, C*H*), 1.84 – 1.63 (m, 2H, CH_2), 1.44 – 1.23 (m, 4H, CH_2), 1.16 – 1.04 (m, 2H, CH_2), 1.01 (t, *J* = 7.2 Hz, 3H, CH_3), 0.86 – 0.77 (m, 3H, CH_3).

^{13}C NMR (CD_2Cl_2): δ 166.53 (*C*O), 139.38 (*C*q$_{aryl}$), 138.38 (*C*q$_{aryl}$), 133.22 (*C*$_{aryl}$H), 131.00 (*C*$_{aryl}$H), 129.14 (*C*$_{aryl}$H), 128.49 (2×*C*$_{aryl}$H), 128.22 (2×*C*$_{aryl}$H), 127.48 (2×*C*$_{aryl}$H), 119.08 (*C*q$_{aryl}$), 76.55 (*C*q), 58.89(*C*H), 38.99 (*C*H_2), 37.88 (*C*H_2), 17.10 (*C*H_2), 16.49 (*C*H_2), 14.39 (*C*H_3), 14.11 (*C*H_3).

HRMS (ESI$^+$) *m/z* calcd for $C_{21}H_{26}BrNO_2$ $[M + H]^+$ 404.1225, found 404.1219.

(S)-2-bromo-N-(2-hydroxy-2-butyl-1-phenylhexyl)benzamide (R=Bu)

2.55 g (10.23 mmol) (*S*)-1-amino-2-butyl-1-phenylhexan-2-ol*;* 2.25 g (10.23 mmol), 2-bromobenzoyl chloride; 4,96 mL (3.62 g, 36,0 mmol) NEt_3 is used.

Column: Flash Silica, 12 g. Running scheme: t(min) 0: EtAc 0 %, 5: EtAc 10 %, 10: EtAc 10%, 15: EtAc 15 %. Yield: 1.70 g yellow oil, 38 %.

^1H NMR (CD_2Cl_2): δ 7.72 – 7.24 (m, 9H, Ar*H*), 7.17 (d, *J* = 8.9 Hz, 1H, N*H*), 5.08 (d, *J* = 8.9 Hz, 1H, C*H*), 1.83 – 1.71 (m, 4H, 2×CH_2), 1.46 – 1.05 (m, 8H, 4×CH_2), 1.04 – 0.95 (m, 3H, CH_3), 0.90 – 0.81 (m, 3H, CH_3).

^{13}C NMR (CD_2Cl_2): δ 166.56 (*C*O), 139.44(*C*q$_{aryl}$) , 138.38 (*C*q$_{aryl}$), 133.21 (*C*$_{aryl}$H), 130.99 (*C*$_{aryl}$H), 128.58 (3×*C*$_{aryl}$H), 128.18 (2×*C*$_{aryl}$H), 127.47 (2×*C*$_{aryl}$H), 119.10 (*C*q$_{aryl}$), 76.52 (*C*q), 58.98 (*C*H), 36,36 (*C*H_2), 35,29 (*C*H_2), 25.87(*C*H_2), 25.33 (*C*H_2), 23.26 (*C*H_2), 23.01 (*C*H_2), 13.89 (*C*H_3), 13.71(*C*H_3).

HRMS (ESI$^+$)*m/z* calcd for $C_{23}H_{30}BrNO_2$ $[M + H]^+$ 432.1538, found 432.1536.

(S)-2-bromo-N-(2-hydroxy-2-hexyl-1-phenyloctyl)benzamide (R=Hex)

4.53 g (14.8 mmol) (*S*)-7-(amino(phenyl)methyl)tridecan-7-ol, 1.94 ml (14.8 mmol) 2-bromobenzoyl chloride, 7.21 ml (5.26 g, 51.9 mmol) NEt_3. The reaction was quenched with demineralized water instead of sat. $NaHCO_3$ solution. The product was obtained as a yellow wax and used for the next step without purification.

General procedure for the synthesis of the alkyl-substituted oxazolines

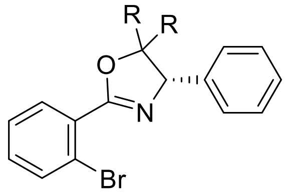

In a dry Schlenk loaded with molsieves (4 Å) and 10 mL xylene the required benzamide (1 equiv), also dissolved in xylene, was added. While stirring the reaction mixture, 0,5-1 mL (0.5-1.6 equiv.) of tetraisopropyl orthotitanate was added and the reaction mixture heated at 135 °C and stirred for 17 h. After cooling down, the reaction mixture was filtered and extracted with Et_2O. The combined Et_2O layers were dried on $MgSO_4$, filtered and evaporated. The crude product was purified by column chromatography by auto column chromatography with cyclohexane/ethyl acetate as eluent affording the product as an orange, yellow or colourless oil or off-white to white solid.

(S)-2-(2-bromophenyl)-5,5-dimethyl-4-phenyl-4,5-dihydrooxazole (R=Me, **I2***)*
Synthesized according to the literature[S1]

(S)-2-(2-bromophenyl)-5,5-diethyl-4-phenyl-4,5-dihydrooxazole (R=Et, **I3***)*
0.61 g (1.64 mmol) (*S*)-2-bromo-*N*-(2-hydroxy-2-ethyl-1-phenylbutyl) benzamide; 0.3 mL (1.00 mmol) $Ti(O^iPr)_4$ is used.

Column: Flash Silica, 12 g. Running scheme: t(min) 0: EtAc 0 %, 5: EtAc 0 %, 0: EtAc 3%, 20: EtAc 3%. Yield: colorless oil 0.16 g 27.6%.

^{1}H NMR (CD_2Cl_2): δ 7.89 (d, J = 9.5 Hz, 1H, ArH), 7.76 (d, J = 7.9 Hz, 1H, ArH), 7.56 – 7.28 (m, 6H, ArH), 5.27 (s, 1H, CH), 2.03 (dh, J = 28.9, 7.4 Hz, 2H, CH_2), 1.46 (dq, J = 14.8, 7.4 Hz, 1H, CH_2), 1.19 (m, J = 7.3 Hz, 1H + 3H, CH_2 + CH_3 overlapping), 0.84 (t, J = 7,5 Hz, 3H, CH_3).

^{13}C NMR (CD_2Cl_2): δ 162.99 (NCO), 138.95(Cq$_{aryl}$), 133.91(2×C_{aryl}H), 131.63 (2×C_{aryl}H)), 131.51 (C_{aryl}H), 130.61 (C_{aryl}H), 128.10 (C_{aryl}H), 128.04 (C_{aryl}H), 127.38 (C_{aryl}H), 127.23 (C_{aryl}H), 121.68 (Cq$_{aryl}$), 92.31 (Cq), 76.37 (CH), 30.00(CH$_2$), 27.46 (CH_2), 8.06 (CH$_3$), 7.53 (CH$_3$).

HRMS (ESI^+) *m/z* calcd for $C_{19}H_{20}BrNO$ $[M + H]^+$ 358.0807, found 358.0801.

(S)-2-(2-bromophenyl)-5,5-dipropyl-4-phenyl-4,5-dihydrooxazole (R=Prop, **I4***)*
0.39 g (0.97 mmol) (*S*)-2-bromo-*N*-(2-hydroxy-2-propyl-1-phenylpentyl) benzamide; 0.5 mL (1,67 mmol) $Ti(O^iPr)_4$ is used.

Column: Flash Silica, 12 g. Running scheme: t(min) 0: EtAc 0%, 5: EtAc 0%, 10: EtAc 3%, 10: EtAc 3%. Yield: colorless oil 0.20 g 55%.

^{1}H NMR (CD_2Cl_2): δ 7.92 (d, J = 7.6 Hz, 1H, ArH), 7.78 (d, J = 6.7 Hz, 1H, ArH), 7.53 – 7.34 (m, 7H, ArH), 5.29 (s, 1H, CH), 2.01 (td, J = 9.6, 5.5 Hz, 2H, CCH_2), 1.79 – 1.57 (m, 2H, CH_2), 1.41 (t, J = 10.5 Hz, 2H, CCH_2), 1.35 – 1.20 (m, 2H, CH_2), 1.15 (t, J = 7.3 Hz, 3H, CH_3), 0.77 (t, J = 7.1 Hz, 3H, CH_3).

^{13}C NMR (CD_2Cl_2): δ 162.96 (N*C*O), 138.93(*C*q_{aryl}), 133.96(*C*$_{aryl}$H), 131.64(*C*$_{aryl}$H), 131.56 (*C*$_{aryl}$H), 130.61 (*C*q_{aryl}), 128.16 (2×*C*$_{aryl}$H), 128.04 (2×*C*$_{aryl}$H), 127.41 (*C*$_{aryl}$H), 127.23 (*C*$_{aryl}$H), 121.72(*C*q_{aryl}), 91.88 (*C*q), 77.10 (*C*H), 40.54 (C*C*H_2), 37.12 (C*C*H_2), 17.30 (*C*H_2), 16.80 (*C*H_2), 14.45 (*C*H_3), 14.33 (*C*H_3). HRMS (ESI$^+$) *m/z* calcd for $C_{21}H_{24}BrNO$ [M + H]$^+$ 386.1119, found 386.1123.

(S)-2-(2-bromophenyl)-5,5-dibutyl-4-phenyl-4,5-dihydrooxazole (R=Bu, **I5***)* 1.09 g (2.31 mmol) (*S*)-2-bromo-*N*-(2-hydroxy-2-butyl-1-phenylhexyl)benzamide; 1 mL (3.34 mmol) Ti(O^{i}Pr)$_4$ is used.

Column: Flash Silica, 12 g. Running scheme: t(min) 0: EtAc 0 %, 5: EtAc 0%, 10: EtAc 3%, 10: EtAc 3%. Yield: l-yellow oil 0,48 g 49.7%.

^1H NMR (CD_2Cl_2): δ 8.00 (d, *J* = 9.5 Hz, 1H, Ar*H*), 7.82 (d, *J* = 7.9 Hz, 1H, Ar*H*), 7.63 – 7.28 (m, 7H, Ar*H*), 5.38 (s, 1H, C*H*), 2.26 – 1.96 (m, 2H, C*H*$_2$), 1.83 – 1.23 (m, 10H, C*H*$_2$), 1.18 (t, *J* = 7.1 Hz, 3H, C*H*$_3$), 0.89 (t, *J* = 7.1 Hz, 3H, C*H*$_3$).

^{13}C NMR (CD_2Cl_2): δ 162.98 (N*C*O), 139.04 (*C*q_{aryl}), 134.06 (*C*$_{aryl}$H), 131.67 (2×*C*$_{aryl}$H), 130.72 (*C*q_{aryl}), 128.29 (2×*C*$_{aryl}$H), 128.12 (2×*C*$_{aryl}$H), 127.51 (*C*$_{aryl}$H), 127.28 (*C*$_{aryl}$H), 121.88 (*C*q_{aryl}), 91.93 (*C*q), 77.30 (*C*H), 38.21 (*C*H_2), 34.65 (*C*H_2), 26.17 (*C*H_2), 25.67 (*C*H_2), 23,56 (*C*H_2), 23.15 (*C*H_2), 14.18 (*C*H_3), 13.86 (*C*H_3).

HRMS (ESI$^+$) *m/z* calcd for $C_{23}H_{28}BrNO$ [M + H]$^+$ 414.1433, found 414.1432.

(S)-2-(2-bromophenyl)-5,5-dihexyl-4-phenyl-4,5-dihydrooxazole (R=Hex, **I6***)* (*S*)-2-bromo-*N*-(2-hydroxy-2-hexyl-1-phenyloctyl)benzamide; 0.5 mL (1.67 mmol) Ti(O^{i}Pr)$_4$ is used.

Column: Flash Silica, 12 g. Running scheme t(min) 0: EtAc 0%, 5: EtAc 2%, 20: EtAc 20%. Product collection in t5-20. Yield: orange oil 2.33 g 33.5% compared to 4.53 g amino alcohol).

^1H NMR (CD_2Cl_2): δ 7.83 (dd, *J* = 7.6, 1.9 Hz, 1H, Ar*H*), 7.74 (dd, *J* = 7.8, 1.5 Hz, 1H, Ar*H*), 7.55 – 7.25 (m, 7H, Ar*H*), 5.19 (s, 1H, C*H*), 2.04 – 1.84 (m, 4H, C*H*$_2$), 1.64 – 1.03 (m, 16H, C*H*$_2$), 0.97 (t, *J* = 7.1 Hz, 3H, C*H*$_3$) 0.84 (t, *J* = 7.1 Hz, 3H, C*H*$_3$).

^{13}C NMR (CD_2Cl_2): δ 162.94 (N*C*O), 138.84 (*C*q_{aryl}), 133.85 (*C*$_{aryl}$H), 131.55 (*C*$_{aryl}$H), 131.45 (*C*$_{aryl}$H), 130.60 (*C*q_{aryl}), 128.13(2×*C*$_{aryl}$H), 127.94 (2×*C*$_{aryl}$H), 127.33 (*C*$_{aryl}$H), 127.17 (*C*$_{aryl}$H), 121.60 (*C*q_{aryl}), 91.99 (*Cq*), 77.06 (*C*H), 38.27 (*C*H_2), 34.75 (*C*H_2), 31.77 (*C*H_2), 31.52 (*C*H_2), 29.70 (*C*H_2), 29.55 (*C*H_2), 23.76 (*C*H_2), 23.24 (*C*H_2), 22.65 (*C*H_2), 22.44 (*C*H_2), 13.85 (*C*H_3), 13.74 (*C*H_3).

HRMS (ESI$^+$) *m/z* calcd for $C_{27}H_{36}BrNO$ [M + H]$^+$ 470.2059, found 470.2051.

Synthesis of the substituted BINAM ligands

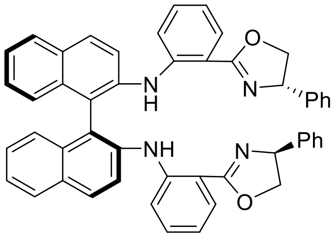

***(R,S,S)* *H/H* ligand (*R*=*H*, L1)**

In a dry Schlenk flask, (*R*)-1,1'-binaphthyl-2,2'-diamine (138 mg, 0.485 mmol), NaOtBu (139 mg, 1.45 mmol), Pd_2dba_3 (18.1 mg, 0.0198 mmol), *rac*-BINAP (25.5 mg, 0.0410 mmol) and (*S*)-oxazoline **I1** (294 mg, 0.973 mmol) were dissolved in toluene (15 mL) and heated at 115 °C overnight (16 h) – color changed from dark purple to dark brown after 5-10 minutes. The reaction mixture was concentrated by rotary evaporation and purified by column chromatography over silica with 0-2% EtOAc/cyclohexane to give (*R*,*S*,*S*)-**L1** as an off-white solid (130 mg, 0.179 mmol, 37% yield).

^{1}H NMR (CD_2Cl_2) δ 10.09 (s, 2H, N*H*), 7.76-6.67 (m, 30H, Ar*H)*, 4.76 (t, *J* = 9.1 Hz, 2H, C*H*), 4.44 (t, *J* = 9.1 Hz, 2H, CH_2), 3.89 (t, *J* = 8.3 Hz, 2H, CH_2).

^{13}C NMR (CD_2Cl_2) δ 164.12 (N*C*O), 144.71 (*C*q_{aryl}), 142.20 (*C*q_{aryl}), 138.45 (*C*q_{aryl}), 133.92 (*C*q_{aryl}), 131.58 (C_{aryl}H), 130.18 (C_{aryl}H), 129.61 (C_{aryl}H), 128.32 (C_{aryl}H), 128.15 (C_{aryl}H), 128.06 (C_{aryl}H), 126.91 (C_{aryl}H), 126.33 (C_{aryl}H), 126.09 (C_{aryl}H), 124.53 (C_{aryl}H), 123.62 (C_{aryl}H), 122.43 (*C*q_{aryl}), 120.45 (C_{aryl}H), 116.88 (C_{aryl}H), 112.93 (C_{aryl}H), 110.55 (*C*q_{aryl}), 72.87 (*C*H_2), 69.73 (*C*H).

For the synthesis of ligands **L2-L6**, this procedure was modified and optimized by catalyst screening:

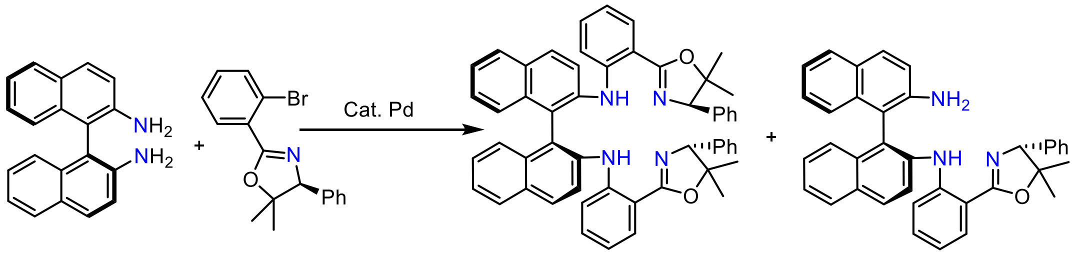

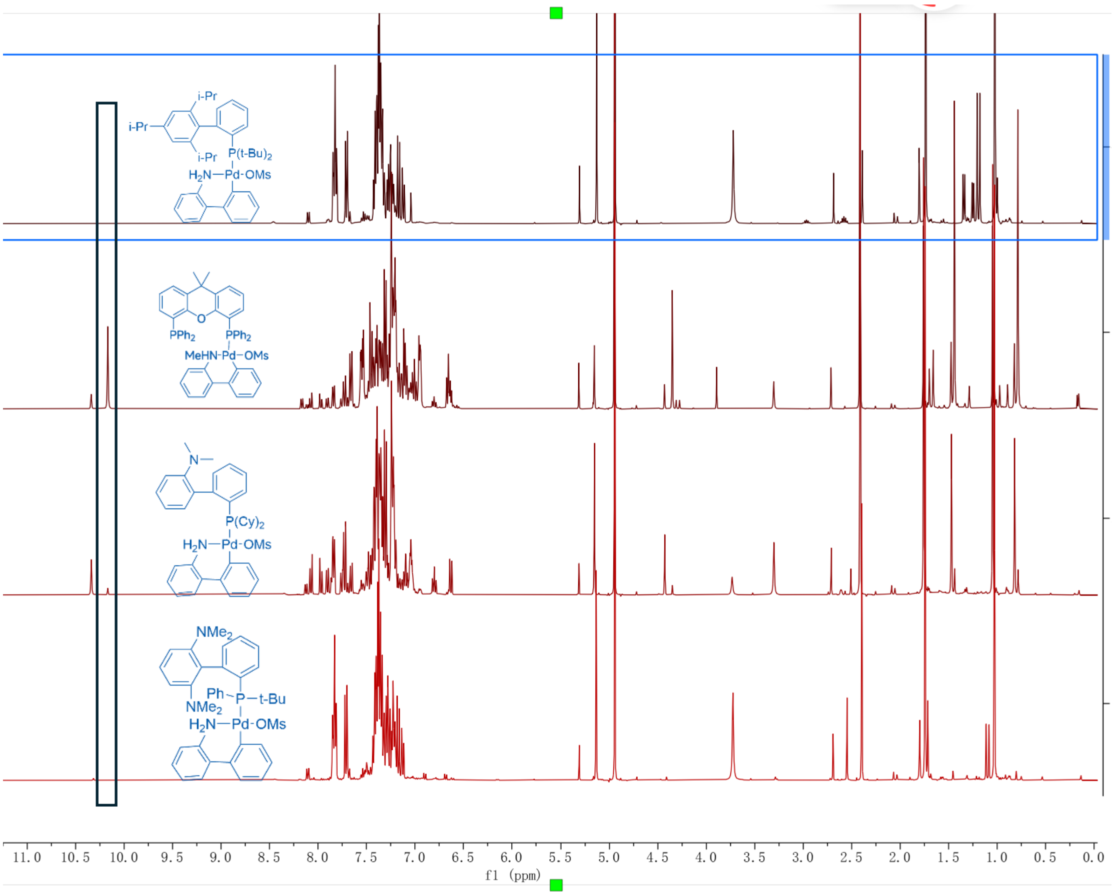

Figure S1. catalyst screening for ligand synthesis.

Xantphos Pd G4 was found to be the best catalyst and this system is used for further synthesis.

Optimized general procedure for L2-L6:

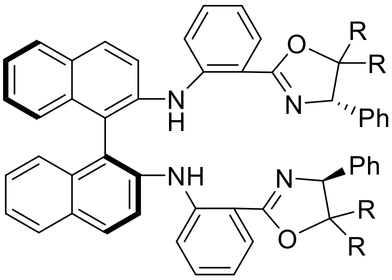

In a dry Schlenk flask, *R*)-1,1'-binaphthyl-2,2'-diamine (1 equiv), Xantphos Pd G4 catalyst (0.2 equiv), sodium t-butoxide (3 equiv), (*S*)-oxazoline precursor **IX** (3 equiv), and dry toluene (20 mL) were added, and the reaction mixture was heated at 110 °C overnight (16 h), after which all volatile materials were removed under vacuum, and the crude product was purified directly by auto column chromatography with cyclohexane/ ethyl acetate as eluent affording the product.

***(R,S,S) Me/Me** ligand (R=Me,* ***L2**)*

(*R*)-1,1'-binaphthyl-2,2'-diamine (0.150 g, 0.59 mmol), (*S*)-oxazoline, (0.508 g, 1.54 mmol) Xantphos Pd G4 (0.099 g, 0.10 mmol) and sodium t-butoxide (0.148 g, 1.54 mmol) were used.

Column: Flash Silica, 12 g. Running scheme: t(min) 0: EtAc 0 %, 8: EtAc 0 %, 0: EtAc 2%, 14: EtAc 2 %, 0: EtAc 3%, 10: EtAc 3 %. Yield: white solid 0.321 g, 78 %.

^{1}H NMR (CD_2Cl_2) δ 10.27 (s, 2H, N*H*), 7.73 – 6.64 (m, 30H, Ar*H*), 4.41 (s, 2H, C*H*), 1.48 (s, 6H, CH_3), 0.82 (s, 6H, CH_3).

^{13}C NMR (CD_2Cl_2) δ 163.18 (N*C*O), 144.93 (Cq_{aryl}), 138.90 (Cq_{aryl}), 138.57 (Cq_{aryl}), 133.96 (Cq_{aryl}), 131.45 (C_{aryl}H), 130.18 (C_{aryl}H), 129.56 (C_{aryl}H), 128.28 (C_{aryl}H), 128.05 (C_{aryl}H), 127.82 (C_{aryl}H), 126.94 (C_{aryl}H), 126.85 (Cq_{aryl}) 126.01 (Cq_{aryl}), 124.52 (C_{aryl}H), 123.54 (C_{aryl}H), 122.52 (Cq_{aryl}), 120.50 (C_{aryl}H), 116.84 (C_{aryl}H), 113.04 (C_{aryl}H), 111.20 (Cq_{aryl}), 84.97 (*C*q), 77.95 (*C*H), 28.22 (CH_3), 23.40 (CH_3).

HRMS (FD^+) *m/z* calcd for $C_{54}H_{46}N_4O_2$ [$M^{\bullet +}$] 782.3621, found 782.3598.

(S,S,S) Me/Me** ligand (R=Me,* **L2)*

(*S*)-1,1'-binaphthyl-2,2'-diamine (0.115 g, 0.40 mmol), (*S*)-oxazoline **I2** (0.400 g, 1.11 mmol), Xantphos Pd G4 (0.078 g, 0.08 mmol) and sodium t-butoxide (0.116 g, 1.20 mmol) were used.

Column: Flash Silica, 25 g. Running scheme: t(min) 0: EtAc 0 %, 8: EtAc 0 %, 0: EtAc 2%, 14: EtAc 2 %, 0: EtAc 3%, 10: EtAc 3 %. Yield: white solid 0.252 g, 80%.

^{1}H NMR (CD_2Cl_2) δ 10.03 (s, 2H, N*H*), 7.92 – 6.56 (m, 30H, Ar*H*), 4.47 (s, 2H, C*H*), 1.47 (s, 6H, CH_3), 0.73 (s, 6H, CH_3).

^{13}C NMR (CD_2Cl_2) δ 163.06 (N*C*O), 145.31 (Cq_{aryl}), 138.96 (Cq_{aryl}), 137.97 (Cq_{aryl}), 134.11 (Cq_{aryl}), 131.41 (C_{aryl}H), 130.25 (Cq_{aryl}), 129.61 (C_{aryl}H), 128.41 (C_{aryl}H), 127.88 (C_{aryl}H), 127.63 (C_{aryl}H), 127.06 (C_{aryl}H), 126.99 (Cq_{aryl}), 126.28 (Cq_{aryl}), 124.71 (C_{aryl}H), 124.04 (C_{aryl}H), 123.95 (Cq_{aryl}), 121.46 (C_{aryl}H), 116.79 (C_{aryl}H), 113.34 (C_{aryl}H), 110.96 (Cq_{aryl}), 84.94 (*C*q), 77.96 (*C*H), 28.42(CH_3), 23.39 (CH_3).

HRMS (FD^+) *m/z* calcd for $C_{54}H_{46}N_4O_2$ [$M^{\bullet +}$] 782.3621, found 782.3639.

The (S,R,R) and *(R,S,S)* stereomers were synthesized using the same procedure.

(S,R,R) Me/Me** ligand (R=Me,* **L2)*

^{1}H NMR (CD_2Cl_2) δ 10.22 (s, 2H, N*H*), 7.75 – 6.50 (m, 30H, Ar*H*), 4.37 (s, 2H, C*H*), 1.44 (s, 6H, CH_3), 0.78 (s, 3H, CH_3).

^{13}C NMR (CD_2Cl_2) δ 163.13 (N*C*O), 144.88 (Cq_{aryl}), 138.86 (Cq_{aryl}), 138.53 (Cq_{aryl}), 133.91(Cq_{aryl}), 131.40 (C_{aryl}H), 130.13 (Cq_{aryl}), 129.51 (C_{aryl}H), 128.24 (C_{aryl}H), 128.01 (C_{aryl}H), 127.77 (C_{aryl}H), 126.91 (C_{aryl}H), 126.81 (Cq_{aryl}),125.96 (Cq_{aryl}), 124.46 (C_{aryl}H), 123.49 (C_{aryl}H), 122.46 (Cq_{aryl}), 120.46 (C_{aryl}H), 116.79 (C_{aryl}H), 113.00 (C_{aryl}H), 111.16 (Cq_{aryl}), 84.93 (*C*q), 77.90 (*C*H), 28.17 (CH_3), 23.34 (CH_3).

HRMS (FD^+) *m/z* calcd for $C_{54}H_{46}N_4O_2$ [$M^{\bullet +}$] 782.3621, found 782.3614.

*(**R,R,R**) **Me/Me** ligand (R=Me,* **L2***)*

^{1}H NMR (CD_2Cl_2) δ 10.02 (s, 2H, N*H*), 7.89 – 6.54 (m, 30H, Ar*H*), 4.46 (s, 2H, C*H*), 1.47 (s, 6H, CH_3), 0.73 (s, 6H, CH_3).

^{13}C NMR (CD_2Cl_2) δ 163.05(N*C*O), 145.30 (*C*q_{aryl}), 138.96 (*C*q_{aryl}), 137.96 (*C*q_{aryl}), 134.11(*C*q_{aryl}), 131.40 (C_{aryl}H), 130.25 (*C*q_{aryl}), 129.60 (C_{aryl}H), 128.40 (C_{aryl}H), 127.87 (C_{aryl}H), 127.62 (C_{aryl}H), 127.05 (C_{aryl}H), 126.98 (*C*q_{aryl})126.27 (*C*q_{aryl}), 124.70 (C_{aryl}H), 124.03 (C_{aryl}H), 123.94 (*C*q_{aryl}), 121.45 (C_{aryl}H), 116.78 (C_{aryl}H), 113.33 (C_{aryl}H), 110.95 (*C*q_{aryl}), 84.94 (*C*q), 77.95 (*C*H), 28.40 (*C*H_3), 23.38 (*C*H_3).

HRMS (FD^+) *m/z* calcd for $C_{54}H_{46}N_4O_2$ [$M^{\bullet+}$] 782.3621, found 782.3605.

**isomer**	**δ NH**	**δ CH**	**δ CH_3**
R,S,S	10.27	4.41	1.48<br>0.82
S,R,R	10.22	4.37	1.44<br>0.78
S,S,S	10.03	4.47	1.47<br>0.73
R,R,R	10.02	4.46	1.47<br>0.73

Table S1. ^{1}H NMR shifts of different isomers.

Et/Et *ligand (R=Et,* **L3***)*

(*R*)-1,1'-binaphthyl-2,2'-diamine (0.110 g, 0.38 mmol), (*S*)-oxazoline **I3** (0.418 g, 1.17 mmol), Xantphos Pd G4 (0.072 g, 0.07 mmol) and sodium t-butoxide (0.115 g, 1.20 mmol) were used.

Column: Flash Silica, 25 g. Running scheme: t(min) 0: EtAc 0 %, 8: EtAc 0 %, 0: EtAc 2%, 14: EtAc 2 %, 0: EtAc 3%, 10: EtAc 3 %. Yield: white solid 0,212 g, 65 %.

^{1}H NMR (CD_2Cl_2) δ 10.19 (s, 2H, N*H*), 7.70 – 6.65 (m, 30H, Ar*H*), 4.37 (s, 2H, C*H*) 1.64 (m, 4H, CH_2), 1.10 (m, 2H, CH_2), 0.98 (m, 2H, CH_2), 0.87 (t, *J* = 7.5 Hz, 6H, CH_3), 0.71 (t, *J* = 7.5 Hz, 6H, CH_3).

^{13}C NMR (CD_2Cl_2) δ 162.96 (N*C*O), 144.80 (*C*q_{aryl}), 138.86 (*C*q_{aryl}), 138.54 (*C*q_{aryl}), 134.00 (*C*q_{aryl}), 131.28 (C_{aryl}H), 130.06 (*C*q_{aryl}), 129.49 (C_{aryl}H), 128.31 (C_{aryl}H), 128.08 (C_{aryl}H), 127.70 (C_{aryl}H), 127.59 (C_{aryl}H), 126.72 (*C*q_{aryl}), 125.91 (C_{aryl}H), 124.46 (C_{aryl}H), 123.35 (ArCH), 122.27 (*C*q_{aryl}), 120.14 (ArCH), 116.79 (ArCH), 113.27 (ArCH), 111.27 (*C*q_{aryl}), 89.28 (*C*q), 75.59 (*C*H) 29.52 (*C*H_2), 27.15 (*C*H_2), 7.87(*C*H_3), 7.27(*C*H_3).

HRMS (FD^+) *m/z* calcd for $C_{58}H_{54}N_4O_2$ [$M^{\bullet+}$] 838.4247, found 838.4260.

***^{n}Pr/^{n}Pr** ligand (R=^{n}Pr, **L4**)*

(*R*)-1,1'-binaphthyl-2,2'-diamine (0.332 g, 1.16 mmol), (*S*)-oxazoline **I4** (1.348 g, 3.49 mmol), Xantphos Pd G4 (0.225 g, 0.23 mmol) and sodium t-butoxide (0.340 g, 3.49 mmol) were used.

Column: Flash Silica, 25 g. Running scheme: t(min) 0: EtAc 0 %, 8: EtAc 0 %, 0: EtAc 2%, 14: EtAc 2 %, 0: EtAc 3%, 10: EtAc 3 %. Yield: white solid 0,620 g, 60 %.

^{1}H NMR (CD_2Cl_2) δ 10.20 (s, 2H, N*H*), 7.91 – 6.36 (m, 30H, Ar*H*), 4.35 (s, 2H, C*H*), 1.67 – 1.52 (m, 4H, CH_2), 1.44 – 1.02 (m, 12H, CH_2), 0.99 (t, *J* = 7.1 Hz, 6H, CH_3), 0.65 (t, *J* = 7.1 Hz, 6H, CH_3).

^{13}C NMR (CD_2Cl_2) δ 162.96 (N*C*O), 144.78 (Cq_{aryl}), 138.81 (Cq_{aryl}), 138.56 (Cq_{aryl}), 134.01 (Cq_{aryl}), 131.28 (C_{aryl}H), 130.04 (Cq_{aryl}), 129.50 (C_{aryl}H), 128.30 (C_{aryl}H), 128.11 (C_{aryl}H), 127.72 (C_{aryl}H), 127.57 (C_{aryl}H), 126.74 (Cq_{aryl}),125.91 (C_{aryl}H), 124.47 (C_{aryl}H), 123.33 (C_{aryl}H), 122.17 (Cq_{aryl}), 120.05 (C_{aryl}H), 116.81(C_{aryl}H) 113.28 (C_{aryl}H), 111.29 (Cq_{aryl}), 88.76 (*C*q), 76.01(*C*H), 40.03 (CH_2), 36.86 (CH_2), 17.10 (CH_2), 16.48 (CH_2), 14.23 (CH_3),14.17 (CH_3).

HRMS (FD^+) *m/z* calcd for $C_{62}H_{62}N_4O_2$ [$M^{\cdot+}$] 894.4873, found 894.4899.

***^{n}Bu/^{n}Bu** ligand (R=^{n}Bu, **L5**)*

(*R*)-1,1'-binaphthyl-2,2'-diamine (0.072 g, 2.48 mmol) (*S*)-oxazoline **I5** (0.308 g, 7.43 mmol), Xantphos Pd G4 (0.052 g, 0.05 mmol) and sodium t-butoxide (0.070 g, 7.43 mmol) were used.

Column: Flash Silica, 12 g. Running scheme: t(min) 0: EtAc 0 %, 8: EtAc 0 %, 0: EtAc 2%, 14: EtAc 2 %, 0: EtAc 3%, 10: EtAc 3 %. Yield: white solid 0,085 g, 35 %.

^{1}H NMR (CD_2Cl_2) δ 10.22 (s, 2H, N*H*), 7.69-6.67 (m, 30H, Ar*H*), 4.35 (s, 2H, C*H*), 1.67 – 1.53 (m, 4H, CH_2), 1.43 – 1.31 (m, 4H, CH_2), 1.44 – 0.88 (m, 16H, CH_2), 0.98 (t, *J* = 7.1 Hz, 6H, CH_3), 0.71 (t, *J* = 6.6 Hz, 6H, CH_3).

^{13}C NMR (CD_2Cl_2) δ 162.94 (N*C*O), 144.76 (Cq_{aryl}), 138.78 (Cq_{aryl}), 138.56 (Cq_{aryl}), 134.01 (Cq_{aryl}), 131.25 (C_{aryl}H), 130.01 (Cq_{aryl}), 129.49 (C_{aryl}H), 128.28 (C_{aryl}H), 128.10 (C_{aryl}H), 127.73 (C_{aryl}H), 127.53 (C_{aryl}H), 126.70 (Cq_{aryl}), 125.90 (C_{aryl}H), 124.45 (C_{aryl}H), 123.31 (C_{aryl}H), 122.11 (Cq_{aryl}), 119.98 (C_{aryl}H), 116.79 (C_{aryl}H), 113.27 (C_{aryl}H), 111.33 (Cq_{aryl}), 88.82 (*C*q), 76.04 (*C*H), 37.43 (CH_2), 34.21 (CH_2), 25.87 (CH_2), 25.27 (CH_2), 23.05 (CH_2), 22.92 (CH_2), 13.82 (CH_3), 13.55 (CH_3).

HRMS (FD^+) *m/z* calcd for $C_{66}H_{70}N_4O_2$ [$M^{\cdot+}$] 950.5499, found 950.5588.

***^{n}Hex/^{n}Hex** ligand (R=^{n}Hex, **L6**)*

Due to the low yield obtained by following the above general procedure, the *gem*-nHex,nHex **L6** ligand synthesis was performed in two steps as follows:

First step:

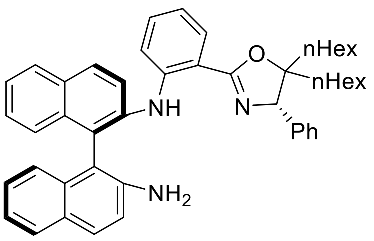

(R)-N2-(2-((S)-5,5-dihexyl-4-phenyl-4,5-dihydrooxazol-2-yl)phenyl)-[1,1'-binaphthalene]-2,2'-diamine. Under inert conditions, (*R*)-1,1’-binaphthyl-2,2’-diamine (201 mg, 0.70 mmol, 1.0 eq), XantPhos Pd G4 (135 mg, 0.14 mmol, 0.2 eq), sodium *tert*-butoxide (102 mg, 1.0 mmol, 1.5 eq) and dry toluene (20 ml) were added to a dry Schlenk flask.

(S)-2-(2-bromophenyl)-5,5-dihexyl-4-phenyl-4,5-dihydrooxazole **I6** (0.50 g, mmol, 1.5 eq) was added and the mixture was stirred at 110 °C for 16 hours. Toluene was removed under reduced pressure and the product was purified by column chromatography on an auto-column with pentane/ethyl acetate as eluent to afford the product as an orange oil (321 mg, 68%).

Column: Flash Silica, 25 g. Running scheme t(min) 0: EtAc 0%, 10: EtAc 2%, 30 EtAc 3%. Product collection in t30-40.

Second step:

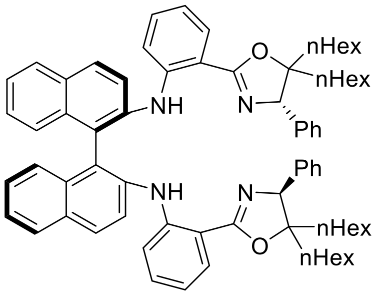

(R)-N2,N2'-bis(2-((S)-5,5-dihexyl-4-phenyl-4,5-dihydrooxazol-2-yl)phenyl)-[1,1'-binaphthalene]-2,2'-diamine. Under inert conditions, (*R*)-*N*2-(2-((*S*)-5,5-dihexyl-4-phenyl-4,5-dihydrooxazol-2-yl)phenyl)-[1,1'-binaphthalene]-2,2'-diamine (321 mg, 0.476 mmol, 1.0 eq), XantPhos Pd G4 (110 mg, 0.114 mmol, 0.24 eq), sodium *tert*-butoxide (85 mg, 0.88 mmol, 1.9 eq) and dry toluene (20 ml) were added to a dry Schlenk flask.

(*S*)-2-(2-bromophenyl)-5,5-dihexyl-4-phenyl-4,5-dihydrooxazole **I6** (0.400 g, 0.850 mmol, 1.78 eq) was added and the mixture was stirred at 110 °C for 16 hours. Toluene was removed under reduced pressure and the product was purified by column chromatography on an auto-column with pentane/ethyl acetate as eluent to afford the product as an orange oil (165 mg, 68%).

Column: Flash Silica, 25 g. Running scheme t(min) 0: EtAc 0%, 10: EtAc 2%, t30 EtAc 3%. Product collection in t10-30.

^{1}H NMR (CD_2Cl_2) δ 10.21 (s, 2H, N*H*), 7.68 – 7.41 (m, 8H, Ar*H*), 7.31 – 6.85 (m, 20H, ArH), 6.67 (t, *J* = 7.5 Hz, 2H, Ar*H*), 4.33 (s, 2H, C*H*), 1.65 – 0.99 (m, 40H, CH_2), 0.95 (d, *J* = 6.9 Hz, 6H, CH_3), 0.81 (t, *J* = 7.1 Hz, 6H, CH_3).

^{13}C NMR (CD_2Cl_2) δ 162.92 (N*C*O), 144.75 (*C*q_{aryl}), 138.80 (*C*q_{aryl}), 138.57(*C*q_{aryl}), 134.01 (*C*q_{aryl}), 131.22 (C_{aryl}H), 130.01 (*C*q_{aryl}), 129.48 (C_{aryl}H), 128.26 (C_{aryl}H), 128.10 (C_{aryl}H), 127.71 (C_{aryl}H), 127.53 (C_{aryl}H), 126.70 (*C*q_{aryl}), 125.89 (C_{aryl}H), 124.45 (C_{aryl}H), 123.30 (C_{aryl}H), 122.11 (*C*q_{aryl}), 119.99 (C_{aryl}H), 116.77 (C_{aryl}H), 113.27 (C_{aryl}H), 111.35 (*C*q_{aryl}), 88.88 (*C*q), 76.03 (*C*H), 37.71 (*C*H_2), 34.52 (*C*H_2), 31.71 (*C*H_2), 31.52 (*C*H_2), 29.61 (*C*H_2), 29.54 (*C*H_2), 23.65 (*C*H_2), 23.06 (*C*H_2), 22.66 (*C*H_2), 22.42 (*C*H_2), 13.86 (*C*H_3), 13.72 (*C*H_3).

HRMS (FD^+) *m/z* calcd for $C_{74}H_{86}N_4O_2$ [$M^{\bullet+}$] 1062.6751, found 1062.6721.

General procedure for the synthesis of the Fe-Complexes

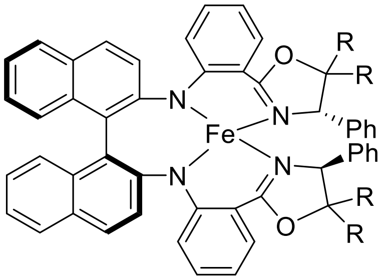

1.1 molar equiv. $Fe(HMDS)_2$ and 1 molar equiv. of the respective ligand **L1-L6** were added to a scintillation vial in an inert atmosphere glovebox. Anhydrous and degassed toluene (5 mL) was added, and the mixture stirred overnight (16 h) at room temperature. The color of the reaction mixture turned blood red. All volatile materials were removed under vacuum, and the resulting solid washed with cold pentane (2 or 3 × 2 mL) to remove unreacted $Fe(HMDS)_2$. The purified complex was dried under vacuum and stored in an airtight container under an inert atmosphere at low temperature. All complexes were obtained as red powder.

***Fe-H/H** complex, (R,S,S)-**1**:*

Ligand (*R,S,S*)-**L1** (64 mg, 0.088 mmol) and $Fe(HMDS)_2$ (36.4 mg, 0.097 mmol) afforded complex *(R,S,S)*-**1** (55.0 mg, 80% yield).

^{1}H NMR (C_6D_6) δ 78.27, 30.04, 26.11, 16.94,15.24, 9.99, 2.12, 0.90, 0.29, -7.25, -24.14, -25.24, -28.82, -31.29, -58.38.

μ_{eff} (C_6D_6, 25 °C) 4.97 μ_B.

HRMS (LIFDI$^+$) *m/z* calcd for $C_{50}H_{36}FeN_4O_2$ [$M^{\bullet+}$] 780.2189, found 780.2174.

***Fe-Me/Me** complex, (R,S,S)-**2**:*

Ligand (*R,S,S*)-**L2** (200 mg, 0.26 mmol) and $Fe(HMDS)_2$ (105.7 mg, 0.28 mmol) afforded complex *(R,S,S)*-**2** (162.4 mg, 75% yield).

^{1}H NMR (C_6D_6) δ 78.94, 26.21, 16.85,15.91, 10.27, 5.79, 4.82 0.90, 1.22, 0.94, 0.86, 0.28, 0.09, -2.26, -25.41, -27.40, -31.42, -35.37, -64.24.

μ_{eff} (C_6D_6, 25 °C) 5.02 μ_B.

HRMS (LIFDI$^+$) *m/z* calcd for $C_{54}H_{44}FeN_4O_2$ [$M^{\bullet+}$] 836.2815, found 836.2854.

***Fe-Me/Me** complex, (R,R,R)-**2**:*

Ligand (*R,R,R*)-**L2** (250 mg, 0.32 mmol) and $Fe(HMDS)_2$ (132.1 mg, 0.35 mmol) afforded *(R,R,R)*- **2** (197.7 mg, 74% yield).

^{1}H NMR (C_6D_6) δ 74.83, 71.60, 19.68, 15.94, 12.44, 11.72, 3.60, 1.64, 1.35, 0.87, 0.09, -7.86, -17.91, -21.23, -22.74, -24.88, -52.42.

μ_{eff} (C_6D_6, 25 °C) 4.95 μ_B.

***Fe-Et/Et** complex, (R,S,S)-**3**:*

Ligand ((*R,S,S*)-**L3** (150 mg, 0.18 mmol) and Fe(HMDS)$_2$ (74.0 mg, 0.20 mmol) afforded (*R,S,S*)-**3** (102.1 mg, 64% yield).

^{1}H NMR (C_6D_6) δ 77.20, 25.18, 16.59, 15.56, 5.42, -1.76, -2.38, -25.39, -27.38, -31.40, -35.42, -64.51.

μ_{eff} (C_6D_6, 25 °C) 5.14 μ_B.

HRMS (LIFDI$^+$) *m/z* calcd for $C_{58}H_{52}FeN_4O_2$ [$M^{\bullet +}$] 892.3441, found 892.3496.

***Fe-^{n}Pr/^{n}Pr** complex, (R,S,S)-**4**:*

Ligand ((*R,S,S*)-**L4** (177 mg, 0.20 mmol) and Fe(HMDS)$_2$ (81.8 mg, 0.22 mmol) afforded (*R,S,S*)-**4** (116.3 mg, 62% yield).

^{1}H NMR (C_6D_6) δ 77.05, 23.84, 16.21, 14.99, 10.83, 5.05, 2.52, -0.47, -2.02, -23.80, -25.82, -29.82, -34.12, -65.65.

μ_{eff} (C_6D_6, 25 °C) 5.10 μ_B.

HRMS (LIFDI$^+$) *m/z* calcd for $C_{62}H_{60}FeN_4O_2$ [$M^{\bullet +}$] 948.4067, found 948.4086.

Fe-^{n}Bu/^{n}Bu** (R,S,S)-**5:

Ligand ((*R,S,S*)-**L5** (84.6 mg, 0.089 mmol) and Fe(HMDS)$_2$ (36.8 mg, 0.098 mmol) afforded (*R,S,S*)-**5** (50.1 mg, 56% yield).

^{1}H NMR (C_6D_6) δ 76.97, 23.69, 16.18, 14.94, 5.02, -6.93, -23.62, -26.05, -29.51,-33.90, -65.63.

μ_{eff} (C_6D_6, 25 °C) 4.96 μ_B.

HRMS (LIFDI$^+$) *m/z* calcd for $C_{66}H_{68}FeN_4O_2$ [$M^{\bullet +}$] 1004.4694, found 1004.4692.

Fe-^{n}Hex/^{n}Hex** (R,S,S)-**6:

Ligand ((*R,S,S*)-**L6** (165 mg, 0.16 mmol) and Fe(HMDS)$_2$ (67 mg, 0.18 mmol) afforded (*R,S,S*)-**6** (167.9 mg, 97% yield). In this specific case, no washing steps were performed, due to the high solubility of the targeted complex in pentane.

^{1}H NMR (C_6D_6) δ 77.32, 24.22, 16.33, 15.18, 10.66, 5.03, 4.47, -1.47, -2.31, -2.92, -24.05, -26.09, -30.13, -34.31, -65.75.

μ_{eff} (C_6D_6, 25 °C) 5.10 μ_B.

HRMS (LIFDI$^+$) *m/z* calcd for $C_{74}H_{84}FeN_4O_2$ [$M^{\bullet +}$] 1116.5946, found 1116.5999.

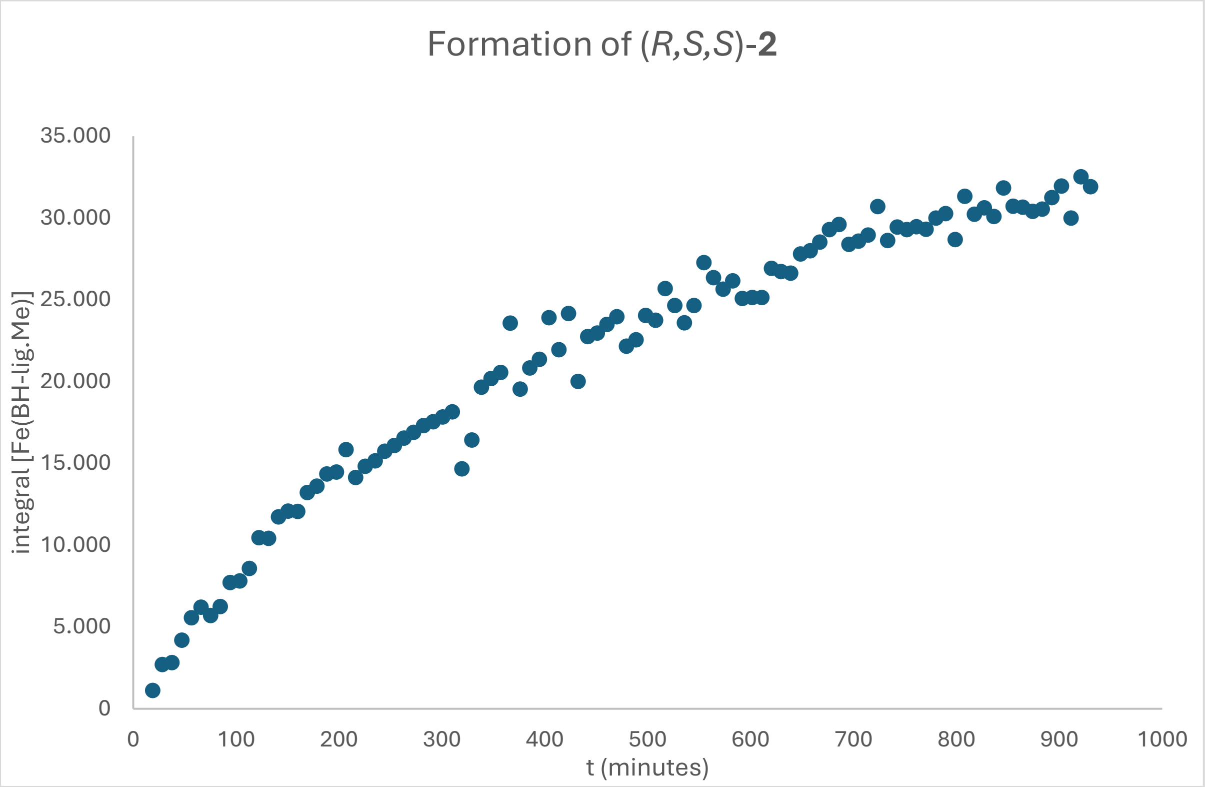

Figure S2. Complex formation over time.

Proposed dehydrogenation of oxazoline in complex 1 upon reaction with aryl azide

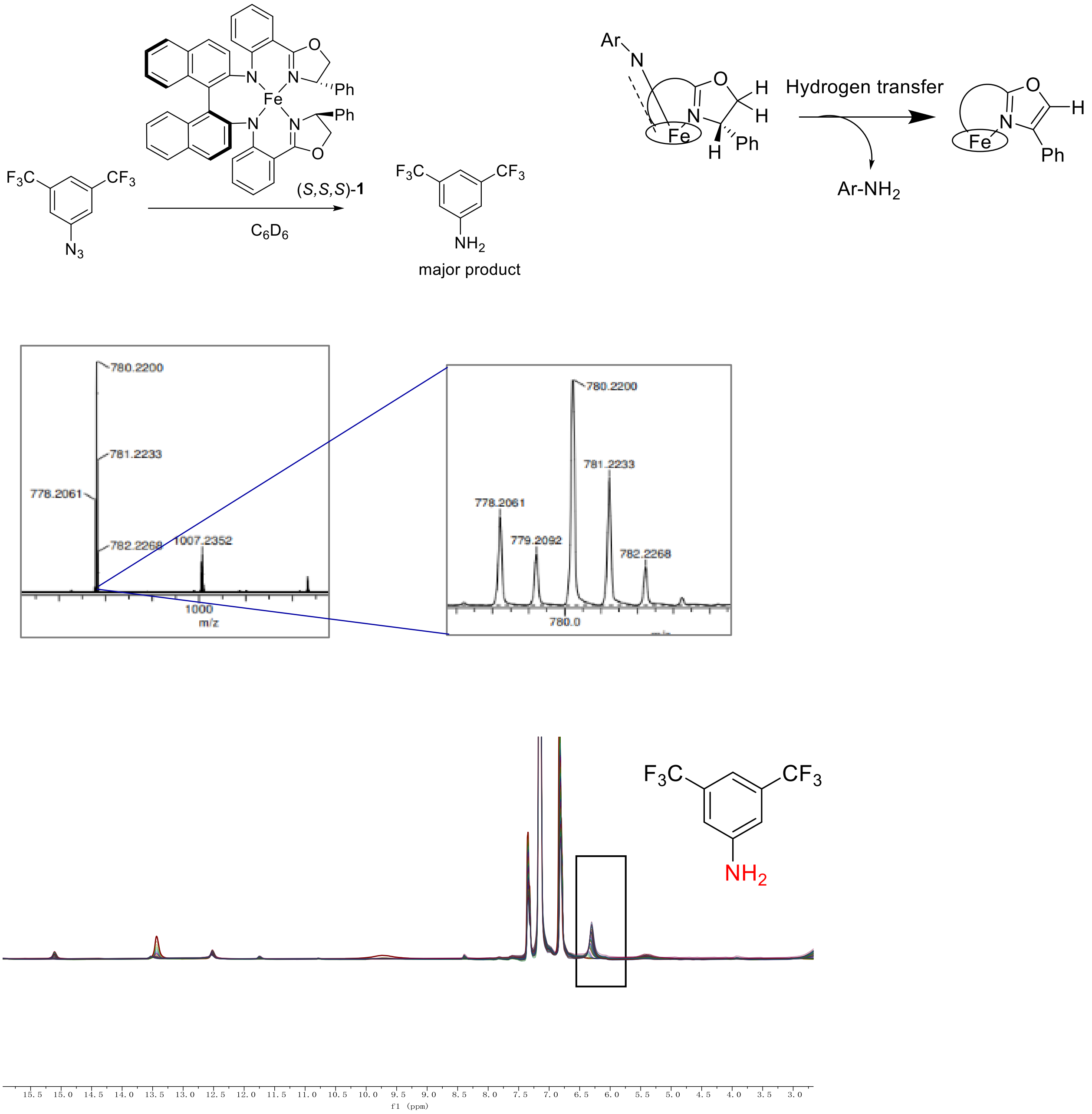

Figure S3. Dehydrogenation of oxazoline fragment in **1** upon reaction with 3,5-bis(trifluorometyhl)phenyl azide to form the oxazole-derivative and 3,5-bis(trifluormethyl) aniline as observed by HR-MS and ^{1}H NMR, respectively

Indications for Fe-NR Species

Mass Spectrometry

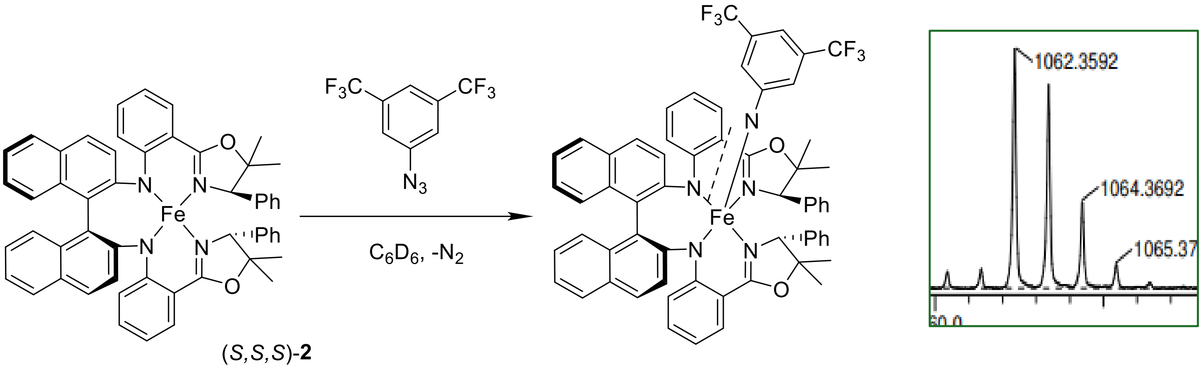

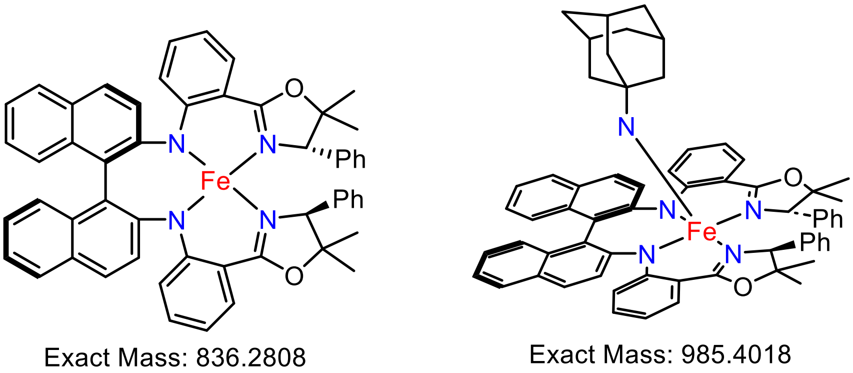

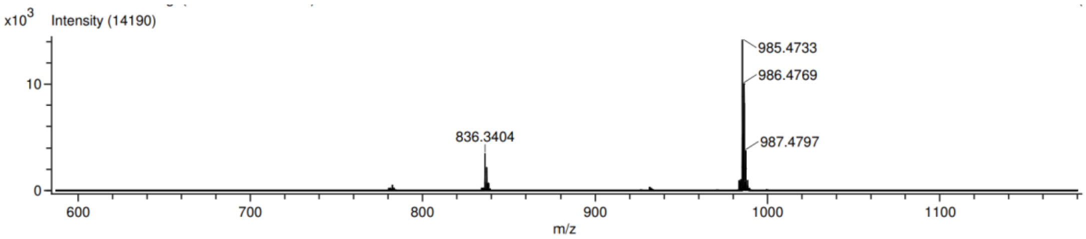

Figure S4. Mass spectrometric data for the reaction of (*S*,*S*,*S*)-**2** with 3,5-bis(trifluoromethyl)phenyl azide (top) and (*R*,*S*,*S*)-**2** with adamantyl azide (bottom)

Mössbauer spectroscopy

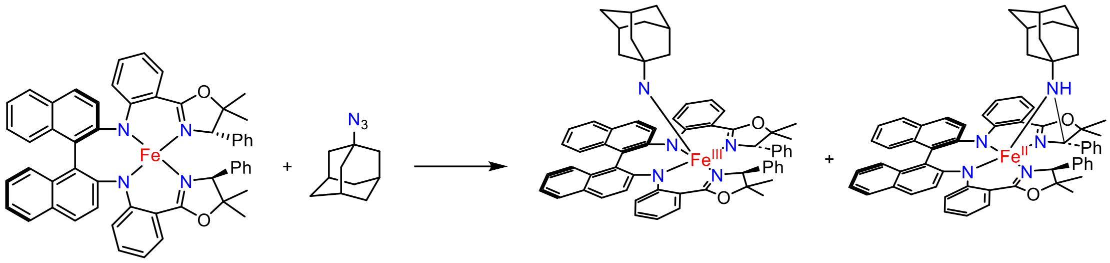

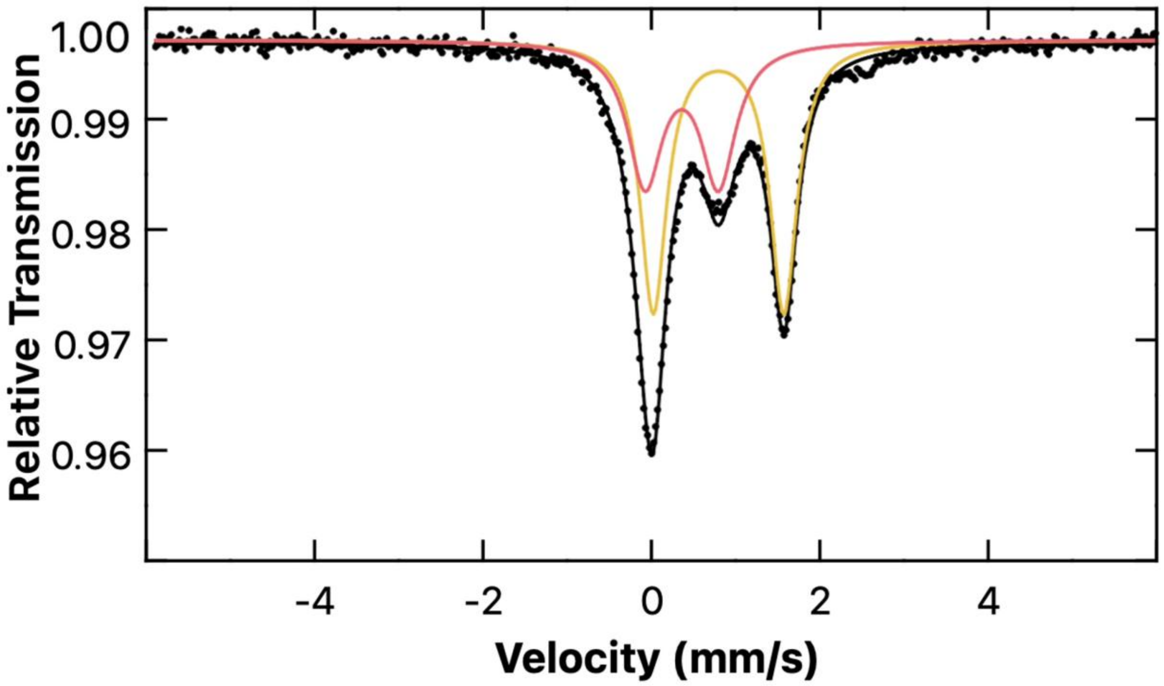

Figure S5. Zero-field 80 K ^{57}Fe Mössbauer spectrum after reaction of (*R*,*S*,*S*)-**2** with adamantyl azide (at 80 °C) leading to a signal interpreted as a high-spin Fe(III)-nitrene radical (red trace; δ = 0.46 mm s^{-1}, $|\Delta E_Q|$ = 0.88 mm s^{-1}, 33%), concurrent with a signal for a follow-up intramolecular rearrangement process involving the ligand backbone to give an Fe(III) aminyl radical) (yellow trace; δ = 0.89 mm s^{-1}, $|\Delta E_Q|$ = 1.41 mm s^{-1}, 67%). Black dotted trace: raw data. Solid trace: simulation.

EPR Spectroscopy

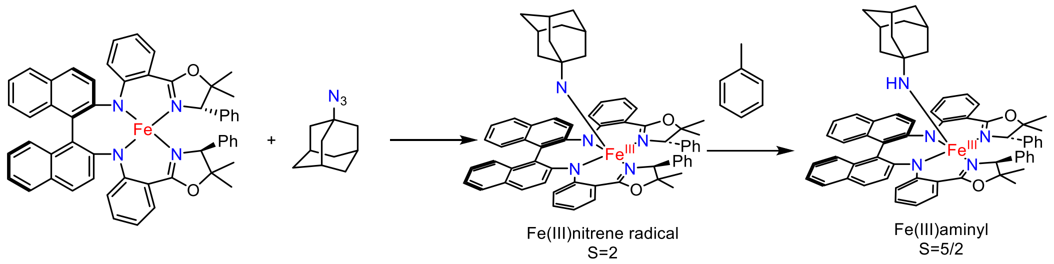

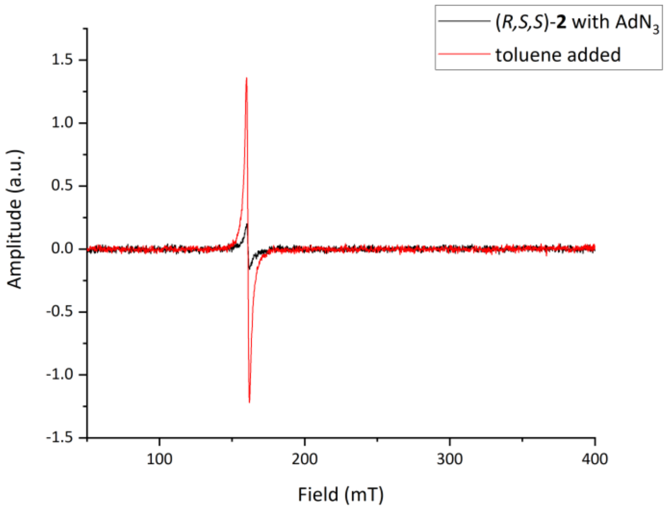

Figure S6. EPR spectrum for the same reaction of (*R*,*S*,*S*)-**2** and adamantyl azide and in the absence and presence of toluene (as H-atom donor). The black trace is attributed to the trace impurity in the otherwise EPR-silent high-spin Fe(III)-nitrene radical (S = 2) species. This trace strikingly intensifies upon addition of toluene (red trace) – we assign this to the corresponding Fe(III) aminyl (S = 5/2) derivative formed upon H-atom transfer from toluene.

In Situ Monitoring (NMR, UV-vis, IR-dip-probe) of Resting State

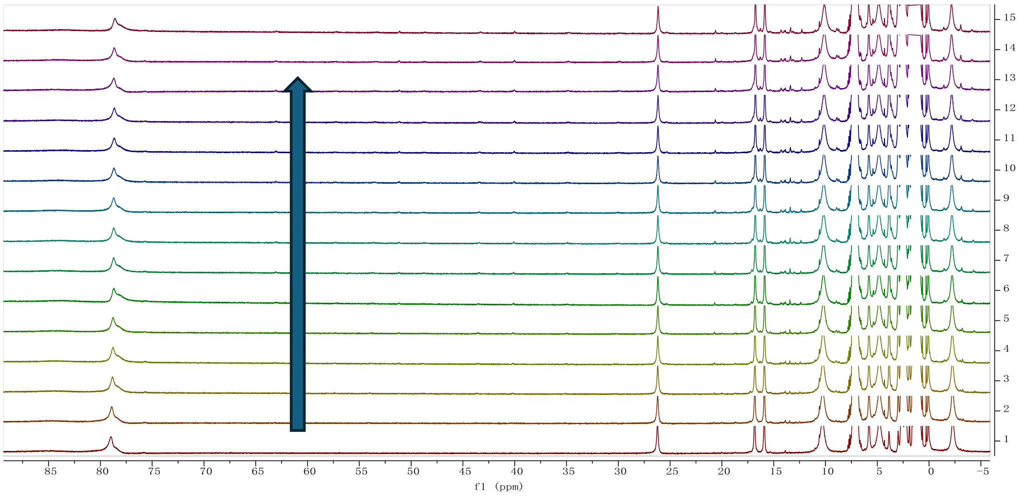

Figure S7. In situ ^{1}H NMR spectra for mixture of (*R*,*S*,*S*)-**2** and benchmark azide

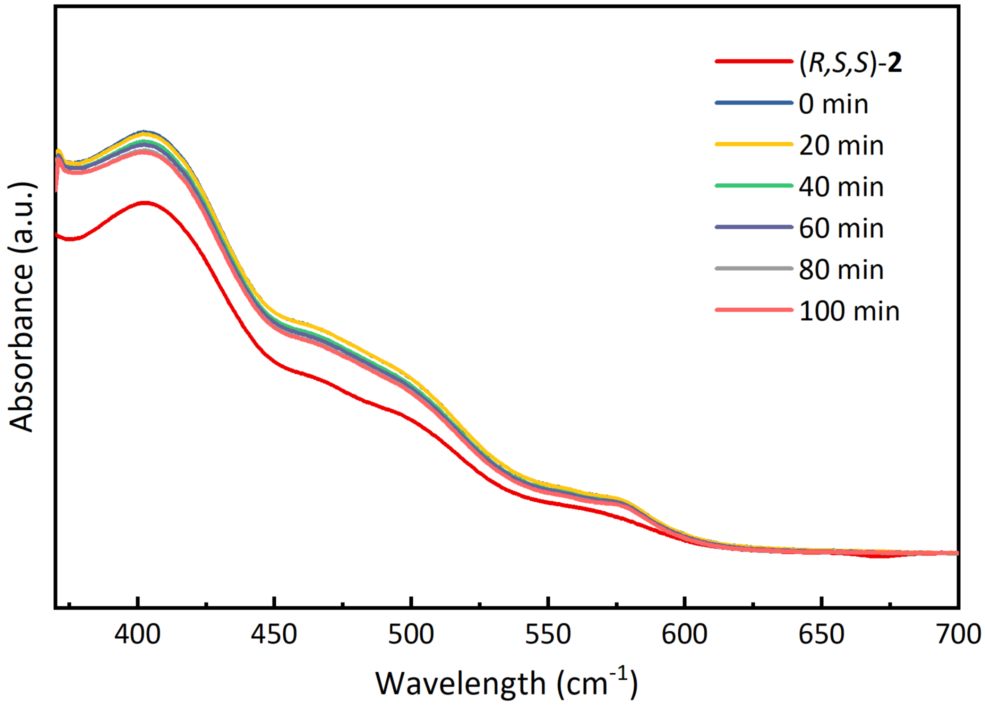

Figure S8. In situ UV-vis spectra for mixture of (*R*,*S*,*S*)-**2** and benchmark azide, with 5mM catalyst solution and 5 μmol azide added, with the reaction mixture then diluted to 50μM

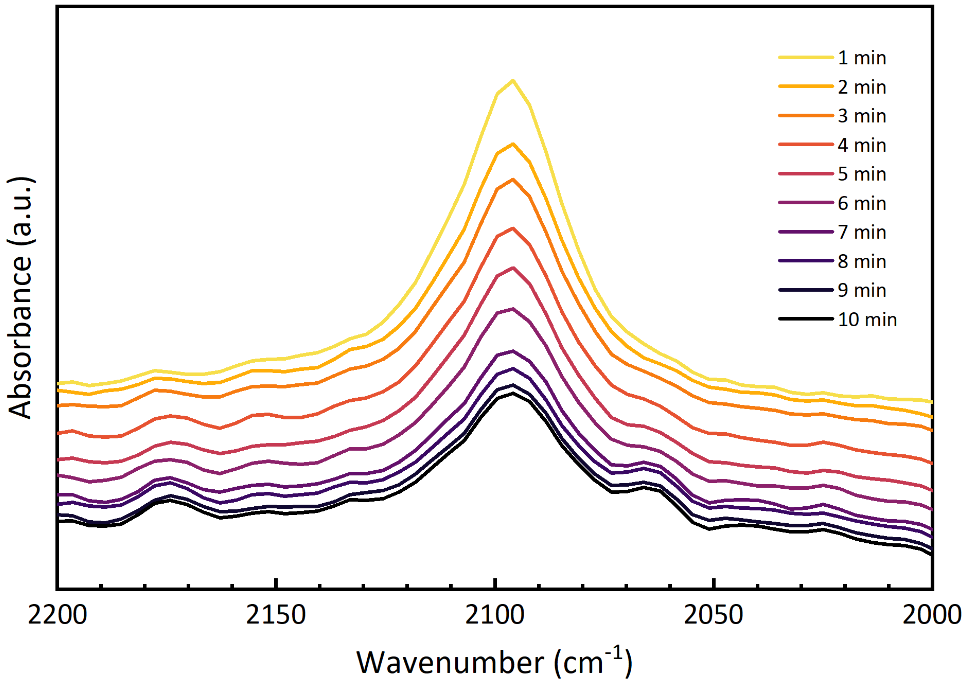

Figure S9. In situ dip-probe IR spectra for mixture of (*R*,*S*,*S*)-**2** and benchmark azide

Kinetic study

Determination of the reaction order - substrate:

Catalytic reaction using complex (*R*,*S*,*S*)-**2** and 4-phenylbutyl azide was performed *in duplo* with 0.5 mol%, 1 mol%, 1.5 mol%, 2.5 mol% and 5 mol% catalyst loading. The ^{1}H NMR spectrum was recorded every 5 minutes for at minimum of 40 minutes and the amount of product and substrate with respect to trimethoxybenzene (internal standard) was determined.

Plotting the substrate concentration $[A]_t$ as $\ln[A]_t - \ln[A]_o$ versus time revealed a linear regression line with R^2>0.99. This indicates a first order dependence for the reaction rate with regard to the substrate concentration [A].

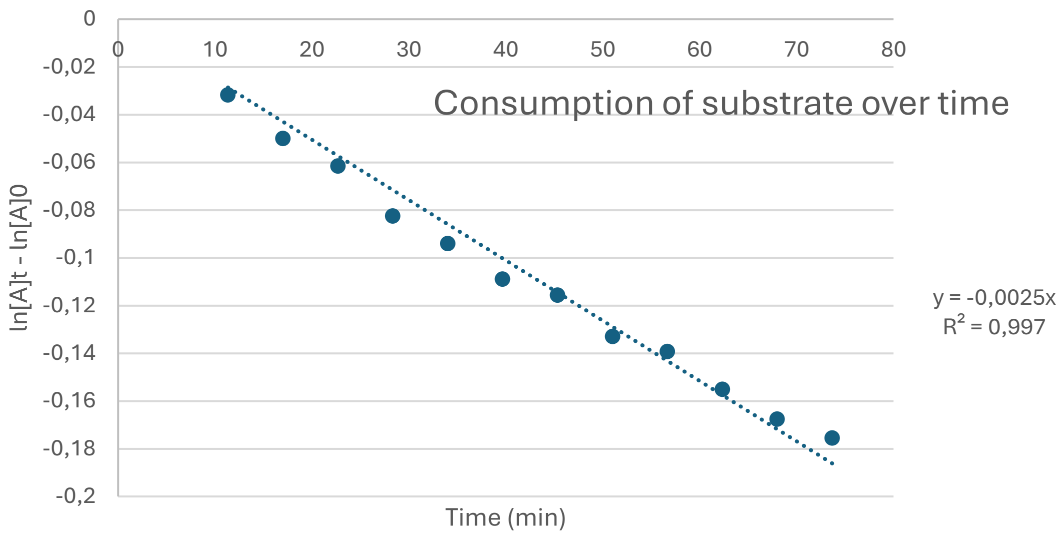

Figure S10. Correlation between substrate consumption (plotted as ln[A]t - ln[A]0) and time.

For subsequent calculations, the following rate law was utilized, assuming first order dependence on catalyst concentration based on preliminary data:

rate = -d[A]/dt = k[A][catalyst] [S1]

During catalysis, catalyst concentration was assumed to remain constant, resulting in an observed pseudo-first-order rate law and observed rate coefficient as follows:

rate = k[A][catalyst] = k_{obs}[A] [S2]

The value of k_{obs} was determined for each measurement from the slope of the linearized plot of substrate concentration over time, as:

$d(\ln[A]_t/\ln[A]_0)/dt = -k_{obs}$ [S3]

From these values, the values of the rate coefficient for a given temperature could then be calculated:

k = k_{obs} [catalyst] [S4]

Table S2. Calculated and obtained values.

[catalyst] (M)	k_{obs} (s^{-1})	k_{obs} (s^{-1})	average k_{obs} (s^{-1})	k_{obs} (h^{-1})
0	0	0	0	0
0.000375	0.000028	0.000029	0.0000285	0.1026
0.00075	0.000063	0.000061	0.000062	0.2232
0.001125	0.000099	0.000099	0.000099	0.3564
0.0015	0.000139	0.000138	0.0001385	0.4986
0.001875	0.000167	0.000173	0.00017	0.612

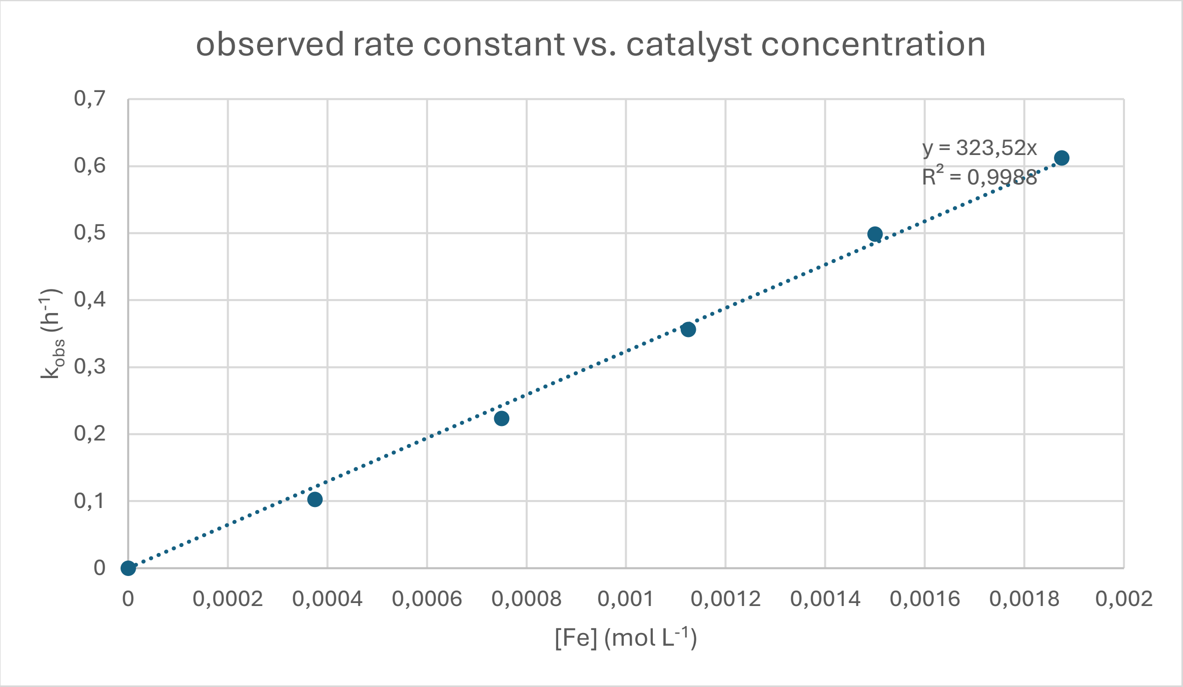

Figure S11. Correlation between the observed reaction rate k_{obs} and the catalyst concentration

VT-NMR Reaction Monitoring

Catalytic reaction using complex (*R*,*S*,*S*)-**2** and 4-phenylbutyl azide was performed *in duplo* at 45 °C, 40 °C, 35 °C, 30 °C and 25 °C . The ^{1}H NMR spectrum was recorded every 5 minutes (4 minutes for 45 °C) for at minimum of 40 minutes and the amount of product and substrate with respect to trimethoxybenzene (internal standard) was determined

Table S3. calculated values for rate coefficient *k* at different temperatures.

**Entry**	**T (K)**	**k_{obs} ×10^{-4} (h^{-1})**	**[catalyst] (mM)**	**k ($M^{-1}h^{-1}$)**	**1/T (K^{-1})**	**ln(k)**	**ln(k/T)**
1	303.15	0.65566	1.5	157.3584	0.003299	5.058526	-0.6557
2	303.15	0.63709	1.5	152.9016	0.003299	5.029795	-0.68443
3	308.15	0.7334	1.5	176.016	0.003245	5.170575	-0.56001
4	308.15	0.7854	1.5	188.496	0.003245	5.239077	-0.49151
5	313.15	0.95564	1.5	229.3536	0.003193	5.435265	-0.31142
6	313.15	0.81255	1.5	195.012	0.003193	5.273061	-0.47362
7	318.15	1.1036	1.5	264.864	0.003143	5.579216	-0.18331
8	318.15	0.98719	1.5	236.9256	0.003143	5.467746	-0.29478
9	298.15	0.45336	1.5	108.8064	0.003354	4.68957	-1.00803
10	298.15	0.42111	1.5	101.0664	0.003354	4.615778	-1.08182

Arrhenius Plot:

To obtain the activation energy of the reaction (E_{act}), the rearranged Arrhenius equation was used:

$\ln(k) = -E_{act}/R*(1/T) – \ln(A)$

Plotting ln(k) versus 1/T resulted in a linear plot with slope $-E_a/R$ (Figure S7).

From this, the energy of activation was quantified as E_{act}=7.78± 0.12 kcal mol^{-1}.

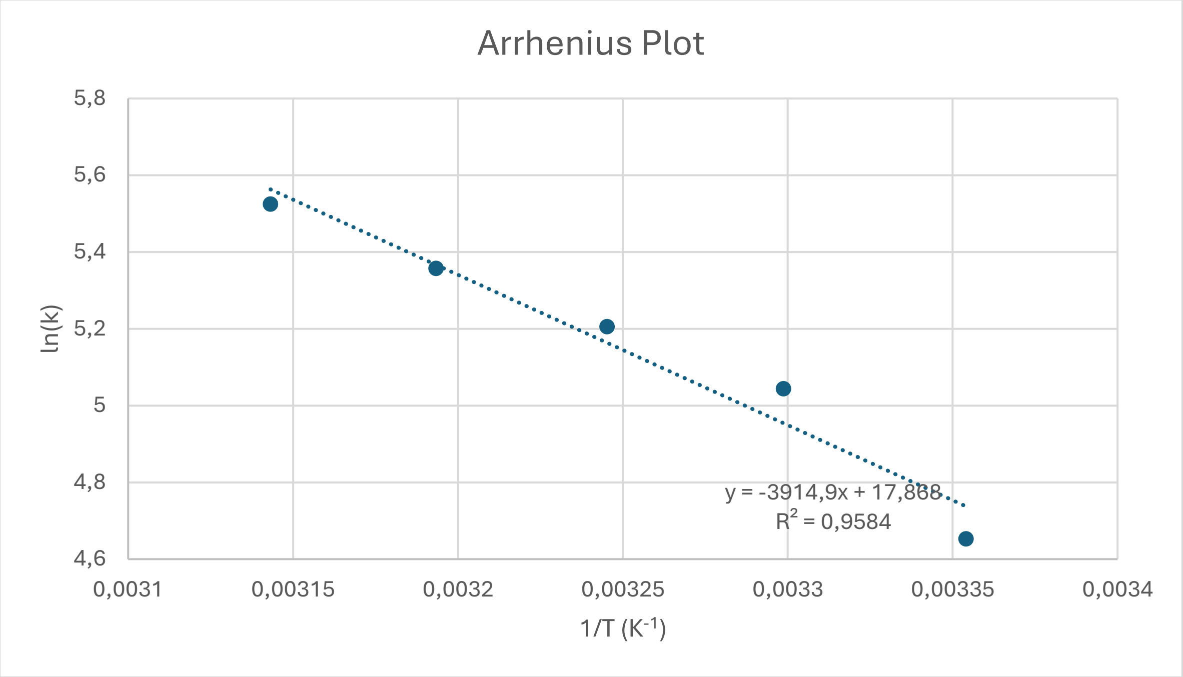

Figure S12. Arrhenius plot of catalysis with (*R*,*S*,*S*)-**2** between 25 ºC and 45 ºC

Eyring Plot:

To obtain the enthalpy of activation ($\Delta H^{\neq}$) and entropy of activation ($\Delta S^{\neq}$), the rearranged Eyring-Polyani equation was used:

$\ln(k/T) = -\Delta H^{\neq}/R * 1/T + \ln(\kappa k_B/T) + \Delta S^{\neq}/R$

Plotting ln(k/T) versus 1/T again yielded a linear plot with slope $-\Delta H^{\neq}/R$ and y-intercept $\ln(\kappa k_B/T) + \Delta S^{\neq}/R$ (Figure S8). To determine the entropy of activation, the transmission coefficient κ was assumed to be 1.

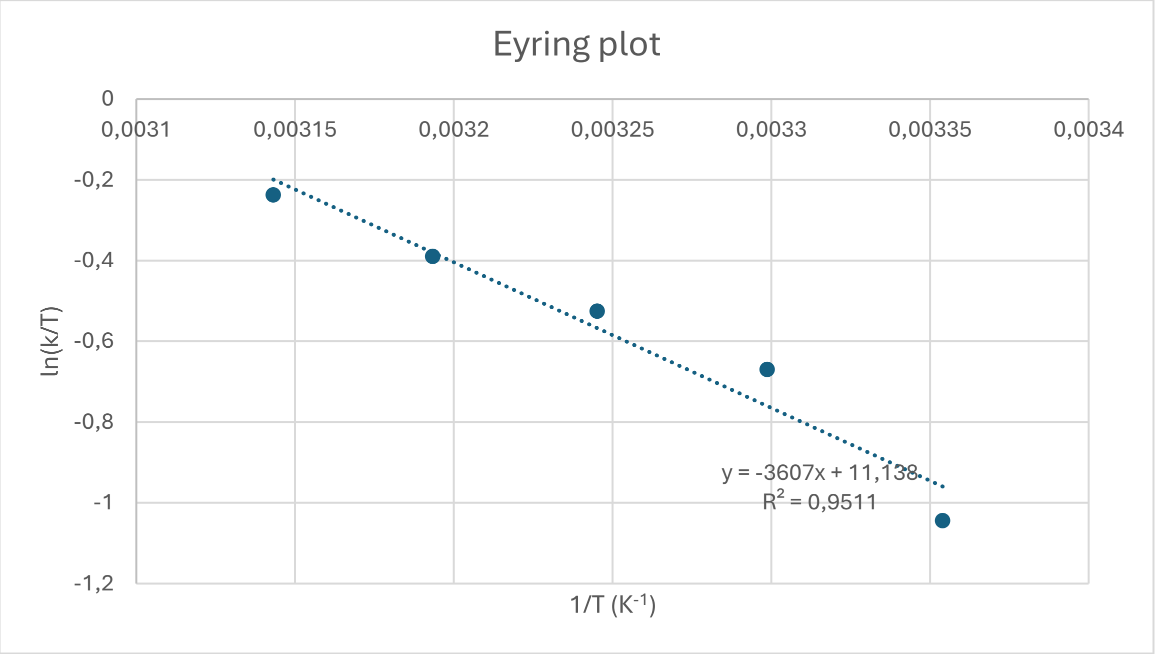

Figure S13. Eyring plot of catalysis using (*R,S,S*)-**2** at temperatures between 25 °C and 45 °C

The enthalpy and entropy of activation calculated from the slope and y-intercept of the Eyring plot were as follows: $\Delta H^{\neq}$=7.16±0.12 kcal mol^{-1}, $\Delta S^{\neq}$=-25.08±0.38 cal mol^{-1} K^{-1}.

Given that $\Delta G^{\neq}_{T} = \Delta H^{\neq} - T\Delta S^{\neq}$, for reaction at room temperature the free energy of activation was determined to be $\Delta G^{\neq}_{298K}$= 14.64±0.17 kcal mol^{-1}.

Effect of *gem*-alkyl,alkyl substition on catalysis:

General information for kinetics with different *gem*-substituted catalysts:

All catalytic reactions were done with 14 mM catalyst, 15 µL organic azide in 0.6 mL C_6D_6. Samples were collected every 5 minutes, and the results shown in stack with superimposed mode. (the reaction was performed at a higher catalyst concentration because of the low reactivity of catalyst **1**)

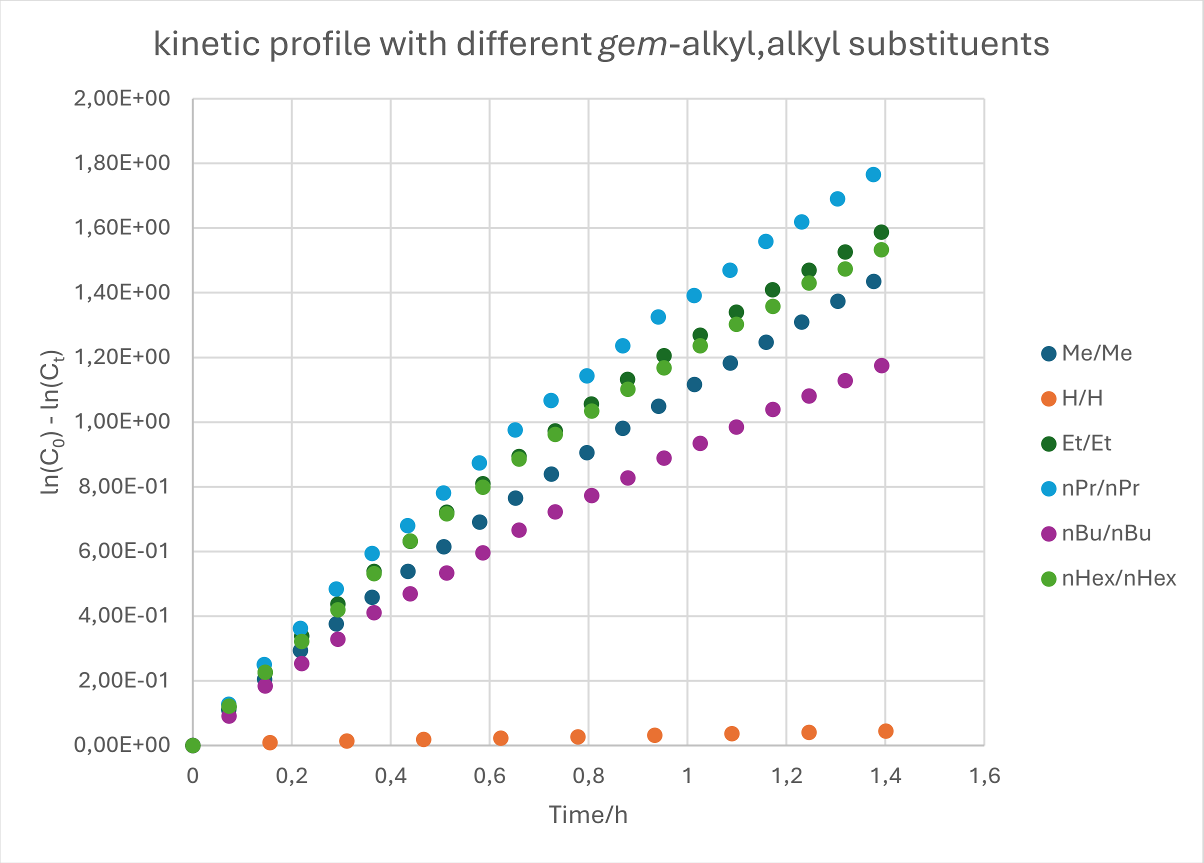

Figure S14. Kinetic profile with different *gem*-substitution catalyst

General information for incubation test:

All catalytic reactions were performed with 14 mM catalyst, 15 µL organic azide in 0.6 mL C_6D_6. Samples were taken every 5 minutes, and the results shown in stack with superimposed mode.

Py-azide reaction profile:

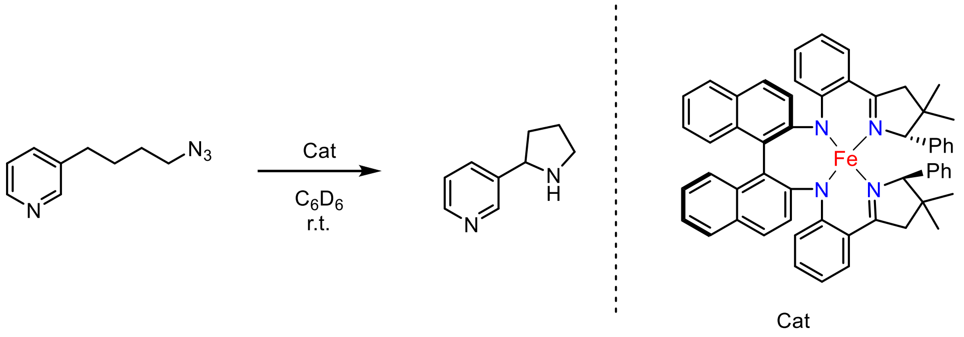

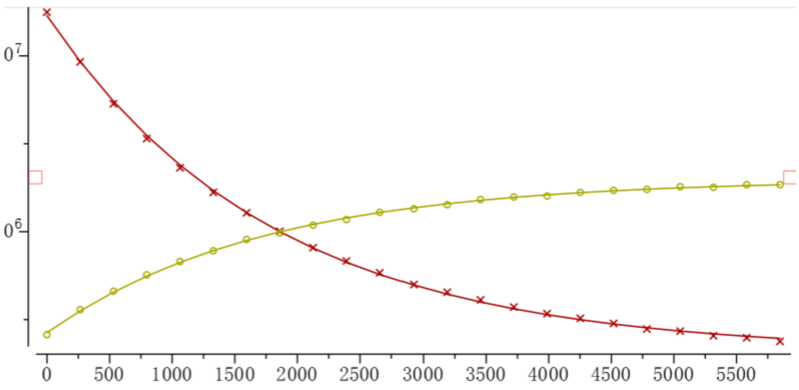

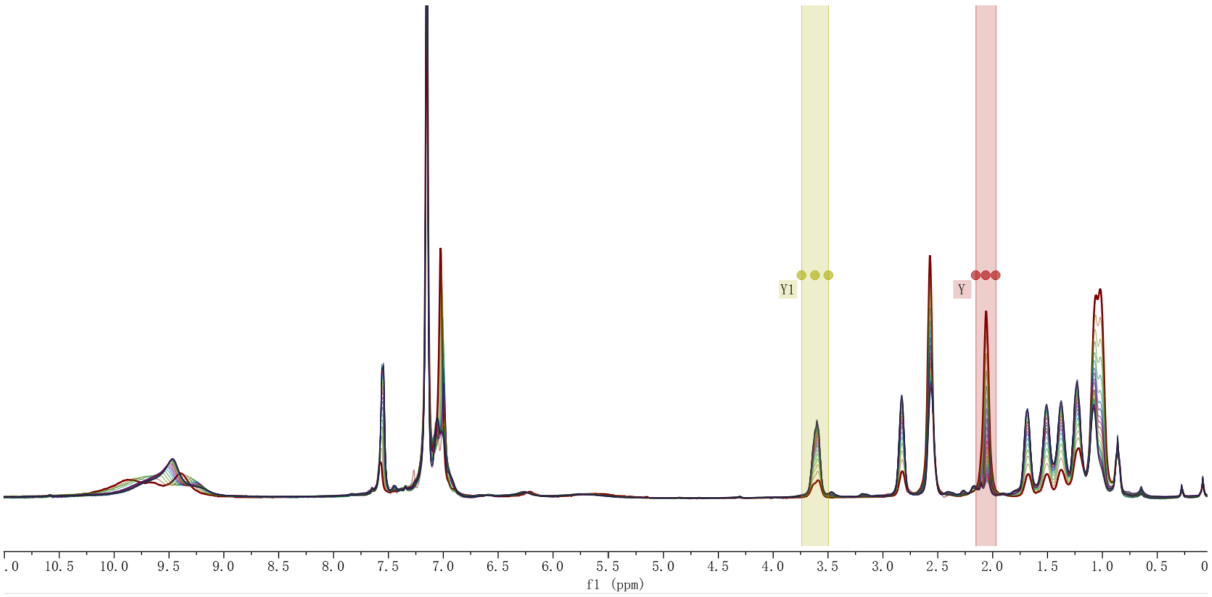

Figure S15. The reaction profile with Py-azide, the red line shows substrate conversion and yellow line shows product formation.

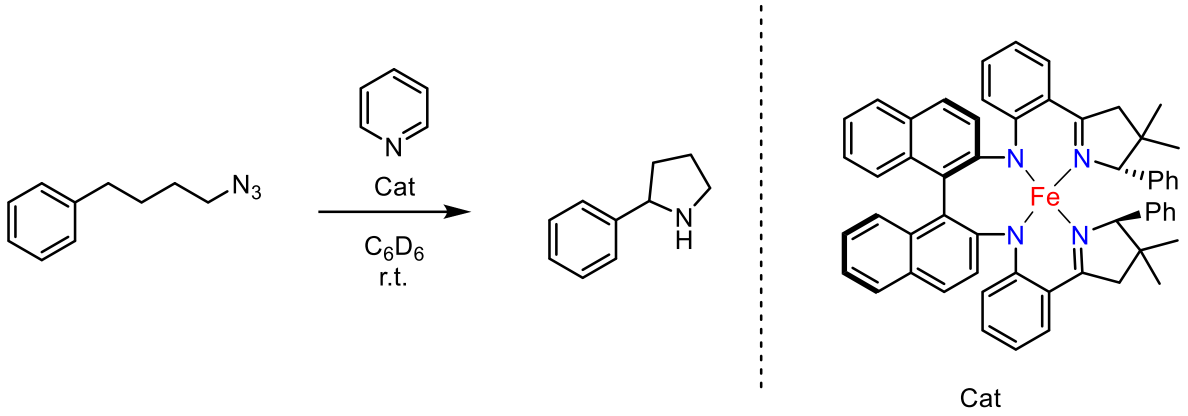

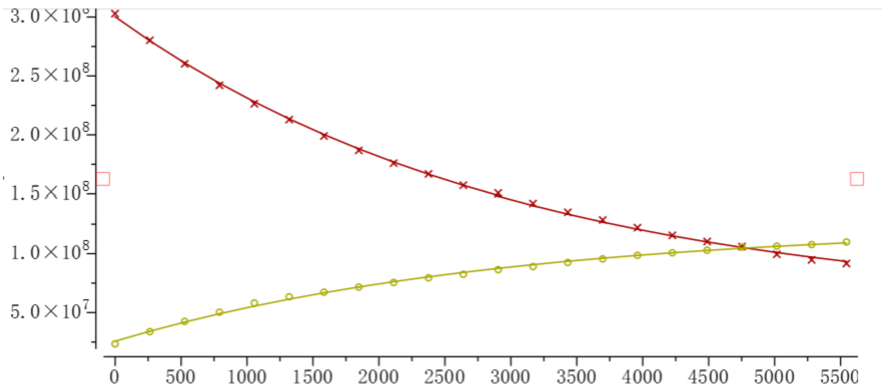

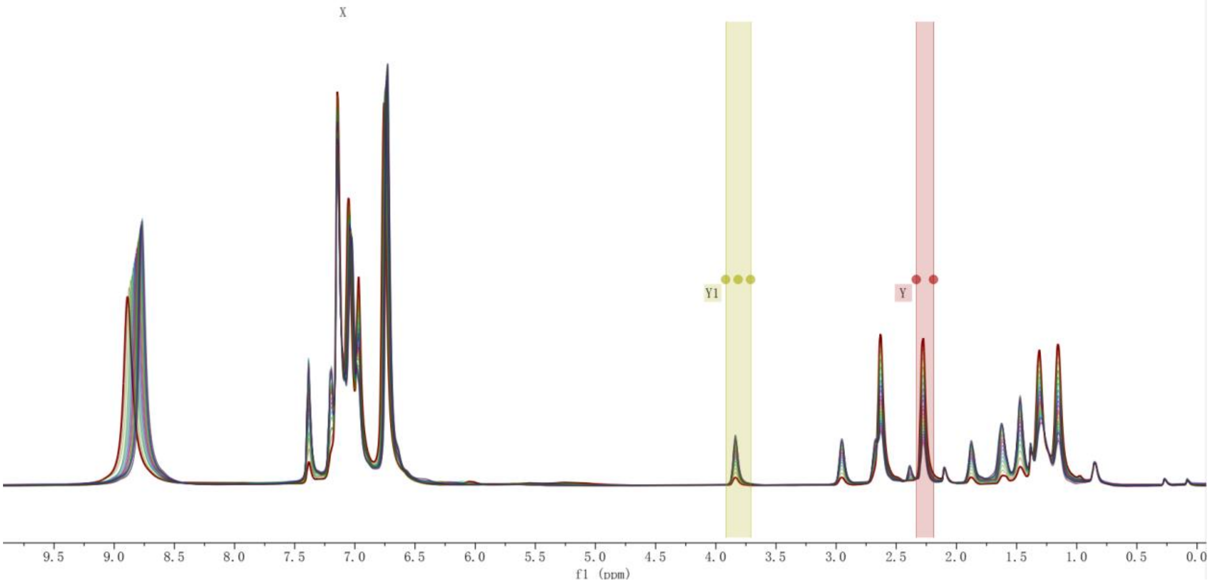

Figure S16. The reaction profile with 15 μL pyridine addition, the red line shows substrate conversion and yellow line shows product formation.

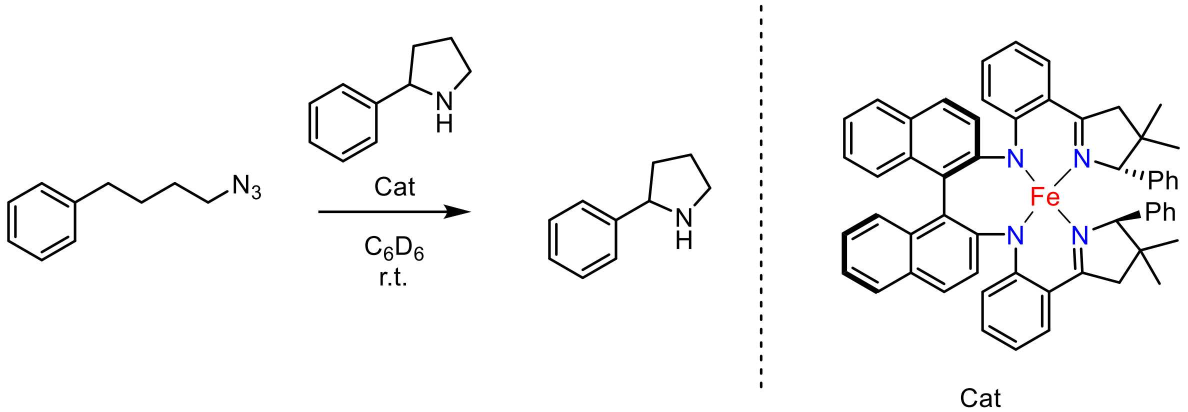

Cat

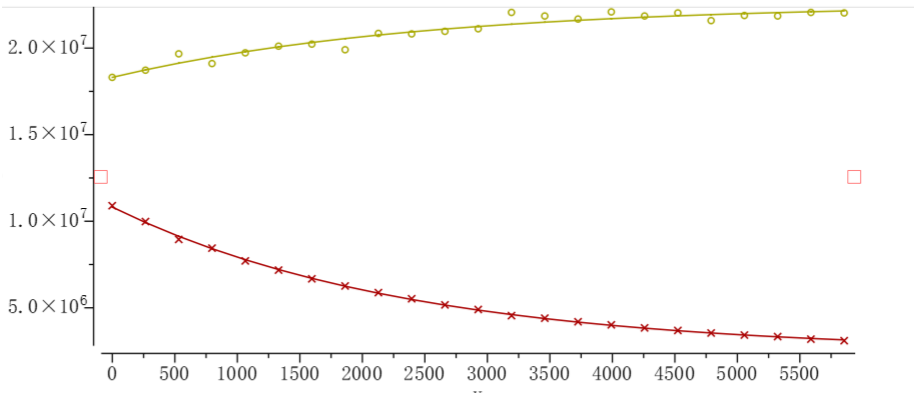

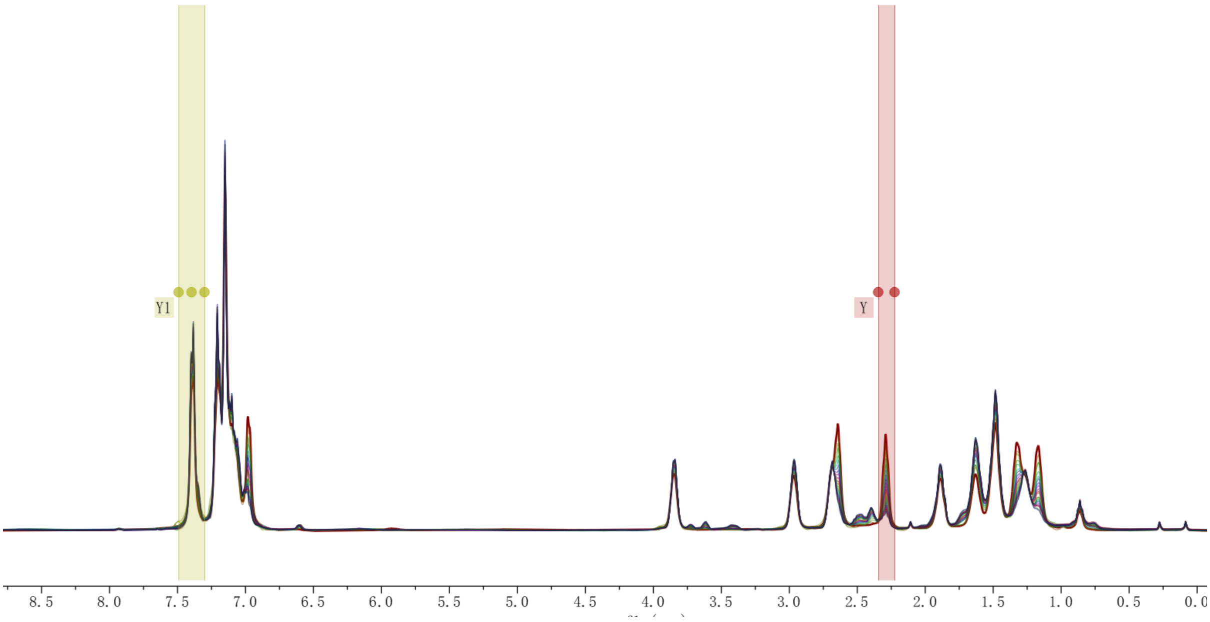

Figure S17. The reaction profile with 15 μL product addition, the red line shows substrate conversion and yellow line shows product formation.

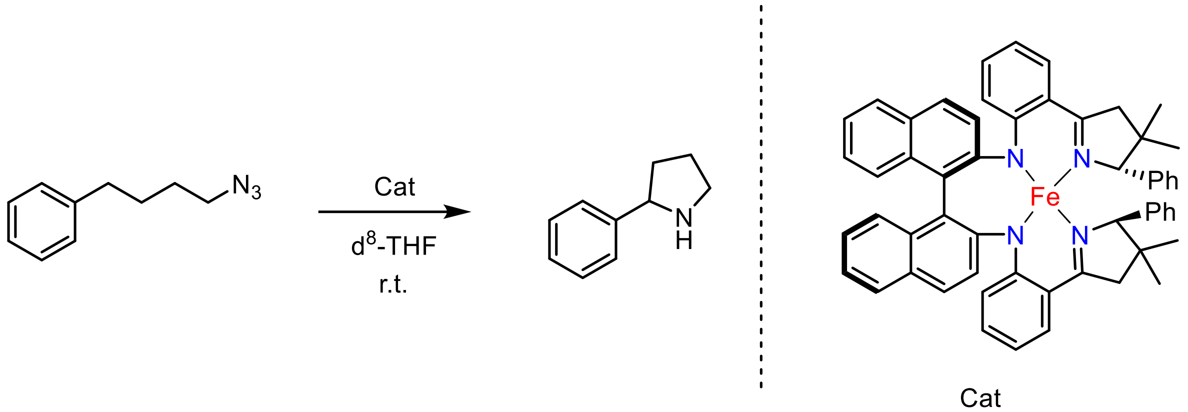

Cat

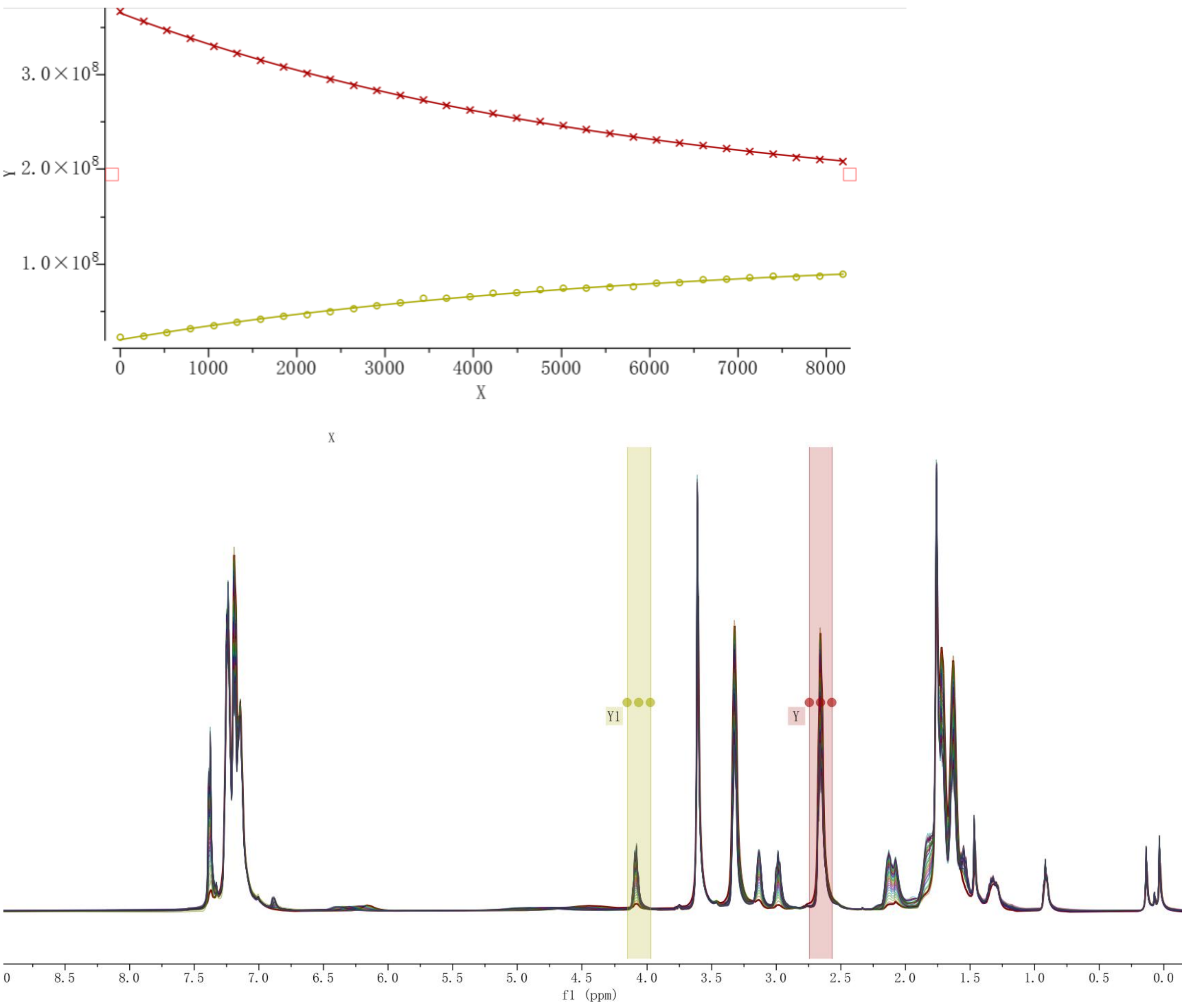

Figure S18. The reaction profile in THF-d^8, the red line shows substrate conversion and yellow line shows product formation.

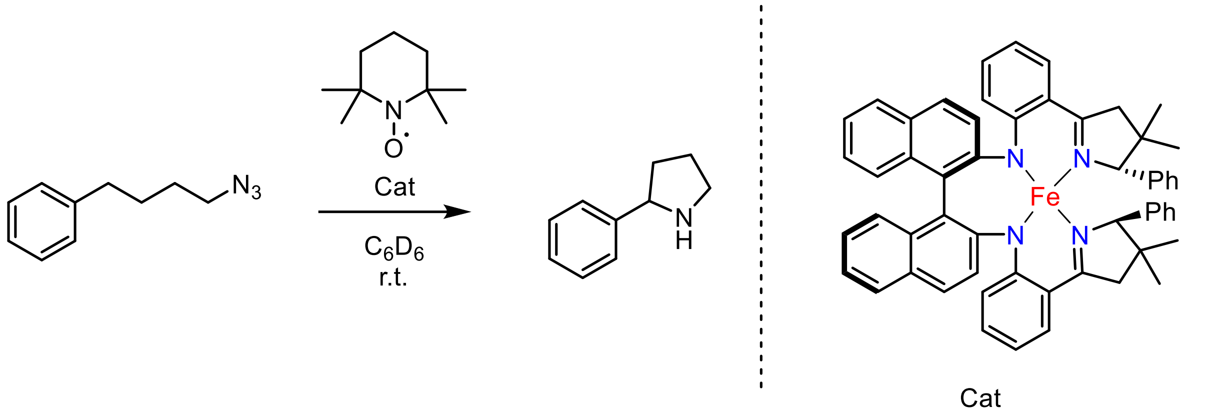

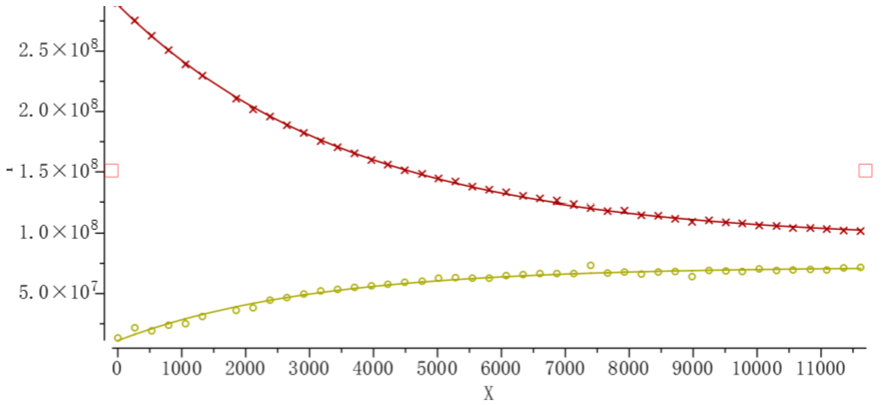

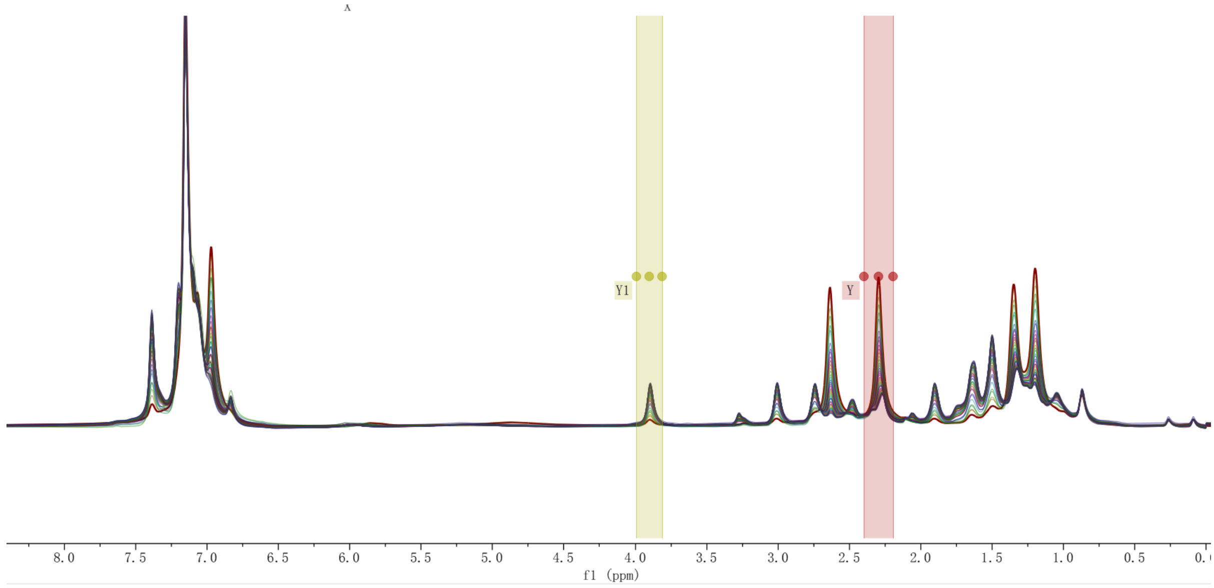

Figure S19. The reaction profile with 2 equiv TEMPO addition, the red line shows substrate conversion and yellow line shows product formation.

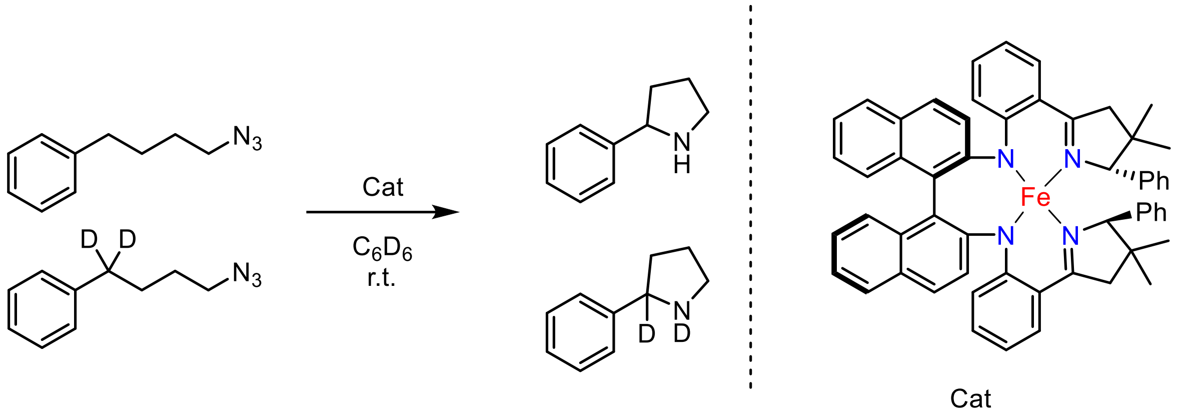

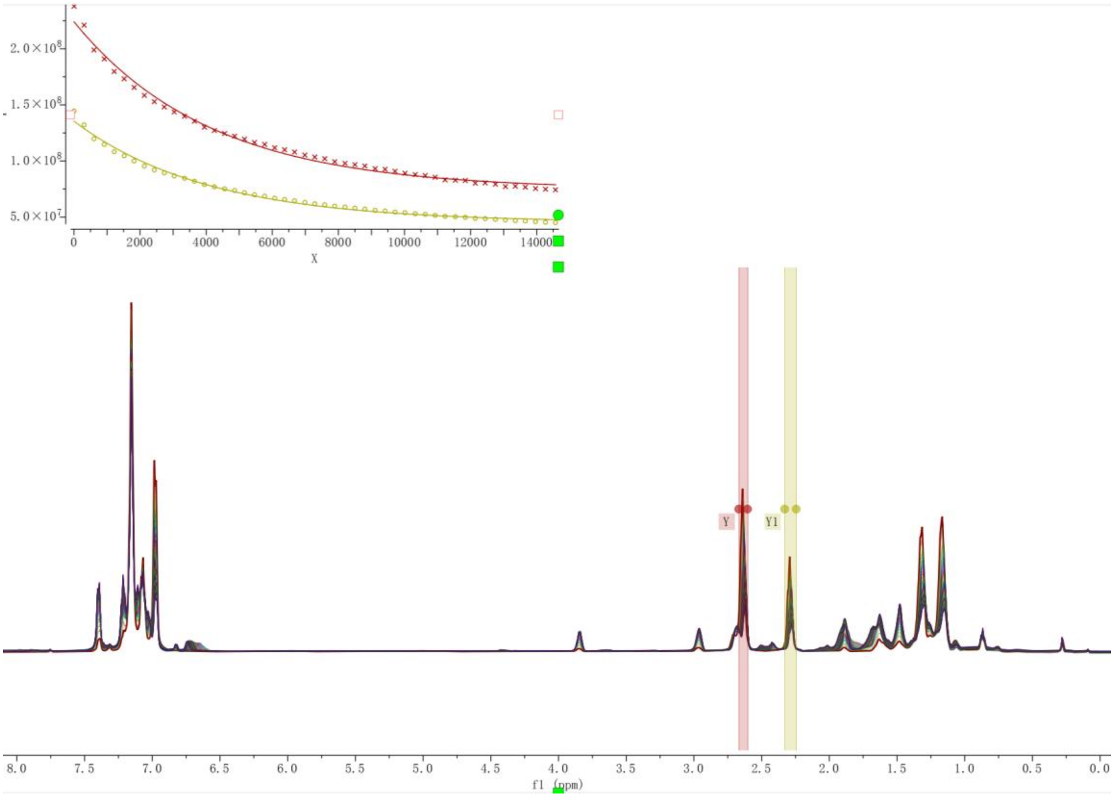

Figure S20. The reaction profile with 13.8 μL bis-deuterated azide and 13.8 μL non-deuterated azide addition, the red line shows substrate conversion (with non-deuterated) and yellow line shows substrate conversion (with half deuterated half non-deuterated).

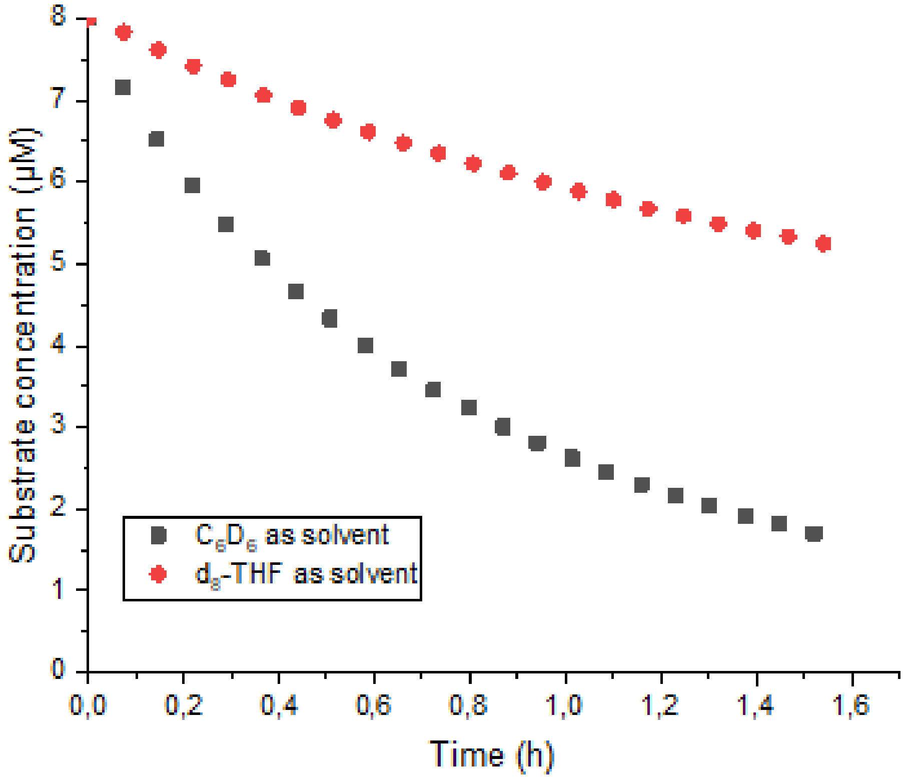

Figure S21. Comparative effect of THF-d_8 vs. benzene-d_6 as solvent for the C(sp^3)-H amination of the benchmark substrate (4-azidobutyl)benzene at room temperature with 14 mM catalyst (*R*,*S*,*S*)-**2** and 0.008 mmol substrate

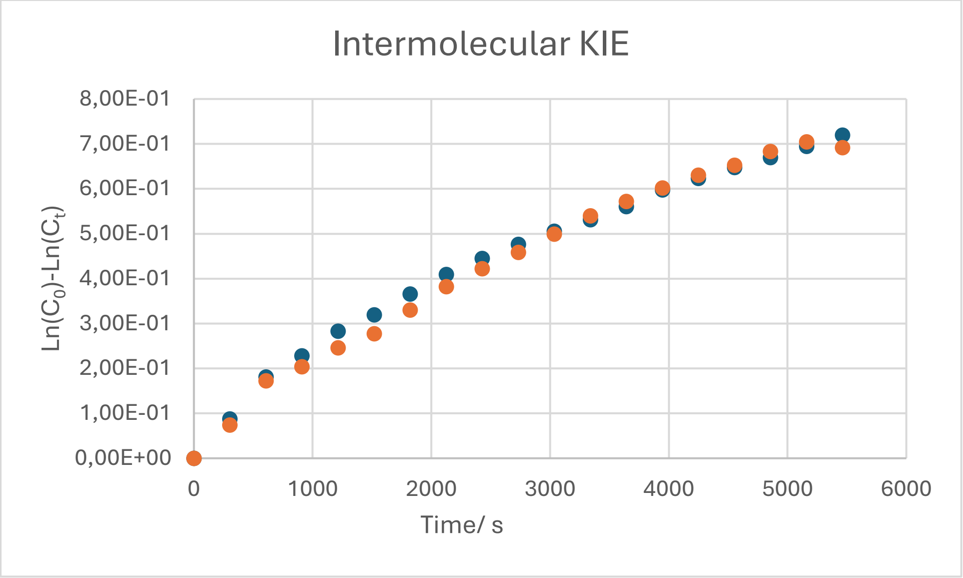

Figure S22. The kinetic profile with 13.8 μL bis-deuterated azide and 13.8 μL non-deuterated azide addition, the red line shows substrate conversion (with non-deuterated) and yellow line shows substrate conversion (with half deuterated half non-deuterated).

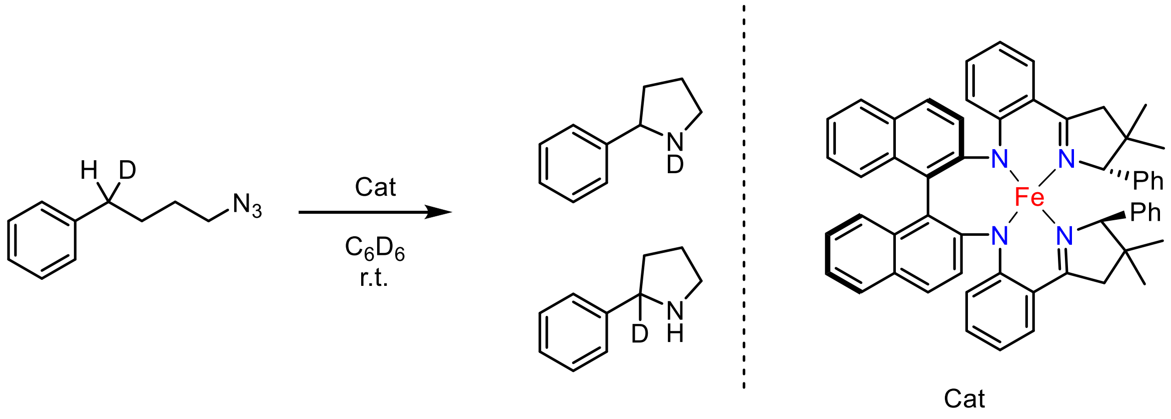

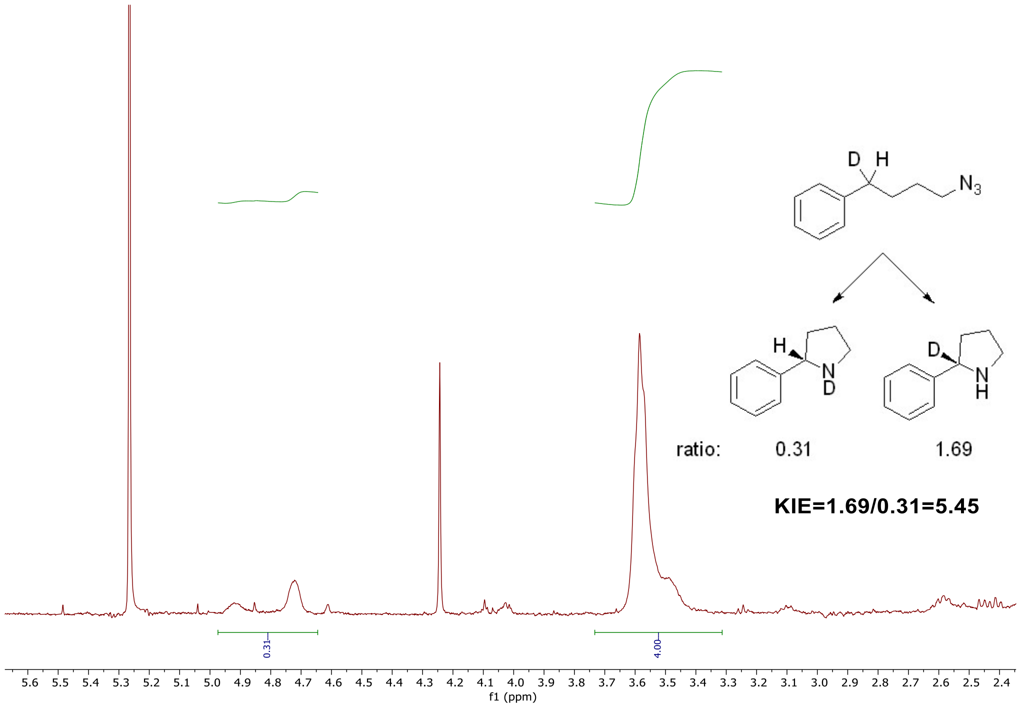

Figure S23. Representative ^{1}H-NMR spectrum of the product mixture (400 MHz, $CDCl_3$).

Catalysis

General procedure of catalysis and characterization of products

Under inert conditions (glovebox), Fe catalyst (7.5 µmol, 5 mol%) was dissolved in pentane, and the respective azide of the series **S2-S7** (0.15 mmol) was added. The reaction mixture was stirred at room temperature overnight.

The catalytic reaction resulted in the desired pyrrolidine (**P2-P7**) along with the corresponding imine side-product. After catalysis, Boc_2O was added to enable assessment of optical purity of the formed pyrrolidine. During work-up outside of the glovebox, the imine is side-product readily converted to the corresponding aldehyde when in contact with moisture from the air through hydrolysis. The mixture was concentrated under reduced pressure and the yields were determined via ^{1}H-NMR spectroscopy.

Substrate, pyrrolidine and aldehyde concentration were determined by comparing their reported peaks with literature and using 1,3,5-trimethoxybenzene as internal standard as follows:

^{1}H-NMR (400 MHz, $CDCl_3$) δ 9.78 (s, 1H, 4-phenylpentanal), δ 6.12 (s, 3H, 1,3,5-trimethoxybenzene), δ 4.89 (m, 1H, *tert*-butyl-2-phenylpyrrolidine-1-carboxylate), δ 3.31 (t, *J* = 6.7 Hz, 2H, (4-azidobutyl) benzene)

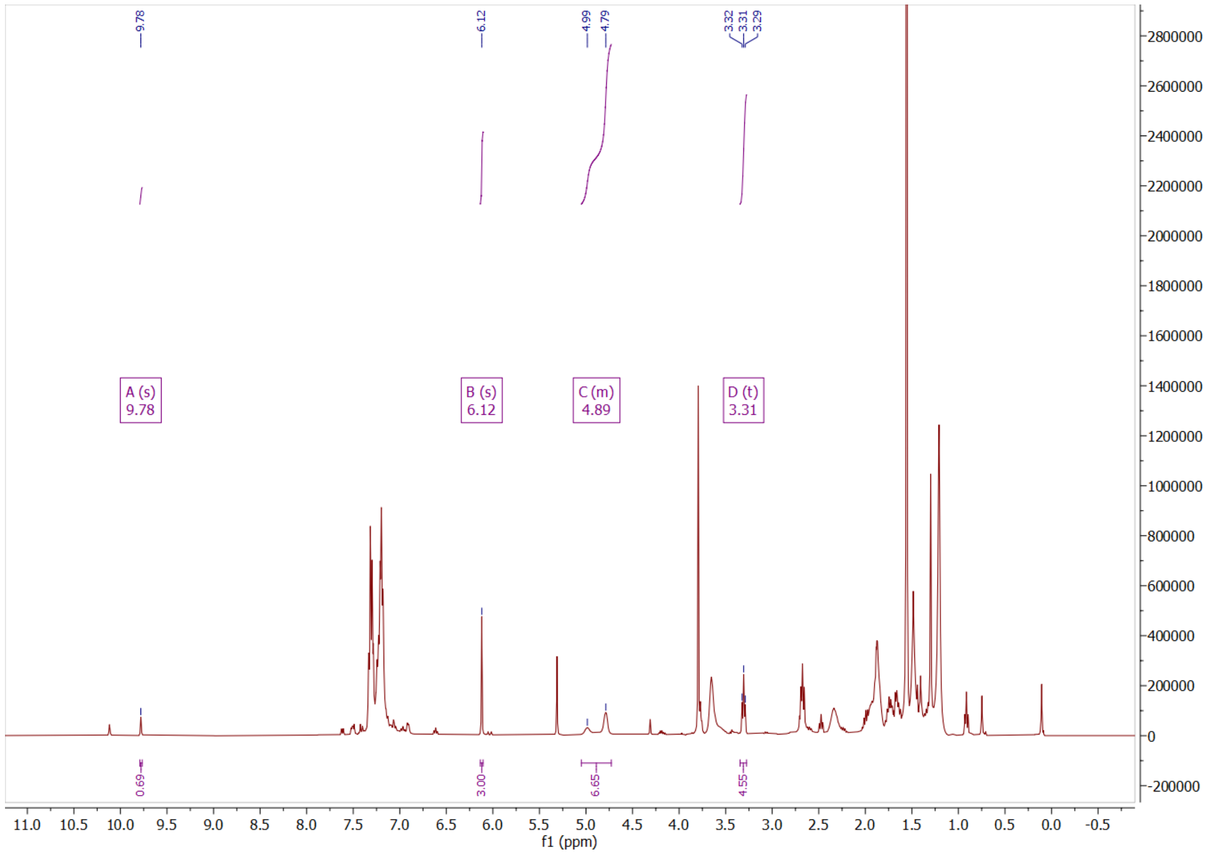

Figure S24. Crude NMR with internal standard after catalysis (400 MHz, $CDCl_3$).

The formed Boc-protected pyrrolidine products was purified by autocolumn and enantiomeric excess was determined via chiral GC or HPLC analysis.

Table S4. Control experiments

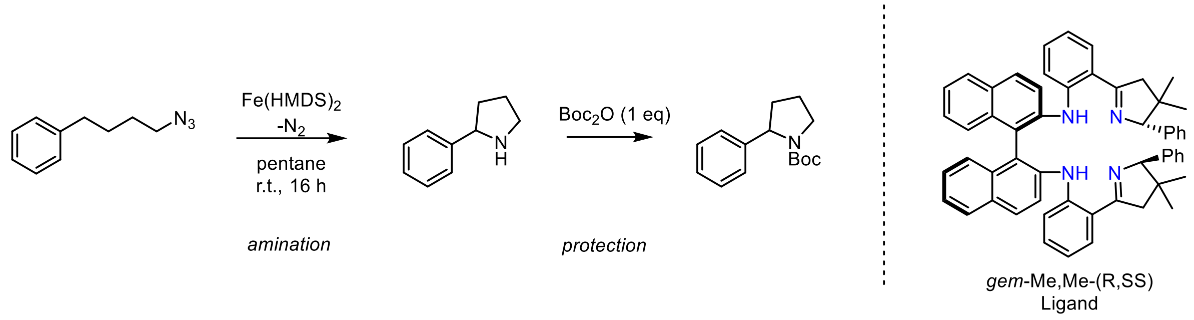

Entry	Deviation	Conversion (%)[a]	Yield (%)[a]
1	-	64	<5
2	Ligand (5 mol%)	95	47

Conditions: substrate (0.15 mmol) and $Fe(HMDS)_2$ (5 mol%) were stirred in pentane (2.5 mL) at r.t. for 16 h. [a]Calculated via ^1H NMR analysis with 1,3,5-trimethoxybenzene as internal standard.

Table S5. Comparison of solvents

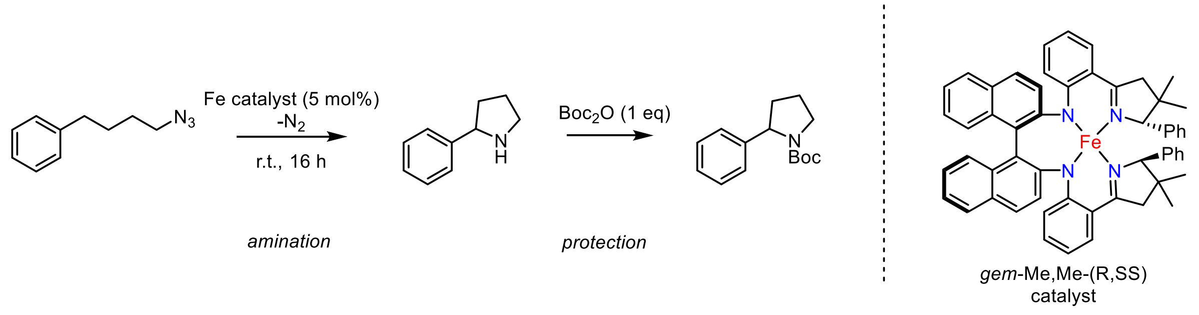

Entry	Solvent	Conversion (%)[a]	Yield (%)[a]	*ee* (%)
1	Pentane	79	62	65
2	Benzene	85	69	61 (-60)[b]
3	Diethyl ether	93	67	63
4	THF	59	46	55

Conditions: substrate (0.15 mmol) and *gem*-Me,Me-(*R*,*S*,*S*) catalyst (5 mol%) were stirred in pentane (2.5 ml) at r.t. for 16 h. [a]Calculated via ^1H NMR analysis with 1,3,5-trimethoxybenzene as internal standard. [b] *ee* recorded for opposite diastereomer (*S*,*R*,*R*) of the *gem*-Me,Me catalyst.

Table S6. Comparison of alkyl chains

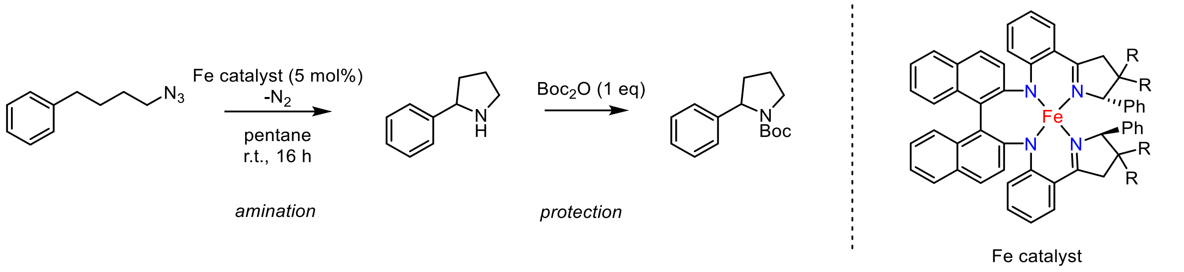

**Entry**	**R**	**Diastereomer**	**Conversion (%)**[a]	**Yield (%)**[a]	***ee* (%)**[b]
**1**	H	R,SS	16	11	-33
**2**	Me	R,SS	79	62	65
**3**	Me	R,RR	0	-	-
**4**	Et	R,SS	>99	87	68
**5**	nPr	R,SS	>99	86	78
**6**	nBu	R,SS	>99	82	79
**7**	nHex	R,SS	>99	79	81

Conditions: substrate (0.15 mmol) and Fe catalyst (5 mol%) were stirred in pentane (2.5 mL) at r.t. for 16 h. [a]Calculated via ^{1}H NMR analysis with 1,3,5-trimethoxybenzene as internal standard. [b]*ee* determined via chiral GC.

Table S7. Survey of ee vs temperature

**Entry**	**Temperature (°C)**	***ee* (%)**
1	25	81
2	50	76
3	-5	82

Conditions: substrate (0.15 mmol) and *gem*-nHex,nHex-(*R*,*S*,*S*) catalyst (5 mol%) were stirred in pentane (2.5 mL) for 16 h. [a]*ee* determined via chiral GC.

Table S8. Concentration and catalyst loading vs *ee*

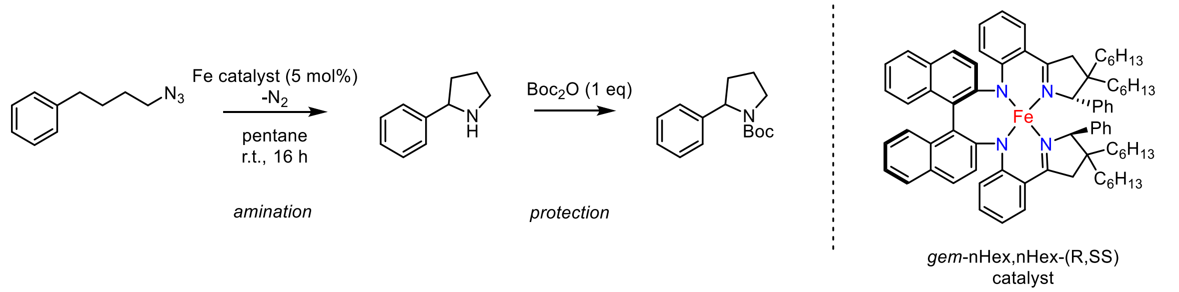

Entry	Volume (mL)	Catalyst loading (mol%)	*ee* (%)
1	2.5	2.5	80
2	5.0	5.0	80
3	2.5	7.5	80

Conditions: substrate (0.15 mmol) and *gem*-nHex,nHex-(*R*,*S*,*S*) catalyst were stirred in pentane at r.t. for 16 h. [a]Calculated via ^{1}H NMR analysis with 1,3,5-trimethoxybenzene as internal standard. [b]*ee* determined via chiral GC.

Table S9. Enantioselectivity over time

Entry	Time (h)	*ee* (%)
1	0.5	80
2	1	80
3	1.5	80
4	2	80
5	2.5	80

Conditions: substrate (0.15 mmol) and *gem*-nHex,nHex-(*R*,*S*,*S*) catalyst (5 mol%) were stirred in pentane (2.5 mL) for 16 h. [a]ee determined via chiral GC.

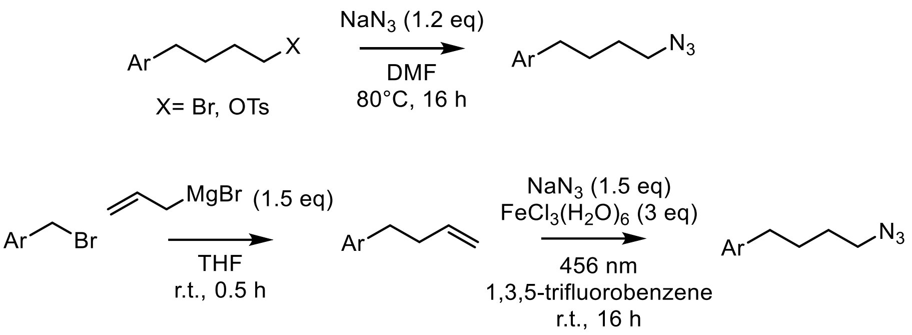

Figure S25. Multiple synthetic routes for azide synthesis

Table S10. Imine formation

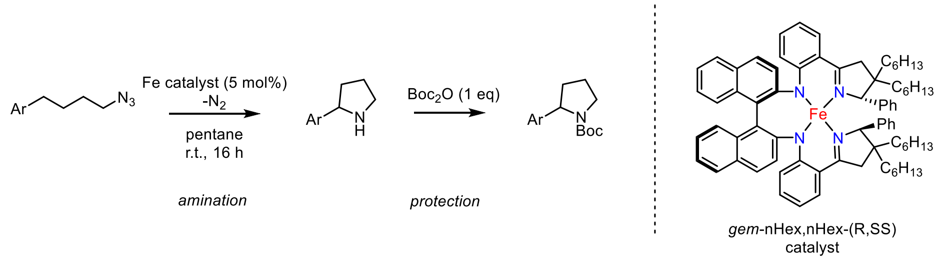

**Substrate**	**Pyrrolidine (%)**	**Imine (%)**
benchmark	79	20
Para-chloro	86	<5
Para-fluoro	73	<5
2,5-difluoro	76	<5
3,5-dimethyl	58	<5
3,4-dimethoxy	64[b]	<5[b]
pyridine	91[b]	<5[b]

Conditions: substrate (0.15 mmol) and *gem*-nHex,nHex-(*R*,*S*,*S*) catalyst (5 mol%) were stirred in pentane (2.5 mL) at r.t. for 16 h unless stated otherwise. Determined *ee* values via chiral HPLC unless stated otherwise. Yields calculated via ^{1}H NMR analysis with 1,3,5-trimethoxybenzene as internal standard. [a]Reaction performed with *gem*-nHex,nHex-(*R*,*S*,*S*) catalyst (10 mol%) in benzene (0.6 mL) at r.t. for 16 h.

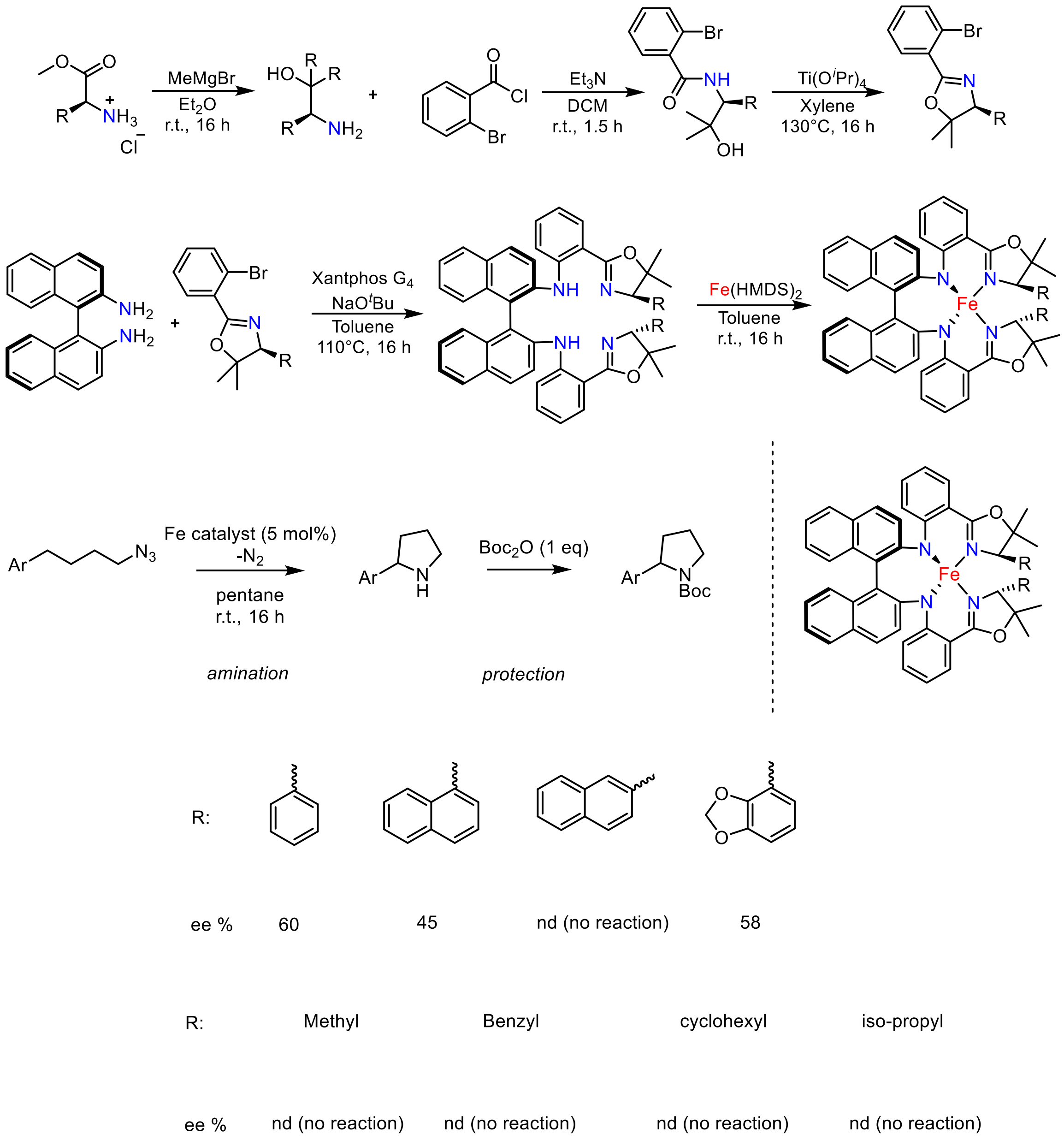

Figure S26. Preliminary results of different *R*-substitutes in oxazoline for catalysis.

***tert*-butyl-2-phenylpyrrolidine-1-carboxylate**

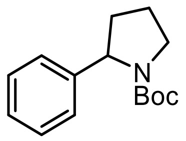

From **S1** following the general procedure to afford **P1** (79%). Enantiomeric excess determined via chiral GC (Supelco β-dex 235 capillary column) analysis. Method: 70°C, hold for 2 min., then 2°C/min to 90°C, hold for 1 min., then 10°C/min to 225°C, hold for 2 min. *ee* = 81%.

^{1}H NMR (300 MHz, $CDCl_3$) δ 7.35 – 7.28 (m, 2H), 7.25 – 7.15 (m, 3H), 5.08 – 4.70 (m, 1H), 3.71 – 3.51 (m, 2H), 2.41 – 2.21 (m, 1H), 2.00 – 1.78 (m, 3H), 1.48 (m, 3H), 1.20 (m, 6H) (two different signals due to restriction of rotation).

tert-butyl-2-(4-chlorophenyl)pyrrolidine-1-carboxylate

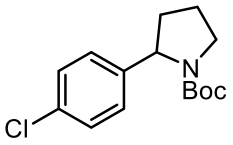

From **S2** following the general procedure to afford **P2** (86%). Enantiomeric excess determined via chiral HPLC analysis using a Daicel Chiralpak **IG** column, *ee* = 66% (220 nm, hexanes/isopropanol = 99:1, flow rate 1.0 mL/min, 25°C, t_r (major) = 11.3 min, t_r (minor) = 9.3 min)

^{1}H NMR (400 MHz, $CDCl_3$) δ 7.26 – 7.23 (m, 2H), 7.12 – 7.06 (m, 2H), 4.96 – 4.67 (m, 1H), 3.67 – 3.47 (m, 2H), 2.36 – 2.20 (m, 1H), 1.94 – 1.72 (m, 3H), 1.42 – 1.39 (m, 3H), 1.26 – 1.11 (m, 6H).

Purified with autocolumn. Flash Silica, 4 g (pentane/ethyl acetate). Running scheme t(minutes): t0-4 EtAc 0%, t4-30 EtAc 5%. Product collection in t4-30.

tert-butyl-2-(4-fluorophenyl)pyrrolidine-1-carboxylate

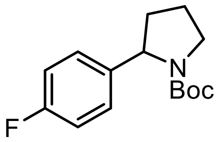

From **S3** following the general procedure to afford **P3** (73%). Enantiomeric excess determined via chiral HPLC analysis using a Daicel Chiralpak **IG** column, *ee* = 64% (254 nm, hexanes/isopropanol = 99:1, flow rate 1.0 mL/min, 25°C, t_r (major) = 10.7 min, t_r (minor) = 10.0 min)

^{1}H NMR (300 MHz, $CDCl_3$) δ 7.17 – 7.07 (m, 2H), 6.97 (tt, 2H), 4.99 – 4.64 (m, 1H), 3.66 – 3.46 (m, 2H), 2.39 – 2.20 (m, 1H), 1.91 – 1.70 (m, 3H), 1.50 – 1.37 (m, 3H), 1.26 – 1.12 (m, 6H).

Purified with autocolumn. Flash Silica, 4 g (pentane/ethyl acetate). Running scheme t(minutes): t0-10 EtAc 0%, t10-40 EtAc 5%. Product collection in t10-40.

tert-butyl-2-(3,4-dimethoxyphenyl)pyrrolidine-1-carboxylate

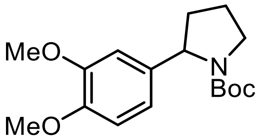

From **S4** following the general procedure to afford **P4** (64%). Enantiomeric excess determined via chiral HPLC analysis using a Daicel Chiralpak **OJ-H** column, *ee* = 63% (220 nm, hexanes/isopropanol = 90:10, flow rate 1.0 mL/min, 25°C, t_r (major) = 6.4 min, t_r (minor) = 5.8 min)

^{1}H NMR (300 MHz, $CDCl_3$) δ 6.83 – 6.76 (m, 1H), 6.73 – 6.66 (m, 2H), 4.98 – 4.62 (m, 1H), 3.85 (s, 6H), 3.68 – 3.40 (m, 2H), 2.27 (s, 1H), 1.96 – 1.76 (m, 3H), 1.49 – 1.35 (m, 3H), 1.28 – 1.15 (m, 6H).

Purified with autocolumn. Flash Silica, 4 g (pentane/ethyl acetate). Running scheme t(minutes): t0-10 EtAc 0%, t10-40 EtAc 15%. Product collection in t10-40.

***tert*-butyl-2-(pyridin-3-yl)pyrrolidine-1-carboxylate**

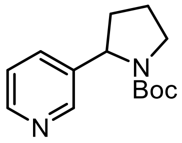

From **S5** following the general procedure to afford **P4** (91%). Enantiomeric excess determined via chiral HPLC analysis using a Daicel Chiralpak **AD-H** column, *ee* = 52% (254 nm, hexanes/isopropanol = 95:5, flow rate 1.0 mL/min, 25°C, t_r (major) = 21.7 min, t_r (minor) = 26.2 min)

^{1}H NMR (400 MHz, $CDCl_3$) δ 8.51 – 8.43 (m, 2H), 7.48 (d, *J* = 5.9 Hz, 1H), 7.26 – 7.18 (m, 2H), 5.03 – 4.70 (m, 1H), 3.70 – 3.48 (m, 2H), 2.43 – 2.26 (m, 1H), 1.94 – 1.79 (m, 3H), 1.52 – 1.38 (m, 3H), 1.28 – 1.12 (m, 6H).

Purified with autocolumn. Flash Silica, 4 g (pentane/ethyl acetate). Running scheme t(minutes): t0-4 EtAc 1%, t4-44 EtAc 50%, t44-t54 EtAc 100%. Product collection in t44-54.

***tert*-butyl-2-(2,5-difluorophenyl)pyrrolidine-1-carboxylate**

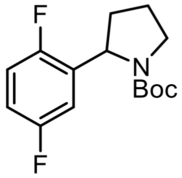

From **S6** following the general procedure to afford **P6** (76%). Enantiomeric excess determined via chiral HPLC analysis using a Daicel Chiralpak **IG** column, *ee* = 53% (220 nm, hexanes/isopropanol = 99:1, flow rate 1.0 mL/min, 25°C, t_r (major) = 10.0 min, t_r (minor) = 8.5 min)

^{1}H NMR (300 MHz, $CDCl_3$) δ 7.00 – 6.77 (m, 3H), 5.18 – 4.95 (m, 1H), 3.64 – 3.42 (m, 2H), 2.43 – 2.22 (m, 1H), 1.93 – 1.77 (m, 3H), 1.49 – 1.41 (m, 3H), 1.27 – 1.18 (m, 6H).

Purified with autocolumn. Flash Silica, 4 g (pentane/ethyl acetate). Running scheme t(minutes): t0-10 EtAc 0%, t10-40 EtAc 5%. Product collection in t10-40.

***tert*-butyl-2-(3,5-dimethylphenyl)pyrrolidine-1-carboxylate**

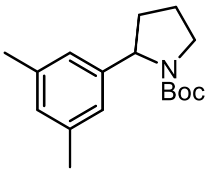

From **S7** following the general procedure to afford **P7** (58%). Enantiomeric excess not determined.

^{1}H NMR (400 MHz, $CDCl_3$) δ 6.83 (s, 1H), 6.76 (s, 2H), 4.90 – 4.65 (m, 1H), 3.68 – 3.46 (m, 2H), 2.28 (s, 6H), 2.00 – 1.90 (m, 1H), 1.90 – 1.74 (m, 3H), 1.48 – 1.40 (m, 3H), 1.23 – 1.11 (m, 6H).

Chiral GC spectra

Supelco β-dex 235 capillary column. Method: 70 °C, hold for 2 min., then 2 °C/min to 90°C, hold for 1 min., then 10 °C/min to 225 °C, hold for 2 min.

Racemic product *tert*-butyl-2-phenylpyrrolidine-1-carboxylate

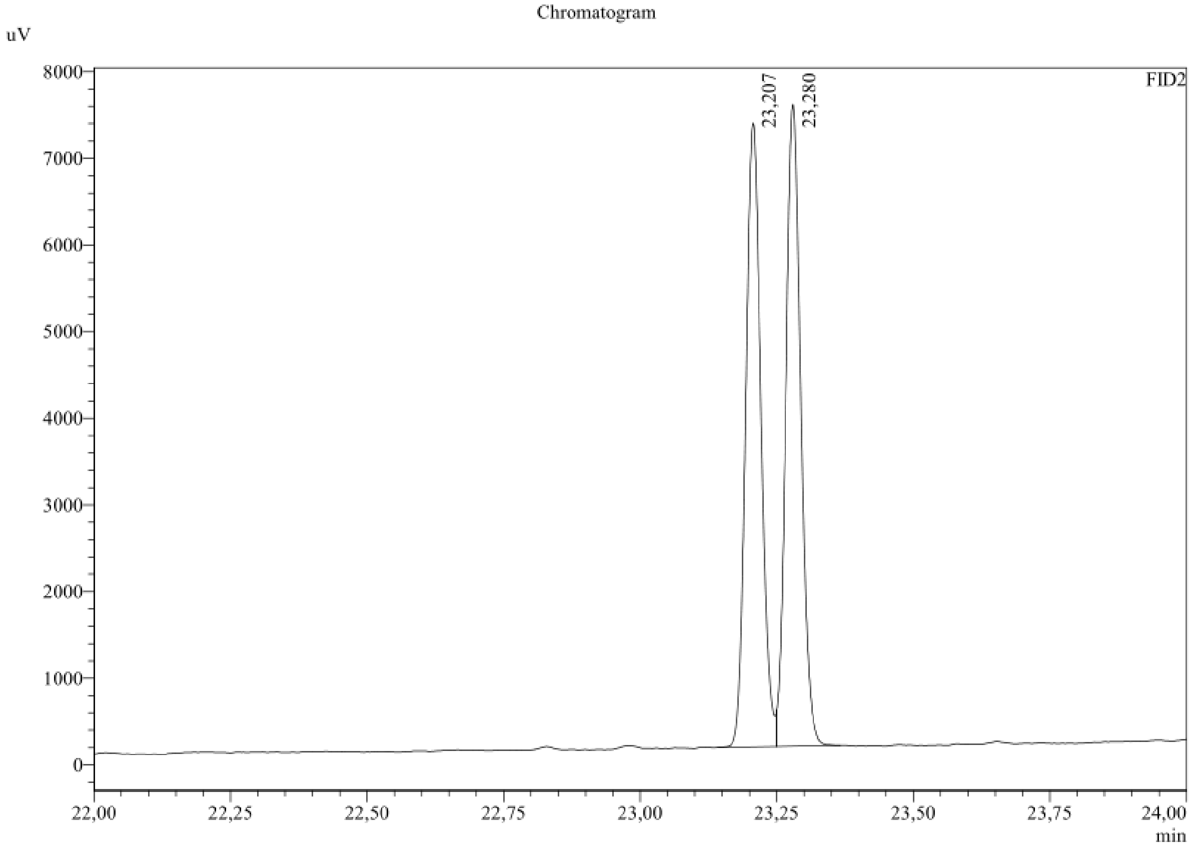

Peak Table

FID2

Peak#	Ret. Time	Area	Height	Area%	Name
1	23,207	14135	7148	50,086	
2	23,280	14086	7347	49,914	
Total		28221	14496	100,000	

Solvent screening

Pentane

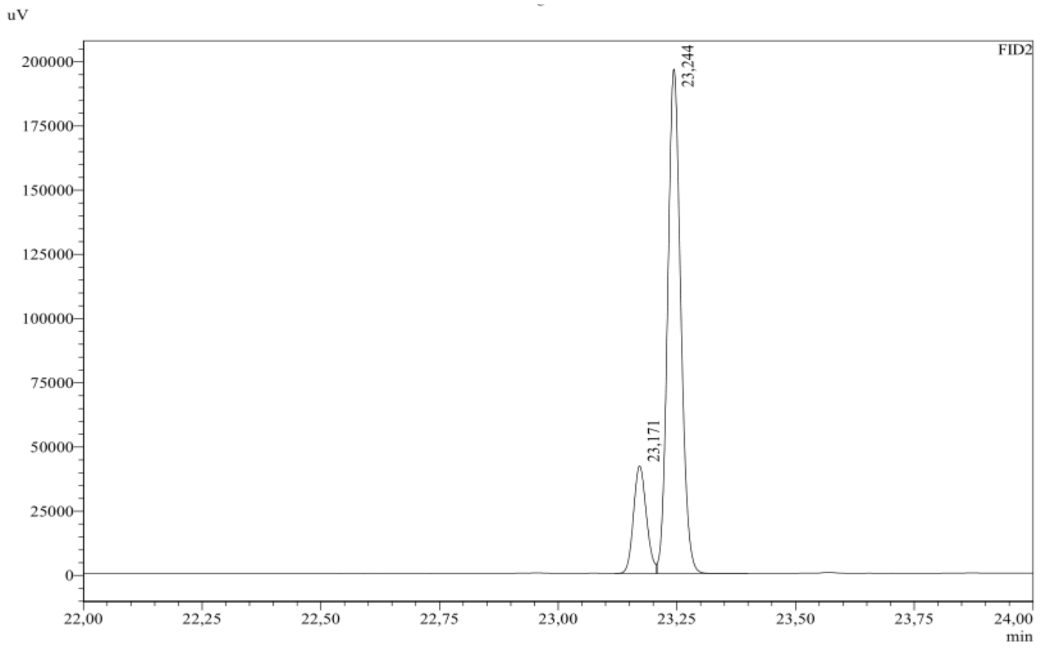

Peak Table

FID2

Peak#	Ret. Time	Area	Height	Area%	Name
1	23,171	79486	41268	17,421	
2	23,244	376770	194842	82,579	
Total		456256	236110	100,000	

Benzene

Chromatogram
Stijn_99 C:\LabSolutions\Data\Project1\Daniel\Stijn_99.gcd

Chromatogram

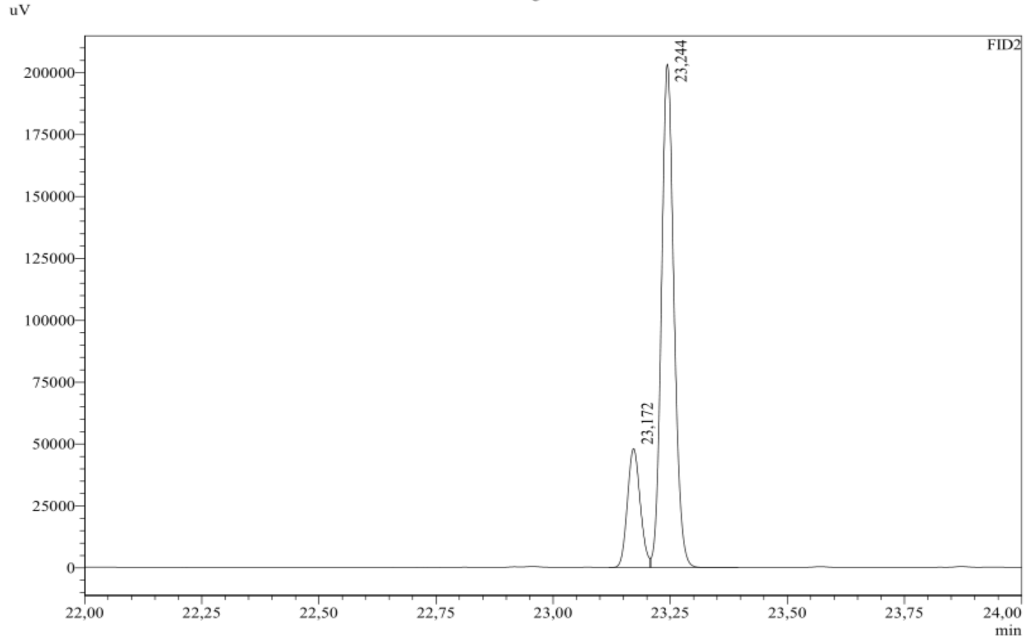

Peak Table

FID2

Peak#	Ret. Time	Area	Height	Area%	Name
1	23,172	91557	47403	19,385	
2	23,244	380747	201774	80,615	
Total		472304	249177	100,000	

THF

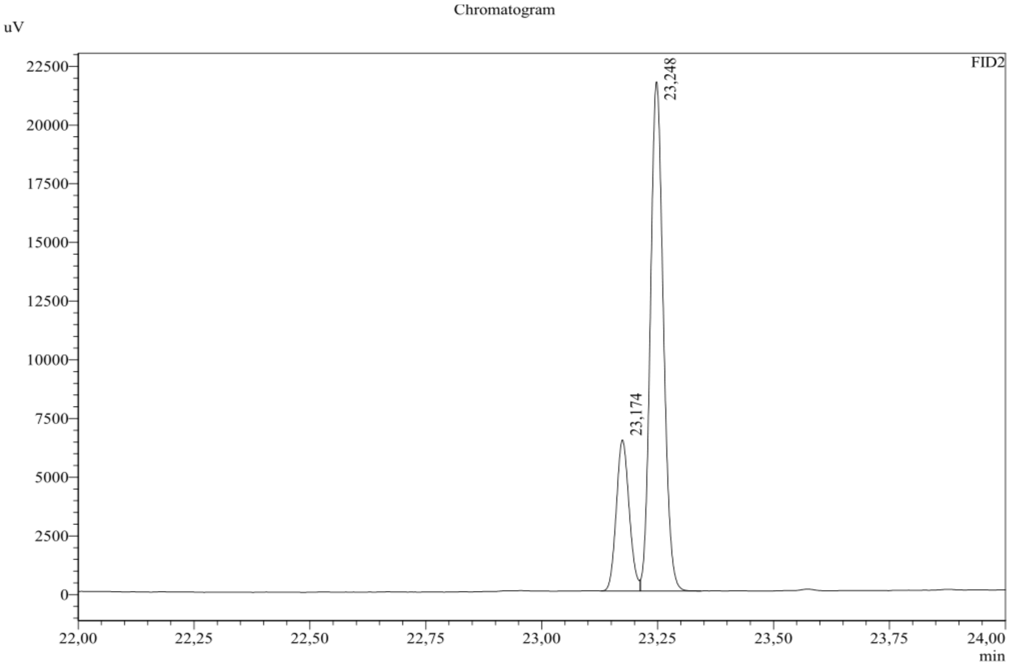

Peak Table

FID2

Peak#	Ret. Time	Area	Height	Area%	Name
1	23,174	12438	6387	22,736	
2	23,248	42269	21566	77,264	
Total		54708	27953	100,000	

Diethyl ether

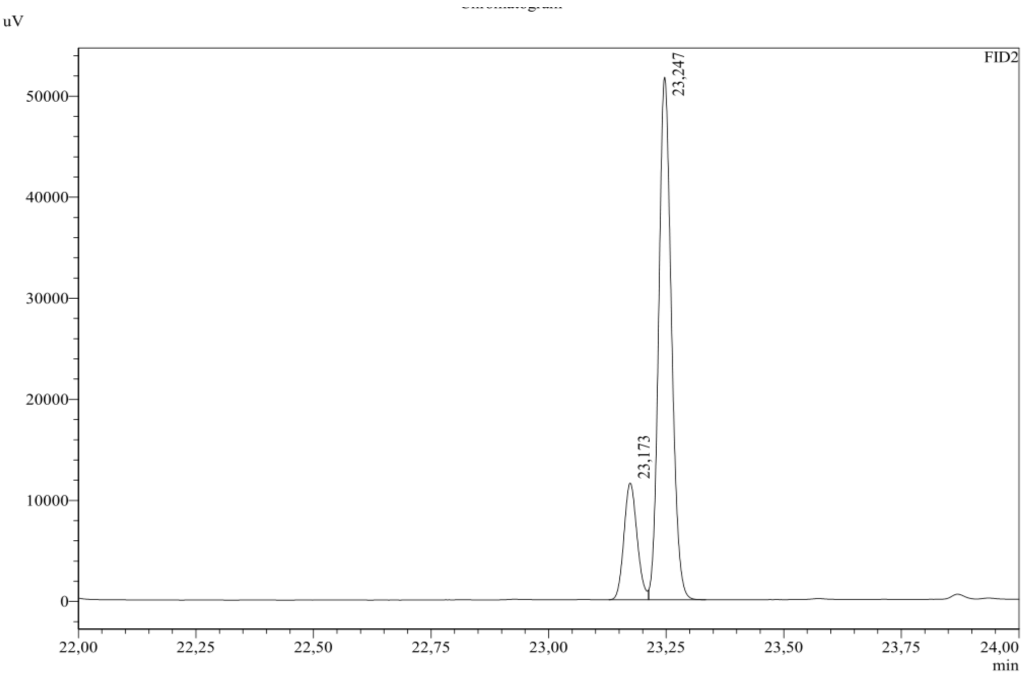

Peak Table

FID2

Peak#	Ret. Time	Area	Height	Area%	Name
1	23,173	22459	11475	18,698	
2	23,247	97657	51154	81,302	
Total		120116	62629	100,000	

Comparison of alkyl chains

gem-H,H-(R,SS)

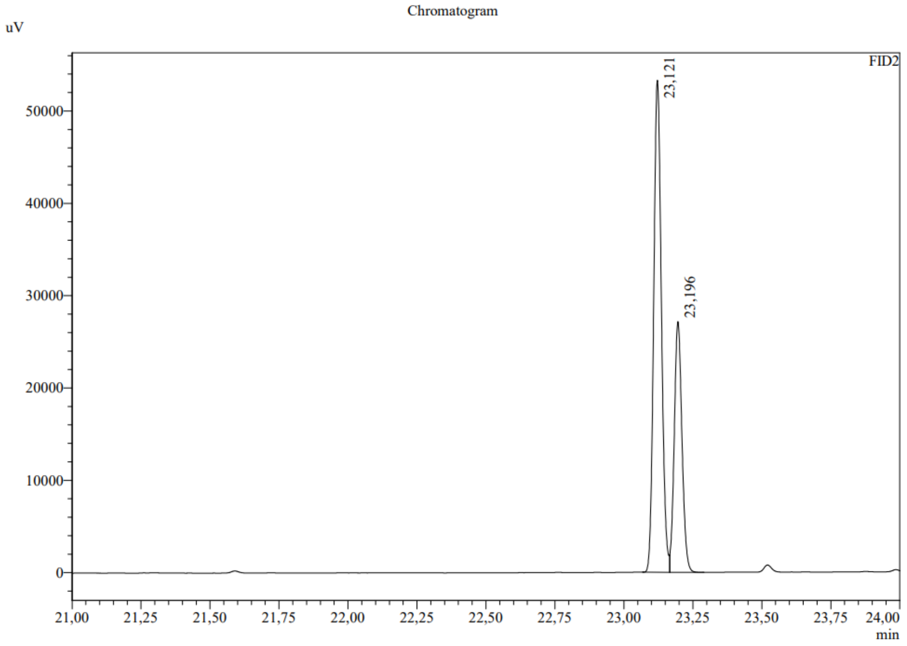

Peak Table

FID2

Peak#	Ret. Time	Area	Height	Area%	Name
1	23,121	103543	52919	66,233	
2	23,196	52789	26959	33,767	
Total		156332	79879	100,000	

gem-Me,Me-(*R*,*S*,*S*)

Is the spectrum in pentane from solvent screening

gem-Me,Me-(*S*,*R*,*R*)

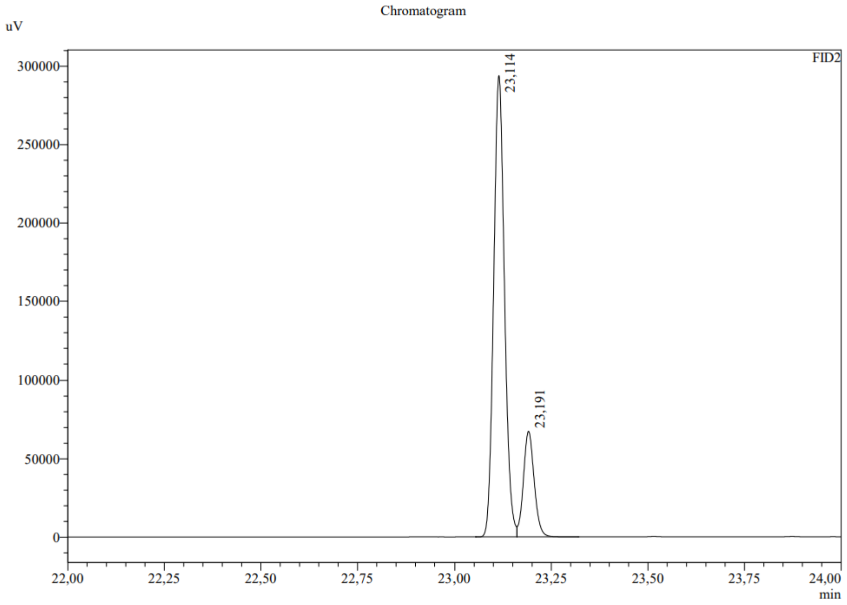

Peak Table

FID2

Peak#	Ret. Time	Area	Height	Area%	Name
1	23,114	571393	288443	81,336	
2	23,191	131112	66945	18,664	
Total		702505	355387	100,000	

***gem*-Et,Et-(*R*,*S*,*S*)**

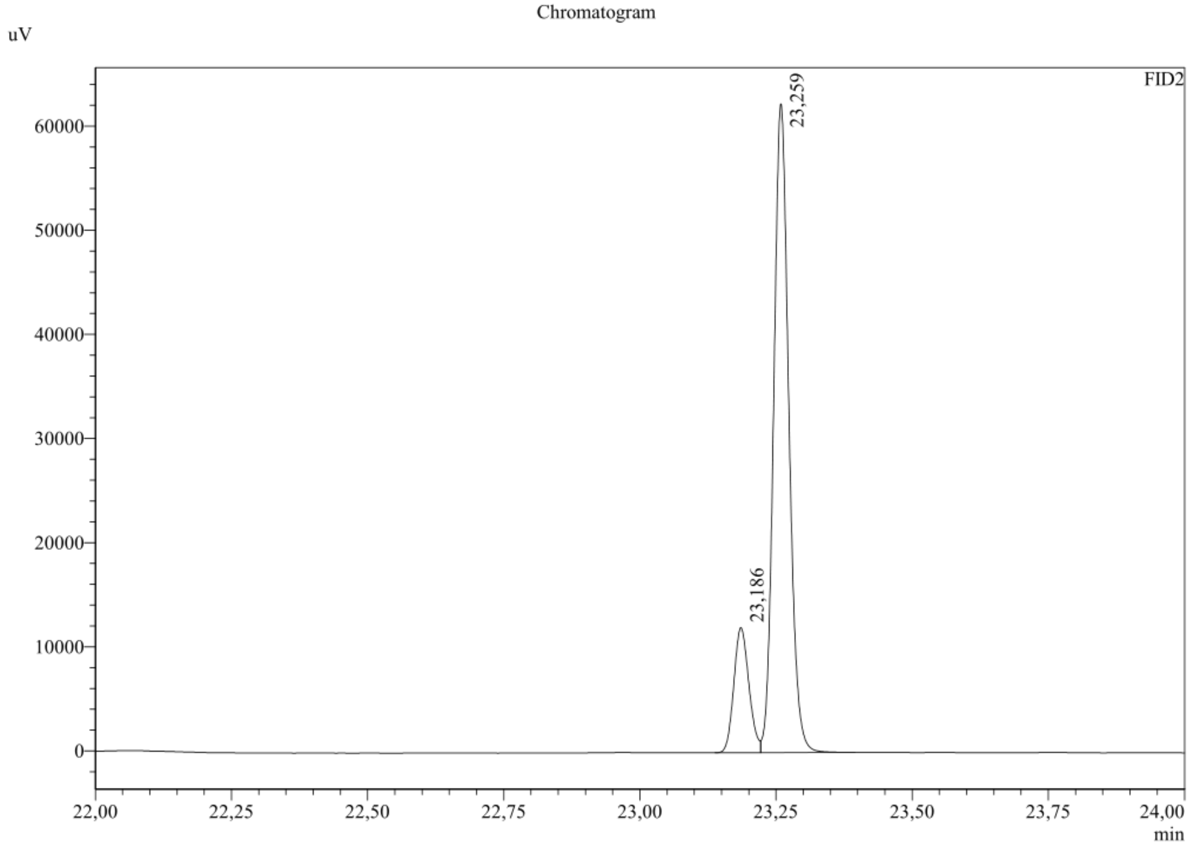

Peak Table

FID2

Peak#	Ret. Time	Area	Height	Area%	Name
1	23,186	23229	11807	16,170	
2	23,259	120430	61423	83,830	
Total		143659	73230	100,000	

***gem*-nPr,nPr-(*R*,*S*,*S*)**

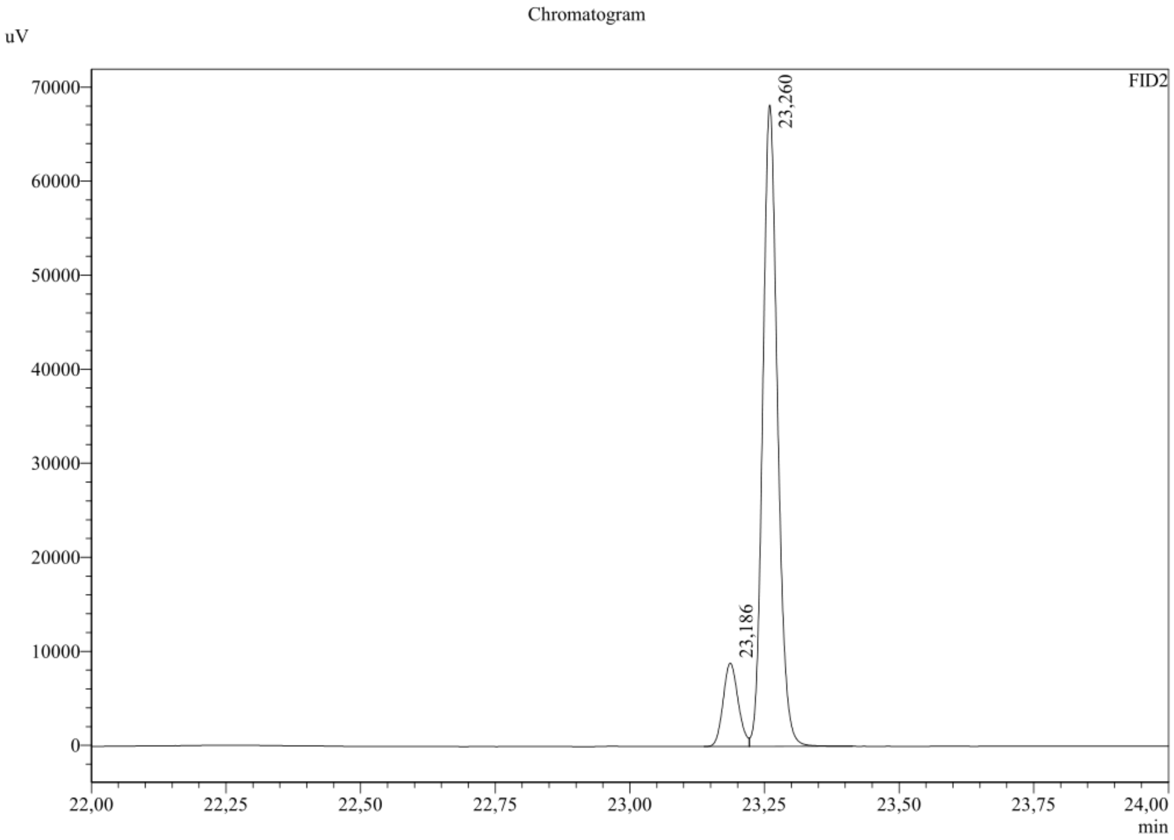

Peak Table

FID2

Peak#	Ret. Time	Area	Height	Area%	Name
1	23,186	17245	8789	11,674	
2	23,260	130476	66564	88,326	
Total		147721	75352	100,000	

***gem*-nBu,nBu-(*R*,*S*,*S*)**

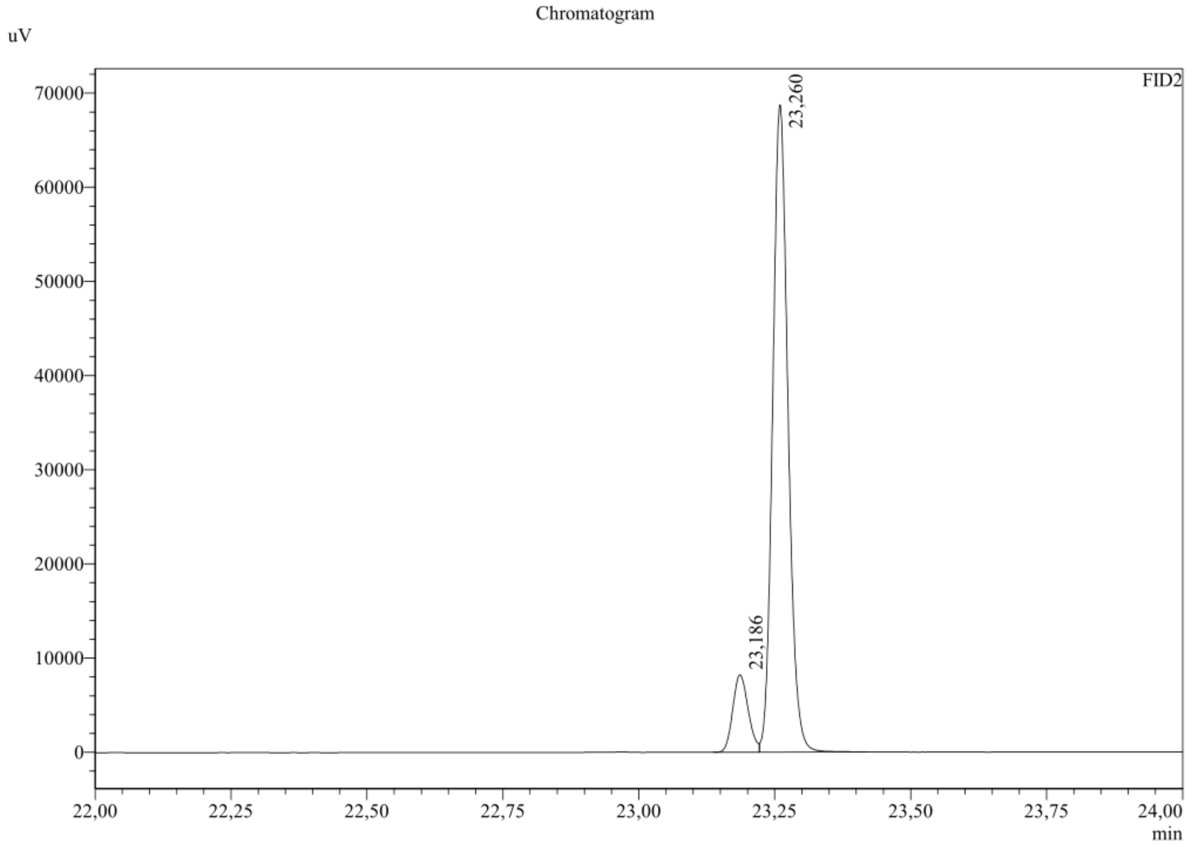

Peak Table

FID2

Peak#	Ret. Time	Area	Height	Area%	Name
1	23,186	16274	8142	10,774	
2	23,260	134773	67287	89,226	
Total		151047	75428	100,000	

***gem*-nHex,nHex-(*R*,*S*,*S*)**

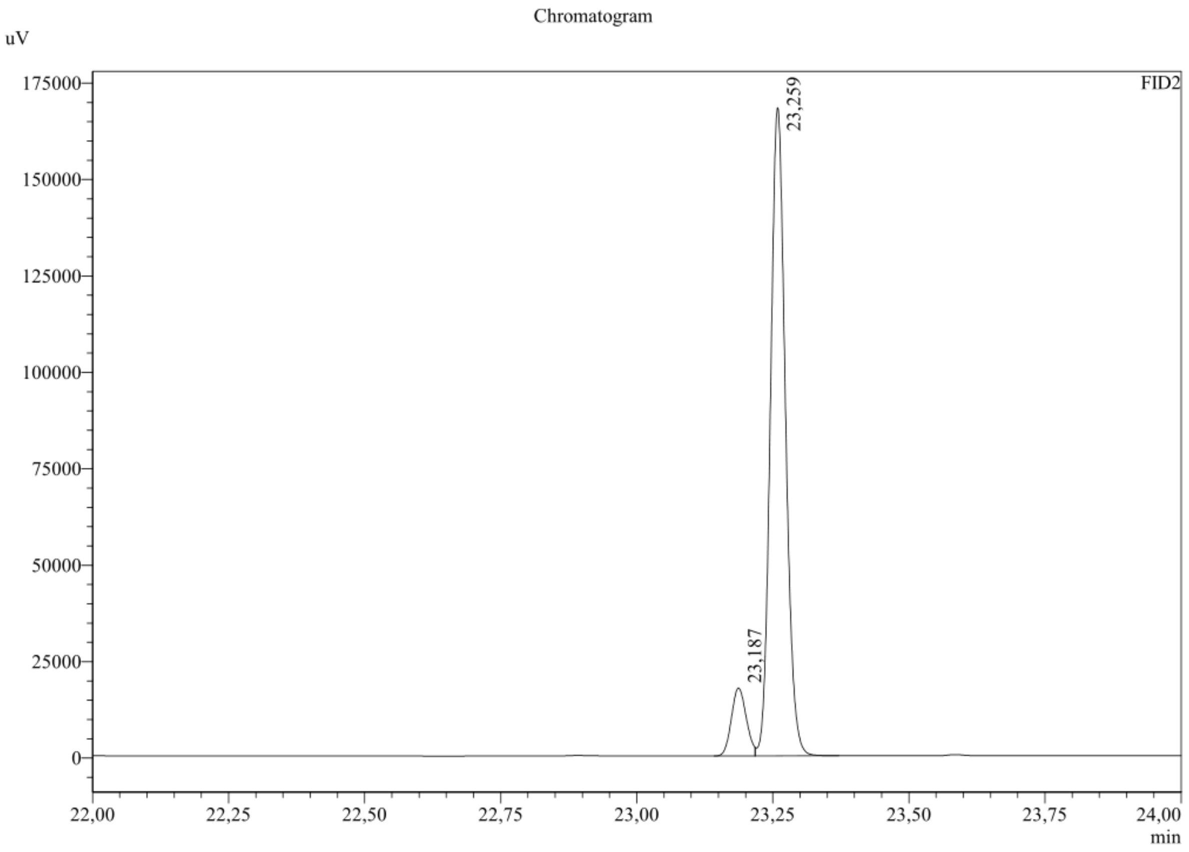

Peak Table

FID2

Peak#	Ret. Time	Area	Height	Area%	Name
1	23,187	33469	17515	9,386	
2	23,259	323125	165764	90,614	
Total		356594	183279	100,000	

Concentration and catalyst loading vs *ee*

2.5 mL, 2.5 mol%

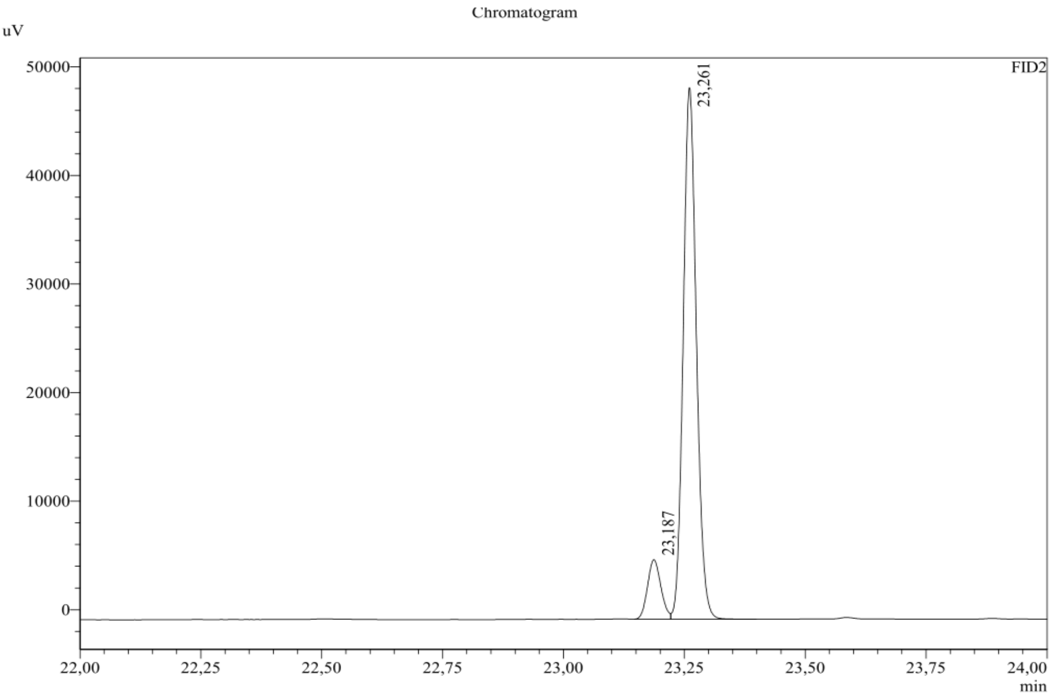

Peak Table

FID2

Peak#	Ret. Time	Area	Height	Area%	Name
1	23,187	10549	5452	10,223	
2	23,261	92641	48527	89,777	
Total		103190	53979	100,000	

5.0 mL, 5.0 mol%

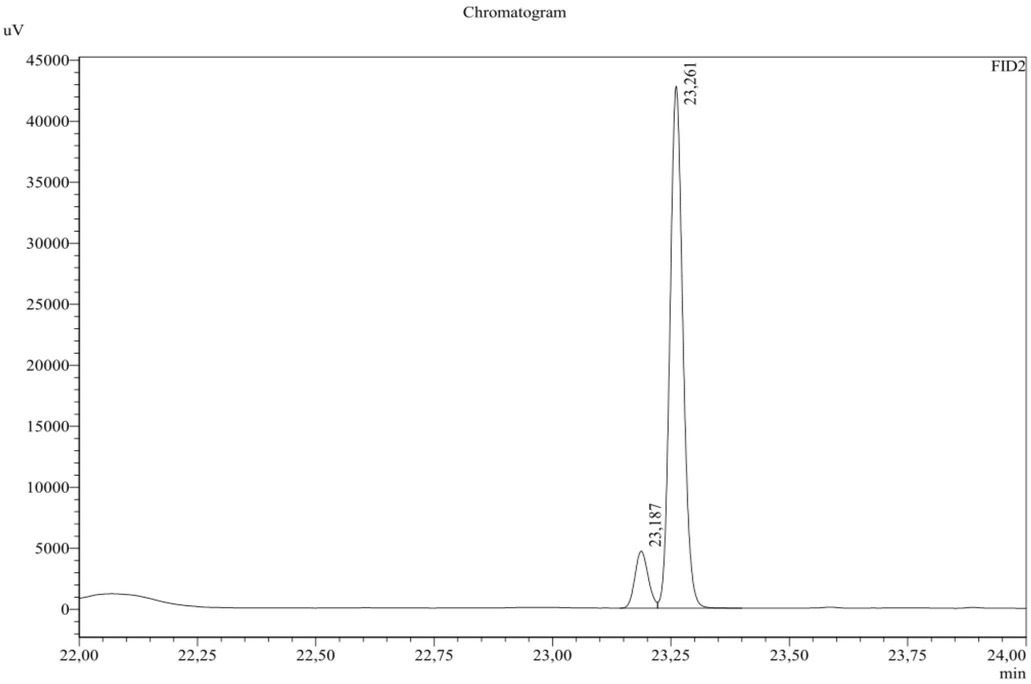

Peak Table

FID2

Peak#	Ret. Time	Area	Height	Area%	Name
1	23,187	9104	4646	10,031	
2	23,261	81653	42464	89,969	
Total		90758	47110	100,000	

2.5 mL, 7.5 mol%

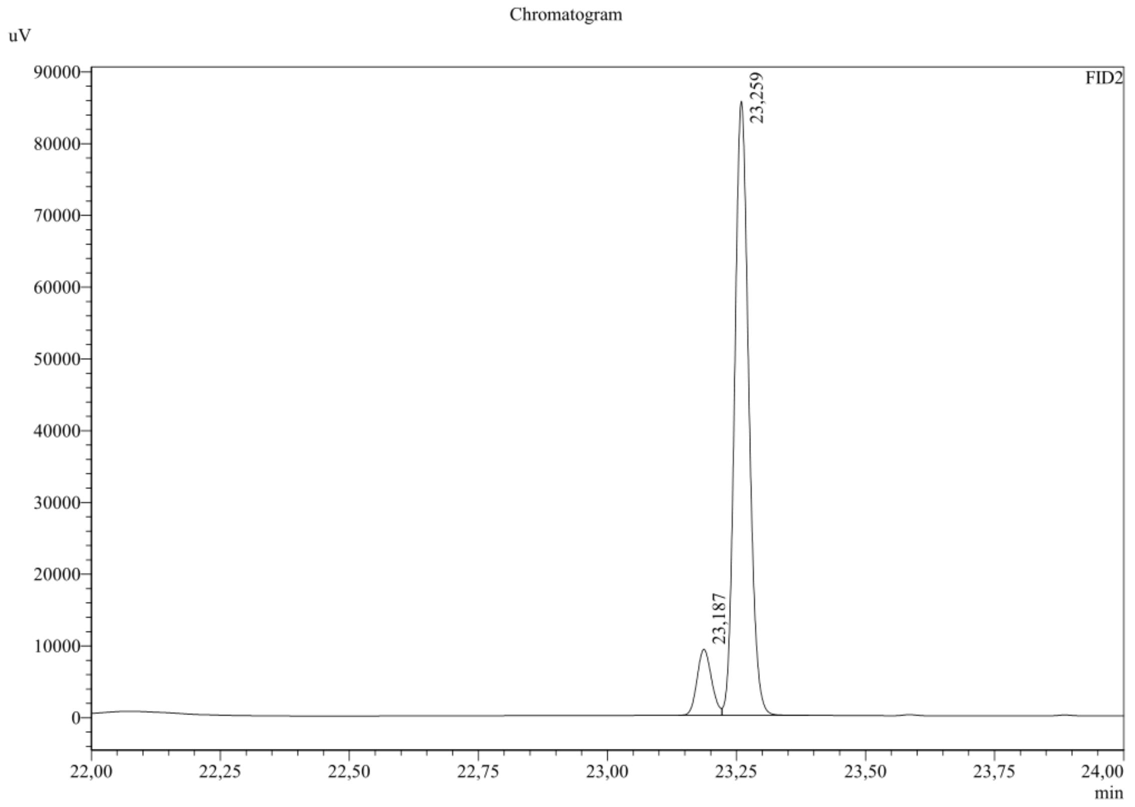

Peak Table

FID2

Peak#	Ret. Time	Area	Height	Area%	Name
1	23,187	17856	9136	9,862	
2	23,259	163199	83794	90,138	
Total		181055	92930	100,000	

Survey of *ee* vs temperature

Room temperature

The same spectrum in pentane from solvent screening

50°C

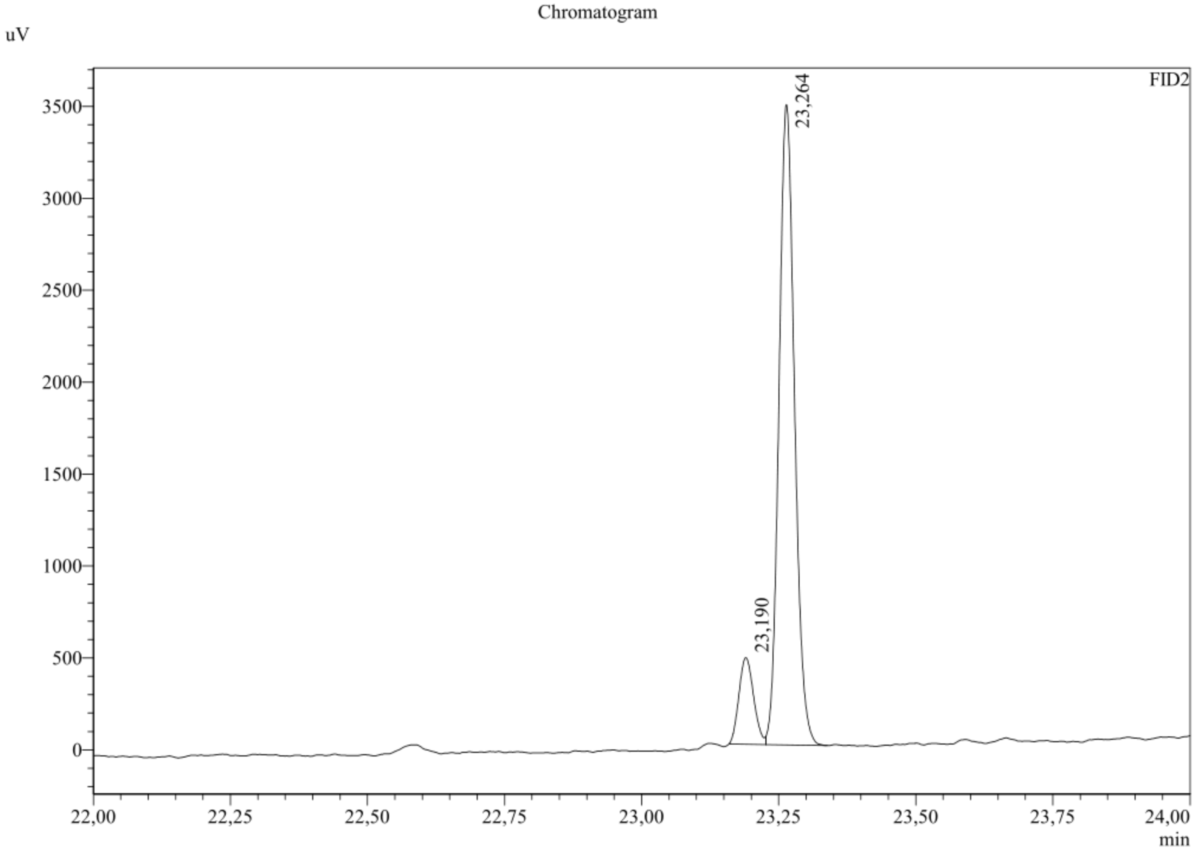

Peak Table

FID2

Peak#	Ret. Time	Area	Height	Area%	Name
1	23,190	885	465	11,361	
2	23,264	6902	3407	88,639	
Total		7786	3872	100,000	

-5°C

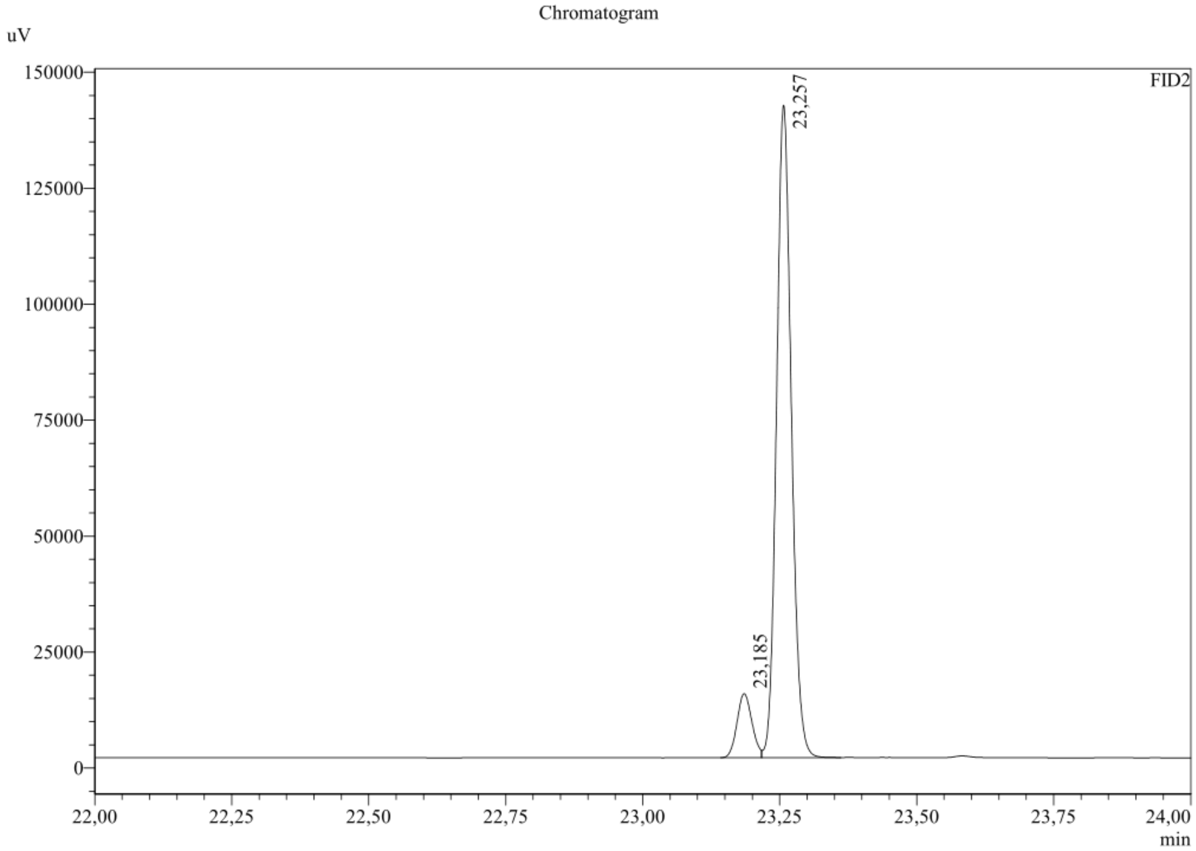

Peak Table

FID2

Peak#	Ret. Time	Area	Height	Area%	Name
1	23,185	26272	13582	9,029	
2	23,257	264691	139807	90,971	
Total		290963	153390	100,000	

Enantioselectivity over time

Conditions: substrate (0.15 mmol) and *gem*-nHex,nHex-(*R*,*S*,*S*) catalyst (5 mol%) were stirred in pentane (2.5 mL) for 16 h. *ee* determined via chiral GC.

0.5 h

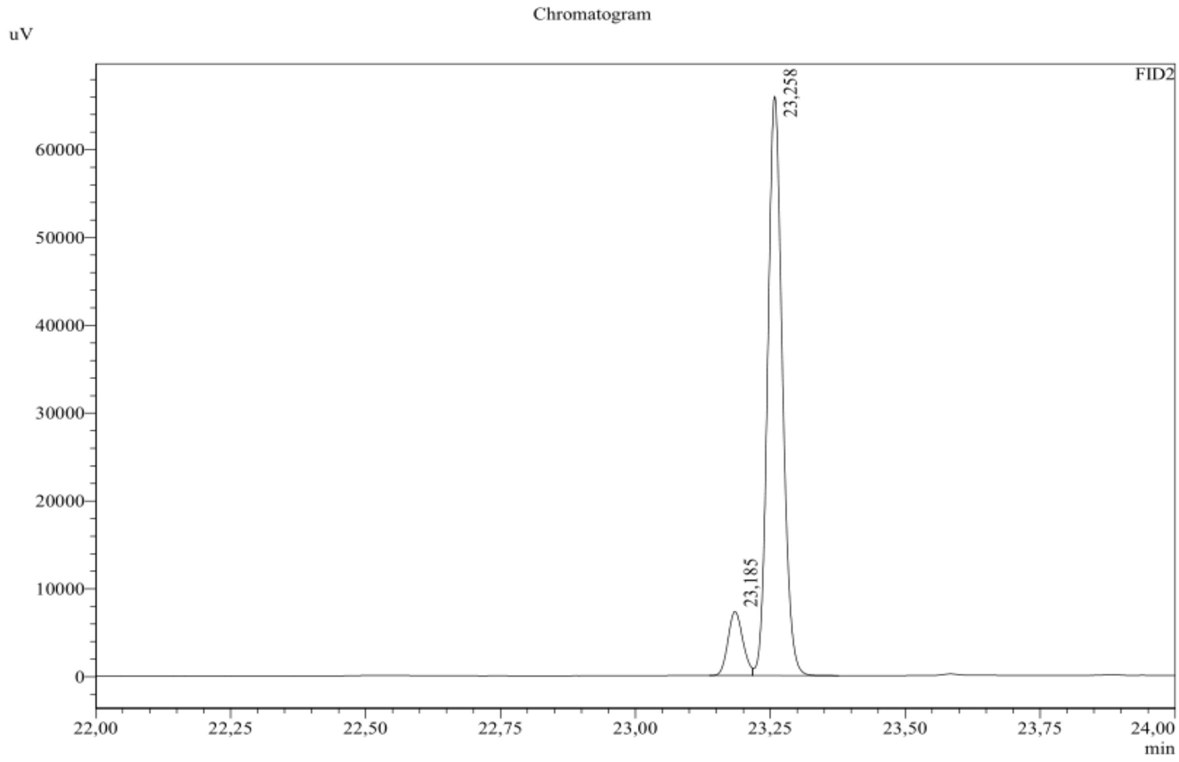

Peak Table

FID2

Peak#	Ret. Time	Area	Height	Area%	Name
1	23,185	14012	7159	10,079	
2	23,258	125010	65371	89,921	
Total		139023	72531	100,000	

1.0 h

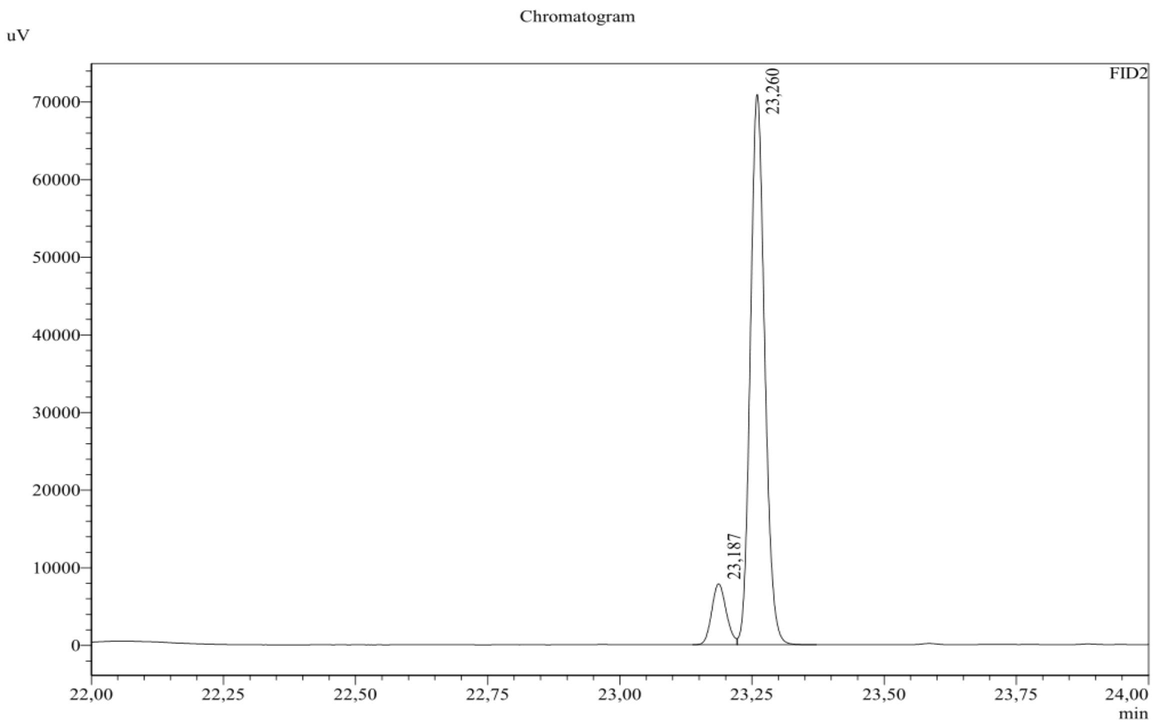

Peak Table

FID2

Peak#	Ret. Time	Area	Height	Area%	Name
1	23,187	15037	7793	10,062	
2	23,260	134408	69258	89,938	
Total		149445	77051	100,000	

1.5 h

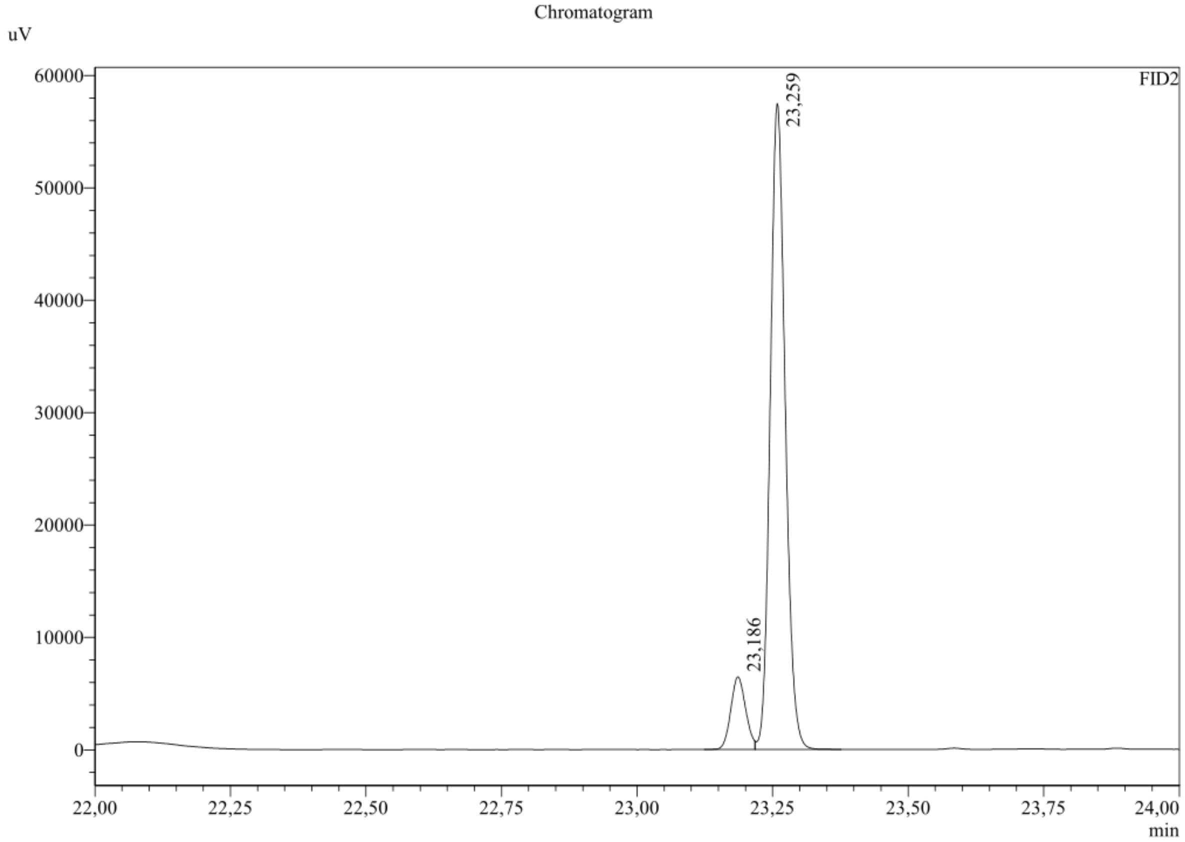

Peak Table

FID2

Peak#	Ret. Time	Area	Height	Area%	Name
1	23,186	12315	6385	10,013	
2	23,259	110680	56720	89,987	
Total		122995	63105	100,000	

2.0 h

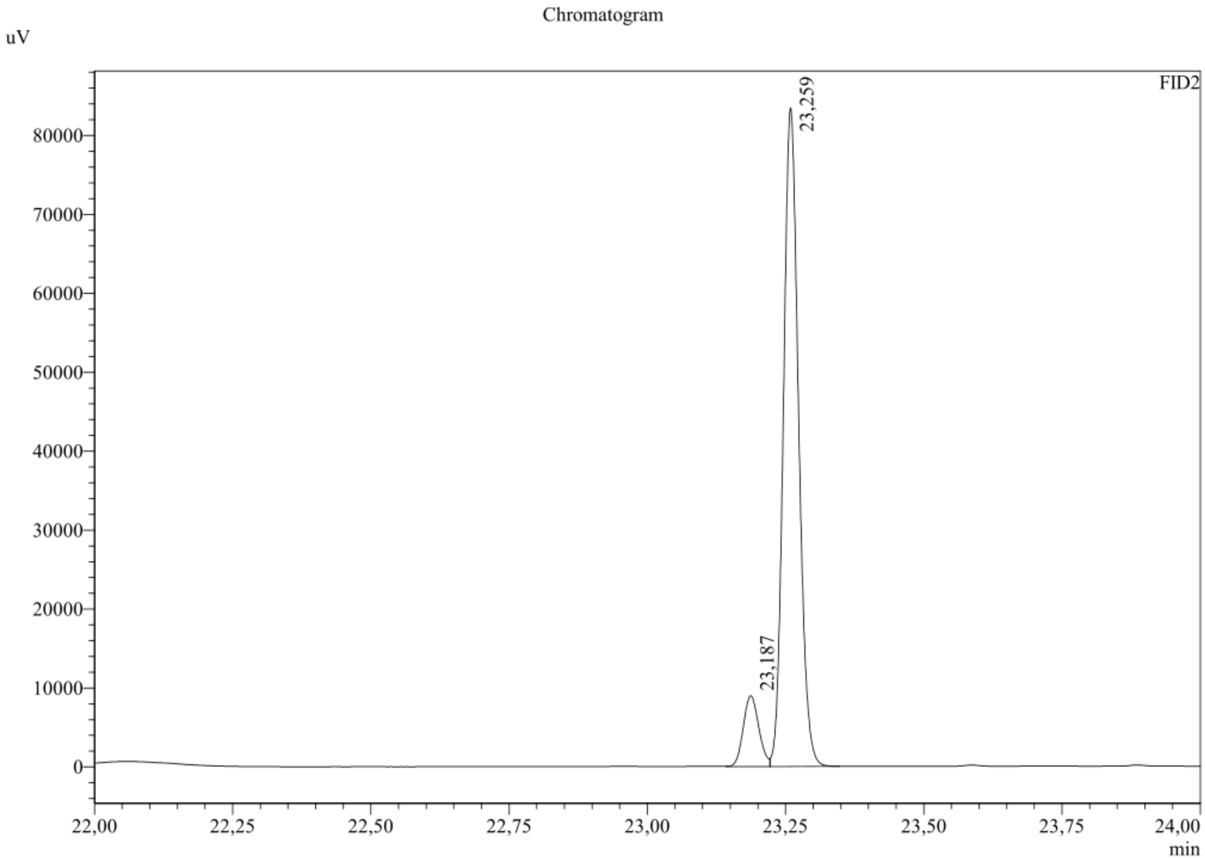

Peak Table

FID2

Peak#	Ret. Time	Area	Height	Area%	Name
1	23,187	17558	8903	9,944	
2	23,259	159015	81659	90,056	
Total		176573	90562	100,000	

2.5 h

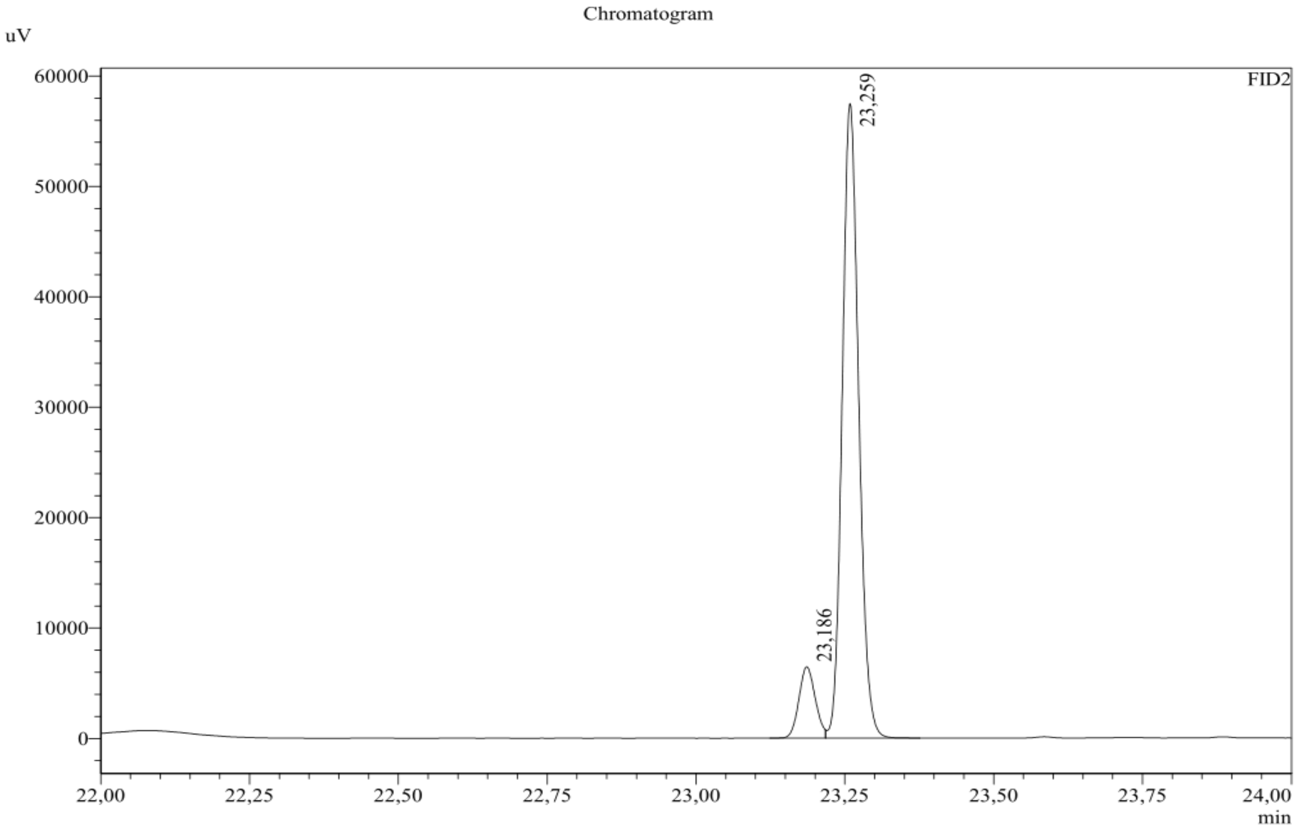

Peak Table

FID2

Peak#	Ret. Time	Area	Height	Area%	Name
1	23,186	12315	6385	10,013	
2	23,259	110680	56720	89,987	
Total		122995	63105	100,000	

Chiral HPLC spectra

HPLC *tert*-butyl-2-(4-chlorophenyl)pyrrolidine-1-carboxylate

<Chromatogram>

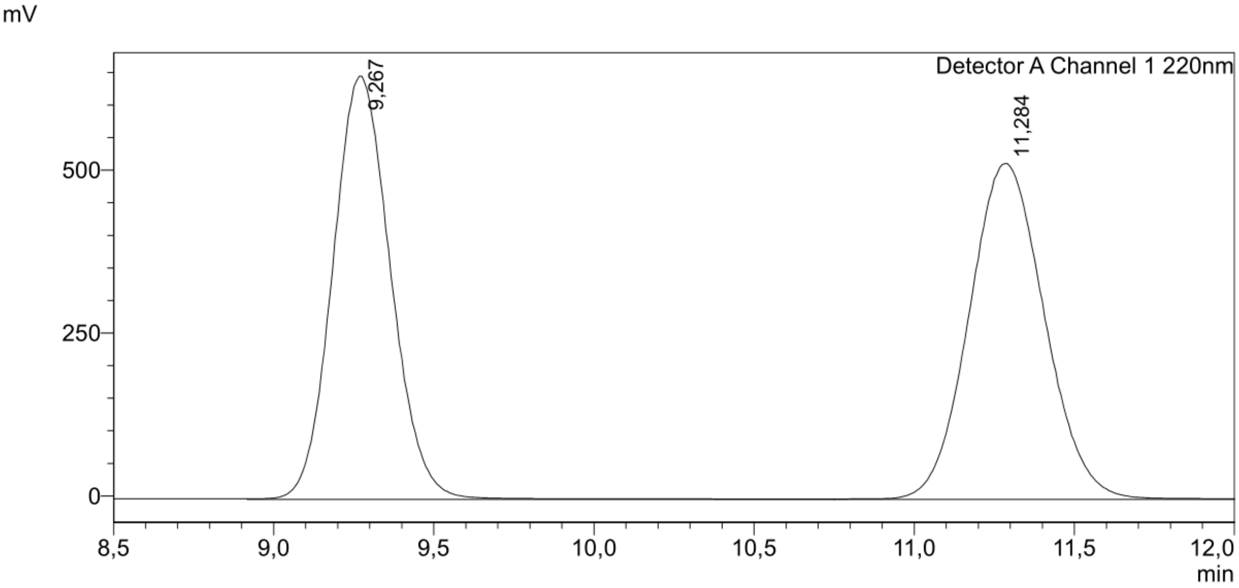

<Peak Table>

Detector A Channel 1 220nm

Peak#	Ret. Time	Area	Height	Conc.	Mark
1	9,267	8194574	649525	48,396	SV
2	11,284	8737904	515718	51,604	SV
Total		16932478	1165243		

<Chromatogram>

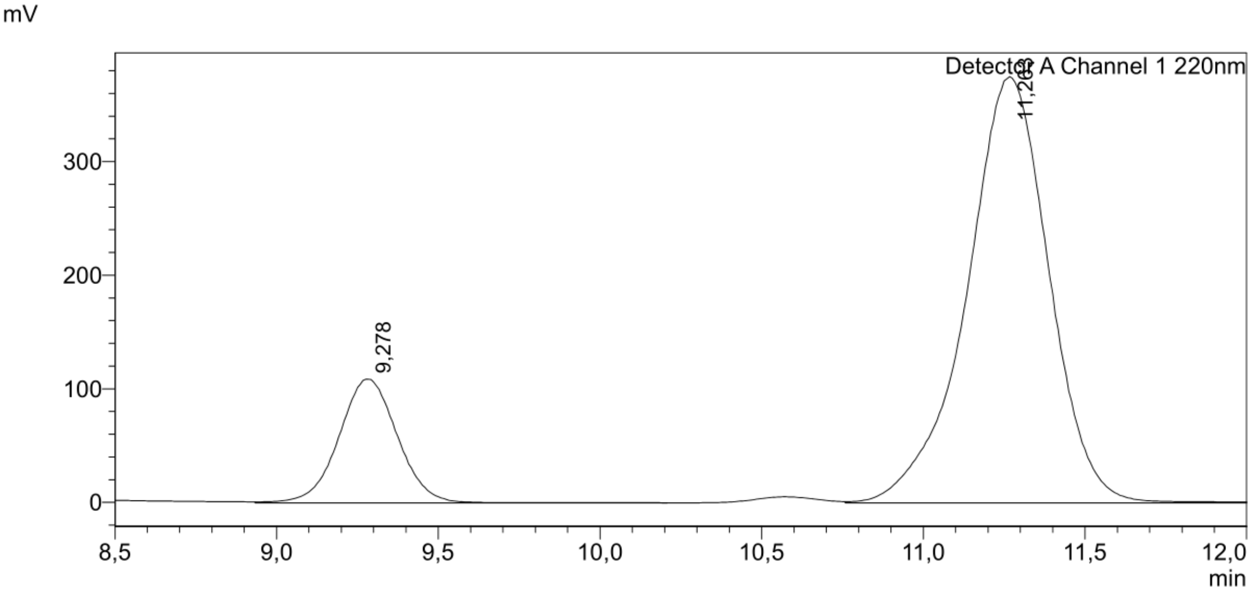

<Peak Table>

Detector A Channel 1 220nm

Peak#	Ret. Time	Area	Height	Conc.	Mark
1	9,278	1367221	108844	17,101	SV
2	11,263	6627803	375074	82,899	V
Total		7995024	483918		

HPLC *tert*-butyl-2-(4-fluorophenyl)pyrrolidine-1-carboxylate

<Chromatogram>

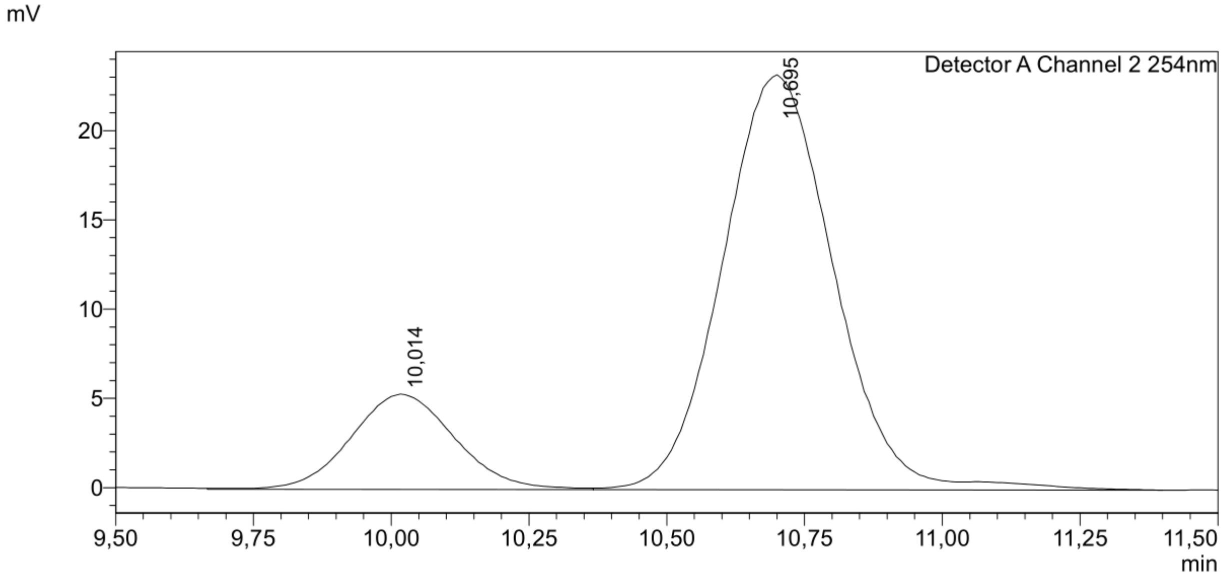

<Peak Table>

Peak Table

Detector A Channel 2 254nm

Peak#	Ret. Time	Area	Height	Conc.	Mark
1	10,014	70432	5349	17,781	
2	10,695	325666	23246	82,219	SV
Total		396098	28595		

<Chromatogram>

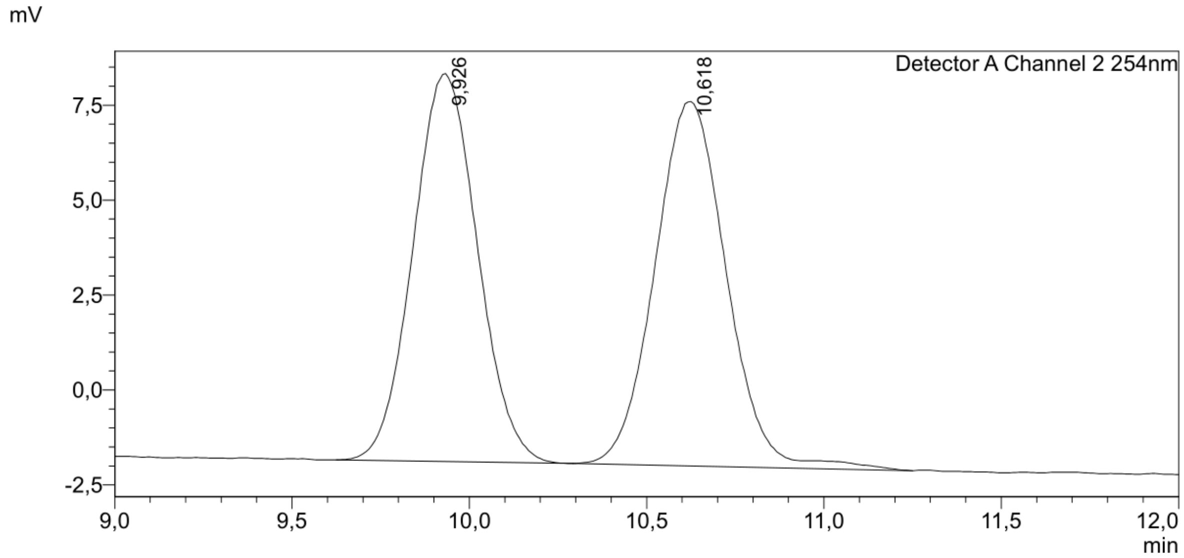

<Peak Table>

Detector A Channel 2 254nm

Peak#	Ret. Time	Area	Height	Conc.	Unit	Mark	Name
1	9,926	129349	10212	49,069			
2	10,618	134259	9597	50,931		S	
Total		263608	19809				

Racemic *tert*-butyl-2-(4-fluorophenyl)pyrrolidine-1-carboxylate was synthesized using Co-porphyrin-catalyzed intramolecular C-H bond amination as described by de Bruin and co-workers.[S4]

HPLC *tert*-butyl-2-(2,5-difluorophenyl)pyrrolidine-1-carboxylate

<Chromatogram>

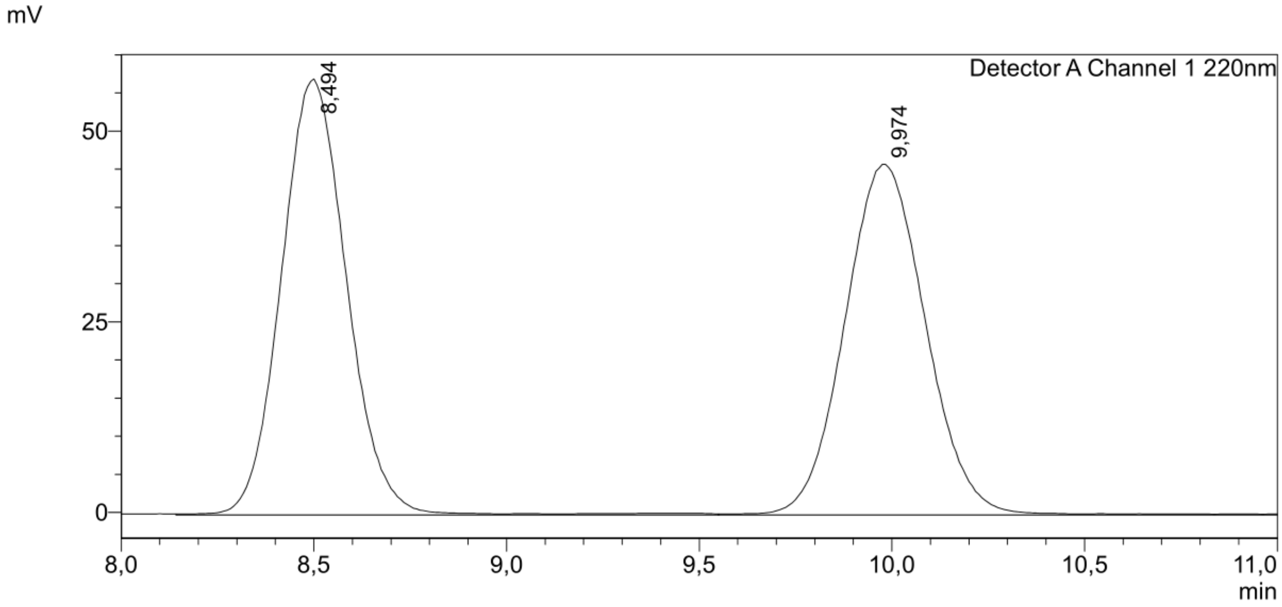

<Peak Table>

Detector A Channel 1 220nm

Peak#	Ret. Time	Area	Height	Conc.	Mark
1	8,494	676746	57159	49,703	SV
2	9,974	684840	45983	50,297	SV
Total		1361587	103141		

<Chromatogram>

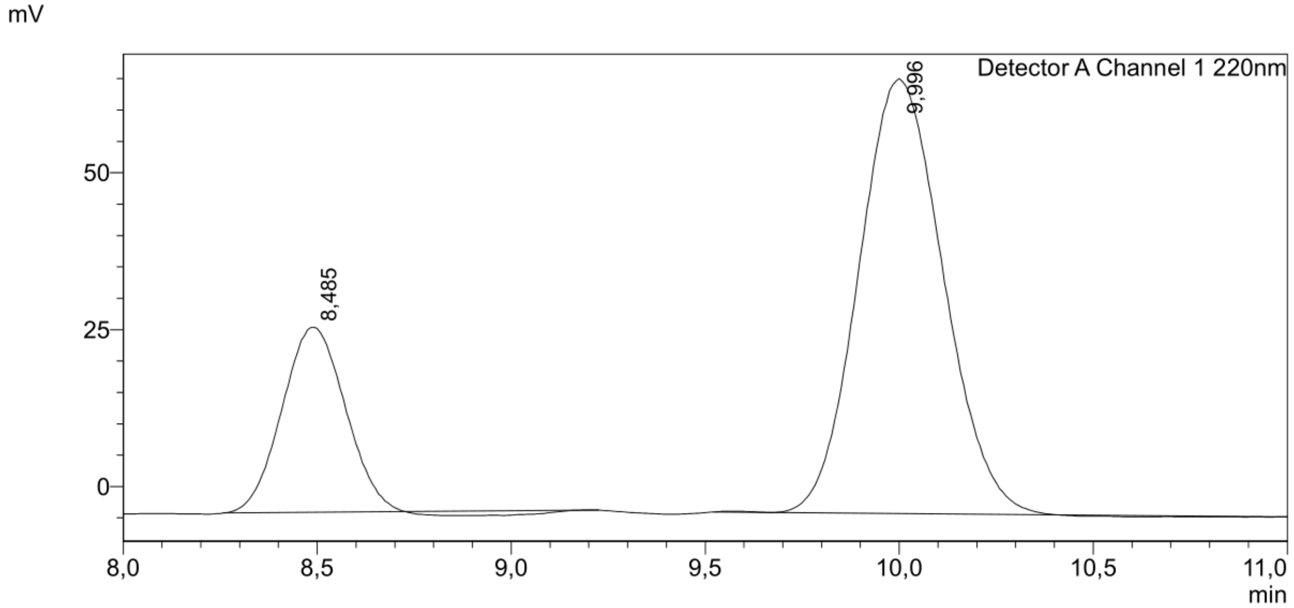

<Peak Table>

Detector A Channel 1 220nm

Peak#	Ret. Time	Area	Height	Conc.	Mark
1	8,485	322337	29485	23,595	M
2	9,996	1043778	69298	76,405	M
Total		1366114	98783		

HPLC *tert*-butyl-2-(3,4-dimethoxyphenyl)pyrrolidine-1-carboxylate

<Chromatogram>

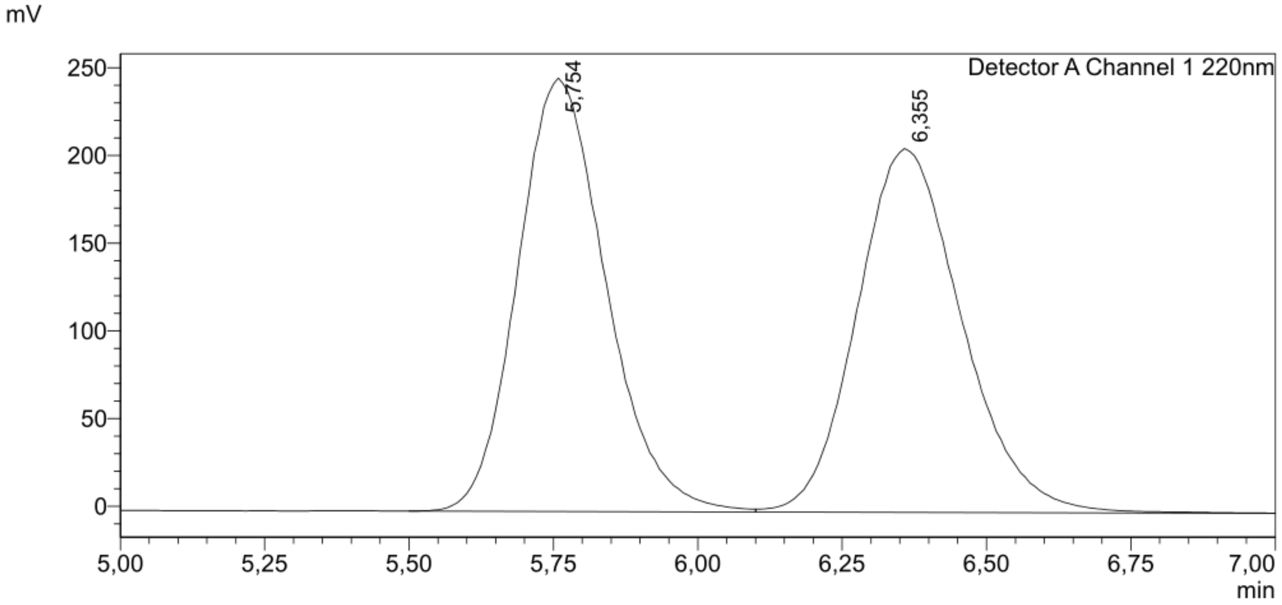

<Peak Table>

Detector A Channel 1 220nm

Peak#	Ret. Time	Area	Height	Conc.	Mark
1	5,754	2628026	247102	50,062	
2	6,355	2621503	207573	49,938	V
Total		5249529	454675		

<Chromatogram>

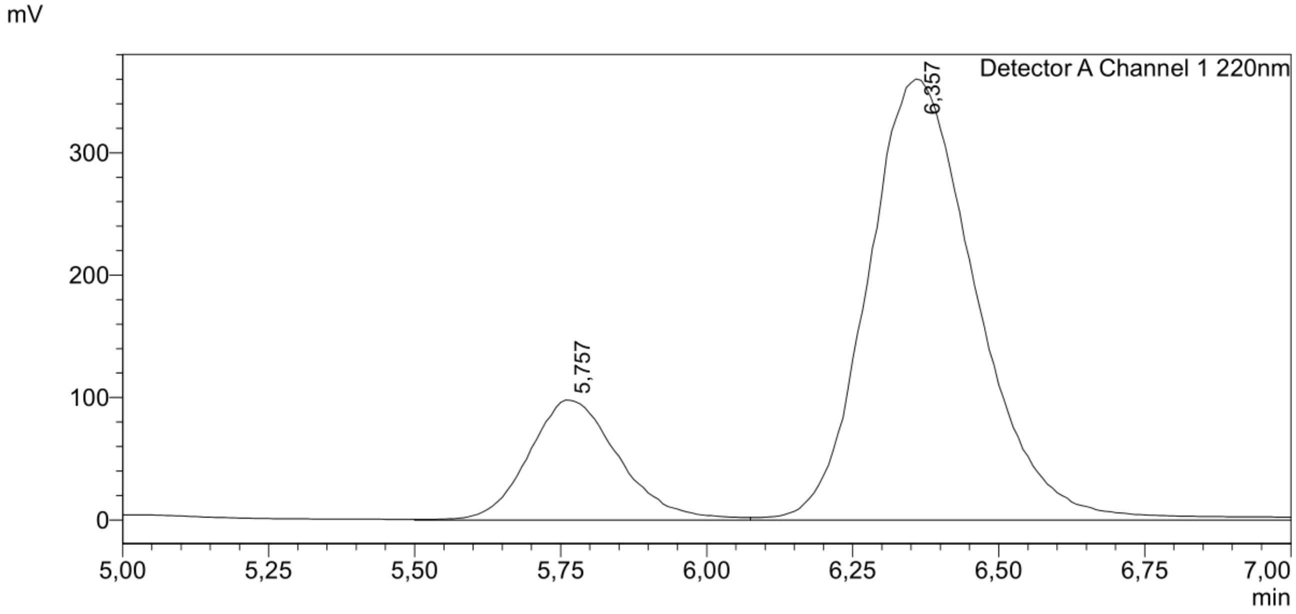

<Peak Table>

Detector A Channel 1 220nm

Peak#	Ret. Time	Area	Height	Conc.	Mark
1	5,757	1056086	98066	18,328	V
2	6,357	4706148	360338	81,672	SV
Total		5762234	458404		

HPLC *tert*-butyl-2-(pyridin-3-yl)pyrrolidine-1-carboxylate

<Chromatogram>

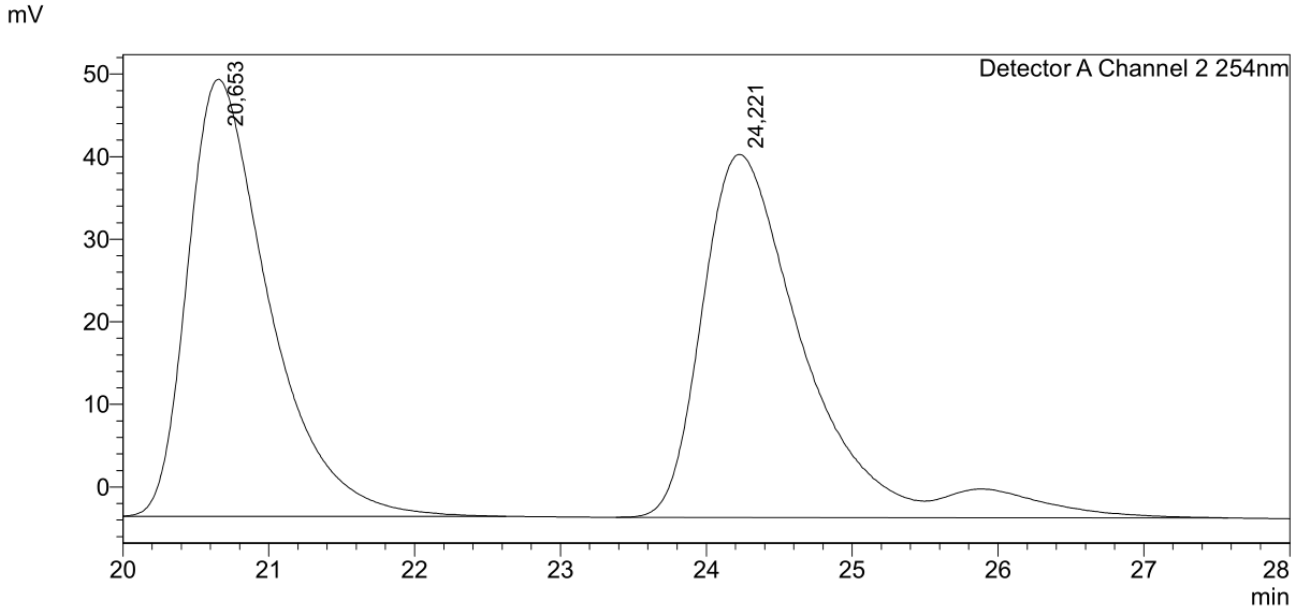

<Peak Table>

Detector A Channel 2 254nm

Peak#	Ret. Time	Area	Height	Conc.	Mark
1	20,653	2052718	52922	48,858	SV
2	24,221	2148688	43973	51,142	SV
Total		4201407	96895		

<Chromatogram>

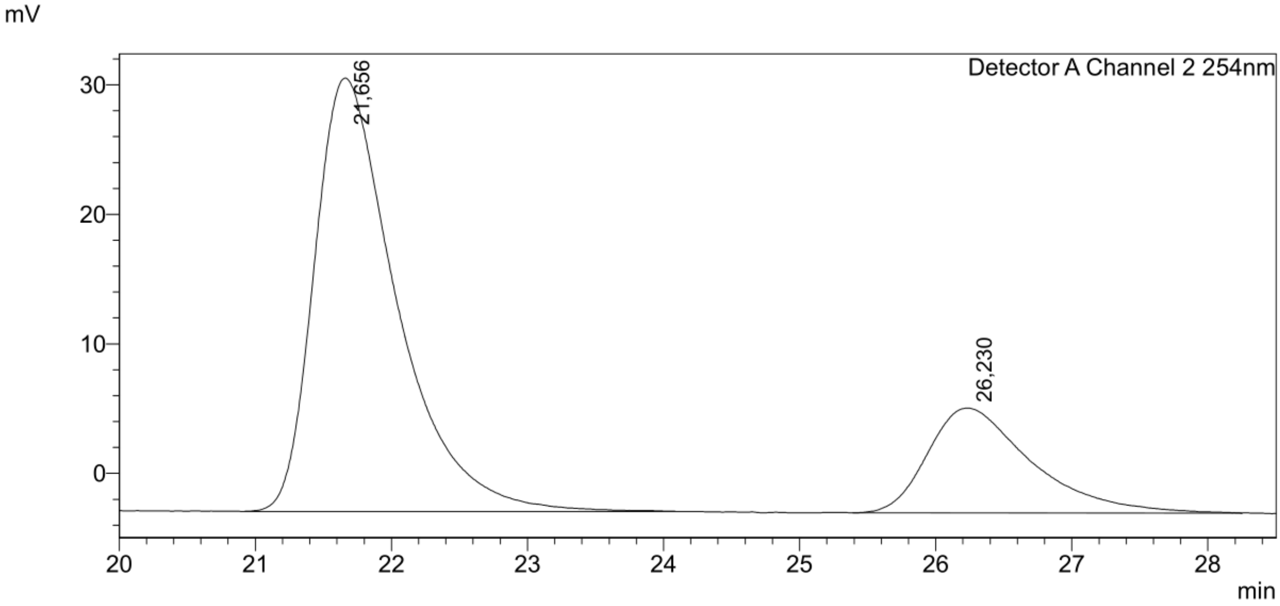

<Peak Table>

Detector A Channel 2 254nm

Peak#	Ret. Time	Area	Height	Conc.	Mark
1	21,656	1410524	33454	76,650	S
2	26,230	429699	8104	23,350	SV
Total		1840223	41558		

NMR Spectra

(S)-2-(2-bromophenyl)-5,5-diethyl-4-phenyl-4,5-dihydrooxazole

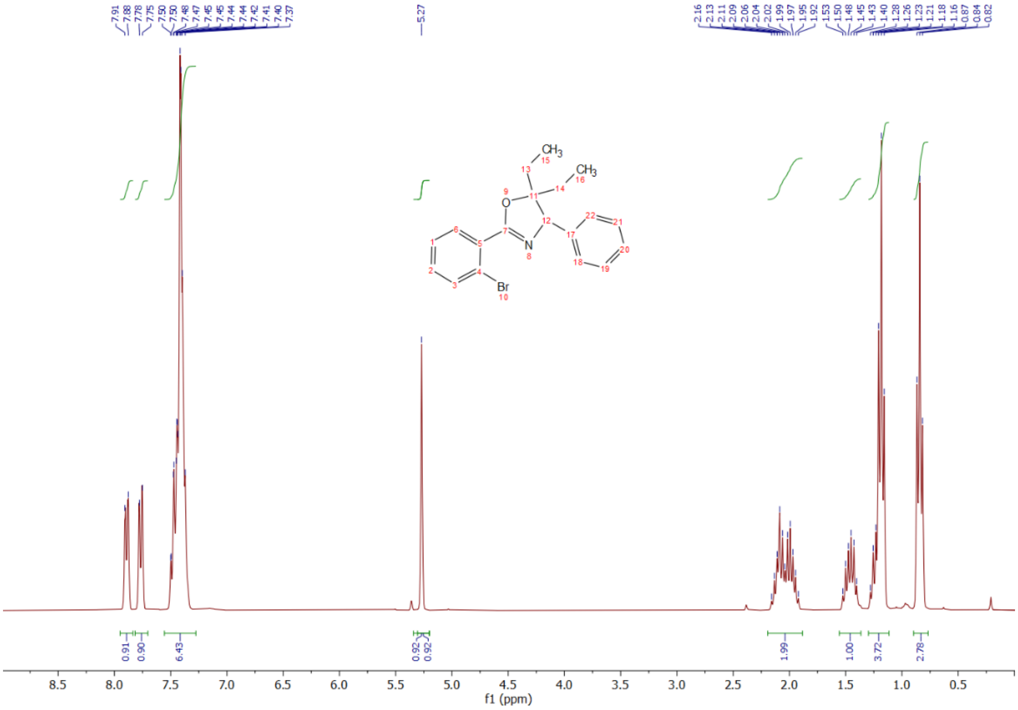

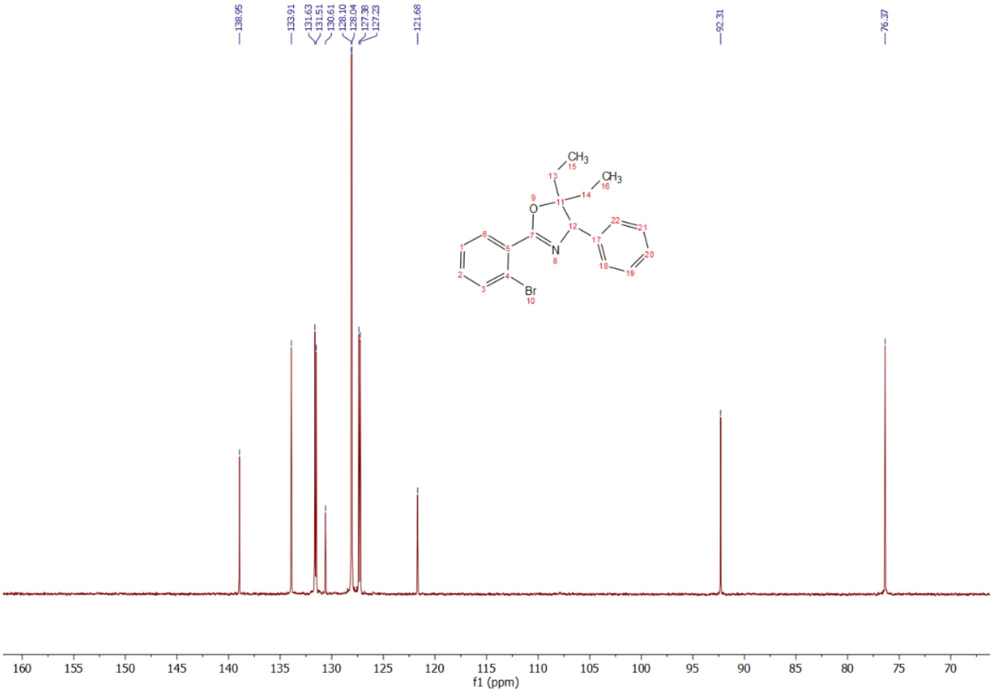

(S)-2-(2-bromophenyl)-5,5-dipropyl-4-phenyl-4,5-dihydrooxazole

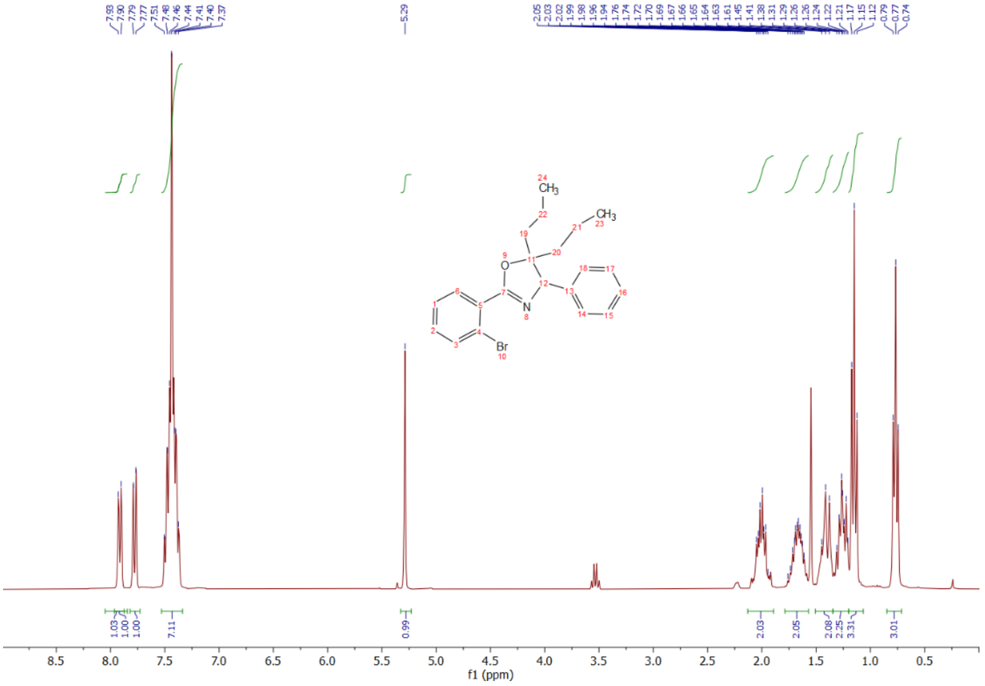

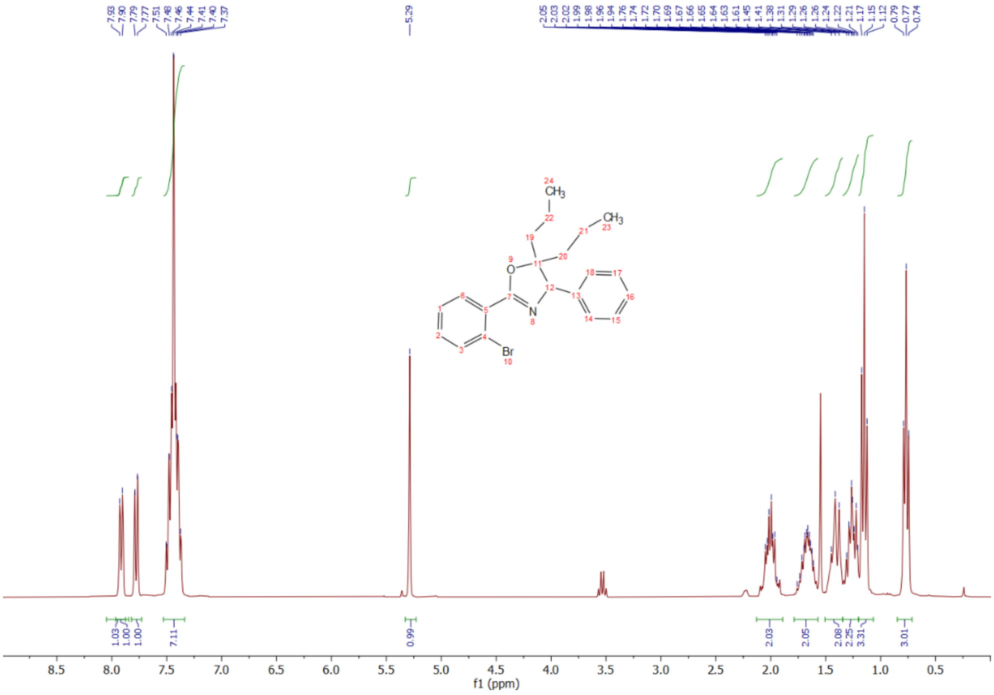

(S)-2-(2-bromophenyl)-5,5-dibutyl-4-phenyl-4,5-dihydrooxazole

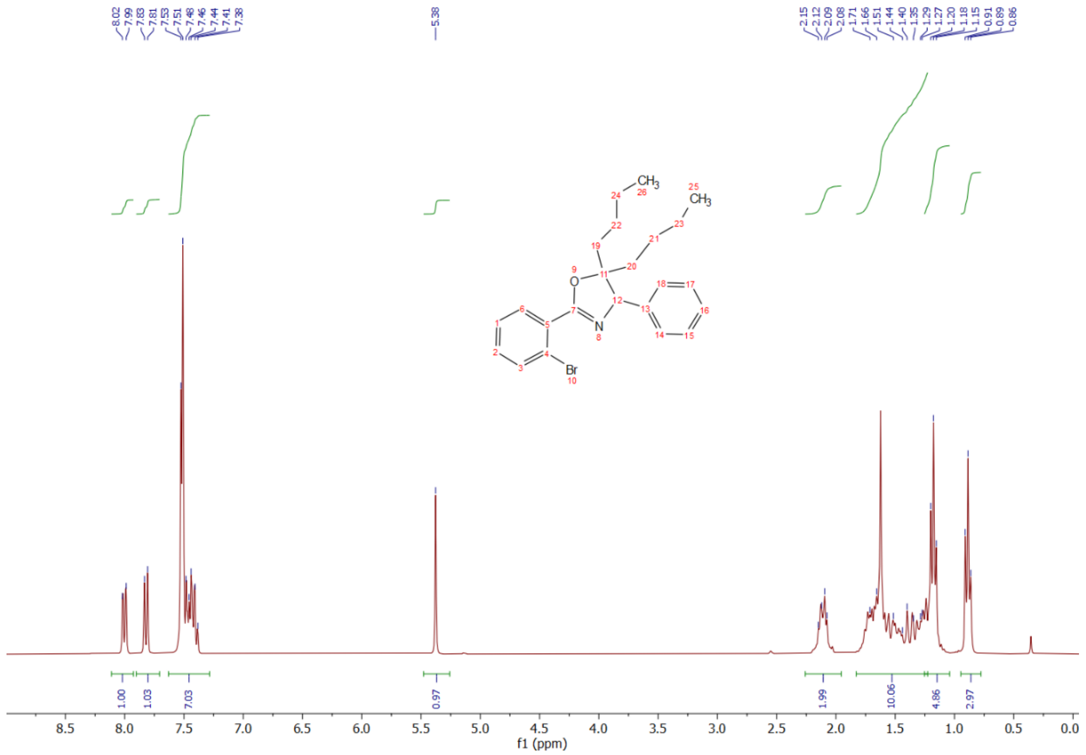

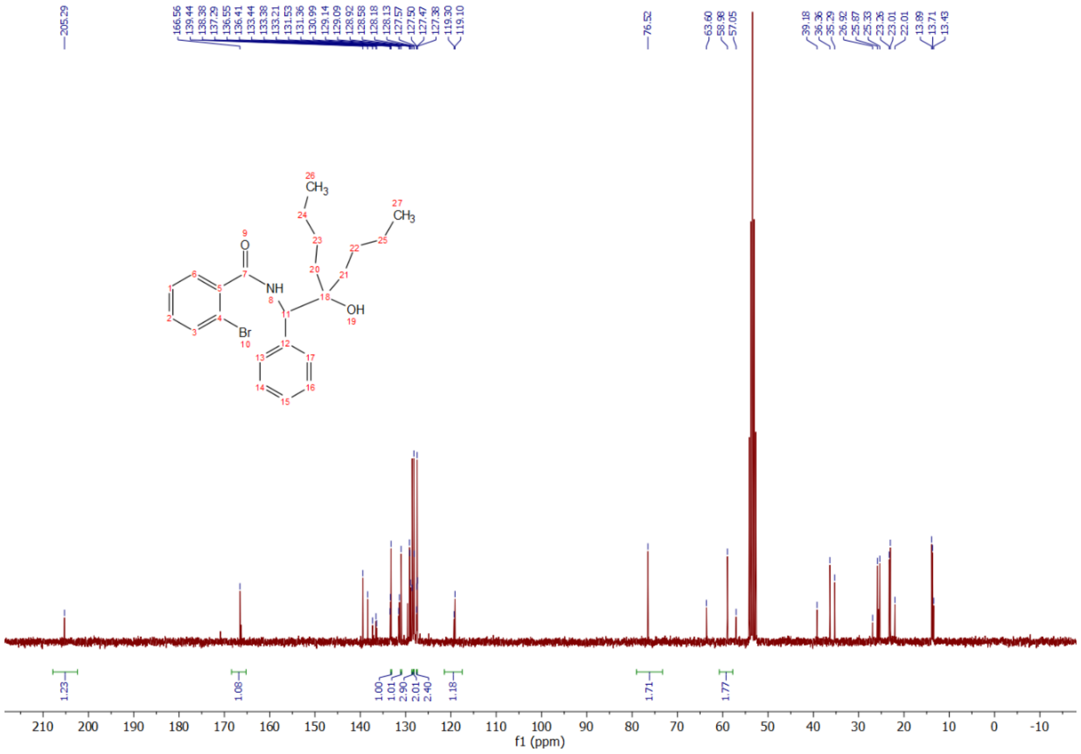

(S)-2-(2-bromophenyl)-5,5-dihexyl-4-phenyl-4,5-dihydrooxazole

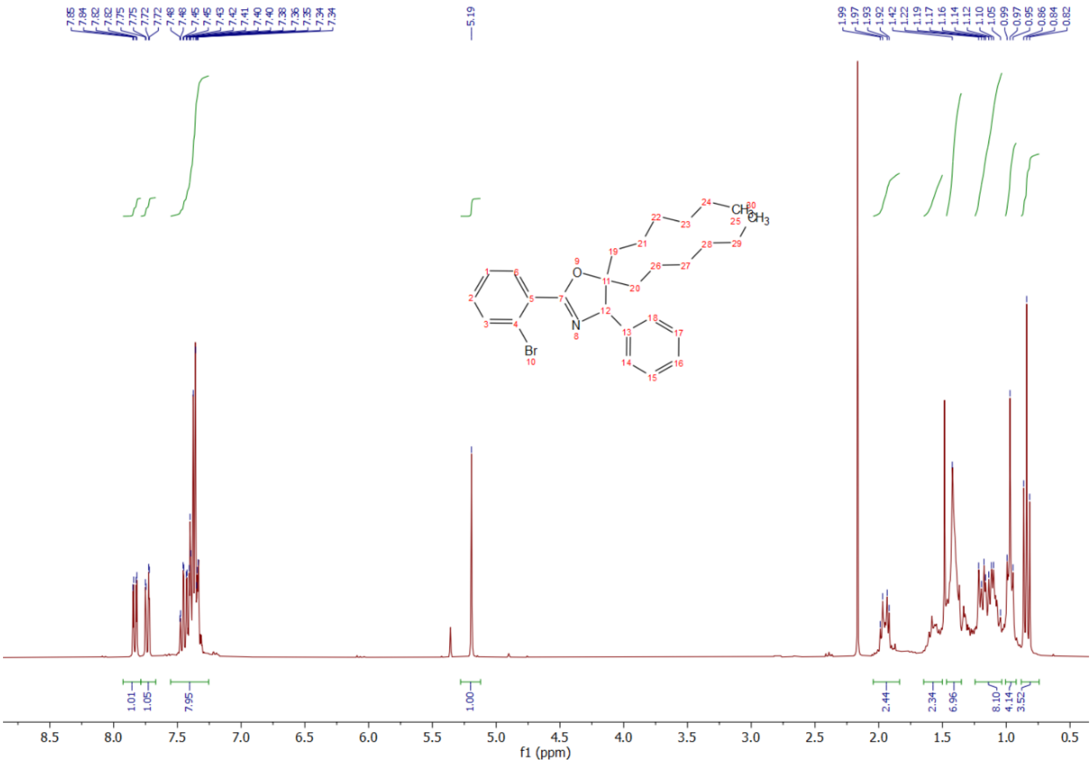

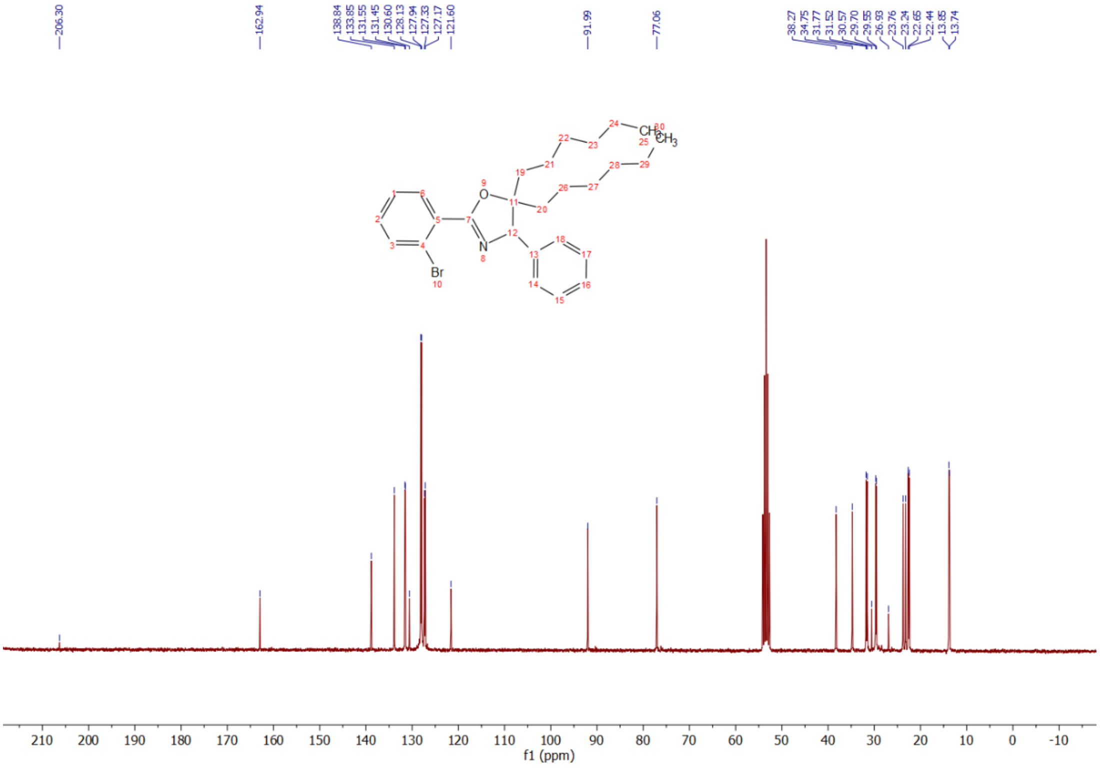

BH-Ligands

H/H Lig

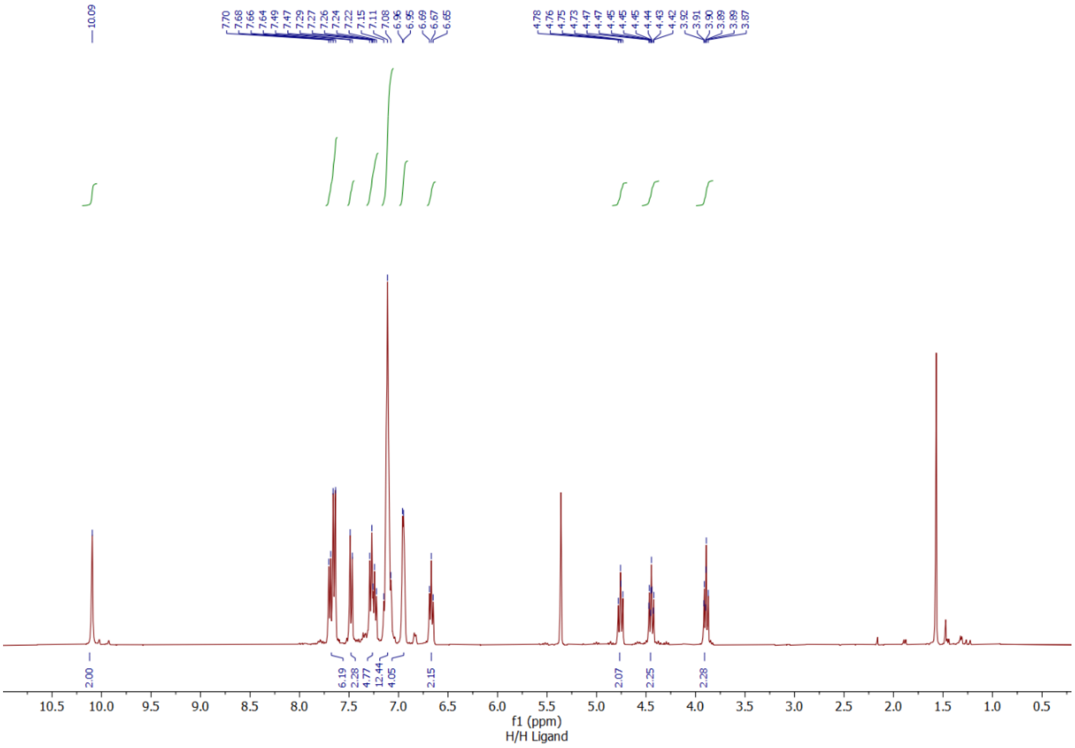

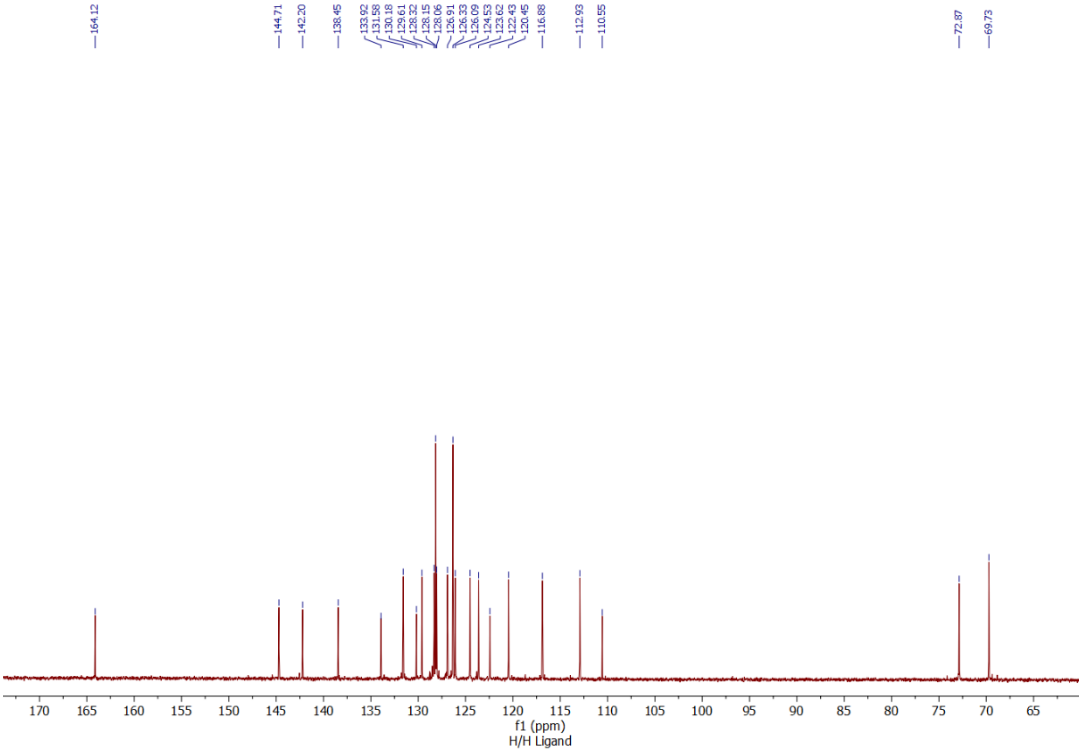

RRR-Me/Me Lig

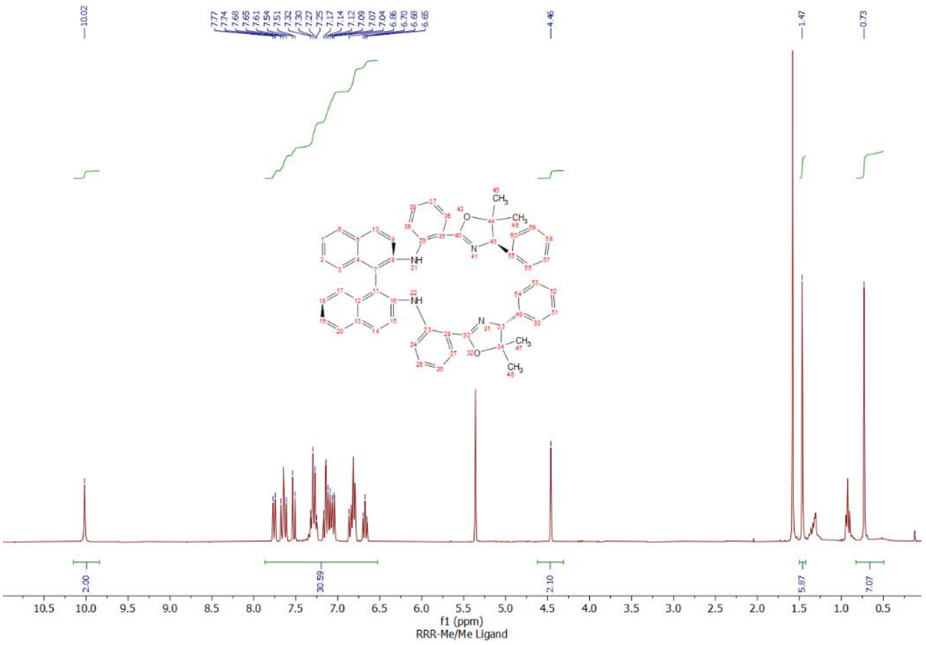

10.02
7.77
7.74
7.68
7.65
7.61
7.54
7.51
7.32
7.30
7.27
7.25
7.17
7.14
7.12
7.09
7.07
7.04
6.86
6.70
6.68
6.65
4.46
1.47
0.73
2.00
30.59
2.10
5.87
7.07
f1 (ppm)
RRR-Me/Me Ligand

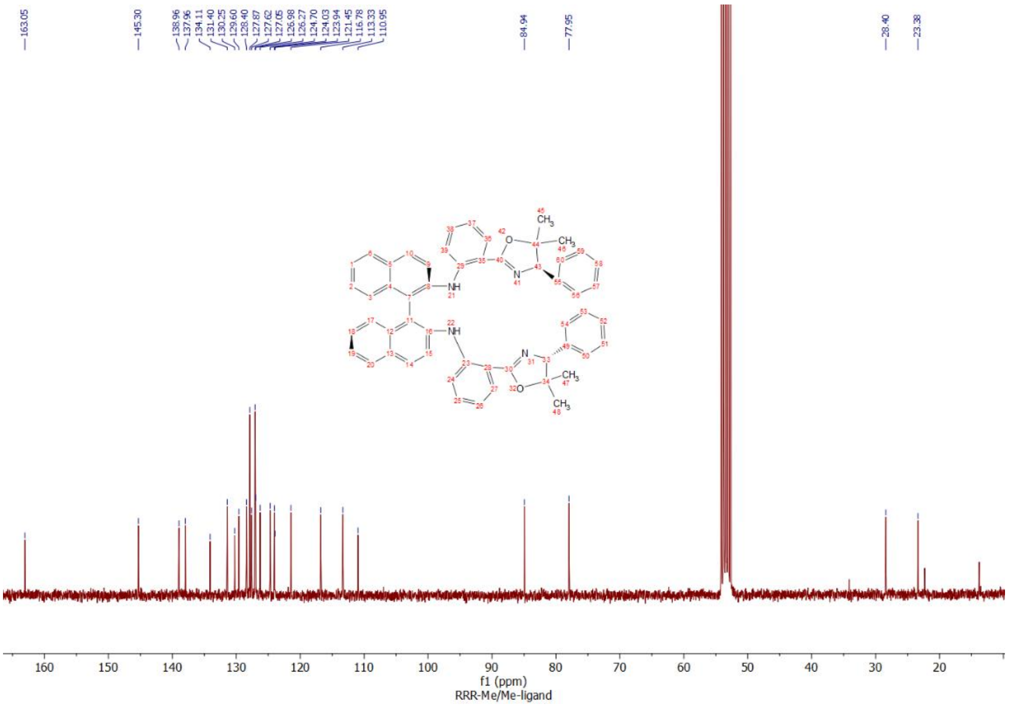

163.05
145.30
138.96
137.96
134.11
131.40
130.25
129.60
128.40
127.87
127.62
127.05
126.98
126.27
124.70
124.03
123.94
121.45
116.78
113.33
110.95
84.94
77.95
28.40
23.38
f1 (ppm)
RRR-Me/Me-ligand

RRS-Me/Me Lig

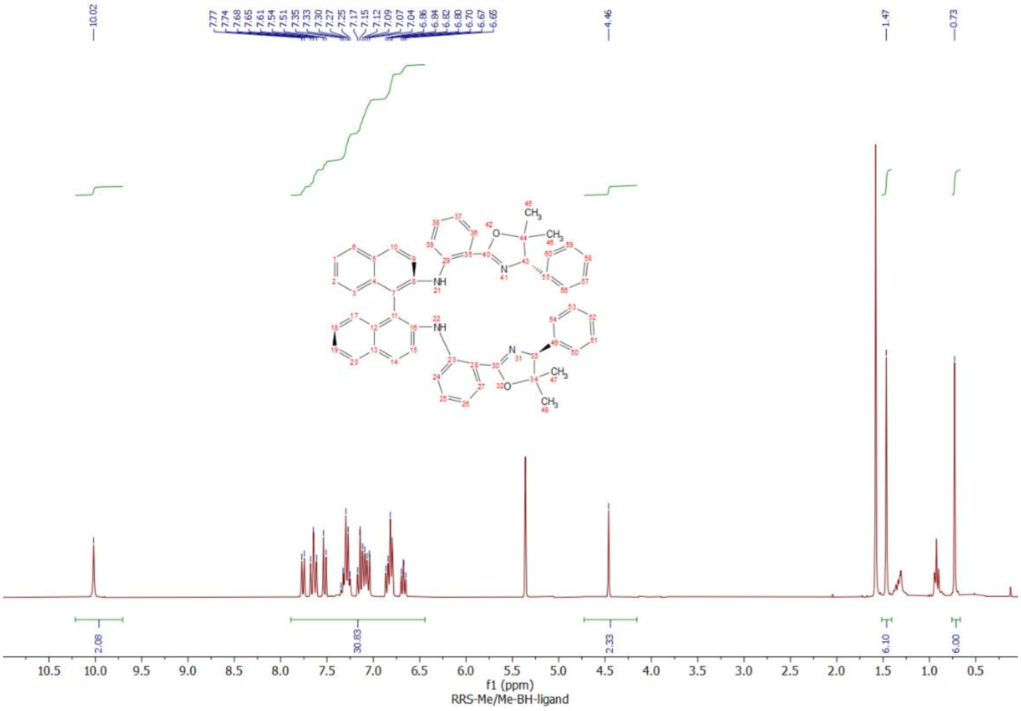
10.02
7.77 7.74 7.68 7.65 7.61 7.54 7.51 7.35 7.33 7.30 7.27 7.25 7.17 7.15 7.12 7.09 7.07 7.04 6.86 6.84 6.82 6.80 6.70 6.67 6.65
4.46
1.47
0.73
2.08
30.83
2.33
6.10
6.00
f1 (ppm)
RRS-Me/Me-BH-ligand

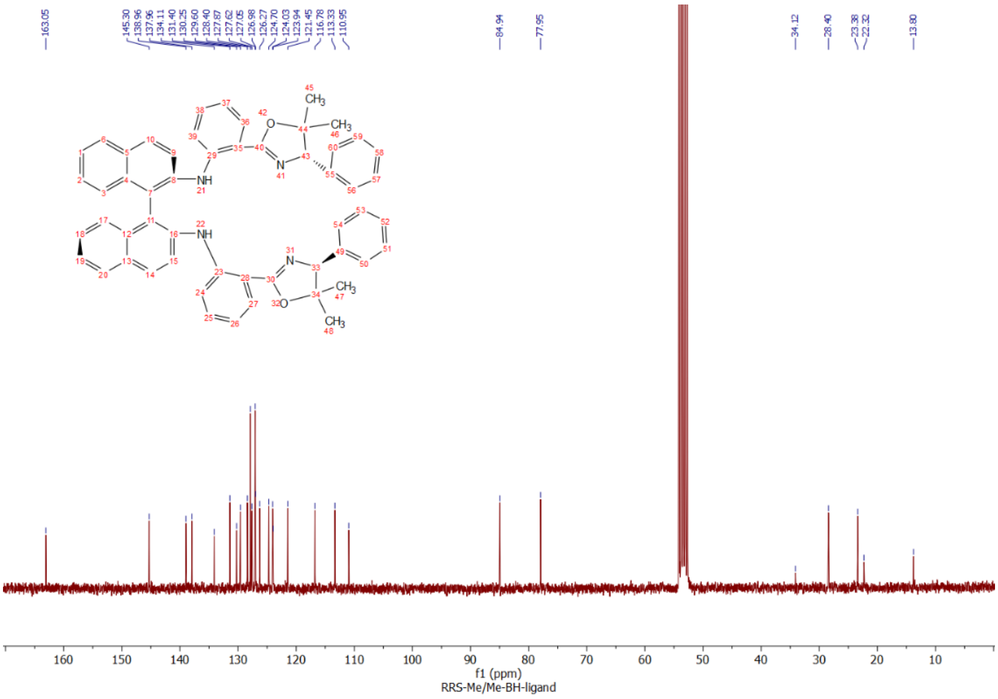
163.05
145.30 138.96 137.96 134.11 131.40 130.25 129.60 128.40 127.87 127.62 127.05 126.98 126.27 124.70 124.03 123.94 121.45 116.78 113.33 110.95
84.94
77.95
34.12
28.40
23.38
22.32
13.80
f1 (ppm)
RRS-Me/Me-BH-ligand

SSS-Me/Me Lig

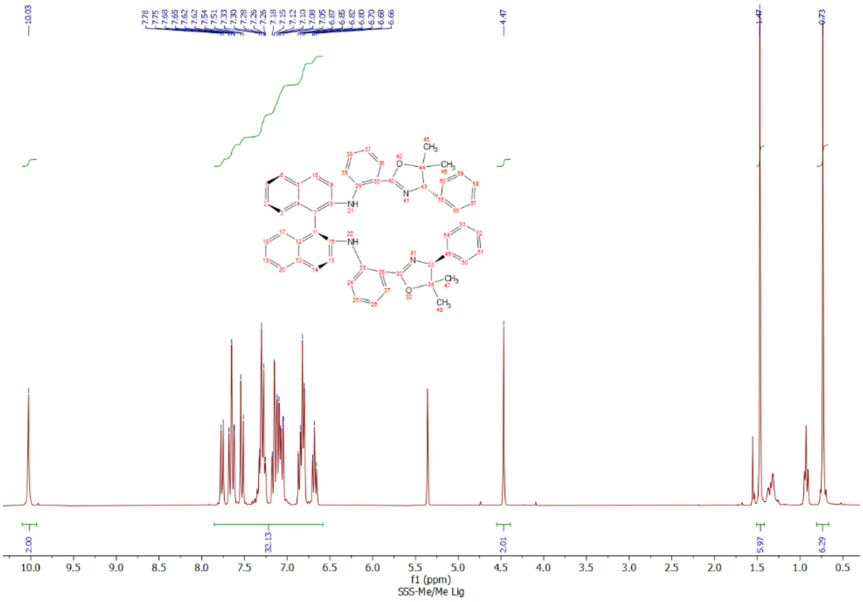
10.03
7.78 7.75 7.68 7.65 7.62 7.62 7.54 7.51 7.33 7.30 7.28 7.26 7.26 7.18 7.15 7.12 7.10 7.08 7.05 6.87 6.85 6.82 6.80 6.70 6.68 6.66
4.47
1.47
0.73
2.00
32.13
2.01
5.97
6.29
f1 (ppm)
SSS-Me/Me Lig

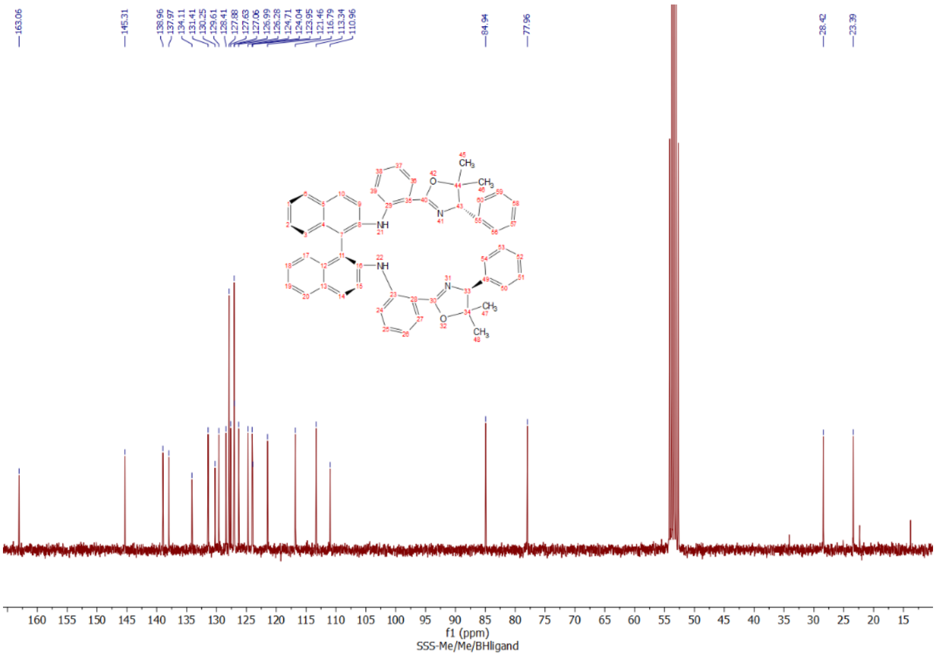
163.06
145.31
138.96 137.97 134.11 131.41 130.25 129.61 128.41 127.88 127.63 127.06 126.99 126.28 124.71 124.04 123.95 121.46 116.79 113.34 110.96
84.94
77.96
28.42
23.39
f1 (ppm)
SSS-Me/Me/BHligand

SRR-Me/Me Lig

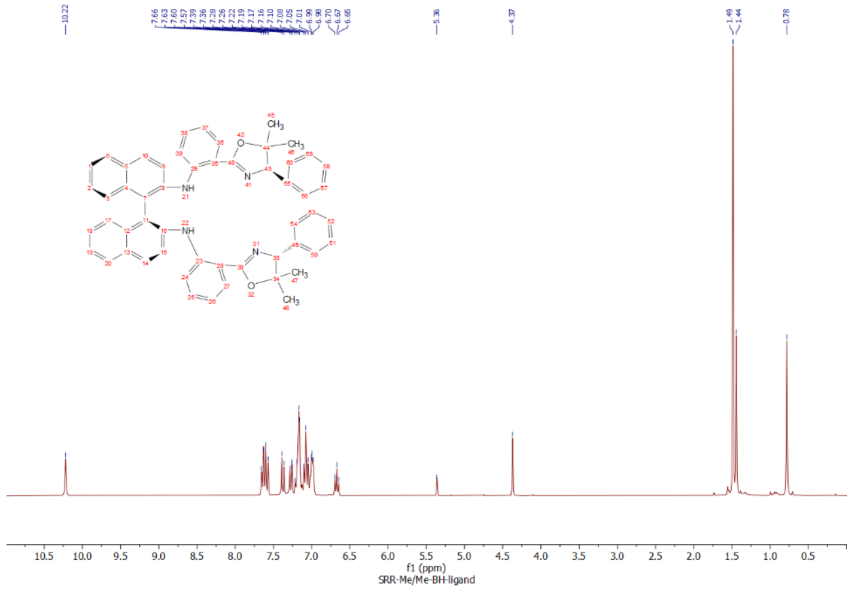

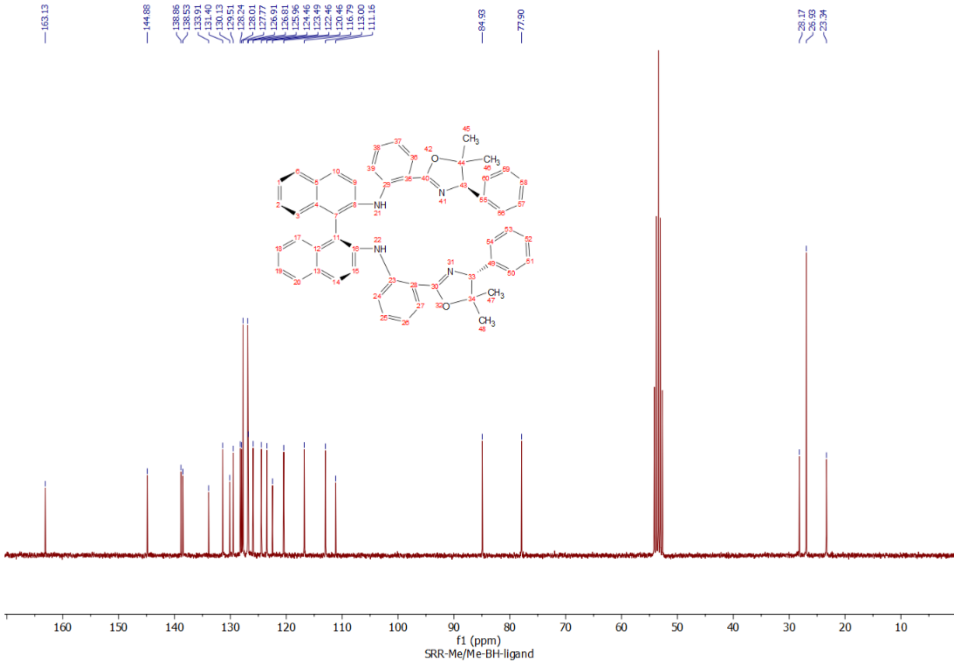

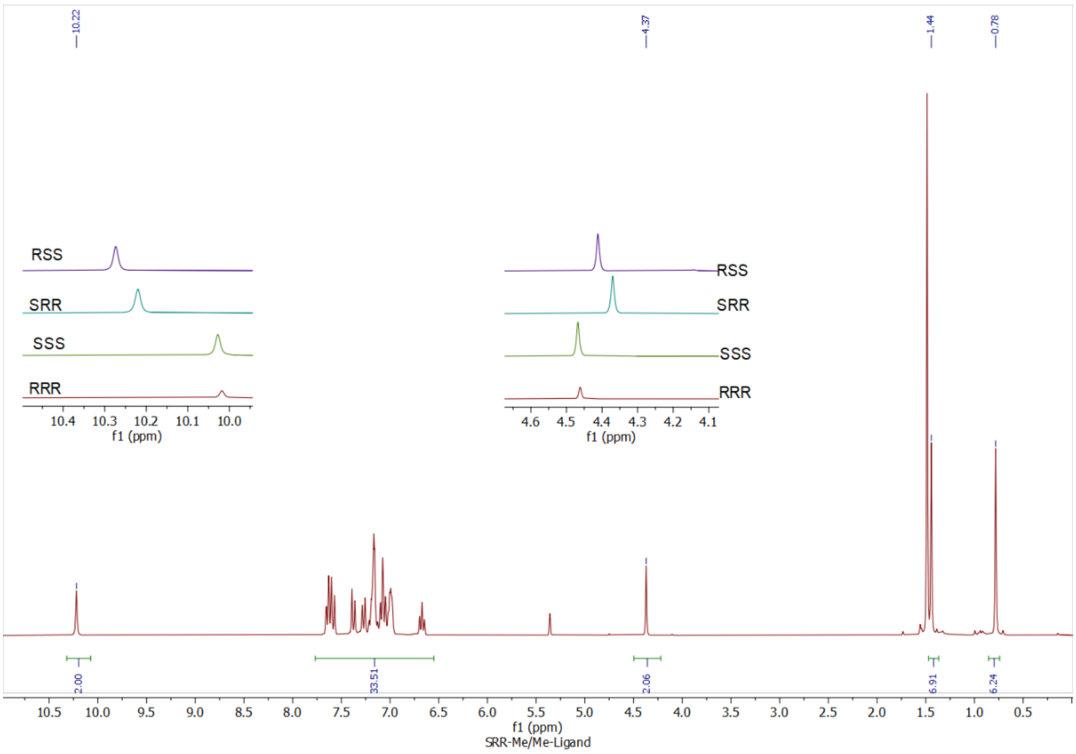

10.22
4.37
1.44
0.78
RSS
SRR
SSS
RRR
10.4 10.3 10.2 10.1 10.0
f1 (ppm)
4.6 4.5 4.4 4.3 4.2 4.1
f1 (ppm)
2.00
33.51
2.06
6.91
6.24
10.5 10.0 9.5 9.0 8.5 8.0 7.5 7.0 6.5 6.0 5.5 5.0 4.5 4.0 3.5 3.0 2.5 2.0 1.5 1.0 0.5
f1 (ppm)
SRR-Me/Me-Ligand

Et/Et Ligand

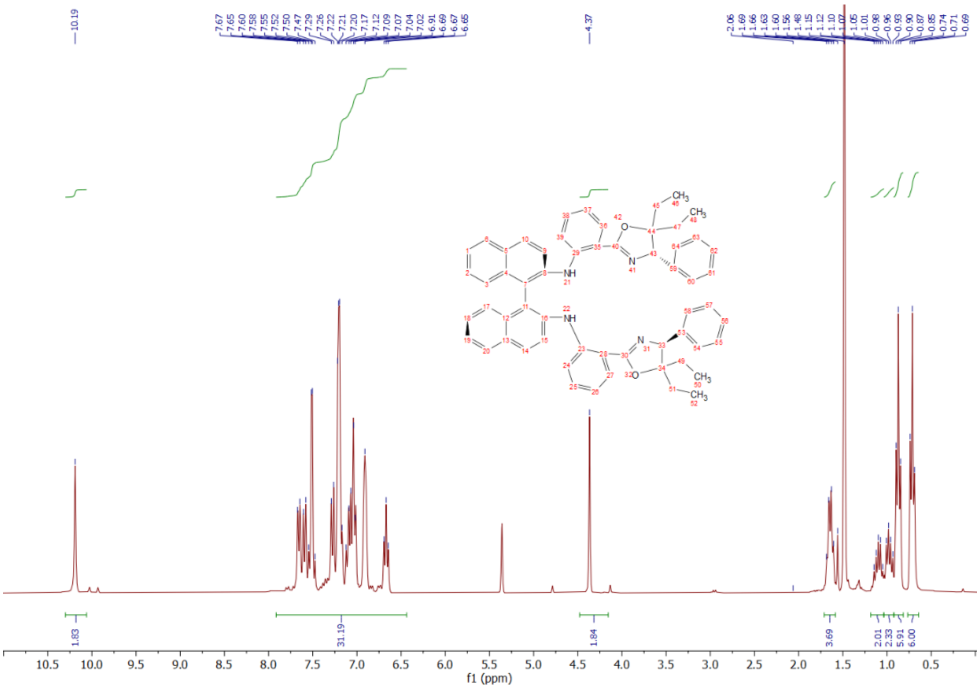
10.19
7.67 7.65 7.60 7.58 7.55 7.52 7.50 7.47 7.29 7.26 7.22 7.21 7.20 7.17 7.12 7.09 7.07 7.04 7.02 6.91 6.69 6.67 6.65
4.37
2.06 1.69 1.66 1.63 1.60 1.56 1.48 1.15 1.12 1.10 1.07 1.05 1.01 0.98 0.96 0.93 0.90 0.87 0.85 0.74 0.71 0.69
1.83
31.19
1.84
3.69
2.01 2.33 5.91 6.00
10.5 10.0 9.5 9.0 8.5 8.0 7.5 7.0 6.5 6.0 5.5 5.0 4.5 4.0 3.5 3.0 2.5 2.0 1.5 1.0 0.5
f1 (ppm)

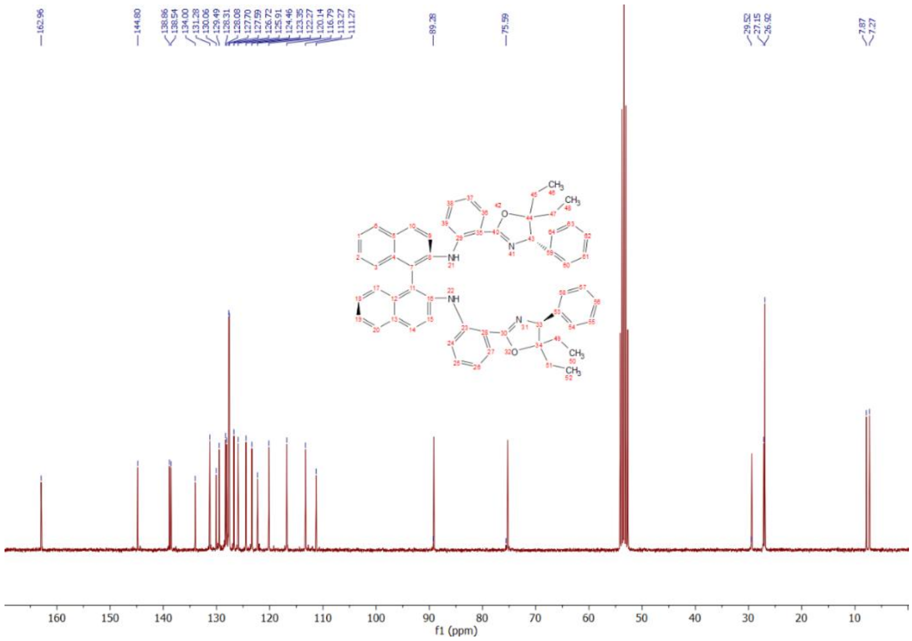
162.96
144.80
138.86 138.54 134.00 131.28 130.06 129.49 128.31 128.08 127.70 127.59 126.72 125.91 124.46 123.35 122.27 120.14 116.79 113.27 111.27
89.28
75.59
29.52 27.15 26.92
7.87 7.27
160 150 140 130 120 110 100 90 80 70 60 50 40 30 20 10
f1 (ppm)

Prop/Prop Ligand

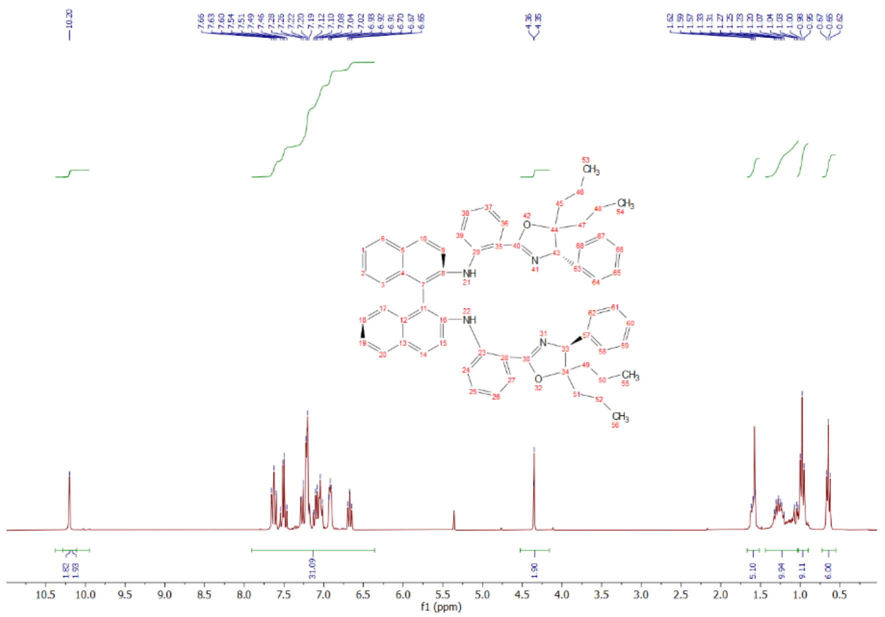

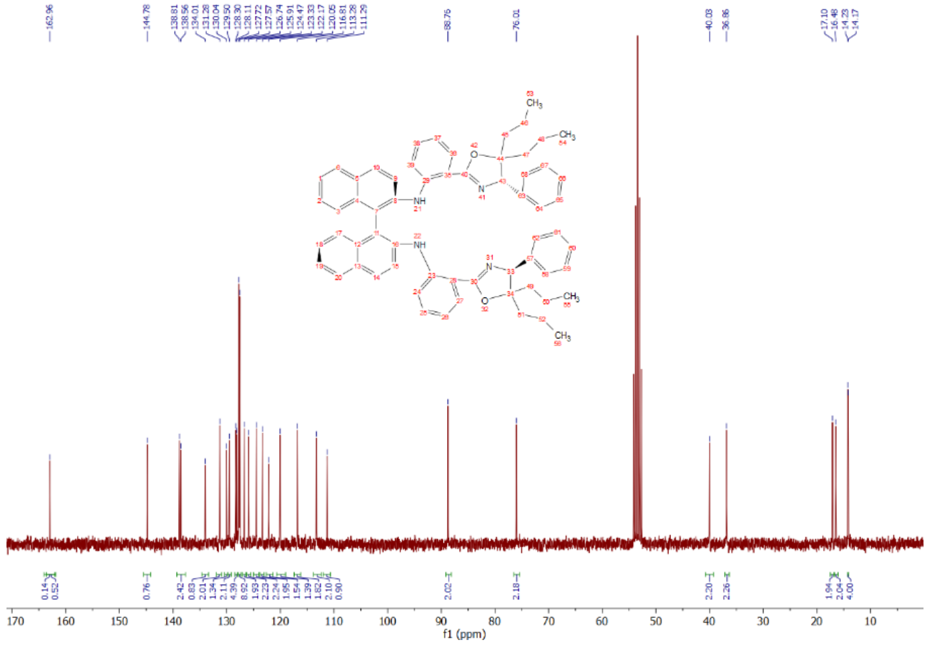

But/But Ligand

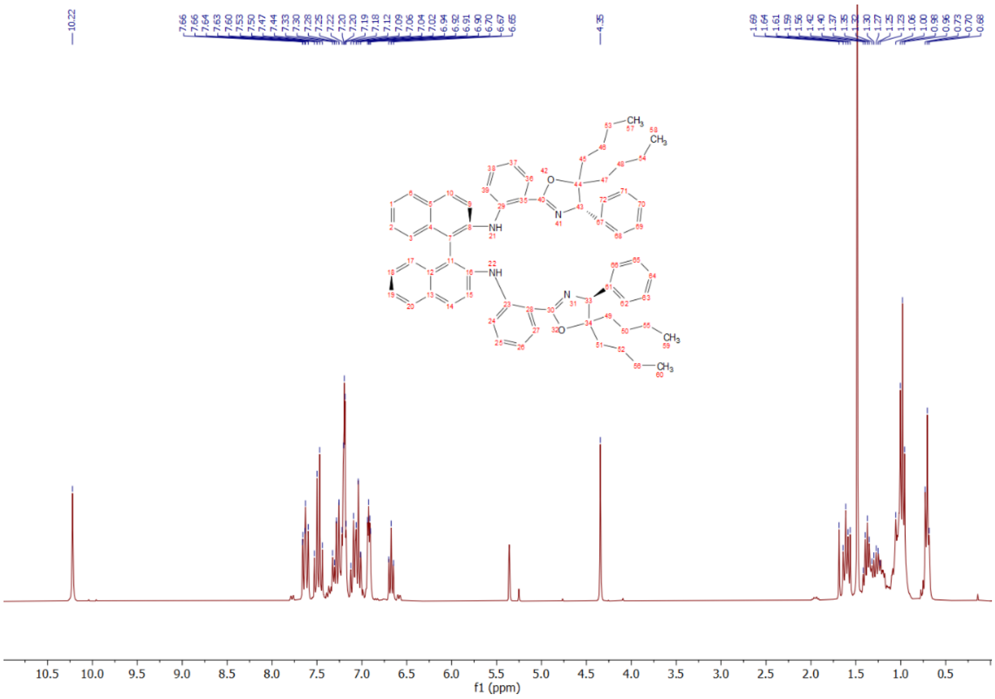

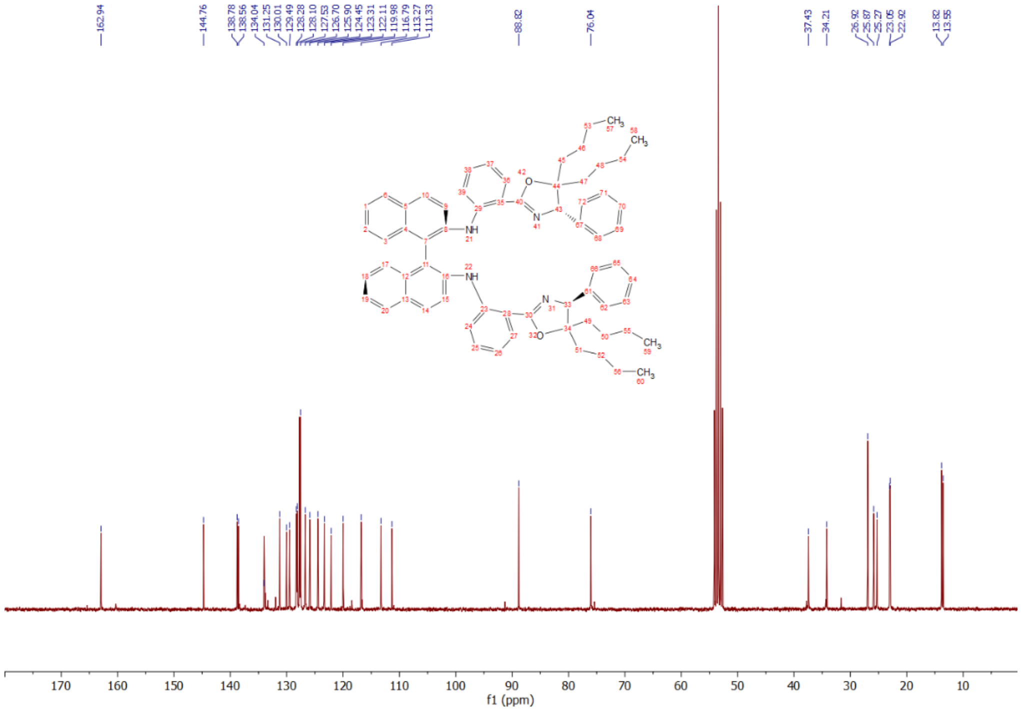

Hex/Hex Ligand

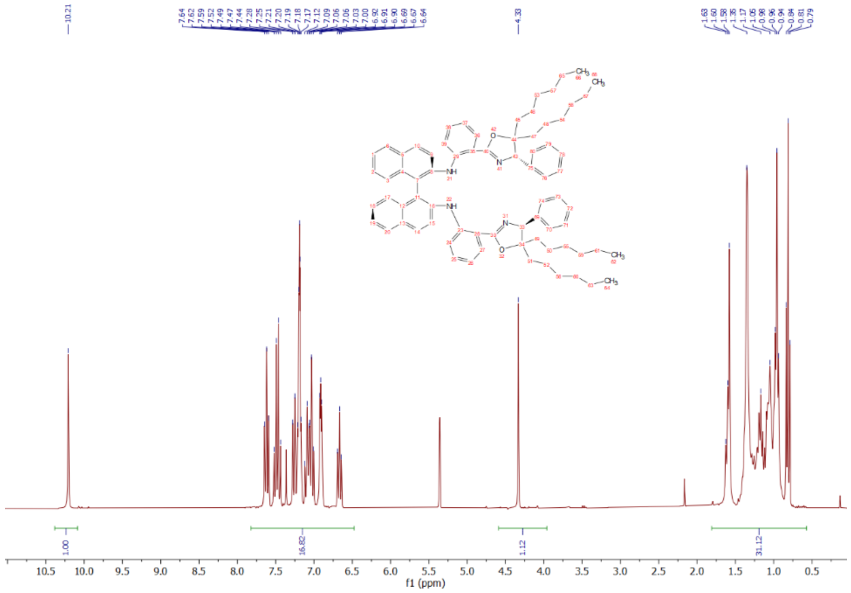

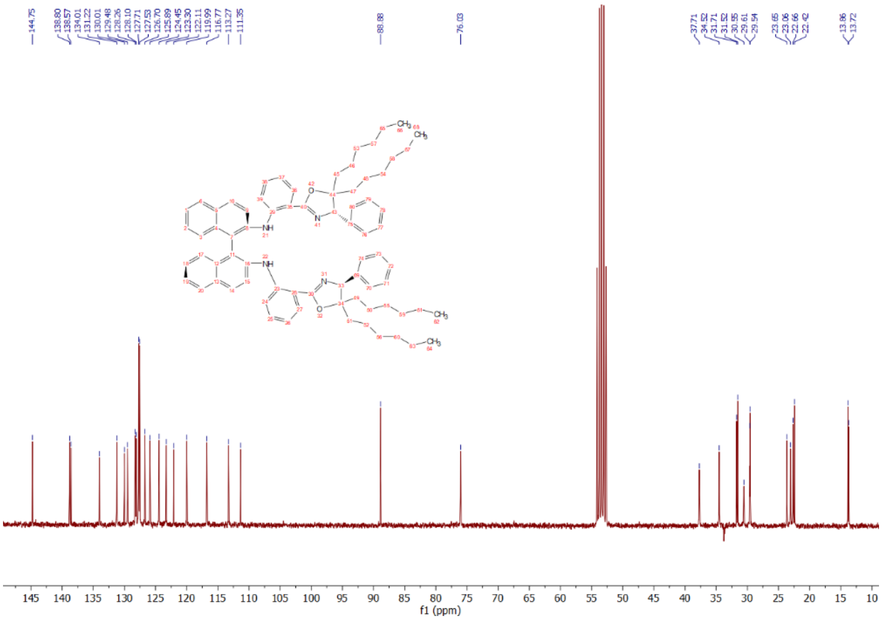

Fe-complexes

H/H-Lig

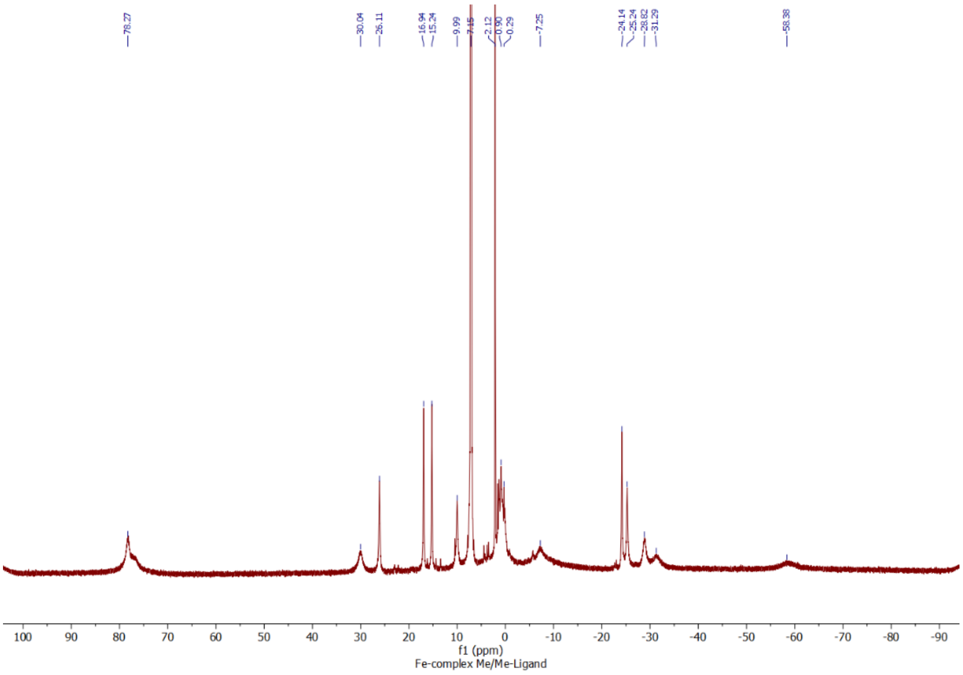

Me/Me-Lig

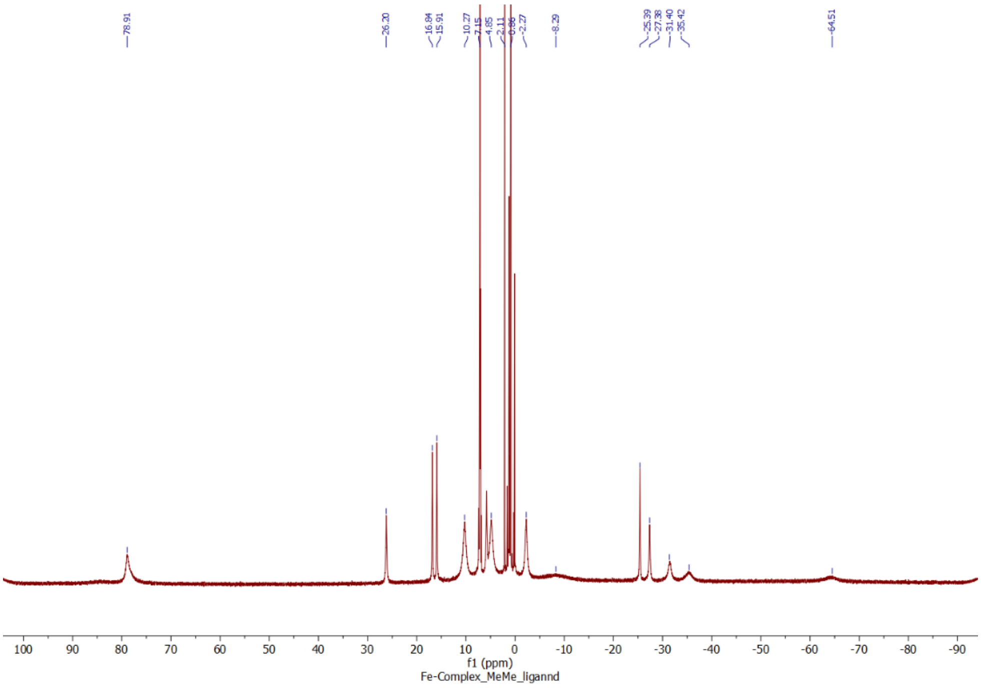

Et/Et-Lig

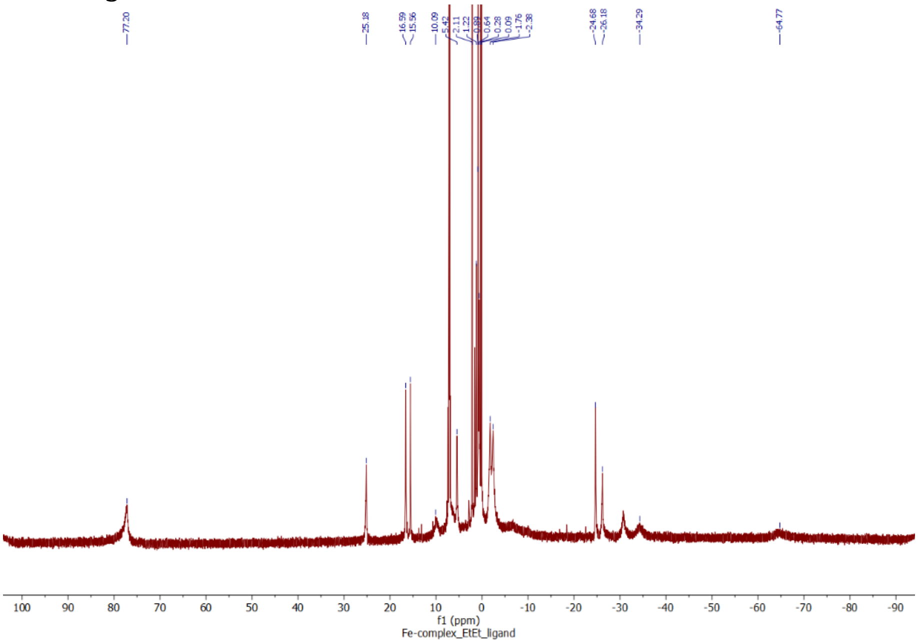

Prop/Prop-Lig

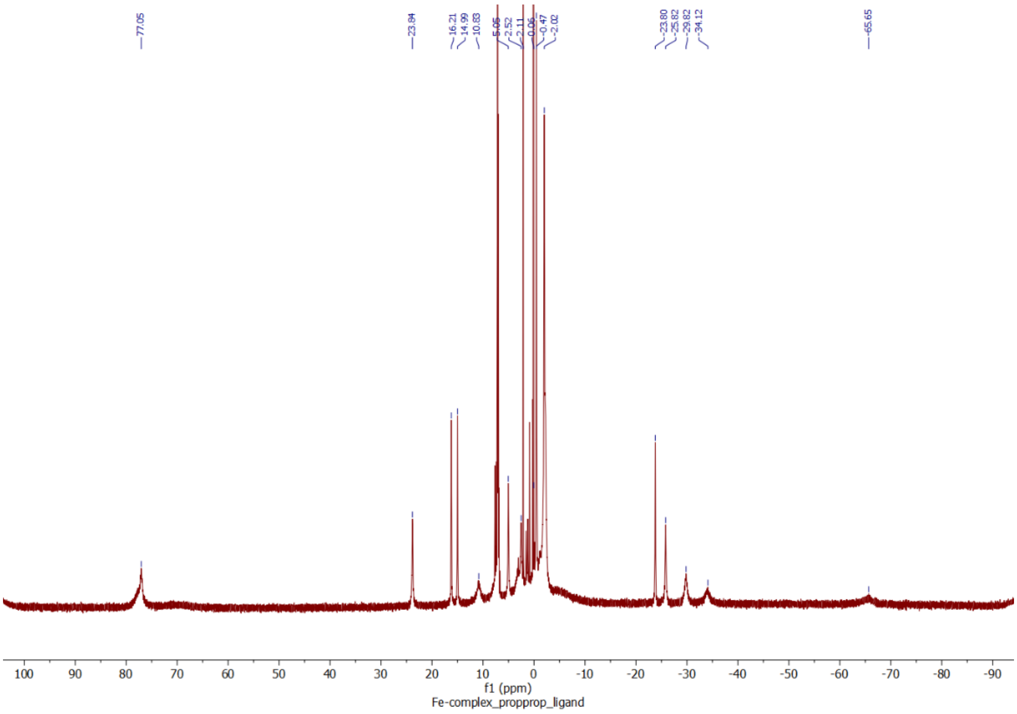

But/But-Lig

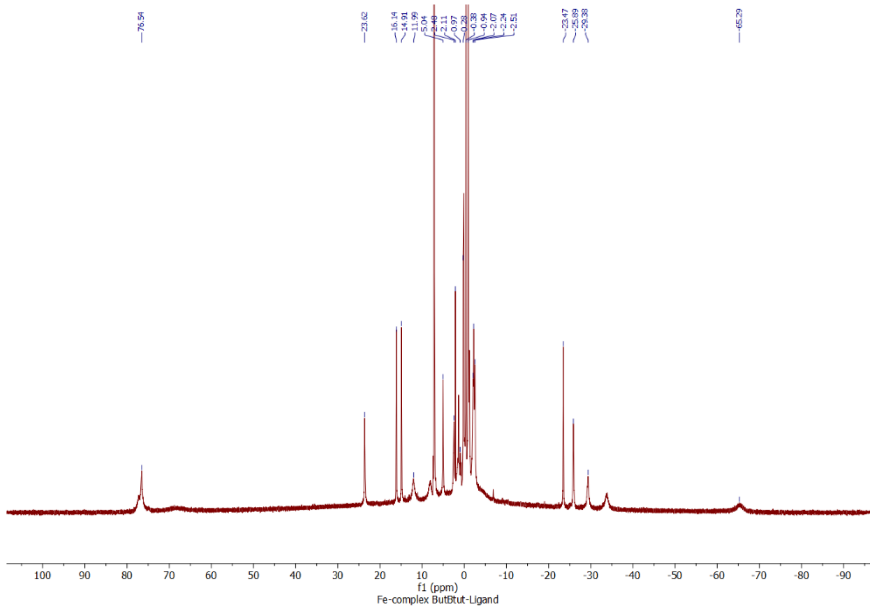

Hex/Hex-Lig

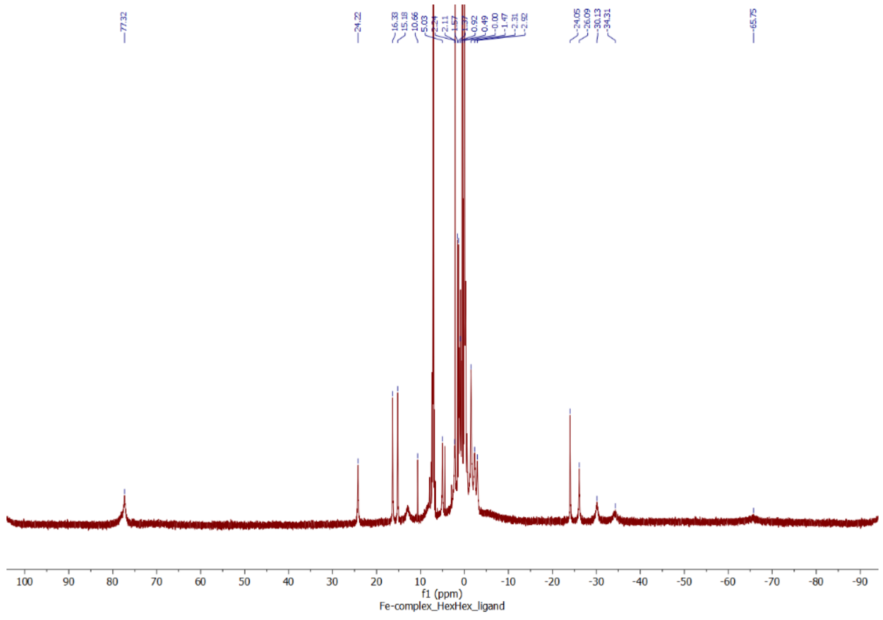

1-(4-azidobutyl)-4-chlorobenzene[S5]

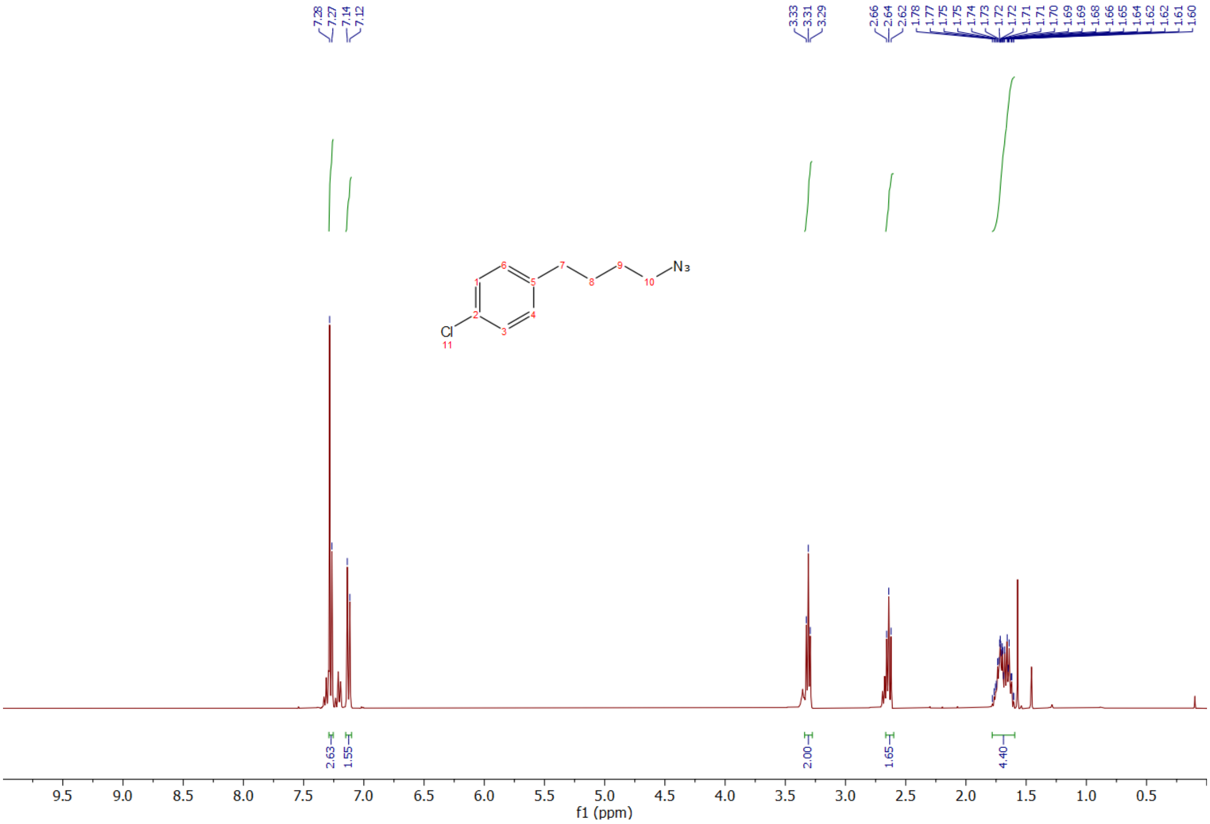

4-(4-azidobutyl)-1,2-dimethoxybenzene[S5]

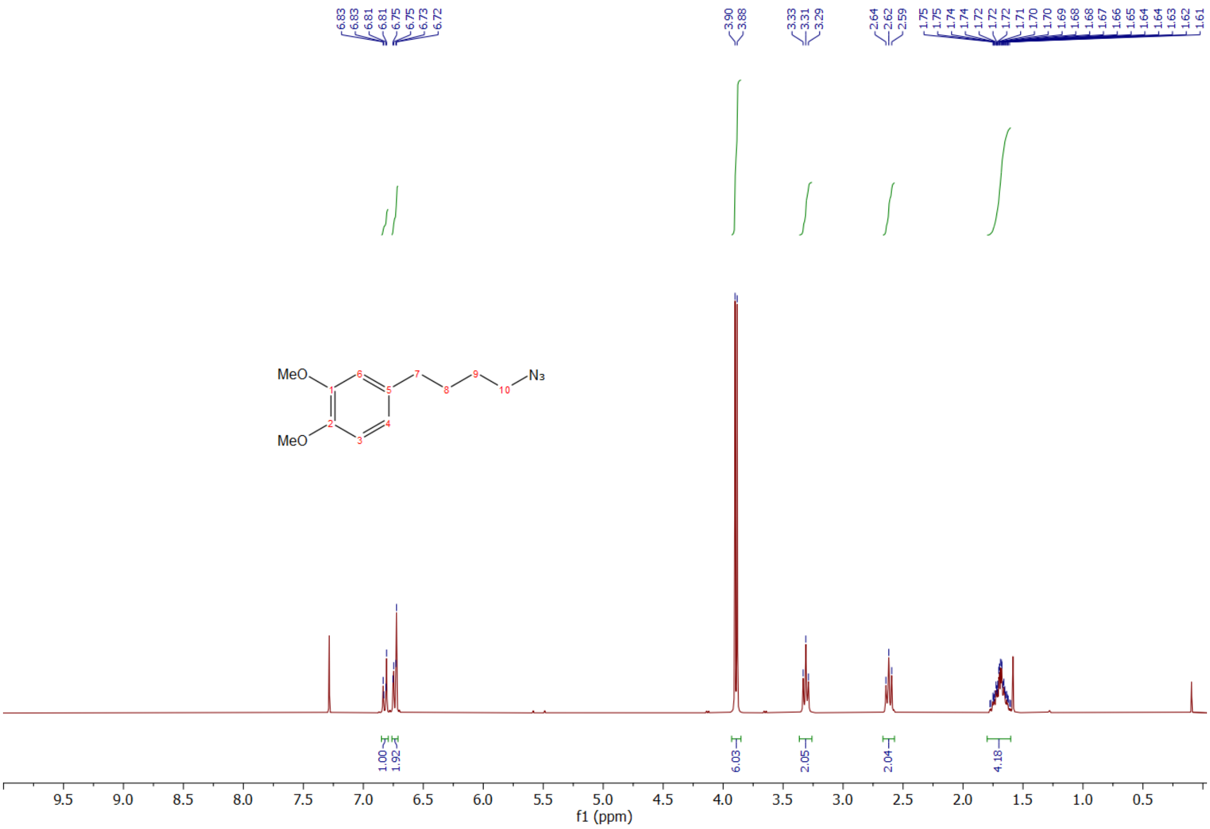

1-(4-azidobutyl)-2,4-dimethylbenzene[S6]

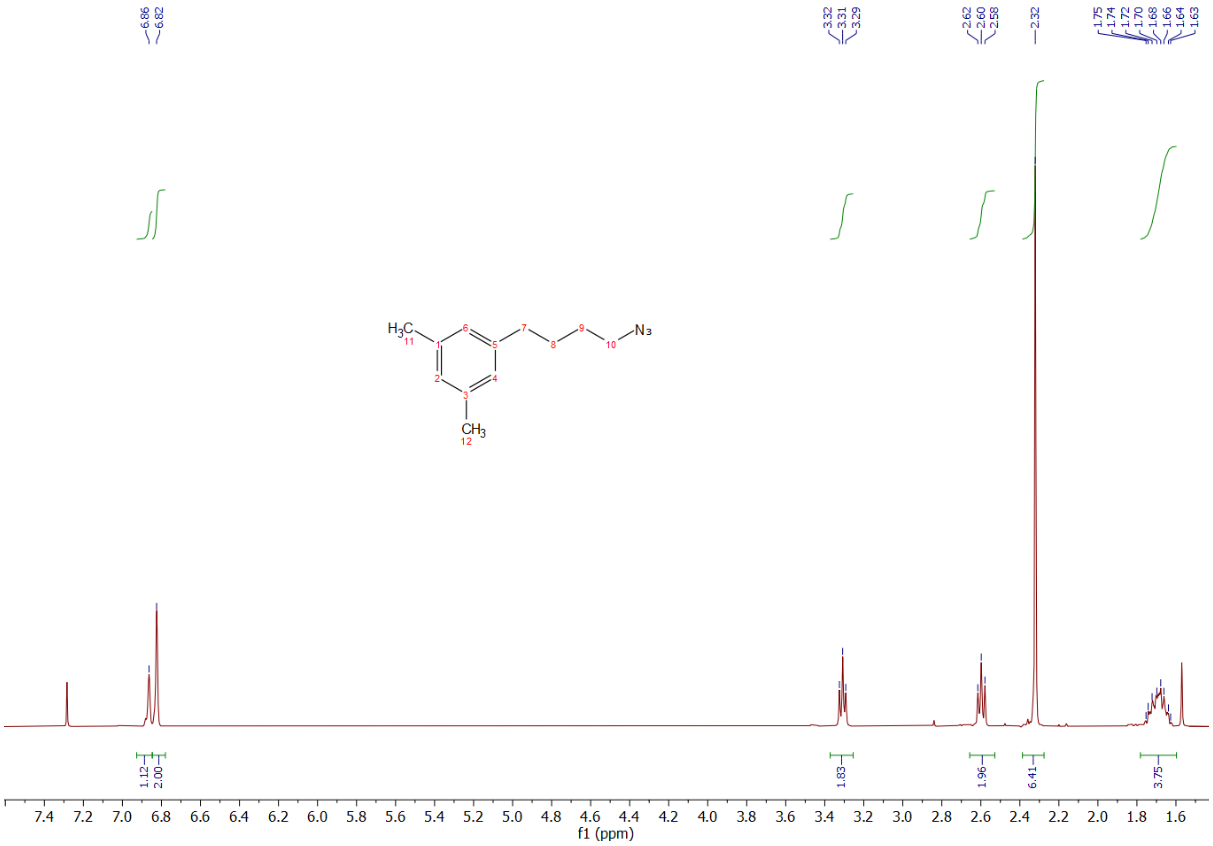

2-(4-azidobutyl)-1,4-difluorobenzene[S6]

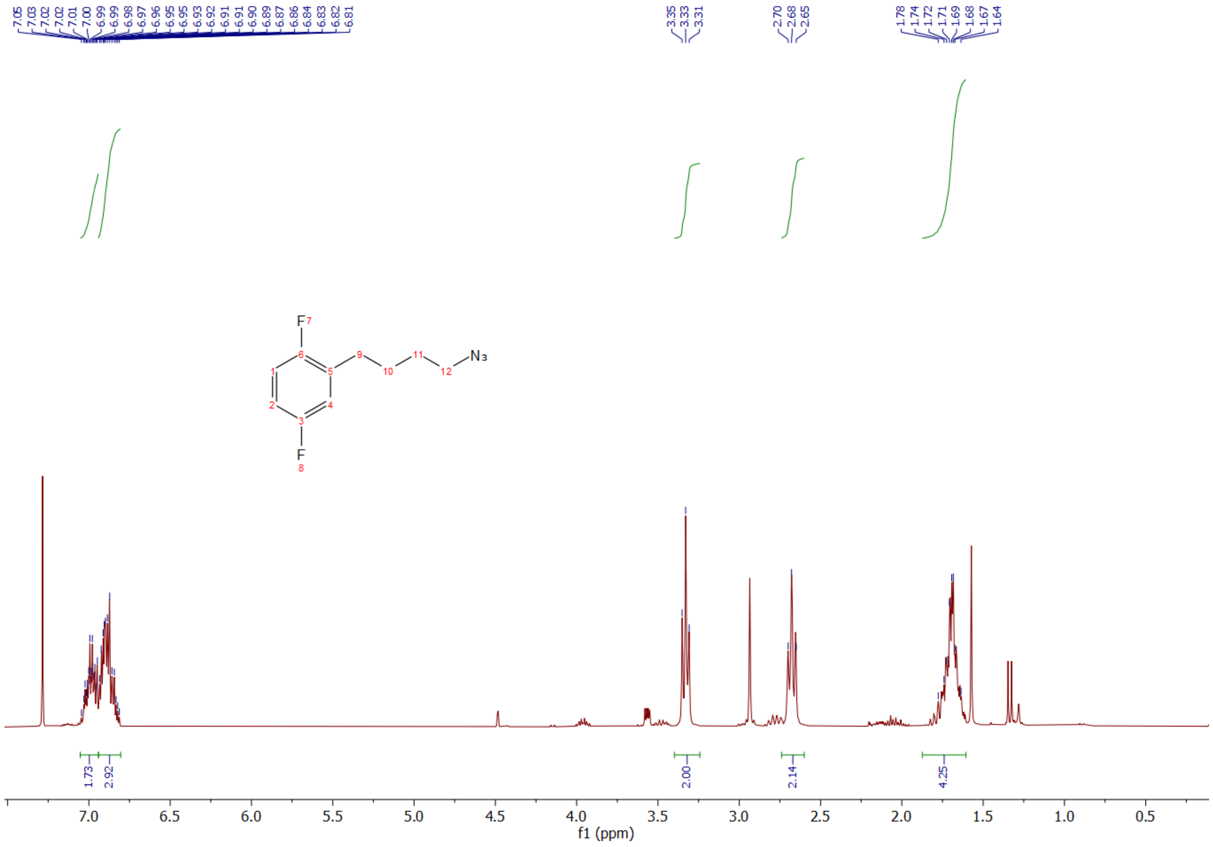

1-(4-azidobutyl)-4-fluorobenzene[S5]

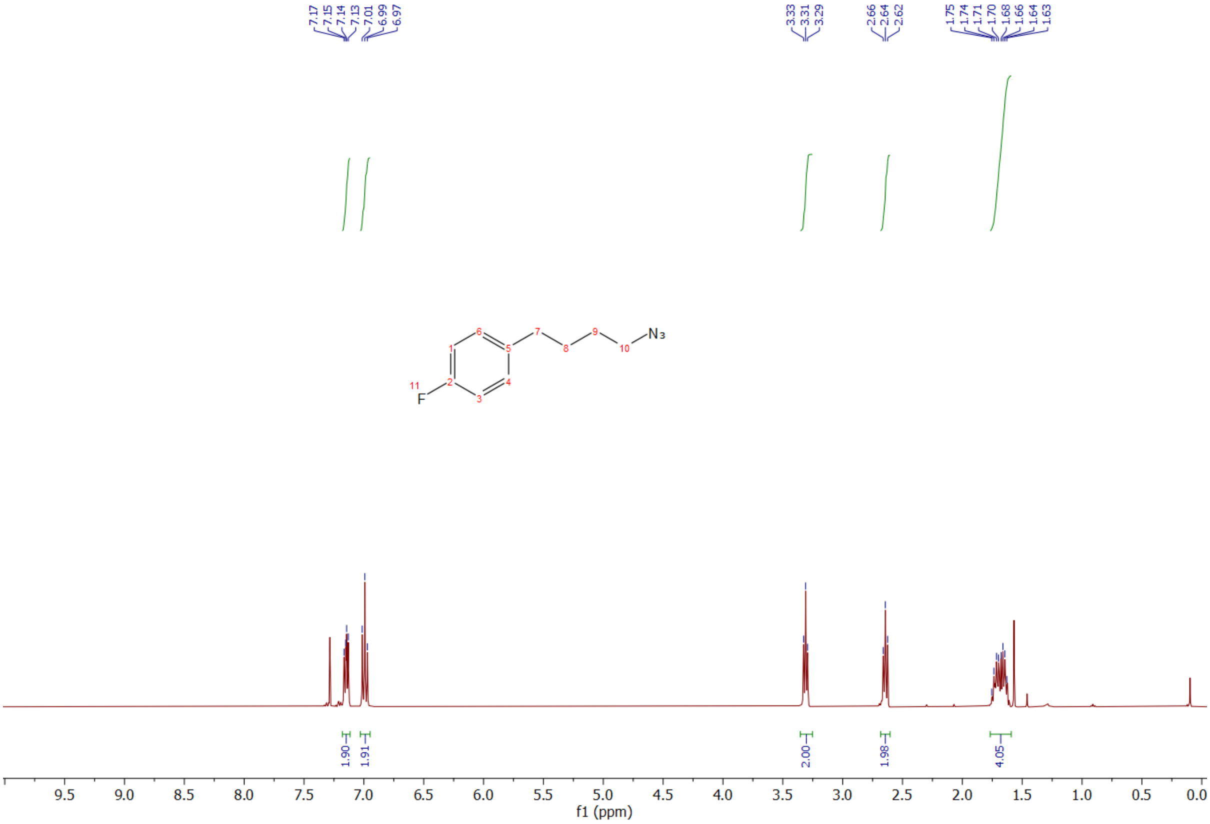

3-(4-azidobutyl)pyridine[S7]

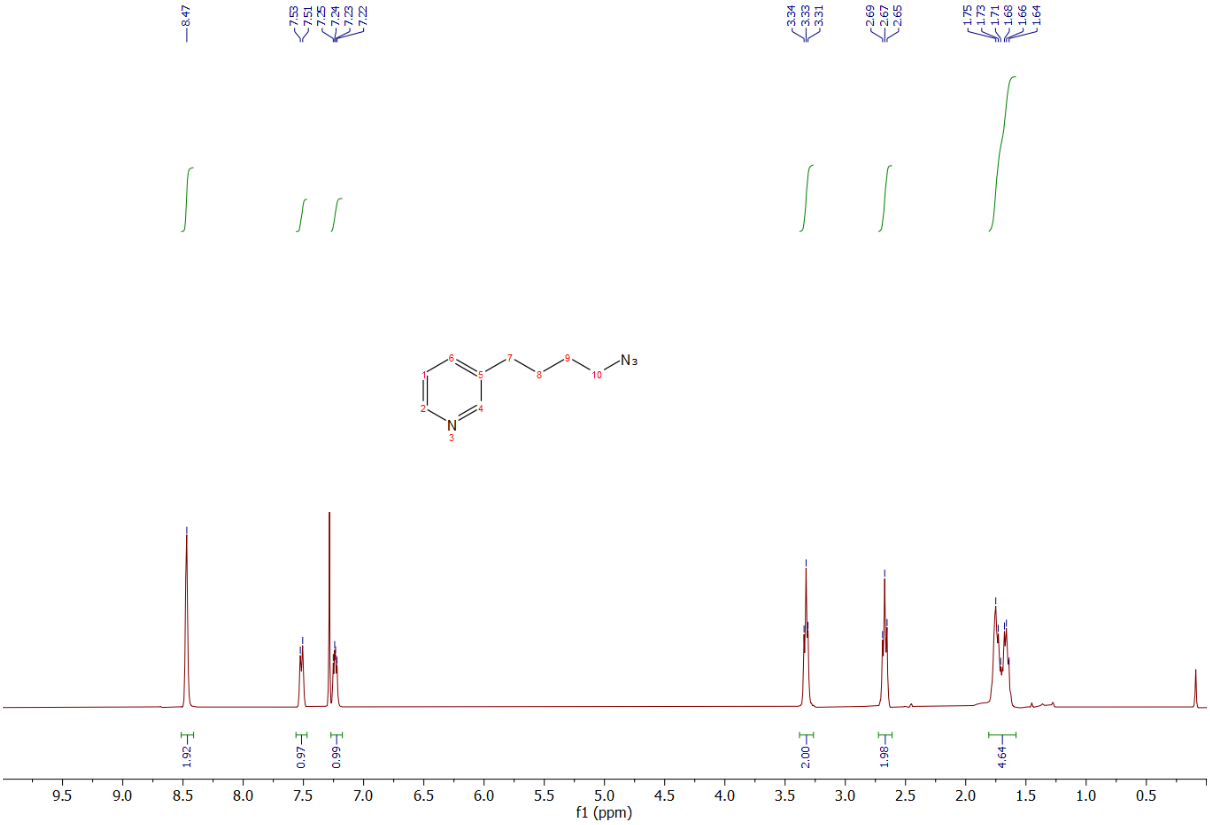

(4-azidobutyl) benzene[S7]

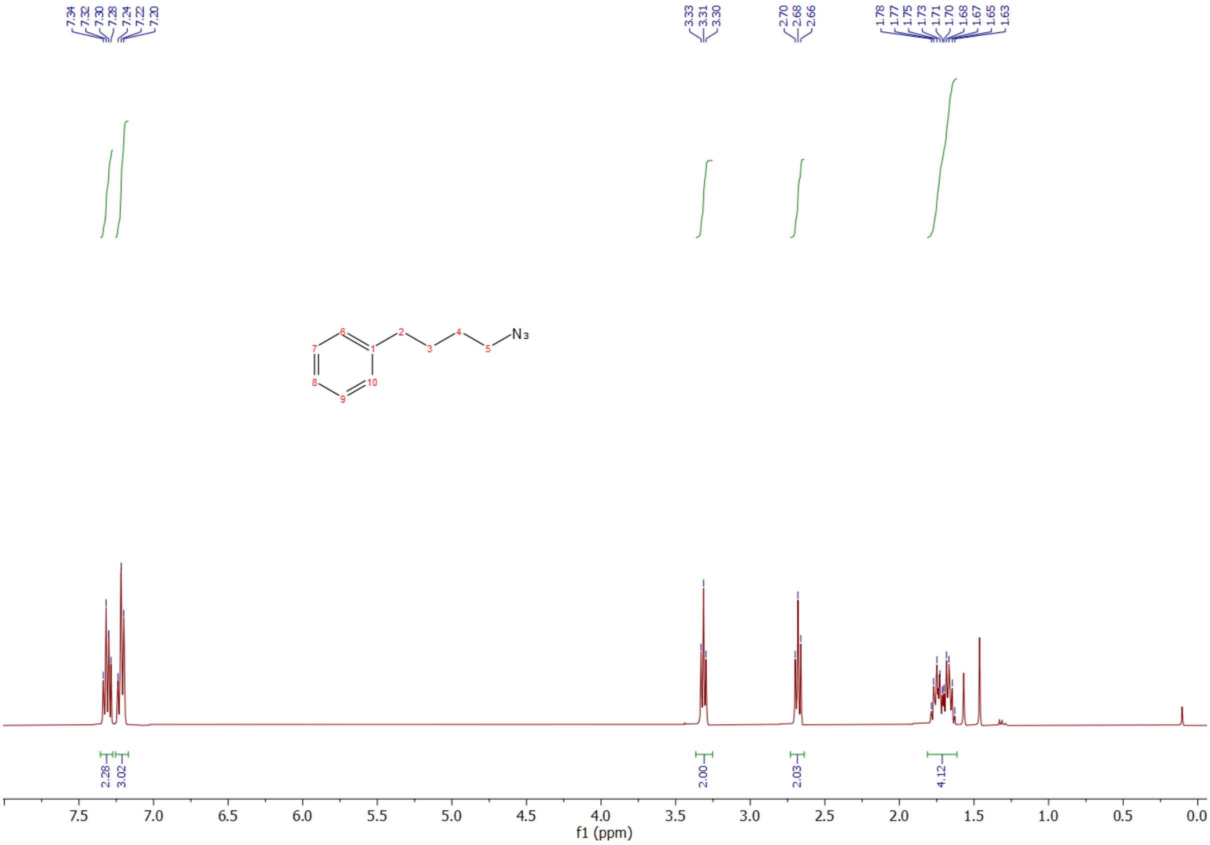

Tert-butyl 2-(4-chlorophenyl)pyrrolidine-1-carboxylate[S5]

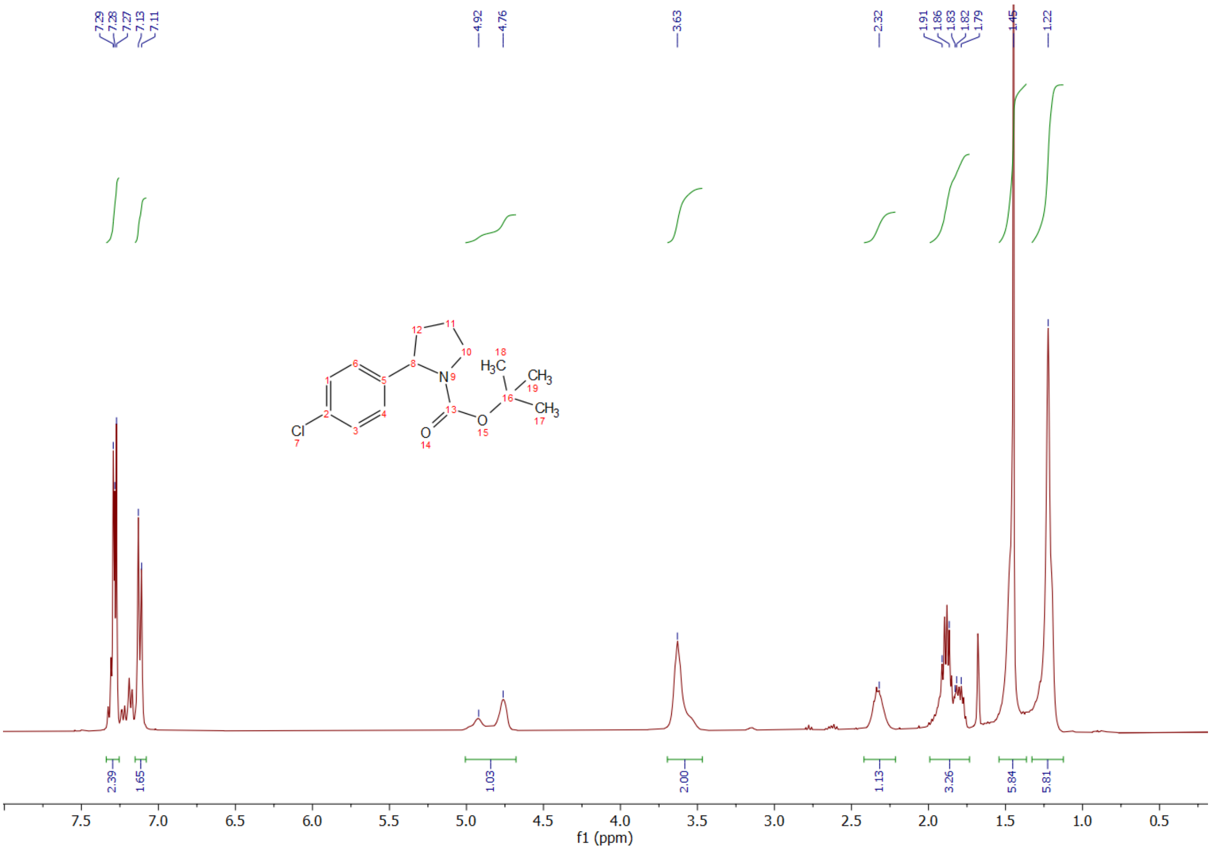

Tert-butyl 2-(3,4-dimethoxyphenyl)pyrrolidine-1-carboxylate[S5]

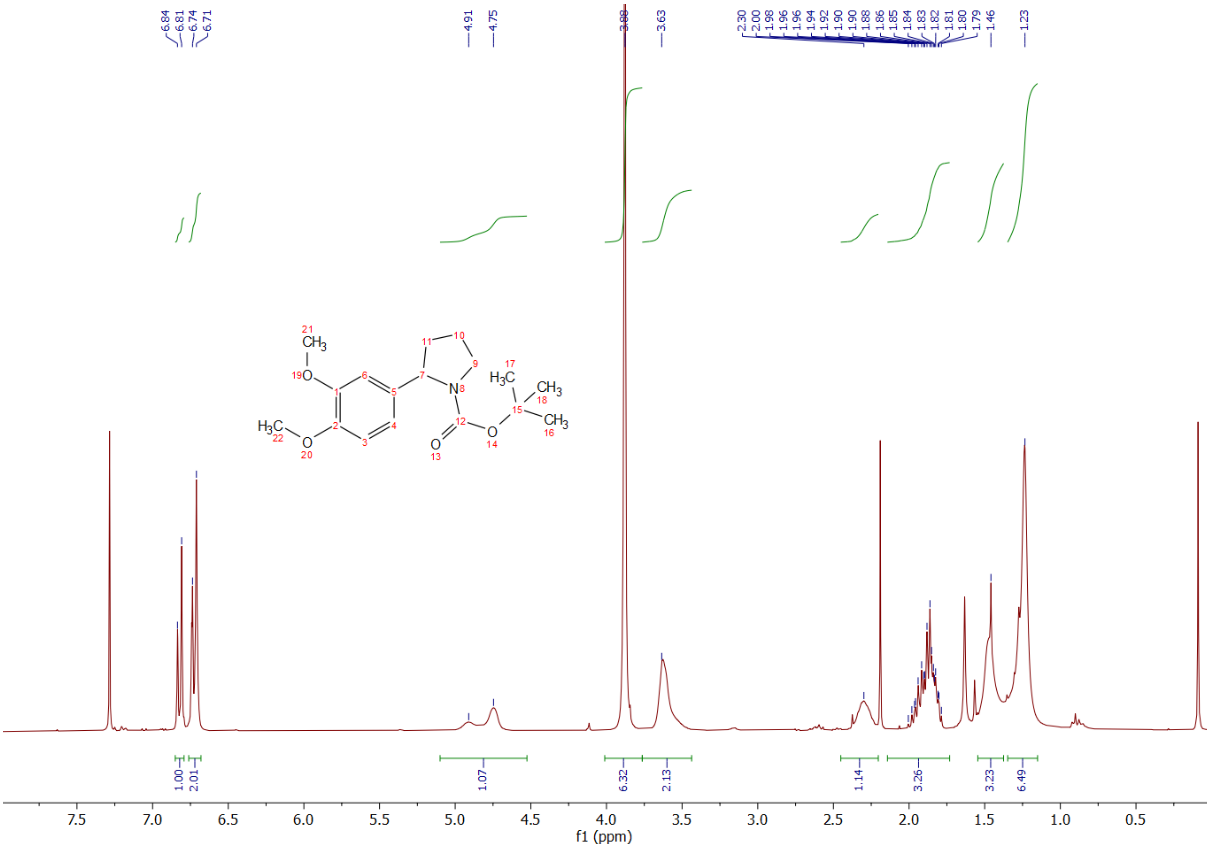

Tert-butyl 2-(3,5-dimethylphenyl)pyrrolidine-1-carboxylate[S6]

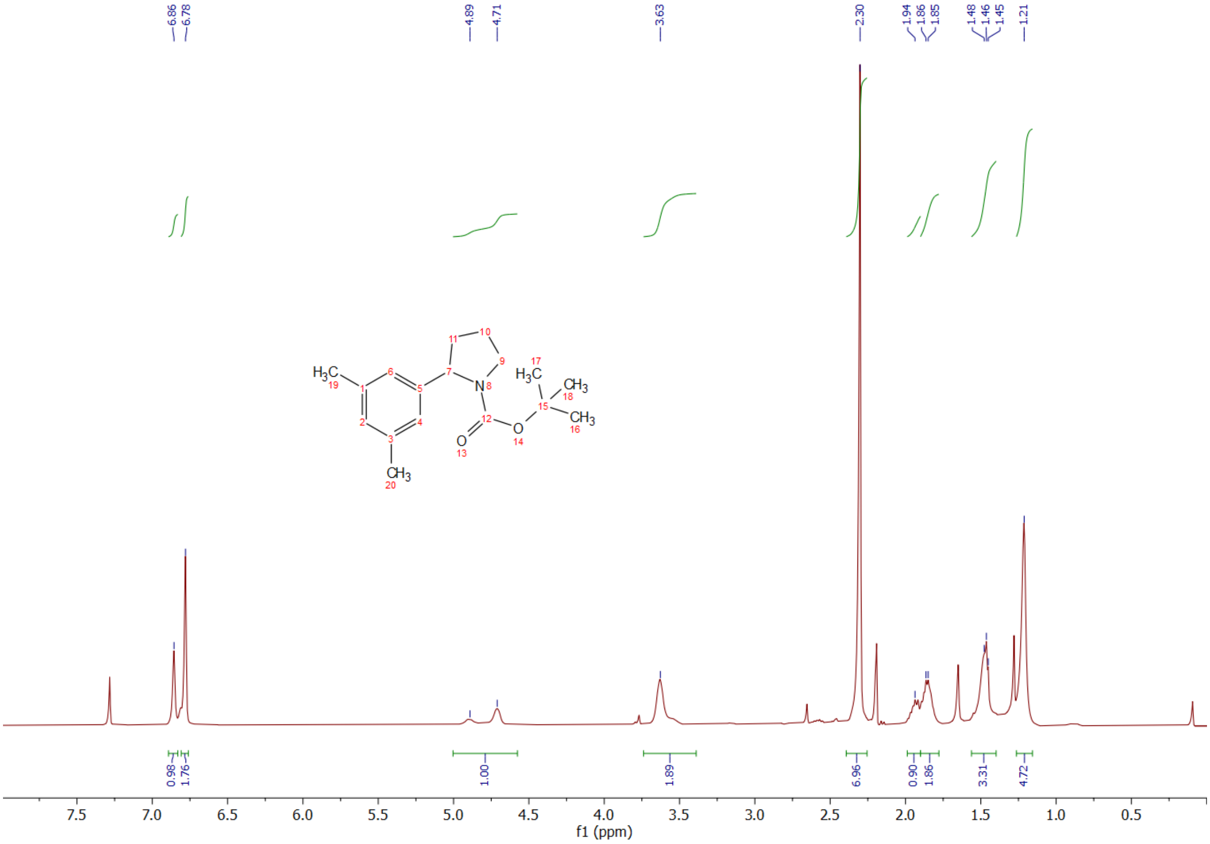

From commercially available (*S*)-2-(3,5-dimethylphenyl)pyrrolidine

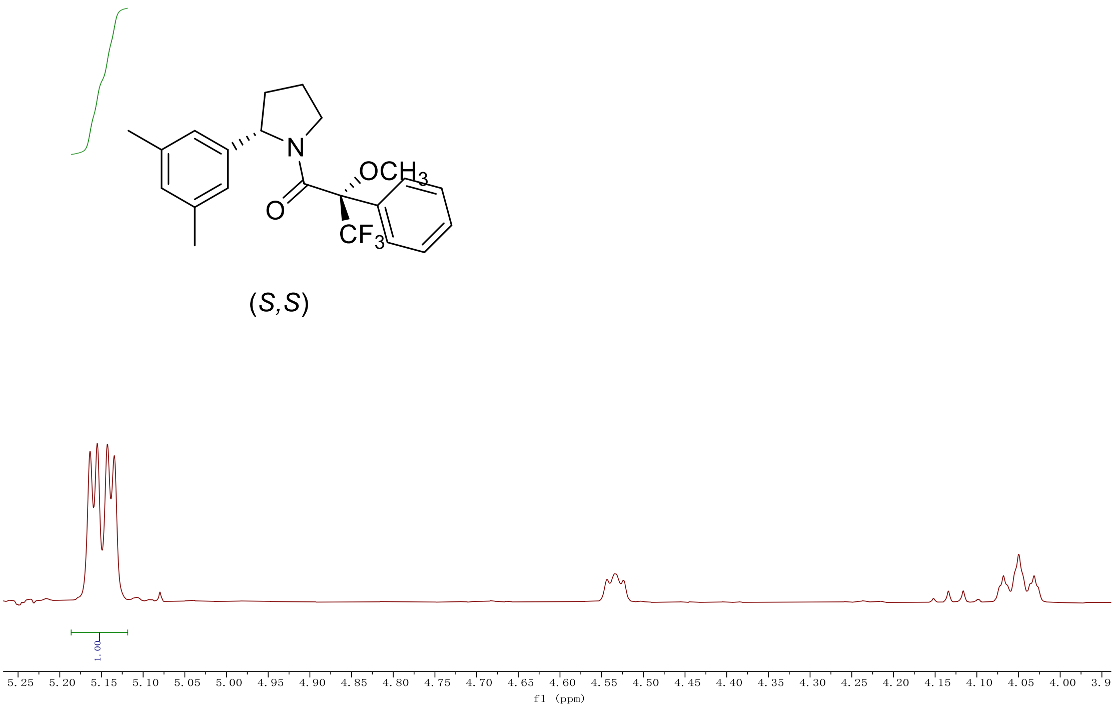

From catalytic reaction.

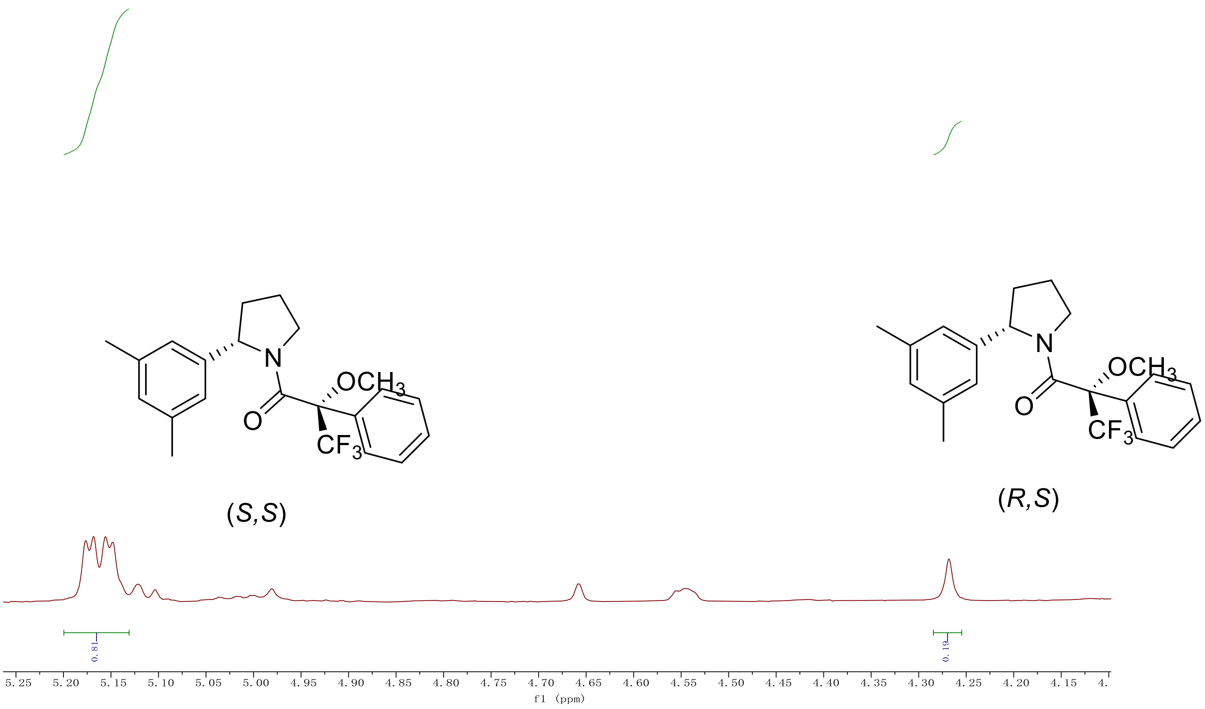

The ee % is 81-19= 62%

Tert-butyl 2-(2,5-difluorophenyl)pyrrolidine-1-carboxylate[S6]

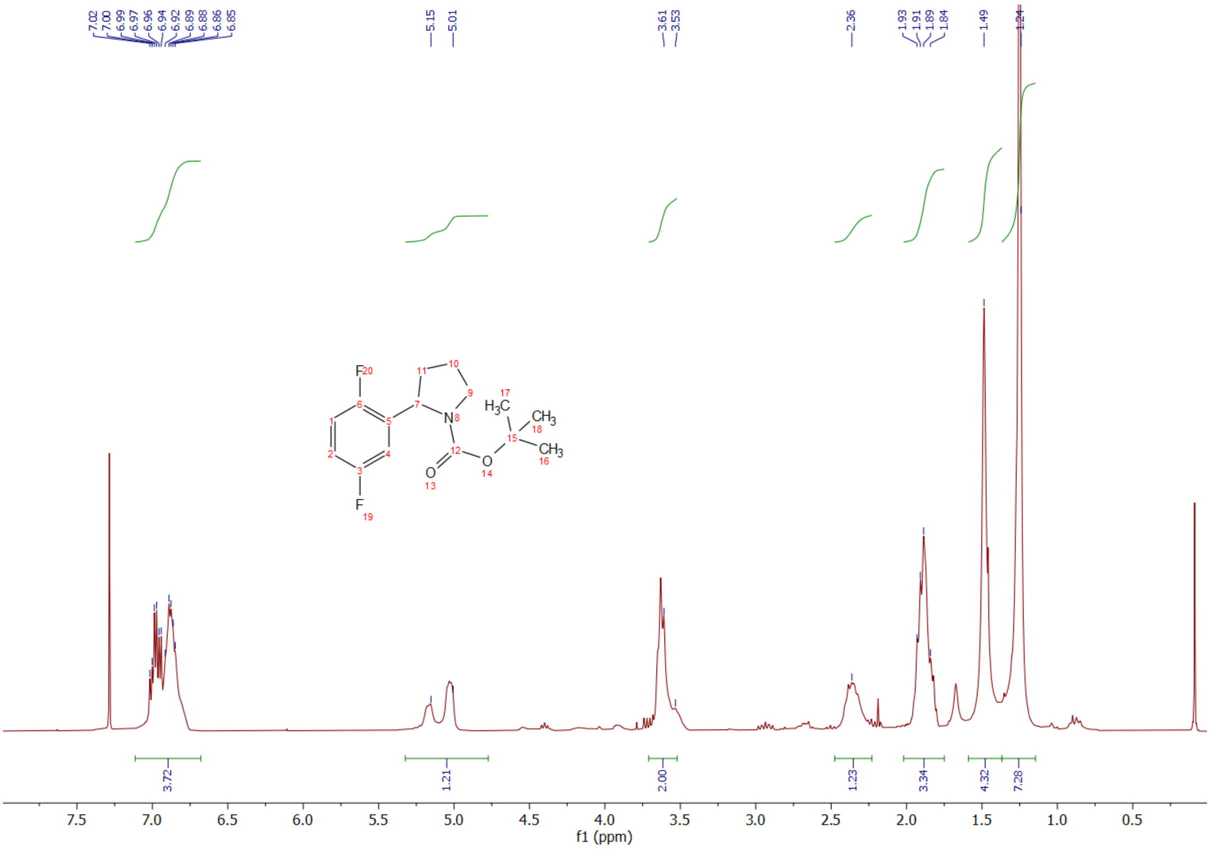

Tert-butyl 2-(4-fluorophenyl)pyrrolidine-1-carboxylate[S5]

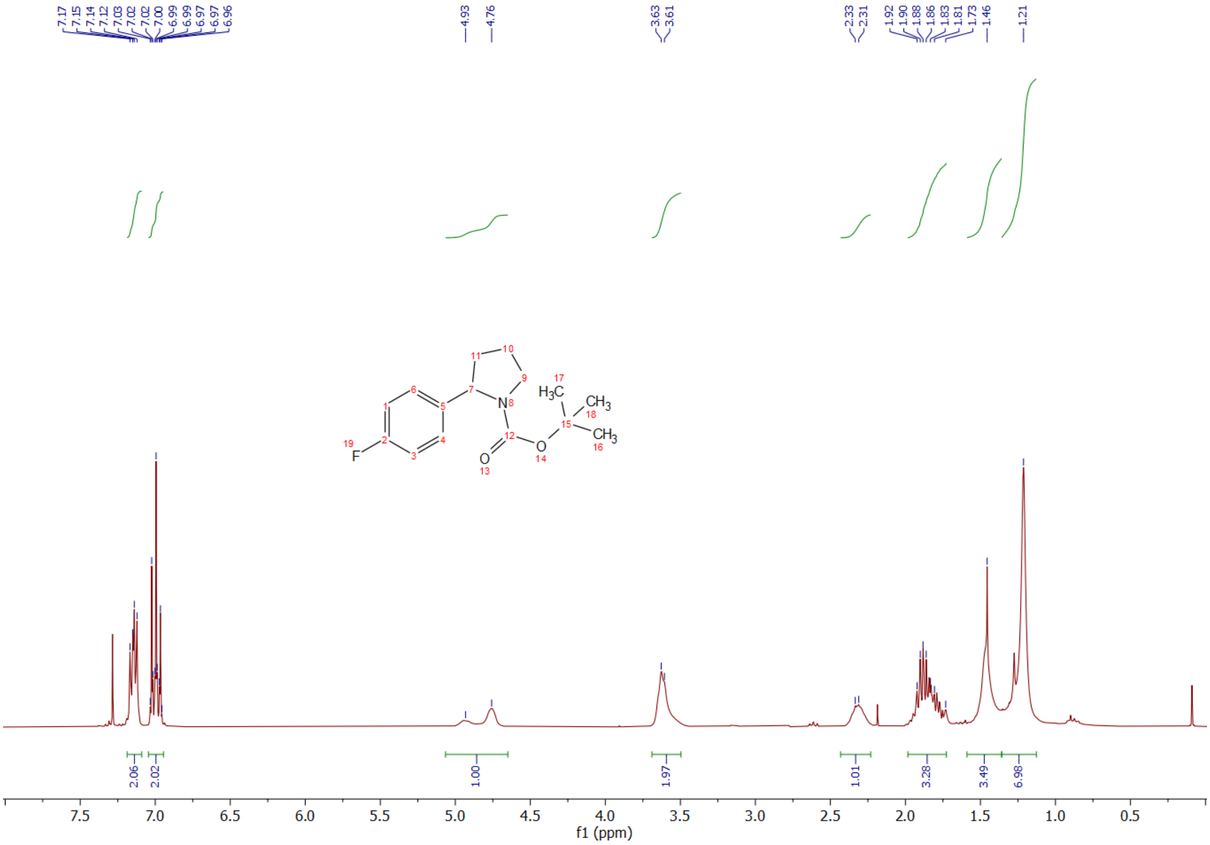

Tert-butyl 2-(pyridin-3-yl)pyrrolidine-1-carboxylate[S7]

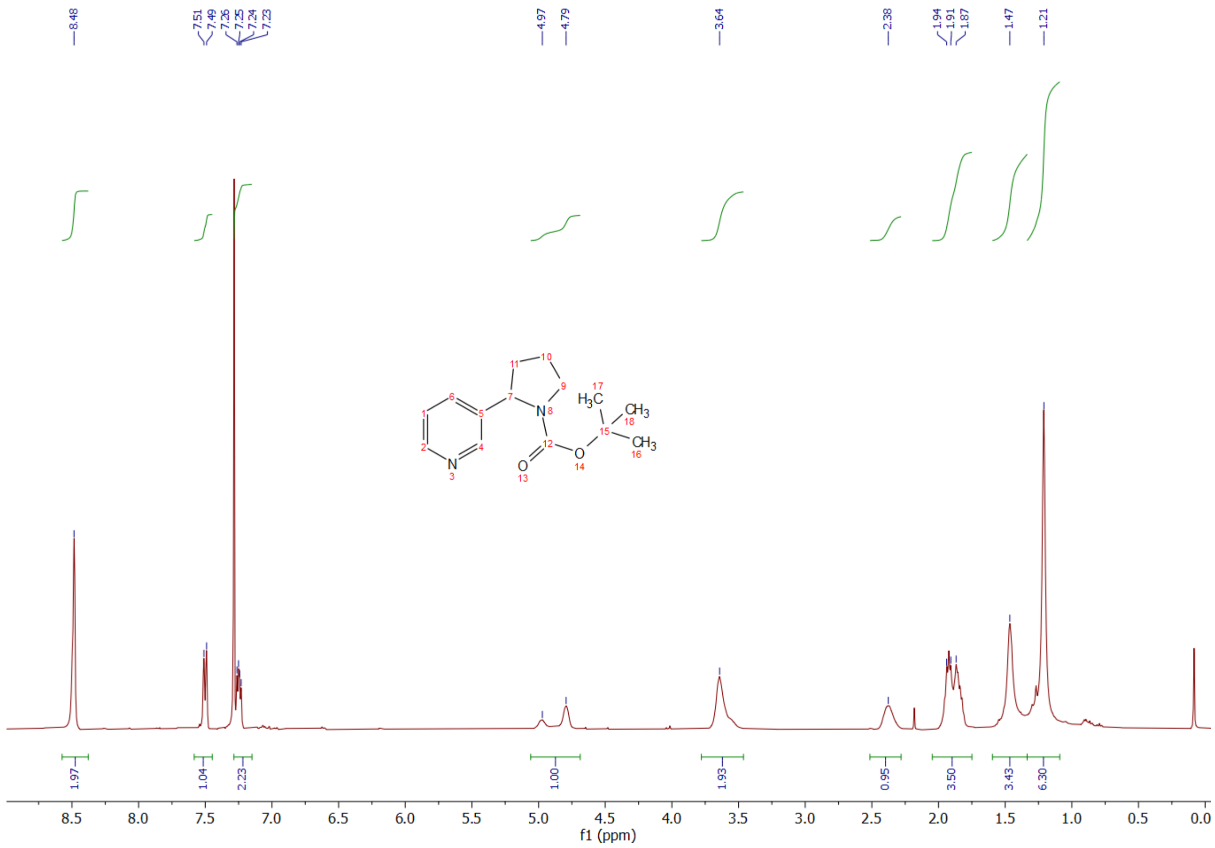

Tert-butyl 2-phenyl pyrrolidine-1-carboxylate [S7]

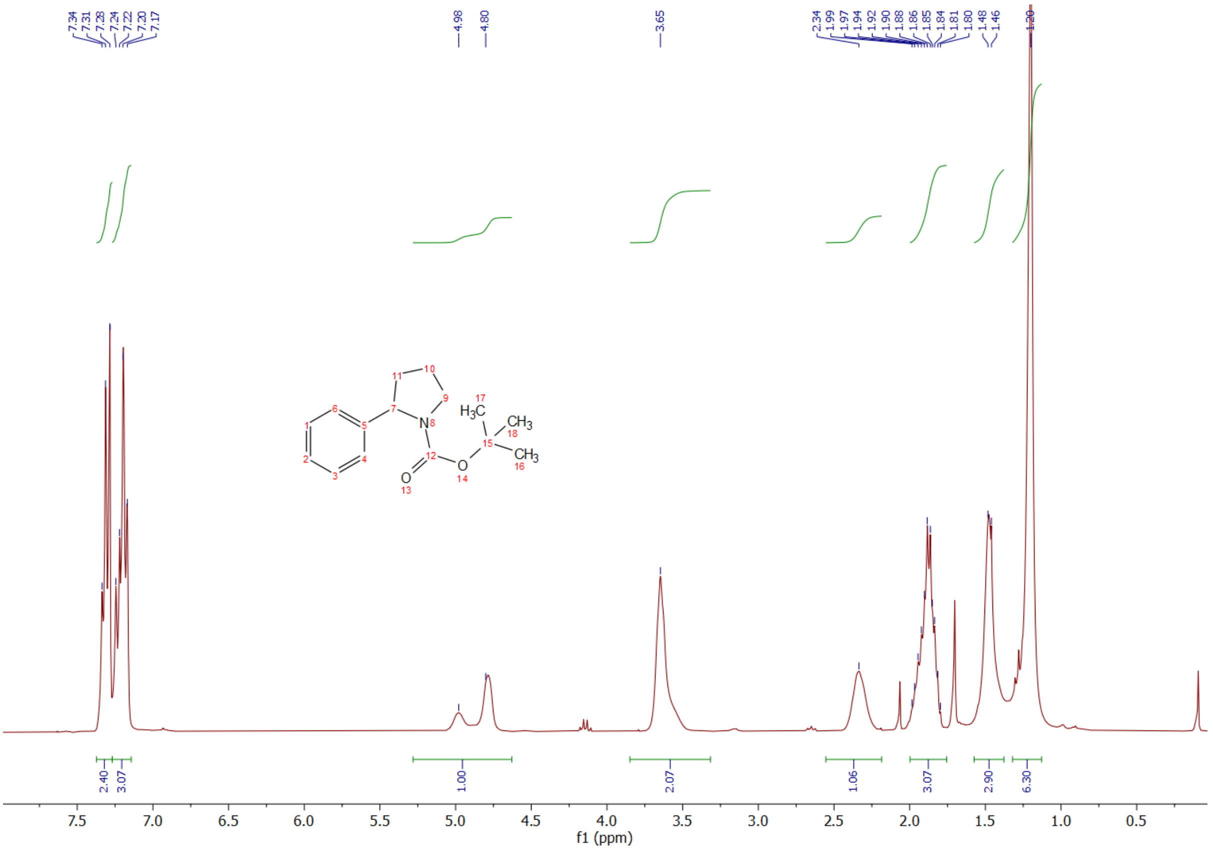

Single-crystal X-ray diffraction (SC-XRD)

Single crystals suitable for SC–XRD were obtained from recrystallization from saturated solutions in the glovebox (*S*,*S*,*S*)-**2** (benzene, r.t.), (*S*,*R*,*R*)-**2** (toluene+ 1 drop pentane, -30°C), (*R*,*R*,*R*)-**2** (benzene, r.t.), and (*R*,*S*,*S*)-**2** (toluene, r.t.). X-ray diffraction data of compound of (*S*,*S*,*S*)-**2**, (*S*,*R*,*R*)-**2**, (*R*,*R*,*R*)-**2** and (*R*,*S*,*S*)-**2** were measured on a Bruker D8 Quest Eco diffractometer using graphite-monochromated (Triumph) Mo K α radiation (γ = 0.71073 Å) and a CPAD Photon III C14 detector. The sample was cooled to 100 K with N_2 via a Cryostream 700 (Oxford Cryosystems). Intensity data were integrated using the SAINT software.[S8] Absorption correction and scaling was executed with SADABS 2016/2.[S9] The structures were solved using intrinsic phasing with the program SHELXT 2018/2[S10] against F^2 of all reflections.

The structure solution of (*S*,*R*,*R*)-**2** contained a partial pentane solvent within the asymmetric unit that could not be refined reliably Thus, the SQUEEZE[S11] procedure in PLATON[S12](version 260325) was applied, showing the presence of 4×~225 Å^3 voids (total solvent accessible volume = 898 Å^3), accounting for 208 electrons per unit cell, congruent with the presence of 5× pentane molecules (42 e^-/molecule, Z=8) in the unit cell (0.625 pentanes/asymmetric unit).

Least-squares refinement was performed with SHELXL-2019/3.[S13] All non-hydrogen atoms were refined with anisotropic displacement parameters. The hydrogen atoms were introduced at calculated positions with a riding model. CheckCIF revealed no A- or B-level alerts.

The X-ray crystallographic data for (*S*,*S*,*S*)-**2**, (*S*,*R*,*R*)-**2**, (*R*,*R*,*R*)-**2** and (*R*,*S*,*S*)-**2** were deposited at the Cambridge Crystallographic Data Centre (CCDC), under the deposition number CCDC 2451830–2451833.

Table S11. Crystal data and structure refinement for (*S*,*S*,*S*)-**2**, (*S*,*R*,*R*)-**2**, (*R*,*R*,*R*)-**2**, (*R*,*S*,*S*)-**2**

**Complex**	**(*S*,*S*,*S*)-2**	**(*S*,*R*,*R*)-2**	**(*R*,*R*,*R*)-2**	**(*R*,*S*,*S*)-2**
**CCDC number**	2451830	2451831	2451832	2451833
**Empirical formula**	$C_{66}H_{56}FeN_4O_2$	$C_{57.12}H_{51.50}FeN_4O_2$	$C_{78}H_{68}FeN_4O_2$	$C_{115}H_{96}Fe_2N_8O_4$
**Formula weight**	992.99	881.87	1149.21	1765.69
**Temperature [K]**	100(2)	100(2)	100(2)	100(2)
**Crystal system**	orthorhombic	orthorhombic	monoclinic	orthorhombic
**Space group (number)**	$P2_12_12_1$ (19)	$P2_12_12_1$ (19)	$P2_1$ (4)	$P2_12_12_1$ (19)
***a* [Å]**	11.8849(6)	18.6889(9)	16.2796(10)	14.9586(9)
***b* [Å]**	15.8610(8)	32.2760(16)	23.6671(14)	18.6704(12)
***c* [Å]**	27.2663(14)	14.9167(7)	16.3870(10)	32.417(2)
**α [°]**	90	90	90	90
**β [°]**	90	90	101.461(3)	90
**γ [°]**	90	90	90	90
**Volume [Å^3]**	5139.9(5)	8997.8(8)	6187.9(7)	9053.6(10)
Z	4	8	4	4
**ρ_{calc} [gcm^{-3}]**	1.283	1.302	1.234	1.295
**μ [mm^{-1}]**	0.345	0.384	0.296	0.382
***F*(000)**	2088	3714	2424	3704
**Crystal size [mm^3]**	0.404×0.257×0.192	0.320×0.271×0.134	0.288×0.234×0.108	0.346×0.271×0.080
**Crystal colour**	red	red	orange	red
**Crystal shape**	block	plate	plate	block
**Radiation**	MoK_α (λ=0.71073 Å)	MoK_α (λ=0.71073 Å)	MoK_α (λ=0.71073 Å)	MoK_α (λ=0.71073 Å)
**2θ range [°]**	5.17 to 63.04 (0.68 Å)	4.37 to 51.38 (0.82 Å)	4.28 to 55.24 (0.77 Å)	3.71 to 55.33 (0.77 Å)
**Index ranges**	−17 ⩽ h ⩽ 17<br>−23 ⩽ k ⩽ 23<br>−40 ⩽ l ⩽ 40	−22 ⩽ h ⩽ 22<br>−39 ⩽ k ⩽ 39<br>−18 ⩽ l ⩽ 18	−21 ⩽ h ⩽ 21<br>−30 ⩽ k ⩽ 30<br>−21 ⩽ l ⩽ 21	−19 ⩽ h ⩽ 19<br>−24 ⩽ k ⩽ 24<br>−42 ⩽ l ⩽ 42
**Reflections collected**	483378	279969	294114	657064
**Independent reflections**	17110<br>R_{int} = 0.0951<br>R_{sigma} = 0.0275	17074<br>R_{int} = 0.1567<br>R_{sigma} = 0.0574	28539<br>R_{int} = 0.0962<br>R_{sigma} = 0.0492	21104<br>R_{int} = 0.0761<br>R_{sigma} = 0.0218
**Completeness to θ = 25.242°**	99.8 %	99.8 %	99.9 %	99.8 %
**Data / Restraints / Parameters**	17110/0/662	17074/0/1108	28539/1/1540	21104/4/1160
**Goodness-of-fit on *F*^{2}**	1.062	1.086	1.089	1.090
**Final *R* indexes [*I*≥2σ(*I*)]**	R_1 = 0.0334<br>wR_2 = 0.0803	R_1 = 0.0616<br>wR_2 = 0.1362	R_1 = 0.0553<br>wR_2 = 0.1213	R_1 = 0.0312<br>wR_2 = 0.0728
**Final *R* indexes [all data]**	R_1 = 0.0390<br>wR_2 = 0.0838	R_1 = 0.0819<br>wR_2 = 0.1466	R_1 = 0.0792<br>wR_2 = 0.1372	R_1 = 0.0385<br>wR_2 = 0.0770
**Largest peak/hole [eÅ^{-3}]**	0.39/-0.24	0.59/-0.63	1.08/-0.90	0.42/-0.40
**Flack X parameter**	0.005(3)	0.11(2)	0.056(14)	0.009(9)

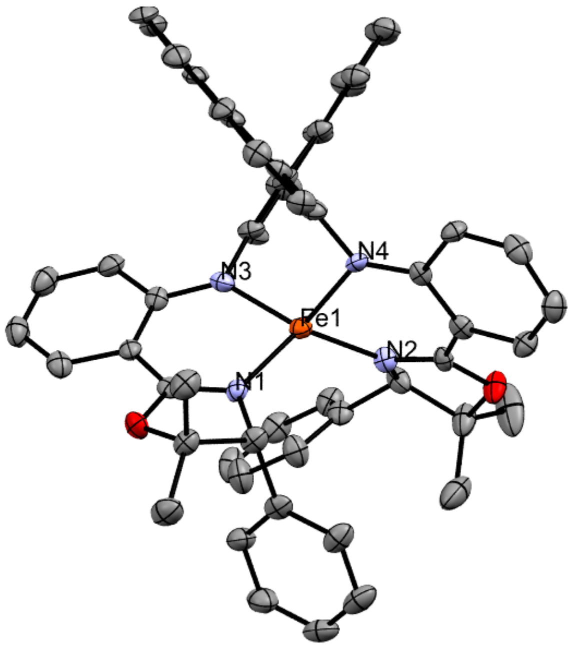

Figure S27. ORTEP plot (50% thermal ellipsoids) of the molecular structure of (*R*,*R*,*R*)-**2.**

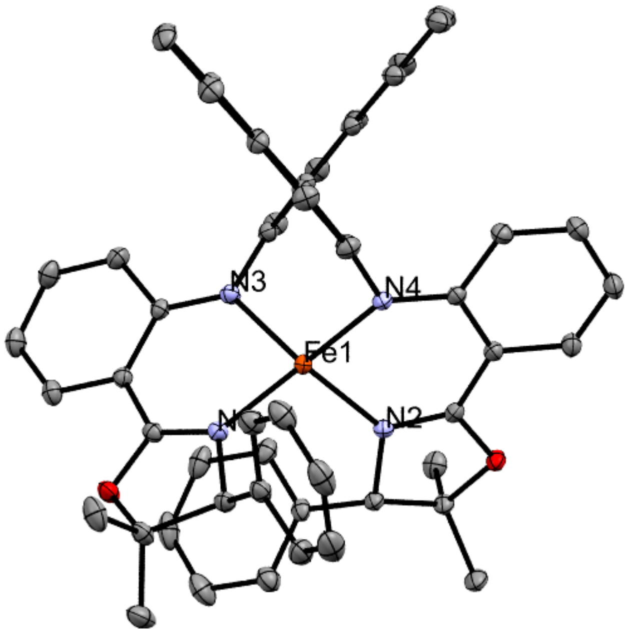

Figure S28. ORTEP plot (50% thermal ellipsoids) of the molecular structure of (*S*,*R*,*R*)-**2.**

DFT calculations

Geometries were fully optimized as minima or transition states using the Turbomole program package,[S14] coupled to the PQS Baker optimizer[S15, S16] via the BOpt package.[S17] We used unrestricted ri-DFT-D3 calculations at the OPBE level,[S18] in combination with the def2-TZVP basis set[S19] and a small (m4) grid size. Grimme's dispersion corrections[S20] (version 3, disp3, zero damping') were used to include Van der Waals interactions. All minima (no imaginary frequencies) and transition states (one imaginary frequency) were characterized by calculating the Hessian matrix. Thermochemical parameters such as the zero-point energy (ZPE), Gibbs free energy and gas-phase thermal corrections (entropy and enthalpy, 298 K, 1 bar) were obtained from these analyses. The nature of the transition states was confirmed by following the intrinsic reaction coordinate (IRC). The relative free energies (ΔG°_{298K} in kcal·mol^{-1}) obtained from these calculations are reported in the main text. For every transition state, the imaginary eigenvalue was followed in both directions to confirm its connection to the relative reactant and product states. A separate archive file is provided, containing an Excel sheet with free energies (ΔG°_{298K}) and negative eigenvalues of the transition states, and all optimized geometries. Optimized geometries of all stationary states and transition states are supplied in .pdb and .xyz format.

Summary for all the data for catalytic reaction.

Table S12. Calculated <S^2> values, energies(in Hartree) of the catalytic intermediates.

	**E(tot)**	**Delta T**	**Delta G**	**<s^2>**	**n_{imag}**
**complex(quintet)**	-3717.93592	0.74407	-3717.19185	6.11	
**complex(triplet)**	-3717.92139	0.74431	-3717.17708	2.1	
**complex(OSS)**	-3717.91715	0.75168	-3717.16547	0	
**TS1(quintet)**	-4271.0289	0.93866	-4270.09024	6.38	-617.81
**TS1(triplet)**	-4271.02573	0.94454	-4270.08119	2.6	-598.69
**nitrene(quintet)**	-4161.56113	0.93686	-4160.62428	6.05	
**nitrene(triplet)**	-4161.55643	0.93632	-4160.6201	2.05	
**TS2-proR(boat)quintet**	-4161.54497	0.93715	-4160.60781	6.07	-1409.05
**TS2-proS(boat)quintet**	-4161.54083	0.93615	-4160.60468	6.07	-1483.63
**TS2-proR(boat)triplet**	-4161.53575	0.93702	-4160.59873	2.7	-1457.98
**TS2-proS(boat)triplet**	-4161.53064	0.93903	-4160.59162	2.07	-1325.35

**TS2-proR(chair)quintet**	-4161.54364	0.93686	-4160.60678	6.06	-1294.39
**TS2-proS(chair)quintet**	-4161.552	0.93724	-4160.61475	6.06	-1224.84
**TS2-proR(chair)triplet**	-4161.5313	0.93716	-4160.59413	2.7	-1608.75
**TS2-proS(chair)triplet**	-4161.54117	0.93687	-4160.6043	2.36	-1284.92
**Int2-proR(boat)quintet**	-4161.55388	0.9428	-4160.61108	6.08	
**Int2-proS(boat)quintet**	-4161.55252	0.93932	-4160.61319	6.06	
**Int2-proR(chair)quintet**	-4161.55176	0.93943	-4160.61234	6.08	
**Int2-proS(chair)quintet**	-4161.5589	0.94113	-4160.61778	6.07	
**Int2-proR(boat)triplet**	-4161.55316	0.93998	-4160.61318	3.07	
**Int2-proS(boat)triplet**	-4161.55648	0.93979	-4160.61668	2.92	
**Int2-proR(chair)triplet**	-4161.55138	0.93974	-4160.61164	3.06	
**Int2-proS(chair)triplet**	-4161.56008	0.94136	-4160.61871	2.93	
**TS3-R-triplet**	-4161.5536	0.94376	-4160.60984	2.55	-115.17
**TS3-S-triplet**	-4161.55485	0.94349	-4160.61136	2.55	-110.47
**product-quintet**	-4161.61739	0.94328	-4160.67412	6.11	
**Product-S-triplet**	-4161.60396	0.94853	-4160.65543	2.09	
**Product-R-triplet**	-4161.59607	0.94921	-4160.64686	2.12	
**TS1,2-HAT-quintet**	-4161.50085	0.93025	-4160.57061	6.15	-1478.49
**TS1,2-HAT-triplet**	-4161.50499	0.93145	-4160.57354	2.22	-1306.55
**After1,2-HAT-quintet**	-4161.59818	0.93336	-4160.66482	6.13	
**After1,2-HAT-triplet**	-4161.59348	0.93892	-4160.65457	2.11	
**alkyazide**	-553.11288	0.17137	-552.94151		
**N2**	-109.51915	-0.01368	-109.53283		

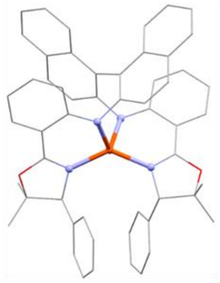

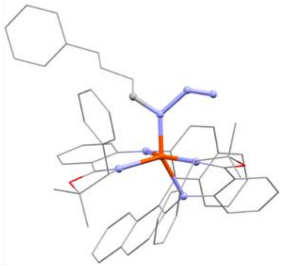

complex(quintet)	-3717.19185
alkyazide	-109.53283
TS1(quintet)	-4270.09024

Structure of Complex

Structure of TS1

Figure S29. Free energy of reaction components for azide activation process at quintet.

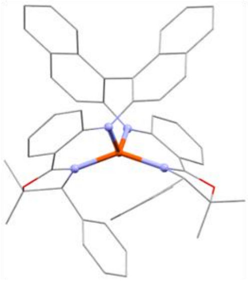

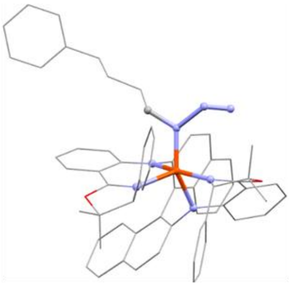

complex(triplet)	-3717.17708
alkyazide	-109.53283
TS1(triplet)	-4270.08119

Structure of Complex

Structure of Int1

Figure S30. Free energy of reaction components for azide activation process at triplet.

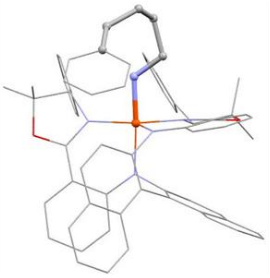

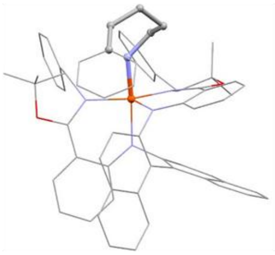

Structure of TS2-ProR(boat)

Structure of Int2-ProR(chair)

nitrene(quintet)	-4160.62428
TS2-proR(boat)quintet	-4160.60781
Int2-proR(chair)quintet	-4160.61234
product-quintet	-4160.67412

Figure S31. Lowest energy pathway of TS2 for R configuration in quintet.

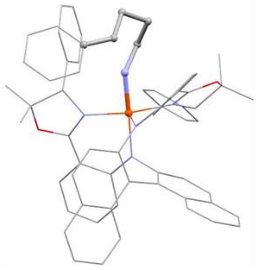

Structure of TS2-ProS(chair)

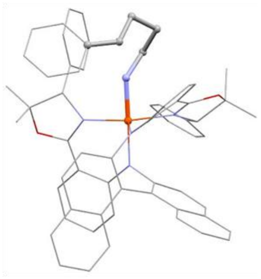

Structure of Int2-ProS(chair)

nitrene(quintet)	-4160.62428
TS2-proS(chair)quintet	-4160.61475
Int2-proS(chair)quintet	-4160.61778
product-quintet	-4160.67412

Figure S32. Lowest energy pathway of TS2 for S configuration in quintet.

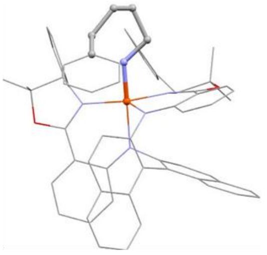

Structure of TS2-ProR(boat)

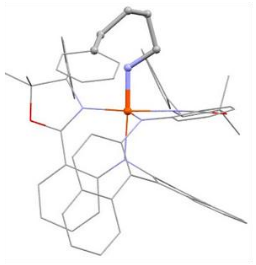

Structure of Int2-ProR(boat)

nitrene(triplet)	-4160.6201
TS2-proR(boat)triplet	-4160.59873
Int2-proR(boat)triplet	-4160.61318
TS3-R-triplet	-4160.60984
Product-R-triplet	-4160.64686

Figure S33. Lowest energy pathway of TS2 for R configuration in triplet.

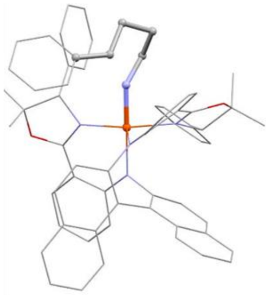

Structure of TS2-ProS(chair)

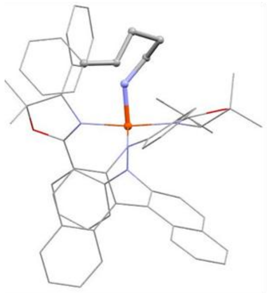

Structure of Int2-ProS(chair)

nitrene(triplet)	-4160.6201
TS2-proS(chair)triplet	-4160.6043
Int2-proS(chair)triplet	-4160.61871
TS3-S-triplet	-4160.61136
Product-S-triplet	-4160.65543

Figure S34. Lowest energy pathway for S configuration in triplet.

In the triplet spin state, hydrogen atom transfer (HAT) also favors abstraction of the pro-*S* benzylic C–H bond (via **TS2-S**, ΔG‡ =12.5 kcal mol^{-1}, rC-H=1.344 Å) over the pro-*R* pathway (via **TS2-R**, ΔG‡ = 16.0 kcal mol^{-1}, rC-H=1.343Å), with a 3.5 kcal mol^{-1} energy difference. These barriers are notably higher than those in the quintet state (6.5 kcal mol^{-1} and 5.7 kcal mol^{-1}, respectively). The resulting benzylic radical intermediates, **Int2-S** (ΔG‡= 3.5 kcal mol^{-1}) and **Int2-R** (ΔG‡ = 7.0 kcal mol^{-1}), exhibit a stability difference of 4.4 kcal mol^{-1} has similiar energy with the quintet states (-0.6 kcal mol^{-1} and -0.5 kcal mol^{-1}, respectively). Because the quintet state undergoes barrierless recombination, spin crossover from quintet to triplet is unlikely. The computed Gibbs free energy barriers for **TS3-S** and **TS3-R** are 4.6 kcal mol^{-1} and 2.1 kcal mol^{-1}, respectively, with a 1.0 kcal mol^{-1} difference. The following product shows that the substrate remains bonded to the metal with a large exergonic energy (product ***S*** ΔG‡ = −27.6 kcal mol^{-1} and product ***R*** ΔG‡ = −23.3 kcal mol^{-1}). These values are significantly more favorable than those of the quintet state (*S*-product: 11.8 kcal mol^{-1}; *R*-product: 17.1 kcal mol^{-1}). Collectively, these thermodynamic data indicate that the reaction pathway proceeds preferentially on the quintet state surface.

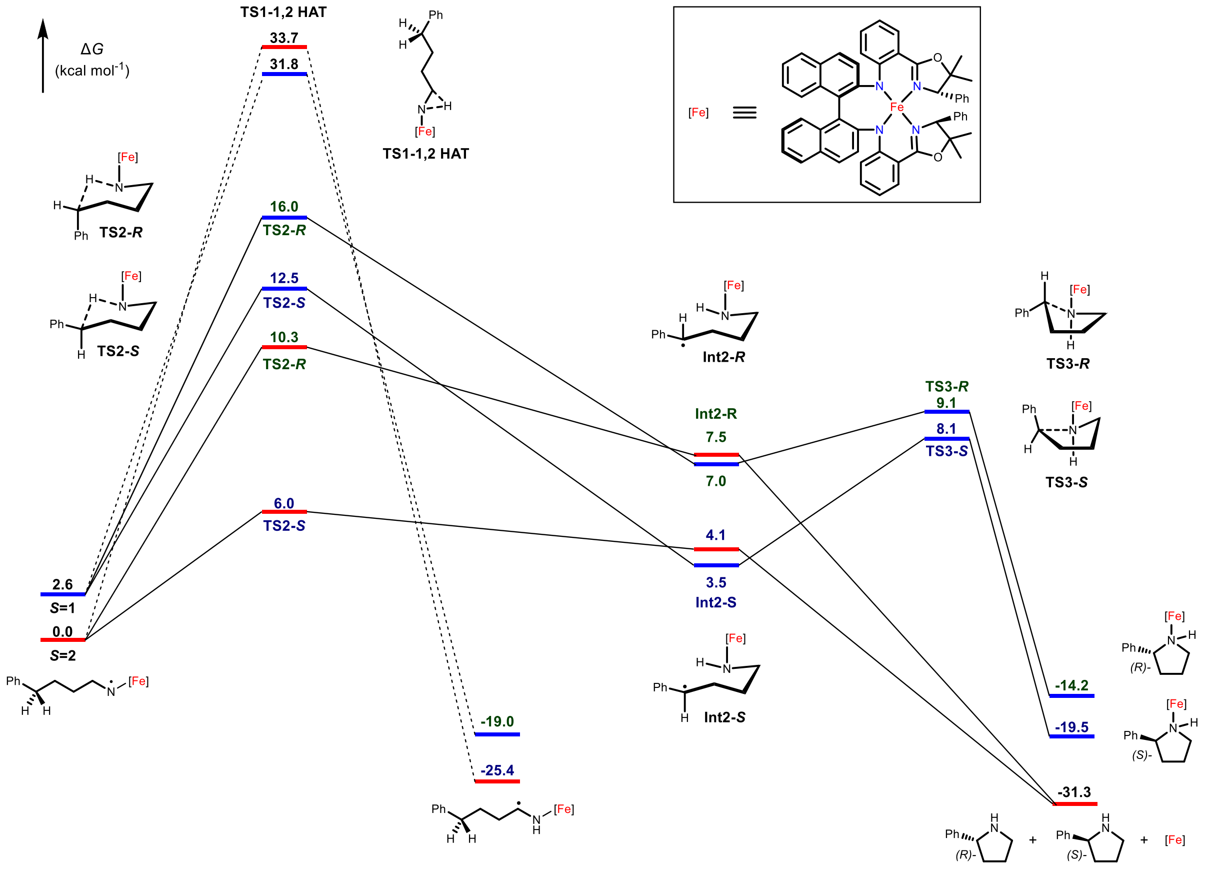

Figure S35. Computed reaction free energy profiles of the Fe-catalyzed asymmetric intramolecular nitrene transfer with *R*,*S*,*S*-**2**. (red for quintet and blue for triplet)

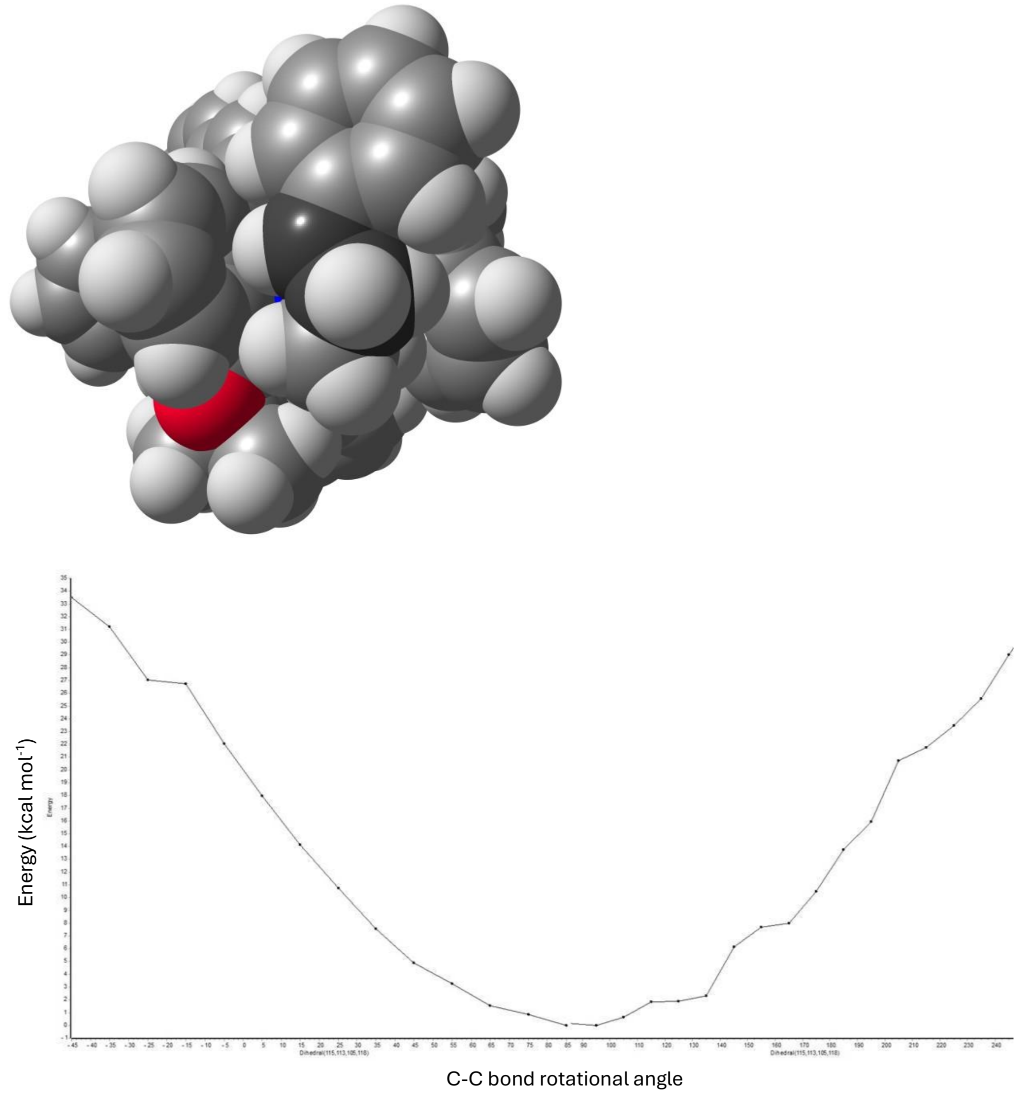

Figure S36. Top: Space-filling model of the benzylic carbon-centered radical derived from **S1** as generated upon HAT. Bottom: Result of a linear transit calculation, whereby the relevant C-C bond (indicated in black in the space-filling model) is rotated in steps of 10 degrees in either direction using constrained geometry optimizations (fixed $C_{aromatic}$-C(H)-C(H_2)-C(H_2) dihedredral angles).

These constrained geometry optimizations suggest that rotating around the CH(radical)-CH_2 bond is associated with very high potential energy walls in the order of 20-30 kcal mol^{-1}. While we did not perform a full MD analysis, which would be out of scope for this paper, these DFT studies do reveal that the barrier for rotation around the C-C bond (thus leading to racemization after the initial HAT step) has a high barrier.

SI for coordinate:

complex(quintet) (E_{tot} -3717.93592)

Fe	5.1408933	16.7630227	15.0727311
O	3.7444293	15.7647981	11.3292508
N	3.6525189	18.0664267	14.9371409
N	4.6910638	15.9741211	13.3466114
C	5.0157263	21.0269839	16.7441759
C	5.9042138	22.1168956	16.5622493
H	6.3988184	22.2238479	15.5999135
C	6.1356783	23.0186800	17.5752494
H	6.8216677	23.8509904	17.4140869
C	5.4986011	22.8700418	18.8280231
H	5.6924968	23.5889249	19.6244889
C	4.6364190	21.8170358	19.0396647
H	4.1428555	21.6904324	20.0044161
C	4.3719491	20.8749466	18.0155353
C	3.5009733	19.7772141	18.2168149
H	3.0223678	19.6564801	19.1897730
C	3.2518434	18.8820572	17.2031189
H	2.5751793	18.0401704	17.3435396
C	3.8679016	19.0295305	15.9382980
C	4.7681944	20.0804607	15.7152300
C	3.0409162	18.4577436	13.7926111
C	2.2535030	19.6422059	13.7610477
H	2.1925546	20.2289486	14.6737728
C	1.6046752	20.0646309	12.6237392
C	1.6859710	19.3241133	11.4298466
C	2.4271683	18.1630297	11.4197166
H	2.5081218	17.5765038	10.5075154
C	3.1162163	17.7039564	12.5689011
C	5.0215902	14.6252928	12.9087095
H	6.0812431	14.4172938	13.0981449
C	4.7697716	14.7376057	11.3802121
C	3.8715231	16.4974233	12.4568750
C	4.1870898	13.5919892	13.6309061
C	3.0704823	13.9601966	14.3836629
H	2.8218978	15.0162040	14.4822537
C	2.2929637	12.9903287	15.0154610
H	1.4262433	13.2930283	15.6042702
C	2.6148996	11.6397703	14.8862652
H	2.0032761	10.8797149	15.3731383
C	3.7334123	11.2682393	14.1398231
H	4.0095552	10.2173551	14.0547679
C	4.5196183	12.2395550	13.5291789
H	5.4145762	11.9474518	12.9832952
C	4.2198283	13.5186963	10.6787677
H	4.9598148	12.7120949	10.6915006
H	3.9973125	13.7722102	9.6356757
H	3.3086857	13.1669946	11.1703986
C	6.0130970	15.2889631	10.6956848
H	6.3592991	16.1864733	11.2200198
H	5.7831620	15.5481112	9.6560676
H	6.8104556	14.5358714	10.7075404
O	6.5368571	15.7678429	18.8172683
N	6.6279290	18.0678216	15.2079175
N	5.5903437	15.9758895	16.7996901
C	5.2625432	21.0302935	13.4058399
C	4.3736725	22.1195358	13.5898863
H	3.8792451	22.2246278	14.5525200
C	4.1414981	23.0229192	12.5784870
H	3.4553315	23.8547585	12.7413248
C	4.7781478	22.8765830	11.3252209
H	4.5835551	23.5966263	10.5299773
C	5.6404416	21.8240997	11.1114224
H	6.1335261	21.6991612	10.1462086
C	5.9060156	20.8806787	12.1340447
C	6.7767749	19.7832192	11.9303675
H	7.2546906	19.6640426	10.9568783
C	7.0267117	18.8865055	12.9424874
H	7.7034081	18.0449402	12.8002974
C	6.4110944	19.0316950	14.2077686
C	5.5104680	20.0818501	14.4329218
C	7.2404253	18.4591551	16.3519889
C	8.0281147	19.6434590	16.3826038
H	8.0884390	20.2300005	15.4696966
C	8.6781050	20.0659346	17.5192353
C	8.5977187	19.3256454	18.7133179
C	7.8562242	18.1647609	18.7243429
H	7.7758923	17.5784947	19.6367615
C	7.1660419	17.7055999	17.5758711
C	5.2586827	14.6278064	17.2390689
H	4.1988423	14.4205598	17.0498615
C	5.5106218	14.7415002	18.7674262
C	6.4101062	16.4995567	17.6889905
C	6.0922885	13.5930121	16.5179843
C	7.2092002	13.9594211	15.7647713
H	7.4586800	15.0151023	15.6649947
C	7.9857504	12.9881904	15.1338689
H	8.8526931	13.2894813	14.5446649
C	7.6625997	11.6380469	15.2645347
H	8.2734892	10.8769288	14.7783970
C	6.5438401	11.2683116	16.0115153
H	6.2667297	10.2177741	16.0977103
C	5.7585633	12.2409822	16.6211985
H	4.8633720	11.9502549	17.1674391
C	6.0595102	13.5227874	19.4700178
H	5.3188097	12.7168224	19.4581114
H	6.2823041	13.7771056	20.5128577
H	6.9703235	13.1697972	18.9786903
C	4.2677748	15.2946821	19.4513791
H	3.9224764	16.1920331	18.9261965
H	4.4979620	15.5546227	20.4907435
H	3.4697152	14.5423222	19.4402701
H	9.1051929	19.6655995	19.6147677
H	9.2525623	20.9925561	17.4898903
H	1.0305433	20.9914716	12.6523527
H	1.1794368	19.6640736	10.5278681

Alkylazide (E_{tot}=-553.11288)

H	-0.9803264	-0.5478401	0.3005760
C	-0.5629162	-0.3142352	1.2814884
C	0.5048029	0.2873959	3.7785296
C	0.2301759	-1.2666022	1.9303374
C	-0.8210151	0.9236959	1.8684942
C	-0.2879719	1.2284078	3.1211446
C	0.7589477	-0.9485999	3.1858181
H	-1.4376732	1.6548380	1.3445885
H	-0.4855005	2.1969161	3.5809384
H	1.3811177	-1.6812047	3.7026650
H	0.9301522	0.5184893	4.7557916
C	0.4578668	-2.6214923	1.3214832
H	1.4405819	-3.0099339	1.6252841
H	0.4674260	-2.5443414	0.2248165
C	-0.6238643	-3.6205293	1.7411388
H	-0.6376199	-3.6896696	2.8387695
H	-1.6067308	-3.2268449	1.4429939
C	-0.4079692	-4.9994586	1.1342144
H	0.5721753	-5.3946710	1.4364937
H	-0.3993420	-4.9309178	0.0372429
C	-1.4901426	-5.9725991	1.5631296
H	-1.5013810	-6.0655870	2.6623251
H	-2.4802999	-5.5987771	1.2518295
N	-1.2366207	-7.2880420	0.9518743

N -2.0440003 -8.1673886 1.2207469

N -2.7298724 -9.0650081 1.3992857

TS1(quintet) (E_{tot}=-4271.0289)

Fe 6.3780535 13.4305930 22.2386313

C 4.8510373 16.4115692 19.4485732

H 4.8586028 15.8648908 18.5110897

O 8.5790972 10.1014195 20.9660929

O 7.0277768 17.3858778 23.4899068

N 6.1961543 14.6611743 20.4791767

N 7.7112144 11.9708030 21.8415084

N 4.9569412 12.3462475 21.3398735

N 7.1065646 15.2056144 22.9861684

C 3.8188140 9.1700852 19.9038602

C 4.8577147 17.8621003 21.8159323

H 4.9093922 18.4322919 22.7418484

C 4.1234876 17.5769502 19.5568947

H 3.5533338 17.9211940 18.6935495

C 1.3878909 15.4894569 17.5112491

H 1.2751854 15.7967917 16.4709547

C 2.4690588 14.7244514 17.8851566

H 3.2161392 14.4266496 17.1531186

C 4.0909562 18.3073831 20.7574802

H 3.4987382 19.2169983 20.8424794

C 11.9483334 15.1614602 22.7061246

H 12.9290527 15.0949863 23.1779977

C 5.0193008 8.4463602 19.7814063

H 5.0233607 7.4399299 19.3666161

C 6.1925939 9.0334976 20.2037409

H 7.1315360 8.4912137 20.1218328

C 0.4222729 15.8835186 18.4657191

H -0.4292739 16.4904399 18.1572606

C 3.8118964 10.4418357 20.4254851

H 2.8803116 10.9945680 20.5123734

C 6.2308504 11.3855579 15.8766126

H 7.1710578 11.2410445 15.3421470

C 4.9848889 12.4567363 17.7036429

C 6.2143780 12.2533805 16.9962162

C 5.5559563 15.8521640 20.5499774

C 3.8305258 11.7565455 17.2699892

H 2.9052541 11.8956247 17.8241643

C 9.9144585 12.1518559 24.6919900

H 10.4516361 13.0387311 24.3679583

C 9.3193013 10.5742095 26.4210064

H 9.3761204 10.2375178 27.4562790

C 3.7534783 13.5459620 19.6576155

C 0.5635118 15.5060492 19.7826333

H -0.1692746 15.8156984 20.5291110

C 2.6359478 14.3089563 19.2308335

C 10.5758178 15.2302047 20.7259591

H 10.4802827 15.2109480 19.6403436

C 7.3371154 13.7235986 18.5643945

H 8.2316137 14.2278594 18.9228292

C 4.9572227 13.3146902 18.8332909

C 6.5482272 16.3665249 22.7627467

C 9.7316273 10.9307699 21.3146185

C 9.1502657 11.4507012 23.7602804

C 2.9339900 13.6110976 21.9530883

H 3.1049782 13.3674892 22.9965554

C 1.6644409 14.7196742 20.2015716

C 1.8637860 14.3824997 21.5617582

H 1.1537628 14.7494727 22.3037789

C 7.3787616 12.9144699 17.4524021

H 8.3156079 12.7657319 16.9135150

C 9.9974899 11.7229848 26.0165984

H 10.5919705 12.2890088 26.7348208

C 3.8795721 10.9169959 16.1807632

H 2.9818466 10.3843717 15.8652583

C 9.5368225 15.3320550 22.9089322

C 11.8366273 15.1463347 21.3146439

H 12.7296729 15.0657326 20.6945570

C 3.8547340 13.1433440 20.9900722

C 8.0586336 16.8583504 24.3764657

C 7.4841704 10.8530962 21.1886948

C 8.5693006 9.8546234 25.4890267

H 8.0392330 8.9515698 25.7923933

C 5.5954807 16.6649449 21.7360458

C 8.4871128 10.2905836 24.1704880

H 7.9061965 9.7197756 23.4512105

C 5.0042817 11.0897023 20.8638475

C 5.0885510 10.7307167 15.4722777

H 5.1147590 10.0636487 14.6102711

C 6.2209498 10.3340475 20.7585039

C 10.8199266 10.0339343 21.8545424

H 11.1774489 9.3660079 21.0625051

H 10.4591246 9.4402271 22.6980291

H 11.6562109 10.6533162 22.1980245

C 9.0974865 11.9138610 22.3236833

H 9.5630304 12.8975724 22.2344528

C 6.1361941 13.9283802 19.2836289

C 9.4310249 15.3227281 21.5163536

H 8.4386027 15.3369837 21.0650141

C 7.4960996 16.7802653 25.7833496

H 6.6370174 16.1079294 25.8168291

H 7.1832342 17.7766425 26.1164147

H 8.2711968 16.4082190 26.4648103

C 8.3025489 15.4330672 23.7796082

H 8.3678319 14.6925617 24.5866057

C 10.8044474 15.2585693 23.4939369

H 10.8946057 15.2723688 24.5800471

C 10.1656712 11.6635767 20.0567294

H 11.0177843 12.3120410 20.2848705

H 9.3440468 12.2770306 19.6793622

H 10.4542805 10.9390914 19.2868903

C 9.2181418 17.8293609 24.2923974

H 9.5976769 17.8971576 23.2690184

H 10.0326508 17.5082974 24.9488843

H 8.8761246 18.8181306 24.6189042

H 2.8768114 8.7176969 19.5912176

H 2.2238108 9.3006397 23.0261416

C 2.6114261 8.9072387 23.9768816

H 1.9431943 9.2653218 24.7724126

C 4.0154981 9.4757132 24.1893499

H 4.6729049 9.0719774 23.4069838

H 4.4137364 9.1185765 25.1507626

C 4.0503882 10.9949500 24.1234089

H 3.4496558 11.4379049 24.9298329

H 3.6150064 11.3112364 23.1728016

C 5.4846701 11.5053562 24.1939344

H 6.0707948 10.9510953 23.4562227

H 5.9271867 11.2680436 25.1738361

N 5.7049362 12.8936994 23.8717004

C 2.6138093 7.4050679 23.9357197

C 2.7061495 4.5937181 23.8527491

C 3.1524561 6.7378907 22.8277875

C 2.1269225 6.6411261 25.0003360

C 2.1702087 5.2466576 24.9620363

C 3.1979202 5.3463508 22.7844112

H 3.5432889 7.3228523 21.9961958

H 1.7049453 7.1471981 25.8703725

H 1.7815156 4.6685368 25.8013962

H 3.6181242 4.8448931 21.9113566

H 2.7386509 3.5043638 23.8191986

N 4.7531357 14.8605661 25.0765734

N 5.0189114 13.7500033 25.0542072

Triplet:

complex(triplet) (E_{tot}=-3717.92139)

Fe 7.2600559 13.6600835 20.6989806

C 7.0333234 17.2574052 18.4651001

H 6.7421567 16.8539357 17.4981246

O 9.6001338 10.5800002 19.5608871

O 7.2684808 16.7929781 23.2419036

N 6.9871668 15.0321690 19.4399115

N 8.4869535 12.4781083 19.9778456

N 5.9849456 12.2984560 20.8236993

N 7.0667949 14.8610355 22.1247954

C 5.1960956 8.7163268 21.0685706

C 7.6977646 18.3196887 20.9601448

H 7.9196823 18.7255158 21.9452484

C 7.2896713 18.6047127 18.6088219

H 7.2047695 19.2577922 17.7387447

C 1.8228015 16.2083244 18.6573275

H 1.5598384 16.8192556 17.7931677

C 2.9329192 15.3948396 18.6076514

H 3.5505978 15.3559359 17.7127961

C 7.6580604 19.1473244 19.8569823

H 7.8760286 20.2096871 19.9554084

C 6.4017648 11.2250471 25.2527718

H 5.7323692 10.7232534 25.9520756

C 6.4580646 8.1341602 20.8301487

H 6.5819402 7.0524508 20.8564490

C 7.5260415 8.9530693 20.5272990

H 8.4981955 8.5257524 20.2882435

C 1.0232965 16.2620267 19.8210026

H 0.1477736 16.9111478 19.8492517

C 5.0271206 10.0842639 21.0493380

H 4.0468878 10.5203879 21.2275782

C 5.1119100 11.9787401 15.0926705

H 5.7017649 11.9337315 14.1758793

C 4.8106863 12.8646445 17.3666449

C 5.5722795 12.7953538 16.1539925

C 7.1514974 16.3599151 19.5623995

C 3.6184056 12.1014442 17.4576078

H 3.0436172 12.1457531 18.3801249

C 10.7723628 14.9837756 20.4232393

H 10.4026117 15.5449853 19.5652299

C 11.8533242 14.9401311 22.5825330

H 12.3493254 15.4596779 23.4022920

C 4.4473022 13.7507485 19.6888441

C 1.3551982 15.4971816 20.9181985

H 0.7465438 15.5336394 21.8230772

C 3.2990964 14.5905746 19.7174201

C 8.3448059 11.1948348 23.8252590

H 9.1963297 10.6668897 23.3984996

C 7.2119913 14.3027608 17.1071821

H 8.1419756 14.8642914 17.0444658

C 5.2684092 13.6694359 18.4485215

C 7.2716832 16.1635195 22.0464591

C 10.2937818 11.6071824 18.7878657

C 10.5528847 13.6068166 20.4899659

C 3.9624998 13.0590844 21.9871230

H 4.2687669 12.4673355 22.8459831

C 2.4887071 14.6489733 20.8994997

C 2.8485443 13.8610204 22.0211025

H 2.2278051 13.9076421 22.9175545

C 6.7726728 13.5395271 16.0540825

H 7.3525553 13.4841575 15.1316795

C 11.4177338 15.6502061 21.4649601

H 11.5632604 16.7291231 21.4087293

C 3.1964078 11.3176963 16.4068864

H 2.2780850 10.7371608 16.4995157

C 6.9774766 13.1874772 23.9527300

C 7.5005723 10.5459148 24.7258053

H 7.6890070 9.5093401 25.0050464

C 4.7787490 13.0015024 20.8270940

C 7.2861894 15.7454033 24.2646895

C 8.4717768 11.1619500 20.0331466

C 11.6218518 13.5648485 22.6636629

H 11.9304298 13.0095267 23.5495976

C 7.4226771 16.9372014 20.8564253

C 10.9687529 12.9062654 21.6272733

H 10.7426822 11.8450411 21.7059940

C 6.1193611 10.9627028 20.8120032

C 3.9464967 11.2538011 15.2112769

H 3.6033366 10.6278524 14.3872335

C 7.3905223 10.3589413 20.4962174

C 11.7817750 11.3659306 18.8822020

H 12.0306044 10.4037266 18.4194620

H 12.1252621 11.3670862 19.9189439

H 12.3099852 12.1612519 18.3440217

C 9.7583066 12.9199650 19.4110600

H 9.5443592 13.6416762 18.6157337

C 6.4753752 14.3729390 18.3186895

C 8.0897727 12.5102313 23.4502145

H 8.7423937 13.0130806 22.7404747

C 6.4579651 16.2117075 25.4379902

H 5.4456485 16.4747554 25.1108016

H 6.9191833 17.0850641 25.9138271

H 6.3976517 15.4018678 26.1743048

C 6.6467546 14.5735382 23.4942099

H 5.5521149 14.6928808 23.5287653

C 6.1403650 12.5377601 24.8617896

H 5.2602371 13.0563777 25.2438635

C 9.7919721 11.4982989 17.3544984

H 10.3005084 12.2388466 16.7253199

H 8.7144682 11.6896758 17.3177200

H 9.9964041 10.4951624 16.9636871

C 8.7344951 15.4843012 24.6303108

H 9.2936856 15.1590456 23.7492547

H 8.7923432 14.6972049 25.3893189

H 9.1836897 16.4036874 25.0224817

H 4.3352777 8.0770815 21.2710999

TS1(triplet) (E_{tot}=-4271.02573)

Fe 6.2095219 13.3707781 22.2491072

C 4.8938173 16.3827252 19.6946609

H 4.8928054 15.9083343 18.7187400

O 8.4580866 9.9446770 21.2020838

O 7.1656600 17.0115208 23.7189110

N 6.1810809 14.5276751 20.6130439

N 7.6563602 11.9304628 21.8831613

N 4.9156880 12.3009429 21.3151911

N 7.1966773 14.8683562 23.0559484

C 3.7324375 9.0571412 20.0794941

C 4.9218191 17.6391360 22.1796685

H 4.9860281 18.1339451 23.1471003

C 4.1910919 17.5531120 19.8988764

H 3.6294641 17.9786651 19.0666733

C 1.5526844 15.5328147 17.3584168

H 1.5087904 15.8780489 16.3248761

C 2.6110515 14.7578496 17.7762410

H 3.4060727 14.4902267 17.0842547

C 4.1679094 18.1815066 21.1553570

H 3.5921544 19.0921814 21.3116766

C 11.9644705 15.1945611 22.1444423

H 12.9996800 15.0923587 22.4722729

C 4.8942657 8.2686661 20.1559246
H 4.8753479 7.2186614 19.8693980
C 6.0629236 8.8515677 20.6004869
H 6.9750096 8.2625982 20.6549115
C 0.5218549 15.8864788 18.2586156
H -0.3120639 16.4988890 17.9149299
C 3.7534356 10.3812781 20.4507507
H 2.8485134 10.9803886 20.3960047
C 6.4304124 11.5025003 15.8459292
H 7.3948869 11.3766694 15.3518044
C 5.1002828 12.4922533 17.6589700
C 6.3633466 12.3126828 17.0064642
C 5.5708749 15.7297658 20.7546603
C 3.9650091 11.8199500 17.1389698
H 3.0140334 11.9380939 17.6531831
C 10.7260635 12.2086557 24.0601902
H 11.5017550 12.4009113 23.3233550
C 10.0337088 12.0502626 26.3707764
H 10.2727566 12.0932790 27.4335871
C 3.7805265 13.5192764 19.5893438
C 0.5785577 15.4646039 19.5684766
H -0.2032538 15.7456378 20.2754148
C 2.6898267 14.2943697 19.1142858
C 10.3479091 15.5806755 20.3993394
H 10.1120094 15.7819092 19.3540144
C 7.4210727 13.6690033 18.7182060
H 8.3074496 14.1067126 19.1664792
C 5.0227027 13.2982936 18.8234725
C 6.6286842 16.0515543 22.9610092
C 9.6460135 10.7671577 21.3519690
C 9.4173747 11.9648288 23.6334683
C 2.8329782 13.5336907 21.8300269
H 2.9573358 13.2786003 22.8765758
C 1.6569575 14.6723635 20.0327370
C 1.7772208 14.3002637 21.3925517
H 1.0202055 14.6416321 22.0995525
C 7.5090965 12.9272609 17.5617759
H 8.4734372 12.7860132 17.0718803
C 11.0345348 12.2480305 25.4169848
H 12.0594399 12.4458812 25.7332506
C 4.0647307 11.0325104 16.0148416
H 3.1825070 10.5188479 15.6317879
C 9.5972267 15.1853318 22.6659966
C 11.6764224 15.4504688 20.8027304
H 12.4841843 15.5469661 20.0768039
C 3.8166562 13.0975534 20.9163181
C 8.2745856 16.4179225 24.4676622
C 7.3952401 10.7485994 21.3826937
C 8.7272145 11.8050006 25.9511682
H 7.9314990 11.6639379 26.6828360
C 5.6327542 16.4391453 22.0020315
C 8.4286876 11.7573805 24.5907118
H 7.4128400 11.6172512 24.2504986
C 4.9381462 11.0123383 20.9311620
C 5.3060088 10.8765121 15.3558692
H 5.3711756 10.2522603 14.4643813
C 6.1204305 10.2075057 20.9928545
C 10.6948013 9.8991438 22.0089511
H 10.8532838 9.0084894 21.3895451
H 10.3802640 9.5944774 23.0111314
H 11.6432758 10.4384256 22.0865232
C 9.0951054 11.9776610 22.1600232
H 9.5039081 12.9024341 21.7398753
C 6.1822861 13.8601882 19.3697785
C 9.3137877 15.4492023 21.3254716
H 8.2743255 15.4997632 21.0087029
C 7.8409570 16.1635696 25.8985775
H 7.0475327 15.4179205 25.9408225
H 7.4818339 17.0942469 26.3522663
H 8.7025415 15.7986376 26.4706620
C 8.4966428 15.0825815 23.6961488
H 8.7205883 14.2753319 24.3970757
C 10.9301298 15.0688090 23.0677813
H 11.1535367 14.8614412 24.1115444
C 10.0640572 11.2187141 19.9614761
H 10.9433213 11.8697886 20.0350653
H 9.2480176 11.7768115 19.4933228
H 10.3082732 10.3504159 19.3386625
C 9.4015364 17.4273083 24.4062961
H 9.6712424 17.6500170 23.3705418
H 10.2842443 17.0380859 24.9230323
H 9.0799338 18.3486364 24.9057131
H 2.7961560 8.6160549 19.7347379
H 1.8979899 9.1432103 22.9070862
C 2.2421679 8.8277860 23.9026364
H 1.5415026 9.2498071 24.6366697
C 3.6377234 9.4116539 24.1300440
H 4.3238884 8.9692189 23.3952619
H 4.0025991 9.1139078 25.1242721
C 3.6646769 10.9244580 23.9776210
H 3.0284192 11.4102717 24.7310419
H 3.2631616 11.1792032 22.9937297
C 5.0911689 11.4557118 24.0761458
H 5.7203177 10.8565586 23.4050298
H 5.4726642 11.2807283 25.0931166
N 5.2431136 12.8512414 23.7208399
C 2.2385654 7.3273759 23.9861561
C 2.3186474 4.5188784 24.1448371
C 2.8166670 6.5676503 22.9611070
C 1.7058842 6.6571849 25.0913284
C 1.7429402 5.2644803 25.1728053
C 2.8562622 5.1773171 23.0372034
H 3.2448335 7.0801508 22.1007291
H 1.2535259 7.2359351 25.8985420
H 1.3187498 4.7603884 26.0421451
H 3.3087118 4.6025902 22.2277397
H 2.3472358 3.4305659 24.2050174
N 5.0834560 13.7171205 25.1160803
N 4.8423882 14.8255868 25.2248142

OSS

complex(OSS) (E_{tot}=-3717.91715)

Fe 6.9010978 13.5139105 20.5766598
C 7.2949094 16.8706312 18.0002556
H 6.7033623 16.5422504 17.1506980
O 9.9408223 11.1152061 19.5653581
O 7.9415009 16.7830787 22.7170716
N 6.8164643 14.7974222 19.2165816
N 8.4721672 12.6701894 20.2619946
N 6.0208445 11.9328133 20.4235736
N 7.2166502 14.8720687 21.7934351
C 5.9039427 8.2574526 20.5716244
C 8.6641710 17.8573670 20.2140285
H 9.1359115 18.2503590 21.1131313
C 7.9643101 18.0780216 17.9352596
H 7.9054632 18.6626299 17.0157659
C 1.6026345 16.0048749 19.4335809
H 1.2458559 16.7569705 18.7292675
C 2.7016390 15.2344271 19.1149272
H 3.2182915 15.3677802 18.1663034
C 8.6976123 18.5695873 19.0300148
H 9.2310268 19.5164736 18.9641175
C 5.4207693 12.0111326 25.3023691

H	4.6302521	11.8340505	26.0320976
C	7.2591750	7.9517028	20.3455492
H	7.5879953	6.9139004	20.3078426
C	8.1697009	8.9761111	20.1583582
H	9.2193477	8.7587110	19.9690663
C	0.9399430	15.8305927	20.6663813
H	0.0746783	16.4469605	20.9110379
C	5.4690241	9.5695853	20.6082266
H	4.4173741	9.8075798	20.7530292
C	4.2844930	12.1539087	14.9115952
H	4.7636561	12.1526112	13.9313883
C	4.3166595	12.8429987	17.2730070
C	4.9267154	12.8303634	15.9762943
C	7.3543173	16.0419322	19.1559128
C	3.0881184	12.1524490	17.4410435
H	2.6244659	12.1448597	18.4249441
C	11.0240075	15.0462028	21.3469714
H	10.7968248	15.7991930	20.5935402
C	12.1149188	14.4018134	23.4039885
H	12.7485980	14.6490999	24.2557015
C	4.3205933	13.4496069	19.6980461
C	1.3909752	14.8803535	21.5615139
H	0.8851951	14.7387299	22.5179821
C	3.1854844	14.2505389	20.0115798
C	7.2516640	11.1958760	23.9569492
H	7.8911923	10.3799219	23.6223609
C	6.7797628	14.1248970	16.8500227
H	7.7544423	14.5883695	16.7235972
C	4.9523768	13.5227551	18.3532419
C	7.7488525	16.0522379	21.5983490
C	10.4531239	12.3907170	19.0680031
C	10.4763805	13.7682622	21.2215775
C	4.0920265	12.3355535	21.8910282
H	4.5124409	11.6383002	22.6104231
C	2.5099278	14.0709942	21.2627436
C	2.9886406	13.0914255	22.1768352
H	2.4792214	12.9749973	23.1343075
C	6.1766980	13.4726456	15.8047265
H	6.6666645	13.4240776	14.8311266
C	11.8408726	15.3622767	22.4313268
H	12.2550408	16.3667395	22.5220549
C	2.4831477	11.5098664	16.3832042
H	1.5358180	10.9917974	16.5360937
C	6.5848048	13.5188318	23.8051738
C	6.2455317	10.9610452	24.8936606
H	6.0985435	9.9601340	25.3001934
C	4.7849230	12.5183605	20.6586504
C	7.6112724	15.9055822	23.8472806
C	8.6987024	11.3855100	20.0317438
C	11.5521830	13.1290292	23.2958451
H	11.7455242	12.3787654	24.0628277
C	7.9869849	16.6264461	20.3126335
C	10.7375094	12.8154027	22.2111470
H	10.3125640	11.8187727	22.1147760
C	6.3817119	10.6345733	20.4391235
C	3.0823062	11.5087789	15.1043183
H	2.5971223	10.9915987	14.2762063
C	7.7637343	10.3244513	20.2201979
C	11.9586957	12.3713336	19.1694861
H	12.3635960	11.6034140	18.5000814
H	12.2798813	12.1683057	20.1943330
H	12.3545445	13.3476909	18.8682535
C	9.7161091	13.4025077	19.9799036
H	9.4557574	14.3079328	19.4257501
C	6.1706346	14.1855841	18.1324517
C	7.4300282	12.4732087	23.4290832
H	8.2134559	12.6670797	22.7054364
C	6.8996542	16.7332372	24.8913336
H	6.0204067	17.2249818	24.4595857
H	7.5723797	17.4964648	25.2998509
H	6.5788422	16.0754151	25.7074323
C	6.6939312	14.8679667	23.1685578
H	5.6772516	15.2913467	23.1146565
C	5.5827979	13.2797006	24.7479669
H	4.9014426	14.0853845	25.0216138
C	9.9728204	12.5476225	17.6338916
H	10.3333272	13.4987355	17.2244454
H	8.8792020	12.5429708	17.6018111
H	10.3555769	11.7245403	17.0200653
C	8.8958381	15.2899746	24.3541563
H	9.3622774	14.6973784	23.5664311
H	8.6833749	14.6345075	25.2051331
H	9.5890524	16.0785583	24.6628650
H	5.1824503	7.4506630	20.7065596

Quintet:

nitrene(quintet) (E_{tot}=-4161.56113)

Fe	6.7600126	13.1400652	21.9840596
C	5.9630117	16.2484406	18.9288269
H	5.9571020	15.6187555	18.0443399
O	8.0573313	9.7866678	19.8146602
O	6.5561807	16.9438836	23.6350457
N	6.6088610	14.3295770	20.2624832
N	7.7802565	11.7040652	20.9397019
N	5.0728621	12.3266487	21.4642627
N	6.9905472	14.8522655	22.9699593
C	3.2027114	9.3045708	20.5555049
C	5.9142341	17.8816763	21.1664460
H	5.8957633	18.5053831	22.0570017
C	5.6083885	17.5733622	18.8166022
H	5.3367073	17.9654716	17.8353408
C	1.8447519	16.2124989	18.0552640
H	1.6956926	16.5686429	17.0354051
C	2.7677487	15.2237013	18.3073382
H	3.3544792	14.7911021	17.5006078
C	5.5757089	18.4153242	19.9419585
H	5.2837013	19.4603120	19.8524037
C	10.7602829	14.7900591	26.0753369
H	11.1746445	14.4604029	27.0287776
C	4.2305743	8.4152631	20.2048816
H	4.0043067	7.3938132	19.9039106
C	5.5384805	8.8610569	20.2357611
H	6.3463016	8.1968204	19.9382056
C	1.0911352	16.7770867	19.1097413
H	0.3643205	17.5613578	18.8968780
C	3.4933510	10.5891059	20.9617035
H	2.6963196	11.2725018	21.2448805
C	5.1601951	11.0571760	15.8805047
H	5.9272530	10.7292331	15.1773496
C	4.5342574	12.3629587	17.8642237
C	5.5335174	11.9262911	16.9352384
C	6.3085674	15.6480301	20.1781933
C	3.2031679	11.9054268	17.6974345
H	2.4518552	12.2335975	18.4122177
C	11.3931559	12.7424686	20.9054031
H	11.4940498	12.9065723	19.8318299
C	12.3263445	12.8176890	23.1286254
H	13.1499339	13.0548626	23.8008192
C	3.9268278	13.7352781	19.9218796
C	1.2790890	16.3406329	20.4023052
H	0.7072338	16.7767087	21.2226576
C	2.9759262	14.7441728	19.6248566
C	11.0410270	15.8172218	23.9127430

H 11.6800224 16.2851493 23.1642846
C 7.1958775 13.2016402 18.1554382
H 8.2018549 13.6038648 18.2532493
C 4.9009489 13.2166882 18.9362430
C 6.6163948 16.0615239 22.6211095
C 9.4085441 10.3063207 19.9546837
C 10.1952968 12.2330398 21.4155876
C 3.3645570 13.8901779 22.2909364
H 3.5446078 13.5332832 23.3024142
C 2.2194126 15.3228318 20.6976282
C 2.4467789 14.8806892 22.0240909
H 1.8776350 15.3378357 22.8345949
C 6.8593400 12.3921213 17.0945025
H 7.6103600 12.1082805 16.3560614
C 12.4585272 13.0284852 21.7565107
H 13.3894085 13.4238533 21.3486247
C 2.8728094 11.0585472 16.6641027
H 1.8455252 10.7094875 16.5552857
C 8.8679454 14.9859705 24.5727814
C 11.5744314 15.4293248 25.1410402
H 12.6281001 15.6023679 25.3599573
C 4.0948967 13.3036173 21.2347659
C 6.6336041 16.1644052 24.8657969
C 7.2386496 10.6031559 20.5014988
C 11.1193753 12.3487780 23.6429835
H 10.9942620 12.2373857 24.7177309
C 6.2873725 16.5258593 21.3131172
C 10.0578027 12.0575808 22.7921814
H 9.0966823 11.7300544 23.1818097
C 4.8281398 11.0673389 21.0384175
C 3.8580057 10.6294978 15.7454259
H 3.5837892 9.9579577 14.9314426
C 5.8659188 10.1746631 20.6287884
C 10.1129769 9.4302751 20.9729594
H 10.1145018 8.3938887 20.6164237
H 9.5945094 9.4827434 21.9355084
H 11.1426801 9.7705060 21.1189517
C 9.1510825 11.7621813 20.4488972
H 9.1617398 12.4309783 19.5801834
C 6.2391467 13.5761166 19.1242144
C 9.6954484 15.5967842 23.6300375
H 9.2796960 15.8984989 22.6709003
C 5.2109641 15.8130573 25.2722972
H 4.7365948 15.2220947 24.4833377
H 4.6291982 16.7273207 25.4362664
H 5.2260452 15.2262777 26.1984981
C 7.3780336 14.8988958 24.3771121
H 6.9934587 13.9987640 24.8661447
C 9.4164783 14.5647014 25.7865280
H 8.7755999 14.0706933 26.5180723
C 10.0758603 10.2693968 18.5981407
H 11.0737640 10.7147703 18.6801734
H 9.4841431 10.8366779 17.8724230
H 10.1815428 9.2356619 18.2485163
C 7.3378726 17.0100175 25.8992276
H 8.3275411 17.3090964 25.5433598
H 7.4618596 16.4345933 26.8225315
H 6.7380978 17.9020619 26.1147733
H 2.1631448 8.9758170 20.5264547
H 2.5592220 9.6680372 23.9464079
C 3.1809137 9.1138111 24.6647886
H 3.0119888 9.5640541 25.6531828
C 4.6459811 9.2935475 24.2637550
H 4.7868950 8.8850775 23.2544287
H 5.2830013 8.7025775 24.9388881
C 5.0775553 10.7506555 24.2681521
H 4.9685954 11.1881553 25.2713396
H 4.4398852 11.3247722 23.5907098
C 6.5289274 10.9122956 23.7928237
H 6.6952514 10.2141249 22.9515050
H 7.2107666 10.5672234 24.5974689
N 6.9000067 12.2188586 23.4213720
C 2.7692747 7.6682841 24.6842908
C 2.0702497 4.9411002 24.6952526
C 2.6708670 6.9479376 23.4870312
C 2.5160065 6.9977822 25.8847809
C 2.1681535 5.6462249 25.8940340
C 2.3239245 5.5988618 23.4900918
H 2.8783317 7.4559757 22.5470892
H 2.5907347 7.5443775 26.8265135
H 1.9719134 5.1430349 26.8417163
H 2.2502153 5.0558986 22.5466749
H 1.7973117 3.8854825 24.6991132

TS2-proS(boat)quintet (E_{tot}=-4161.54083)

Fe 6.4408461 12.3939403 21.7020894
C 5.5633133 15.8412670 19.0750507
H 5.6941605 15.3597830 18.1106692
O 8.8021820 10.3050434 19.0208516
O 5.5322122 15.8156739 23.8596046
N 6.2412340 13.7880485 20.1708810
N 7.9331484 11.5780111 20.6408917
N 5.0548687 11.3547375 20.8118261
N 6.1953731 13.8683347 22.9822677
C 4.2300388 8.5041168 18.6626868
C 5.1744121 17.0989902 21.5126891
H 5.0136486 17.5722063 22.4782500
C 5.1353130 17.1483719 19.1238736
H 4.9419950 17.6758171 18.1881523
C 1.3678284 15.4520090 18.1956120
H 1.2094750 16.0389815 17.2902023
C 2.4104229 14.5556524 18.2573278
H 3.0821957 14.4232242 17.4124091
C 4.9343304 17.8019812 20.3523890
H 4.5864333 18.8330664 20.3894354
C 9.3753025 13.6336081 26.6917395
H 9.6659807 13.1724557 27.6365725
C 5.4970235 8.0602277 18.2503219
H 5.5987651 7.1974918 17.5939032
C 6.6150146 8.7518717 18.6731575
H 7.6018047 8.4405534 18.3387766
C 0.5037451 15.6258579 19.3008914
H -0.3168969 16.3412029 19.2399192
C 4.1051640 9.5902585 19.5010657
H 3.1236415 9.9370175 19.8169638
C 5.5065693 11.2798666 15.1432036
H 6.3584844 11.1602534 14.4720884
C 4.5939830 12.1055439 17.2688131
C 5.7034121 11.9477799 16.3772971
C 5.8304434 15.0760225 20.2524100
C 3.3327794 11.5847372 16.8843865
H 2.4959832 11.7000796 17.5699560
C 11.2186427 13.2473979 21.4768357
H 11.4073893 13.7313430 20.5182734
C 11.8917498 12.7732598 23.7449016
H 12.5967335 12.8932883 24.5668946
C 3.7139032 12.8704927 19.5288399
C 0.7044883 14.8996288 20.4544293
H 0.0493271 15.0367174 21.3160776
C 2.6357418 13.7837547 19.4252585
C 9.8536715 15.1107481 24.8478826
H 10.5282038 15.7970111 24.3373336
C 7.1224720 13.0897063 17.9766786

H 8.0772940 13.5298009 18.2463210

C 4.7876773 12.7451062 18.5204922

C 5.7973528 15.1031265 22.7542420

C 9.9427549 11.0347634 19.5476591

C 10.0738795 12.4600231 21.6398476

C 3.0765953 12.3688573 21.8297665

H 3.3041245 11.8390186 22.7494668

C 1.7685134 13.9700482 20.5534707

C 2.0259528 13.2526926 21.7479652

H 1.3840491 13.4228084 22.6131319

C 6.9604511 12.4684431 16.7596358

H 7.8023834 12.3745782 16.0726563

C 12.1281166 13.3988535 22.5210152

H 13.0211346 14.0085891 22.3791468

C 3.1728175 10.9428056 15.6777213

H 2.1965842 10.5441132 15.3996445

C 7.7619894 13.8974934 24.9027964

C 10.2226180 14.5497007 26.0699389

H 11.1802044 14.8061768 26.5231563

C 3.9151491 12.1565290 20.7113822

C 5.4266086 14.8537170 24.9526657

C 7.7358953 10.6142439 19.7798565

C 10.7191113 12.0451111 23.9303708

H 10.4905092 11.6196170 24.9049859

C 5.6196806 15.7559674 21.4967900

C 9.8105445 11.8964504 22.8871176

H 8.8712690 11.3782840 23.0444980

C 5.2350285 10.3054345 19.9799840

C 4.2685980 10.7855732 14.7980301

H 4.1299978 10.2710826 13.8466076

C 6.5239551 9.8786998 19.5187791

C 10.7587186 10.0451772 20.3597973

H 11.0528835 9.2089727 19.7153962

H 10.1627641 9.6672266 21.1967843

H 11.6517324 10.5288769 20.7666940

C 9.2606290 12.1338112 20.4207483

H 9.1472152 13.0546981 19.8325259

C 6.0549324 13.2029277 18.8930911

C 8.6335517 14.7819284 24.2648214

H 8.3476733 15.2203101 23.3112543

C 3.9881227 14.3851483 25.0136959

H 3.7044316 13.9104075 24.0710116

H 3.3258638 15.2385338 25.1995028

H 3.8762528 13.6589776 25.8204964

C 6.3440904 13.7198841 24.4321060

H 5.9592706 12.7496606 24.7473916

C 8.1526462 13.3090831 26.1075801

H 7.4719905 12.6202894 26.6052525

C 10.7194210 11.5978731 18.3785970

H 11.5731169 12.1716134 18.7544451

H 10.0812775 12.2499818 17.7744438

H 11.0978931 10.7846644 17.7478498

C 5.8573953 15.5416236 26.2250257

H 6.8683244 15.9469487 26.1299687

H 5.8451661 14.8166067 27.0451028

H 5.1550403 16.3505482 26.4579533

H 3.3321889 7.9876053 18.3205452

H 3.9686989 9.8727117 23.6309891

C 4.7017399 10.0802077 24.4168698

H 5.5635423 10.9195043 23.6708546

C 5.6284337 8.9016486 24.6825621

H 5.2150963 8.0164196 24.1767910

H 5.6545347 8.6691835 25.7564004

C 7.0504800 9.1163899 24.1583284

H 7.5860032 8.1583571 24.0930828

H 7.6156423 9.7753168 24.8325563

C 6.9849353 9.7789742 22.7848432

H 6.2832644 9.2170273 22.1426952

H 7.9606667 9.7456047 22.2844036

N 6.5615068 11.1402559 22.9534884

C 4.1350808 10.7971690 25.5461711

C 3.0094682 12.1402050 27.7765301

C 4.9075604 11.1046089 26.6879556

C 2.7823500 11.2005778 25.5571971

C 2.2271647 11.8475352 26.6557707

C 4.3572442 11.7682852 27.7781949

H 5.9547585 10.8065619 26.7066966

H 2.1568649 10.9694774 24.6944705

H 1.1739552 12.1310930 26.6389180

H 4.9824295 11.9959982 28.6430775

H 2.5771924 12.6553672 28.6338989

TS2-proR(boat)quintet (E_{tot}=-4161.54497)

Fe 6.4162130 12.3335077 21.6383306

C 5.2162128 15.8848240 19.3427319

H 5.4707652 15.5443493 18.3440231

O 8.7283109 10.1526999 18.9617027

O 5.2594987 15.4451410 24.1063329

N 6.2313758 13.8825818 20.2600520

N 7.8832025 11.5200667 20.5209486

N 5.0125266 11.4144561 20.6486219

N 6.1782523 13.7012658 23.0478884

C 4.1100371 8.4841111 18.6402708

C 4.4915675 16.7637435 21.8680170

H 4.2142888 17.0910876 22.8670522

C 4.5133722 17.0574659 19.4967960

H 4.2346994 17.6250133 18.6080665

C 1.6040354 15.3203421 17.4341818

H 1.5410084 15.8534443 16.4848143

C 2.6650898 14.4782747 17.6766338

H 3.4441317 14.3419285 16.9306625

C 4.1329445 17.5148246 20.7705533

H 3.5632492 18.4349808 20.8888577

C 9.6996317 13.8837384 26.4436271

H 10.1241114 13.4960583 27.3703616

C 5.3659151 7.9551554 18.2982212

H 5.4446718 7.0413297 17.7115472

C 6.5035582 8.6294306 18.6969481

H 7.4836198 8.2582869 18.4067719

C 0.5964355 15.5048192 18.4080633

H -0.2385691 16.1757155 18.2047040

C 4.0142069 9.6332082 19.3942221

H 3.0454978 10.0403751 19.6687810

C 6.3517110 11.7699263 15.0098045

H 7.2693020 11.8386494 14.4234825

C 5.1207934 12.2817144 17.0723265

C 6.3203562 12.3667233 16.2944044

C 5.5846918 15.0592095 20.4477156

C 4.0027379 11.6016363 16.5285504

H 3.0992968 11.5361737 17.1306130

C 11.1876176 13.1573097 21.3199132

H 11.3782462 13.6116499 20.3472965

C 11.8498746 12.7565745 23.6041984

H 12.5483522 12.9061905 24.4264647

C 3.8570422 12.9106434 19.1967589

C 0.6753592 14.8421790 19.6128509

H -0.0916504 14.9860879 20.3753398

C 2.7679866 13.7712479 18.9013245

C 9.8492181 15.3135155 24.5084877

H 10.3976963 16.0414458 23.9114311

C 7.3754766 13.5893039 18.1018923

H 8.2013531 14.1796911 18.4933985

C 5.0805974 12.8576414 18.3683444

C 5.5625850 14.8587311 22.9358384
C 9.8888954 10.8622231 19.4720748
C 10.0441247 12.3723461 21.5017660
C 2.9235475 12.4656009 21.4066758
H 3.0030131 11.9380022 22.3504986
C 1.7538056 13.9687169 19.8961901
C 1.8724512 13.3123776 21.1453057
H 1.1061811 13.4782006 21.9030807
C 7.4252178 13.0687666 16.8284933
H 8.3139157 13.2033778 16.2107009
C 12.0904494 13.3456784 22.3632059
H 12.9801878 13.9569256 22.2084902
C 4.0655918 11.0282121 15.2792412
H 3.1999338 10.4986343 14.8802646
C 7.9125306 13.8974339 24.8061657
C 10.3851201 14.8506812 25.7092984
H 11.3483678 15.2225358 26.0582339
C 3.9040871 12.2400480 20.4175147
C 5.4576969 14.4393432 25.1402400
C 7.6679553 10.5269899 19.6995205
C 10.6836443 12.0221874 23.8034930
H 10.4596661 11.6150503 24.7867716
C 5.2140551 15.5542277 21.7414687
C 9.7797313 11.8381973 22.7615950
H 8.8449631 11.3115664 22.9260241
C 5.1635672 10.3178253 19.8719256
C 5.2495400 11.1118901 14.5107111
H 5.2872433 10.6525907 13.5225373
C 6.4392013 9.8134121 19.4626250
C 10.6707838 9.8792516 20.3232434
H 10.9476238 9.0137192 19.7106987
H 10.0613480 9.5458775 21.1690442
H 11.5734451 10.3539683 20.7194688
C 9.2341622 12.0147728 20.2928668
H 9.1602836 12.9003525 19.6517837
C 6.2346179 13.4280764 18.9184469
C 8.6184336 14.8410530 24.0599323
H 8.1987547 15.2023706 23.1234959
C 4.1424150 13.7048633 25.3240364
H 3.8722323 13.1698639 24.4098400
H 3.3471509 14.4151115 25.5763067
H 4.2414788 12.9781964 26.1368101
C 6.4985137 13.5123687 24.4615272
H 6.3247130 12.4699694 24.7335291
C 8.4745624 13.4049660 25.9867411
H 7.9324935 12.6541870 26.5631362
C 10.6878652 11.3657715 18.2911481
H 11.5516149 11.9319164 18.6559608
H 10.0650596 12.0118685 17.6653160
H 11.0515163 10.5237040 17.6904259
C 5.8943156 15.1606091 26.3928270
H 6.7995766 15.7449284 26.2078571
H 6.1057147 14.4341206 27.1839737
H 5.0893986 15.8248914 26.7286757
H 3.1985992 7.9805187 18.3140885
C 4.8619259 10.0899430 24.5628403
H 5.5972286 10.8146991 23.6112878
C 5.6776095 8.8120734 24.5996670
H 5.1604217 8.0330127 24.0249048
H 5.7479834 8.4400739 25.6348579
C 7.0777231 8.9859795 24.0143935
H 7.5781679 8.0114576 23.9193762
H 7.6958983 9.6229496 24.6631336
C 6.9644556 9.6516295 22.6432118
H 6.2656887 9.0699574 22.0167471
H 7.9317541 9.6301877 22.1250549
N 6.5298379 11.0051047 22.8115199
C 3.5111337 10.0479357 24.0190710
C 0.9051284 10.0401160 22.9058620
C 2.4473418 10.7458522 24.6291240
C 3.2231850 9.3497186 22.8264288
C 1.9432744 9.3390648 22.2887807
C 1.1690047 10.7464102 24.0801685
H 2.6322041 11.2838101 25.5570691
H 4.0246849 8.8455764 22.2936080
H 1.7594240 8.7976736 21.3614898
H 0.3697169 11.2978941 24.5770497
H -0.0932861 10.0455559 22.4697719
H 4.9476316 10.6935161 25.4720673

TS2-proS(chair)quintet (E_{tot}=-4161.552)

Fe 6.6249210 12.3840873 21.5798081
C 6.0891309 16.2031730 19.4644989
H 6.1948627 15.8528458 18.4426327
O 8.8124761 9.5234620 19.6224278
O 5.2086548 15.3853689 24.0753699
N 6.4415356 13.9437391 20.2878711
N 7.9507732 11.4343302 20.4171076
N 5.1817420 11.3880393 20.6610730
N 5.9439477 13.5836849 22.9712528
C 4.1943244 7.9077964 20.0343662
C 5.6936113 17.1421727 22.0448981
H 5.5012716 17.4827416 23.0598539
C 5.8812101 17.5443966 19.6973089
H 5.8419606 18.2263245 18.8460777
C 1.8424526 15.4878205 17.6062421
H 1.8443908 16.1466206 16.7372495
C 2.9225622 14.6668621 17.8437505
H 3.7752618 14.6707345 17.1693779
C 5.7061295 18.0421520 21.0014545
H 5.5484634 19.1044223 21.1799438
C 8.0028013 11.7601555 26.9665625
H 7.9622318 11.0524161 27.7952364
C 5.4390787 7.2522254 20.0840075
H 5.4984313 6.1678975 20.0060181
C 6.5878838 8.0115735 20.1871129
H 7.5639005 7.5317002 20.1558053
C 0.7339073 15.4888564 18.4815941
H -0.1146910 16.1439082 18.2829709
C 4.1098674 9.2779154 20.1571152
H 3.1440835 9.7771861 20.1263832
C 6.4152834 12.1980130 14.9092836
H 7.2994153 12.3545127 14.2892441
C 5.2693751 12.4865969 17.0625561
C 6.4250370 12.6843110 16.2397139
C 6.1770398 15.2479630 20.5242096
C 4.1517878 11.8102840 16.5130423
H 3.2755048 11.6646893 17.1415728
C 10.8253442 13.6728565 19.5527955
H 10.9063400 13.4214599 18.4955572
C 11.4116989 15.0949841 21.4106278
H 11.9666174 15.9401982 21.8177079
C 4.0486817 12.9406284 19.2385192
C 0.7328620 14.6685077 19.5882659
H -0.1127669 14.6685775 20.2777195
C 2.9462658 13.7995226 18.9650711
C 9.2324756 13.3173533 25.5988133
H 10.1634880 13.8312424 25.3628149
C 7.5266535 13.7977837 18.0897968
H 8.3667535 14.3418837 18.5150767
C 5.2653441 12.9729272 18.4002002
C 5.7159116 14.8769432 22.9402414
C 9.9193192 10.4734282 19.5498166

C 9.9545777 12.9371990 20.3646057
C 2.9448131 12.1851663 21.2721800
H 2.9934088 11.5840550 22.1737504
C 1.8279420 13.8129995 19.8614523
C 1.8637879 12.9928709 21.0155116
H 1.0256827 13.0251819 21.7131415
C 7.5329295 13.3815041 16.7790276
H 8.3957603 13.5795423 16.1411447
C 11.5584597 14.7375544 20.0700610
H 12.2283417 15.3026323 19.4214200
C 4.1739072 11.3465060 15.2161705
H 3.3066693 10.8236725 14.8119507
C 6.9023900 12.8813769 25.1214289
C 9.1951395 12.4192992 26.6655089
H 10.0921289 12.2316078 27.2558063
C 4.0381974 12.1404566 20.3754041
C 4.7542262 14.2485682 24.8720541
C 7.7601890 10.1612570 20.1747890
C 10.5368728 14.3737667 22.2214024
H 10.4045485 14.6602429 23.2619579
C 5.9083957 15.7609272 21.8376231
C 9.8198370 13.2954935 21.7056027
H 9.1380711 12.7452245 22.3499634
C 5.2627369 10.0774164 20.3978716
C 5.3141218 11.5412528 14.4047244
H 5.3196451 11.1704852 13.3794237
C 6.5404245 9.4144248 20.3225411
C 10.9183318 10.1469071 20.6411694
H 11.2677964 9.1148987 20.5236299
H 10.4714403 10.2618754 21.6310182
H 11.7719123 10.8293593 20.5700194
C 9.1784099 11.8222935 19.7385009
H 8.8866231 12.1611831 18.7353656
C 6.4148322 13.5512106 18.9313091
C 8.0917213 13.5494559 24.8316438
H 8.1241198 14.2281393 23.9832039
C 3.3007618 13.9863028 24.5168540
H 3.2078836 13.7871667 23.4453964
H 2.6872955 14.8572869 24.7749182
H 2.9405774 13.1099227 25.0667160
C 5.6494975 13.1125568 24.3222747
H 5.0831704 12.1793546 24.2699623
C 6.8654917 11.9971471 26.2001065
H 5.9352398 11.4823678 26.4207542
C 10.5360924 10.3457472 18.1721951
H 11.3347387 11.0879073 18.0633095
H 9.7789962 10.5143577 17.3986839
H 10.9685220 9.3472363 18.0392211
C 4.9394421 14.6001097 26.3282481
H 5.9842874 14.8450209 26.5395271
H 4.6501624 13.7447869 26.9476648
H 4.3011665 15.4539271 26.5838961
H 3.2800462 7.3263652 19.9054515
C 5.4540877 9.1790110 23.6894278
H 5.9608480 10.3348948 23.1460718
N 6.8757005 11.0798577 22.7534065
C 8.0661600 10.4974733 23.2899031
H 8.8838722 10.6585045 22.5775479
H 8.3620615 10.9948374 24.2286572
C 7.9164596 8.9954477 23.5480085
H 8.8314227 8.6287595 24.0345486
C 6.6929782 8.7289685 24.4166171
H 6.8133700 9.2825111 25.3563557
H 6.6336536 7.6577961 24.6774244
H 7.8064750 8.4751795 22.5900511
H 5.2986848 8.6599765 22.7391095
C 4.2214162 9.4936577 24.3864787
C 1.7960979 10.3248896 25.6127341
C 4.1386968 9.7254388 25.7768882
C 3.0232777 9.6255946 23.6483921
C 1.8404115 10.0421863 24.2434102
C 2.9522593 10.1422401 26.3739282
H 5.0100046 9.5480045 26.4029601
H 3.0493671 9.3942541 22.5854519
H 0.9412830 10.1486909 23.6345788
H 2.9264288 10.3161763 27.4511018
H 0.8706120 10.6588039 26.0813724

TS2-proR(chair)quintet (E_{tot}=-4161.543647)

Fe 6.4576150 12.3081287 21.6474131
C 5.4022205 15.9834716 19.4942352
H 5.6454931 15.6644928 18.4861043
O 8.5655991 9.5504933 19.3804236
O 5.3175195 15.3410232 24.2390218
N 6.3015647 13.9022319 20.3583194
N 7.8075163 11.3458652 20.4938223
N 4.9935521 11.4378553 20.6404551
N 6.2754304 13.6569921 23.1125216
C 3.8768949 8.2256051 19.2661960
C 4.7017653 16.8190453 22.0430230
H 4.4334619 17.1268583 23.0508023
C 4.7595910 17.1857208 19.6826919
H 4.5188295 17.7953695 18.8107038
C 1.8003282 15.5049171 17.3960223
H 1.8166979 16.1129835 16.4908035
C 2.8577764 14.6702125 17.6782743
H 3.7097224 14.6135837 17.0054869
C 4.3934811 17.6233062 20.9677850
H 3.8735215 18.5689605 21.1112819
C 9.6781315 13.5020611 26.6186807
H 10.1345007 12.9304583 27.4273370
C 5.0764437 7.4893719 19.2384015
H 5.0811371 6.4432837 18.9361157
C 6.2548489 8.1317825 19.5594391
H 7.2036542 7.6059950 19.4766311
C 0.6956086 15.5857771 18.2731453
H -0.1352956 16.2522241 18.0401616
C 3.8603122 9.5384571 19.6796266
H 2.9239621 10.0854640 19.7356094
C 6.5399586 12.1362072 14.9926663
H 7.4651718 12.2594986 14.4275747
C 5.2689890 12.4845001 17.0658036
C 6.4811921 12.6383971 16.3165440
C 5.7236526 15.1080617 20.5757916
C 4.1689464 11.8316380 16.4548795
H 3.2532189 11.7164575 17.0309385
C 11.0063825 13.3065926 20.4217385
H 11.0914455 13.4290794 19.3417403
C 11.7996578 13.7796392 22.6517050
H 12.5032352 14.2745102 23.3211425
C 3.9416095 13.0024821 19.1751203
C 0.6734551 14.8253490 19.4211099
H -0.1717239 14.8846338 20.1085693
C 2.8618920 13.8679928 18.8467992
C 9.6925525 15.3904811 25.1193924
H 10.1649661 16.2993154 24.7471548
C 7.4968447 13.7383030 18.2233850
H 8.3151797 14.2859715 18.6885855
C 5.1975280 12.9794469 18.3954554
C 5.6670855 14.8218419 23.0467559
C 9.7503477 10.3728888 19.5732207
C 9.9843586 12.5083512 20.9445285
C 2.7945766 12.3462627 21.2277853

H 2.7897738 11.7263254 22.1187334

C 1.7444426 13.9546583 19.7411298

C 1.7434490 13.1795909 20.9268643

H 0.8887520 13.2463402 21.6010597

C 7.5712741 13.3122555 16.9159361

H 8.4713885 13.4925146 16.3263949

C 11.9127507 13.9395577 21.2700566

H 12.7047222 14.5618499 20.8520183

C 4.2600250 11.3497632 15.1684067

H 3.4068993 10.8425751 14.7168779

C 7.9033460 13.7657554 24.9856496

C 10.2673085 14.6877956 26.1789197

H 11.1840331 15.0494383 26.6447302

C 3.8924020 12.2531984 20.3444283

C 5.4408830 14.2596280 25.2071031

C 7.5389662 10.1607542 20.0053846

C 10.7643086 13.0079542 23.1758789

H 10.6447675 12.9100122 24.2506365

C 5.3663964 15.5813929 21.8809220

C 9.8562234 12.3851305 22.3265541

H 9.0305546 11.8093796 22.7329472

C 5.0390676 10.1965075 20.1323048

C 5.4545315 11.5035374 14.4279624

H 5.5137700 11.1189294 13.4094540

C 6.2746952 9.4823686 19.9724085

C 10.6083127 9.7170150 20.6373031

H 10.8523543 8.6943312 20.3278395

H 10.0829750 9.6906219 21.5957067

H 11.5316719 10.2885476 20.7747984

C 9.1264332 11.7283030 20.0047610

H 8.9731743 12.3192596 19.0944133

C 6.3375502 13.5133015 18.9992055

C 8.5232954 14.9280247 24.5217796

H 8.0809416 15.4748943 23.6921476

C 4.1137699 13.5248517 25.2468693

H 3.9117709 13.0423638 24.2866986

H 3.3045873 14.2270295 25.4768128

H 4.1487302 12.7569826 26.0259013

C 6.5207749 13.3823492 24.5289334

H 6.3426353 12.3227689 24.7180242

C 8.5025794 13.0465589 26.0222112

H 8.0293536 12.1310343 26.3795207

C 10.4699033 10.4746497 18.2455820

H 11.3227586 11.1542551 18.3534246

H 9.7928970 10.8638030 17.4775220

H 10.8424722 9.4918954 17.9334741

C 5.7965464 14.8679480 26.5428055

H 6.6960258 15.4838993 26.4631116

H 5.9862481 14.0696315 27.2684034

H 4.9618615 15.4817238 26.9010846

H 2.9405308 7.7484269 18.9727288

C 4.7802616 9.9436821 24.4268602

H 5.5139664 10.6941932 23.4968905

N 6.5057904 10.9774397 22.8225013

C 3.4367627 9.8341456 23.8778641

C 0.8251898 9.6900611 22.7874425

C 2.3700420 10.5884145 24.4145002

C 3.1444000 8.9927325 22.7856990

C 1.8633204 8.9250741 22.2528952

C 1.0888177 10.5199565 23.8790840

H 2.5551697 11.2276282 25.2748323

H 3.9390983 8.4141637 22.3252078

H 1.6815060 8.2830044 21.3921615

H 0.2888317 11.1183396 24.3172659

H -0.1751058 9.6422721 22.3576197

H 4.8181291 10.5716727 25.3213613

C 7.4016757 9.9140697 23.1467769

H 8.1248807 9.7989208 22.3309141

H 7.9889969 10.1404314 24.0619864

C 6.6520126 8.5986796 23.3419704

H 7.3683054 7.7845550 23.5273917

C 5.6698112 8.7150138 24.5084901

H 6.2519087 8.7609741 25.4405481

H 5.0537763 7.8039142 24.5583374

H 6.1241620 8.3760844 22.4096528

Int2-proS(boat)quintet (E_{tot}=-4161.55252)

Fe 6.3001941 12.4796195 21.5701702

C 5.7467652 15.8527883 18.8610402

H 5.9284131 15.3431448 17.9198344

O 8.8095379 10.3364496 19.0561009

O 5.2039422 15.9135383 23.6044157

N 6.1847386 13.7854612 20.0515369

N 7.8447575 11.5955445 20.6333085

N 4.9503121 11.3743565 20.6489431

N 5.8357864 13.9048285 22.8313776

C 4.2606679 8.5680227 18.3893834

C 5.2338782 17.1868922 21.2353687

H 5.0199263 17.6912120 22.1742479

C 5.4222985 17.1909156 18.8525604

H 5.3595336 17.7128021 17.8962001

C 1.4113035 15.5012554 17.8688689

H 1.3199757 16.1204377 16.9758793

C 2.4618479 14.6199476 17.9866083

H 3.2056912 14.5306352 17.1985111

C 5.1662170 17.8838622 20.0484786

H 4.9064048 18.9411064 20.0398495

C 8.6450946 13.5313015 26.8014361

H 8.8219032 13.0550502 27.7663228

C 5.5479107 8.1221649 18.0513424

H 5.6880157 7.2676930 17.3913665

C 6.6382584 8.8057303 18.5527104

H 7.6432758 8.4962555 18.2760753

C 0.4509971 15.6164139 18.8999727

H -0.3768996 16.3177649 18.7937220

C 4.0890138 9.6438888 19.2330596

H 3.0898723 9.9901349 19.4882579

C 5.6333558 11.3224922 14.9806255

H 6.5135329 11.1825707 14.3514408

C 4.6439780 12.1503584 17.0706186

C 5.7912654 11.9629300 16.2346225

C 5.8502459 15.0991298 20.0698816

C 3.3873887 11.6773323 16.6164523

H 2.5213932 11.8100485 17.2607999

C 11.0586255 13.2347915 21.7420864

H 11.3079185 13.7518470 20.8150074

C 11.5692731 12.7023074 24.0395730

H 12.2076542 12.8112127 24.9159320

C 3.6796695 12.9018048 19.2974858

C 0.5669713 14.8496795 20.0388581

H -0.1640235 14.9400178 20.8438375

C 2.6018125 13.8070484 19.1401510

C 9.3699866 14.9653918 25.0040403

H 10.1299677 15.5980664 24.5479787

C 7.1706645 13.0262772 17.9193053

H 8.1283453 13.4138434 18.2501908

C 4.7966707 12.7732822 18.3373292

C 5.5500890 15.1598464 22.5469602

C 9.9191855 11.0344406 19.6799315

C 9.9187202 12.4266371 21.7973675

C 2.8634753 12.3072225 21.5163832

H 3.0115773 11.7274015 22.4253720

C 1.6380399 13.9364492 20.1954251

C	1.8008669	13.1697207	21.3755082
H	1.0709705	13.2814542	22.1783614
C	7.0479010	12.4223880	16.6894963
H	7.9214868	12.2932760	16.0496812
C	11.8860595	13.3670373	22.8553182
H	12.7765743	13.9940374	22.7987859
C	3.2660542	11.0604046	15.3925131
H	2.2917898	10.7006489	15.0600009
C	7.2060840	13.8946680	24.8916012
C	9.6000131	14.3851905	26.2511523
H	10.5346151	14.5769958	26.7784040
C	3.8079225	12.1591925	20.4739986
C	4.9339064	14.9934656	24.6960263
C	7.6973204	10.6467577	19.7495309
C	10.4013635	11.9460800	24.1140607
H	10.1103371	11.4851261	25.0560811
C	5.5660830	15.8123722	21.2769199
C	9.5779383	11.8108451	23.0009073
H	8.6401058	11.2692187	23.0661881
C	5.1871197	10.3481957	19.7959542
C	4.3977759	10.8800143	14.5647175
H	4.2890207	10.3877150	13.5979450
C	6.4986301	9.9216871	19.4057578
C	10.6489551	10.0160727	20.5375400
H	10.9804307	9.1858753	19.9035995
H	9.9782228	9.6356344	21.3147485
H	11.5133807	10.4755763	21.0260095
C	9.1928118	12.1324540	20.5160408
H	9.1389831	13.0646234	19.9349470
C	6.0576469	13.1850994	18.7706428
C	8.1822178	14.7153591	24.3234229
H	8.0076501	15.1650679	23.3485232
C	3.4593334	14.6316554	24.6135253
H	3.2516642	14.0878241	23.6867324
H	2.8593027	15.5486579	24.6300000
H	3.1837034	14.0138581	25.4716163
C	5.8281930	13.7964966	24.2963650
H	5.3450455	12.8746501	24.6277485
C	7.4529028	13.2885992	26.1234985
H	6.6964856	12.6474593	26.5650792
C	10.7987433	11.6010744	18.5884775
H	11.6249989	12.1565239	19.0450736
H	10.2245960	12.2732887	17.9430116
H	11.2179412	10.7920757	17.9786105

C	5.2829646	15.6763990	25.9958656
H	6.3139453	16.0391479	25.9760173
H	5.1851792	14.9563775	26.8142581
H	4.5980330	16.5148765	26.1687917
H	3.3827681	8.0631942	17.9837647
H	3.7973284	10.3861064	24.5898174
C	4.6299207	9.9906624	25.1729144
H	5.7036983	11.3545883	23.6655012
C	5.3327494	8.7809308	24.6667693
H	4.6955335	8.2705328	23.9309542
H	5.4882779	8.0745111	25.4983781
C	6.6969167	9.0456532	24.0020924
H	7.1593914	8.0723895	23.7798621
H	7.3566723	9.5780181	24.6994938
C	6.5914755	9.8409637	22.7048742
H	5.7760721	9.4192639	22.0916866
H	7.5072416	9.6990542	22.1198600
N	6.4069184	11.2497407	22.9313738
C	4.9068598	10.6138898	26.4088118
C	5.4170642	11.9292692	28.8737888
C	5.9719406	10.2062531	27.2614675
C	4.1006427	11.6958442	26.8610021
C	4.3534649	12.3401148	28.0592272
C	6.2127681	10.8525754	28.4640935
H	6.6154554	9.3843407	26.9565386
H	3.2579122	11.9994582	26.2424225
H	3.7181545	13.1694025	28.3719781
H	7.0426999	10.5251947	29.0915249
H	5.6223872	12.4411825	29.8131419

Int2-proR(boat)quintet (E_{tot}=-4161.55388)

Fe	6.4913550	12.3944291	21.6008992
C	5.3301533	15.8816481	19.2996858
H	5.6708405	15.5723356	18.3161988
O	8.8283161	10.1575871	18.9876504
O	5.1394696	15.3729206	24.0532965
N	6.2926615	13.8627392	20.2334414
N	7.9644475	11.5165080	20.5449586
N	5.0784271	11.4227633	20.6544487
N	6.2112743	13.7096517	23.0080584
C	4.1870541	8.5752719	18.5234897
C	4.4014879	16.6822402	21.7842435
H	4.0517810	16.9826039	22.7690548
C	4.5848364	17.0321349	19.4271637
H	4.3597543	17.6165334	18.5343840

C	1.5790506	15.4135791	17.6365470
H	1.4876420	15.9724983	16.7044859
C	2.6436502	14.5615039	17.8216373
H	3.3986752	14.4421992	17.0484808
C	4.0939087	17.4434394	20.6777595
H	3.4858549	18.3414292	20.7728603
C	9.7733063	14.3423359	26.2995722
H	10.2307879	14.0756094	27.2529130
C	5.4415571	8.0503864	18.1733709
H	5.5210056	7.1596819	17.5522495
C	6.5783404	8.7028081	18.6102131
H	7.5589637	8.3345584	18.3183716
C	0.6058493	15.5774545	18.6484207
H	-0.2324481	16.2565104	18.4903711
C	4.0918878	9.6963772	19.3190988
H	3.1217915	10.1002527	19.5943028
C	6.1996546	11.7084941	14.9977201
H	7.1111538	11.7101153	14.3981149
C	5.0415836	12.3056450	17.0799042
C	6.2322810	12.3027078	16.2835794
C	5.6277834	15.0364230	20.4084933
C	3.8701000	11.7054952	16.5539689
H	2.9739961	11.7016756	17.1702360
C	11.2749267	13.0825132	21.4591500
H	11.5246680	13.5122503	20.4887975
C	11.8025041	12.7335029	23.7864382
H	12.4562435	12.8961520	24.6423041
C	3.8775182	12.9493575	19.2563406
C	0.7227561	14.8847281	19.8330788
H	-0.0166935	15.0137441	20.6250427
C	2.7844444	13.8223472	19.0233566
C	9.8325294	15.5460418	24.2127372
H	10.3420795	16.2189739	23.5235776
C	7.4050695	13.4434428	18.0721596
H	8.2833688	13.9597092	18.4511434
C	5.0653959	12.8800176	18.3766160
C	5.5110777	14.8173454	22.8872430
C	9.9859133	10.8231879	19.5561887
C	10.1052299	12.3276920	21.5986295
C	3.0173066	12.4513624	21.4839354
H	3.1242135	11.8908717	22.4063976
C	1.8057130	13.9998081	20.0576199
C	1.9612353	13.3082068	21.2833154
H	1.2199853	13.4534096	22.0693630

C 7.3984326 12.9091036 16.8035759

H 8.2914751 12.9575967 16.1792577

C 12.1246795 13.2791730 22.5439457

H 13.0341208 13.8685178 22.4225239

C 3.8706531 11.1346051 15.3021836

H 2.9625706 10.6702961 14.9163731

C 7.9548222 14.1082177 24.7135948

C 10.4101410 15.2399654 25.4432416

H 11.3684659 15.6775494 25.7226840

C 3.9662354 12.2489355 20.4601043

C 5.4463413 14.3992777 25.0935384

C 7.7520801 10.5442184 19.7006340

C 10.6135893 12.0252551 23.9390503

H 10.3288903 11.6467517 24.9187195

C 5.1704862 15.5004225 21.6844176

C 9.7655336 11.8255856 22.8530474

H 8.8144647 11.3173485 22.9810299

C 5.2393749 10.3613482 19.8289581

C 5.0447019 11.1352620 14.5142685

H 5.0329550 10.6783333 13.5243340

C 6.5156421 9.8599743 19.4161594

C 10.6946002 9.8106519 20.4372639

H 10.9687846 8.9371253 19.8349411

H 10.0340038 9.4978842 21.2521710

H 11.5941012 10.2524067 20.8767003

C 9.3317214 11.9850900 20.3606230

H 9.2992976 12.8777986 19.7240479

C 6.2628309 13.3864754 18.8973166

C 8.6084666 14.9873841 23.8512769

H 8.1581664 15.2232044 22.8894896

C 4.2192903 13.5246332 25.2850961

H 3.9929305 12.9813277 24.3640208

H 3.3541761 14.1387398 25.5589758

H 4.4084045 12.7982927 26.0829911

C 6.5737371 13.5815351 24.4192938

H 6.5237270 12.5298430 24.7130782

C 8.5590448 13.7730880 25.9284443

H 8.0633642 13.0655867 26.5940963

C 10.8548461 11.3066426 18.4169915

H 11.7232751 11.8357905 18.8234255

H 10.2863569 11.9818181 17.7694215

H 11.2114443 10.4562630 17.8237693

C 5.8011836 15.1760310 26.3384286

H 6.6511898 15.8379943 26.1526335

H 6.0659304 14.4866149 27.1463647

H 4.9349414 15.7701685 26.6527753

H 3.2747830 8.0934353 18.1677423

C 4.5948323 9.5275005 24.7780804

H 5.6608720 11.0891092 23.4234515

C 5.5729973 8.4461446 24.4906410

H 5.1523476 7.7348353 23.7687917

H 5.7402142 7.8782669 25.4231158

C 6.9442857 8.9047045 23.9713331

H 7.5660383 8.0092542 23.8208187

H 7.4471360 9.5311229 24.7219814

C 6.8546139 9.6864749 22.6602235

H 6.1189652 9.1989932 21.9988286

H 7.8150576 9.6318852 22.1385397

N 6.5394335 11.0708908 22.8955068

C 3.3847338 9.7532405 24.0813961

C 0.8927058 10.1840992 22.7916991

C 2.3914161 10.6094436 24.6387359

C 3.0820166 9.1489224 22.8291072

C 1.8612408 9.3595557 22.2101570

C 1.1751106 10.8135675 24.0095983

H 2.5966588 11.0953107 25.5913876

H 3.8290131 8.5397197 22.3279232

H 1.6674247 8.8925009 21.2453972

H 0.4354970 11.4719185 24.4668136

H -0.0576786 10.3570754 22.2887247

H 4.7720946 10.1276896 25.6726475

Int2-proS(chair)quintet (E_{tot}=-4161.5589)

Fe 6.6922370 12.4642610 21.5312868

C 6.0967100 16.2025780 19.3794744

H 6.2167832 15.8398648 18.3635055

O 8.8328673 9.5547601 19.6383433

O 5.2084986 15.4304540 23.9968753

N 6.4800306 13.9641033 20.2413635

N 7.9739354 11.4665696 20.4348503

N 5.2015089 11.4273912 20.6756358

N 5.8998316 13.6055281 22.8901465

C 4.2022154 7.9500460 20.0492496

C 5.6761857 17.1704730 21.9492492

H 5.4750910 17.5225150 22.9585373

C 5.8726623 17.5430115 19.5952493

H 5.8309275 18.2148054 18.7361727

C 1.8531521 15.5241426 17.6315056

H 1.8524171 16.1825606 16.7622071

C 2.9312575 14.6988100 17.8625302

H 3.7798470 14.6987649 17.1830105

C 5.6851507 18.0551353 20.8941517

H 5.5154813 19.1181319 21.0573461

C 7.8180020 11.6422125 26.8988180

H 7.7386874 10.9052490 27.6988163

C 5.4472476 7.2913363 20.0819232

H 5.5040959 6.2075259 19.9948985

C 6.5973313 8.0478696 20.1802256

H 7.5719601 7.5657195 20.1387841

C 0.7504207 15.5308158 18.5141282

H -0.0965106 16.1895681 18.3207912

C 4.1225384 9.3192440 20.1782499

H 3.1570374 9.8197630 20.1541825

C 6.3430531 12.1325792 14.8943152

H 7.2207302 12.2620863 14.2591059

C 5.2360624 12.4739170 17.0603379

C 6.3833173 12.6363113 16.2178912

C 6.1859471 15.2637198 20.4541176

C 4.0972072 11.8123310 16.5373553

H 3.2284865 11.6920179 17.1814024

C 10.8461338 13.7149456 19.6101908

H 10.9248573 13.4858945 18.5474809

C 11.4386574 15.0962970 21.4974467

H 11.9939447 15.9335190 21.9204270

C 4.0597173 12.9699690 19.2517623

C 0.7530740 14.7106644 19.6210254

H -0.0884293 14.7142959 20.3155767

C 2.9594321 13.8327863 18.9848140

C 9.1145719 13.2328005 25.6355567

H 10.0591718 13.7433876 25.4519951

C 7.5361390 13.7536125 18.0335328

H 8.3882206 14.2963664 18.4369249

C 5.2624802 12.9781433 18.3913695

C 5.6990834 14.9076870 22.8587520

C 9.9418494 10.5005264 19.5729340

C 9.9827135 12.9581218 20.4095597

C 2.9648495 12.2180022 21.2914126

H 3.0142662 11.6120032 22.1902226

C 1.8460561 13.8505194 19.8874459

C 1.8832562 13.0265286 21.0388036

H 1.0456950 13.0585355 21.7372353

C 7.5140495 13.3173711 16.7292050

H 8.3692439 13.4903816 16.0740803

C 11.5784721 14.7703139 20.1481301

H 12.2429350 15.3524208 19.5090850

C 4.0902274 11.3305045 15.2470528

H 3.2069693 10.8191018 14.8634614

C 6.8010178 12.8397119 25.0544642

C 9.0249562 12.3044306 26.6741750

H 9.8946107 12.0907021 27.2954613

C 4.0575441 12.1742103 20.3929078

C 4.7289547 14.3152037 24.7974397

C 7.7763894 10.1925537 20.1872426

C 10.5708142 14.3536493 22.2966079

H 10.4440450 14.6133103 23.3453086

C 5.9014642 15.7868213 21.7594254

C 9.8539542 13.2859134 21.7595950

H 9.1772509 12.7191496 22.3946384

C 5.2791703 10.1195091 20.4126083

C 5.2213834 11.4917415 14.4157270

H 5.2038139 11.1069574 13.3957132

C 6.5561103 9.4515096 20.3280860

C 10.9419689 10.1664282 20.6616442

H 11.2800716 9.1305436 20.5443727

H 10.5021029 10.2888106 21.6532621

H 11.8027211 10.8393063 20.5847338

C 9.2075405 11.8518781 19.7666545

H 8.9255618 12.1983626 18.7636329

C 6.4320693 13.5407303 18.8927210

C 8.0086890 13.5016854 24.8317096

H 8.0806107 14.2046097 24.0053798

C 3.2607039 14.1063979 24.4662646

H 3.1443691 13.9015938 23.3982101

H 2.6869696 15.0036949 24.7252656

H 2.8733312 13.2517960 25.0323446

C 5.5759125 13.1437447 24.2393465

H 4.9467783 12.2498070 24.1835377

C 6.7141205 11.9141920 26.0946276

H 5.7733845 11.3939580 26.2565018

C 10.5599233 10.3798157 18.1948483

H 11.3540406 11.1273682 18.0880017

H 9.8014049 10.5466092 17.4222924

H 10.9978320 9.3840722 18.0585048

C 4.9473740 14.6568069 26.2523145

H 6.0024902 14.8682065 26.4473550

H 4.6426418 13.8094341 26.8753244

H 4.3405895 15.5300344 26.5188113

H 3.2854258 7.3712531 19.9257147

C 5.4380063 8.8481055 23.8111208

H 6.0722944 10.8243898 23.2559930

N 6.9120342 11.1378955 22.7702367

C 8.0755477 10.4838261 23.2799969

H 8.8921231 10.6557725 22.5747383

H 8.3884385 10.9379845 24.2371112

C 7.9207598 8.9770792 23.4895403

H 8.8639136 8.5955671 23.9082513

C 6.7688355 8.6164127 24.4307825

H 6.8756265 9.1830747 25.3649709

H 6.8639252 7.5475950 24.7035808

H 7.7617174 8.4886709 22.5228919

H 5.3589299 8.6546595 22.7419510

C 4.2725017 9.2804846 24.4782168

C 1.9230426 10.3111170 25.6961930

C 4.1935383 9.4846914 25.8852286

C 3.0979543 9.5566276 23.7207734

C 1.9588714 10.0700931 24.3154226

C 3.0458523 9.9964739 26.4716039

H 5.0444426 9.2241664 26.5113450

H 3.1266607 9.3683916 22.6492330

H 1.0856467 10.2929160 23.7009850

H 3.0172207 10.1491951 27.5516295

H 1.0279681 10.7214251 26.1629102

Int2-proR(chair)quintet (E_{tot}=-4161.55176)

Fe 6.5432001 12.3367490 21.6875002

C 5.2582255 15.8306110 19.4577187

H 5.5930360 15.5443414 18.4654942

O 8.7799339 10.0096469 19.0710432

O 5.1703062 15.2587003 24.2068568

N 6.3087414 13.8398565 20.3603584

N 7.9620726 11.4576675 20.5751022

N 5.0955711 11.3932061 20.7578803

N 6.2661629 13.6443247 23.1101265

C 4.1286870 8.4066637 18.8629438

C 4.3319506 16.5690800 21.9638643

H 3.9853806 16.8443774 22.9571948

C 4.4730367 16.9511261 19.6087902

H 4.2127338 17.5345789 18.7251040

C 1.6709671 15.2723234 17.5248539

H 1.6090269 15.8078537 16.5767286

C 2.7382191 14.4389640 17.7712260

H 3.5232110 14.3125545 17.0297835

C 3.9843879 17.3317170 20.8705087

H 3.3459374 18.2062983 20.9829687

C 9.8953920 14.4190682 26.2945016

H 10.4014215 14.1573325 27.2244289

C 5.3718373 7.8384232 18.5376977

H 5.4298358 6.8940703 17.9987241

C 6.5231431 8.5119231 18.8959100

H 7.4952123 8.1105109 18.6188423

C 0.6564551 15.4460001 18.4934282

H -0.1834377 16.1099703 18.2871046

C 4.0584630 9.5944902 19.5583452

H 3.0969609 10.0222132 19.8279359

C 6.4307305 11.7539433 15.1026323

H 7.3467785 11.8272472 14.5144897

C 5.2024225 12.2554725 17.1691892

C 6.4011102 12.3461151 16.3895275

C 5.6011451 14.9843822 20.5530546

C 4.0860397 11.5714698 16.6265753

H 3.1849798 11.4991583 17.2314364

C 11.2361578 13.2078061 21.2179066

H 11.4106119 13.5881150 20.2110066

C 11.8947361 13.0420602 23.5326186

H 12.5751281 13.3000158 24.3429289

C 3.9380978 12.8807902 19.2959432

C 0.7349072 14.7811085 19.6968921

H -0.0371288 14.9173066 20.4558779

C 2.8417156 13.7305321 18.9949112

C 9.8054955 15.6787410 24.2419206

H 10.2466609 16.4012904 23.5556909

C 7.4577902 13.5650774 18.1982769

H 8.2801228 14.1588937 18.5926346

C 5.1623893 12.8281330 18.4664693

C 5.5288123 14.7311112 23.0231486

C 9.9530589 10.7066295 19.5696479

C 10.1270384 12.3917033 21.4633714

C 2.9912615 12.4185398 21.4954343

H 3.0613521 11.8782915 22.4335129

C 1.8195551 13.9166216 19.9841660

C 1.9318419 13.2533040 21.2298202

H 1.1540264 13.4015049 21.9787906

C 7.5060569 13.0492018 16.9225573

H 8.3925179 13.1897446 16.3027374

C 12.1200495 13.5285262 22.2450263

H 12.9809361 14.1664170 22.0422290

C	4.1480698	11.0024066	15.3752685
H	3.2837758	10.4700426	14.9771008
C	8.0379450	14.1146271	24.7662248
C	10.4454089	15.3762334	25.4423774
H	11.3837583	15.8662853	25.7020845
C	3.9858642	12.2122604	20.5176286
C	5.5350968	14.2774076	25.2218644
C	7.7261851	10.4357853	19.7966986
C	10.7674459	12.2656501	23.7882502
H	10.5585023	11.9297667	24.8013466
C	5.1407648	15.4167820	21.8386520
C	9.8835391	11.9472212	22.7615312
H	8.9818589	11.3782983	22.9623616
C	5.2231364	10.2806199	19.9987578
C	5.3293908	11.0945731	14.6035703
H	5.3659762	10.6399580	13.6131929
C	6.4851095	9.7329289	19.6031922
C	10.6772794	9.7591908	20.5077373
H	10.9397773	8.8432997	19.9661268
H	10.0349774	9.5081870	21.3573355
H	11.5857772	10.2311735	20.8943552
C	9.3151449	11.9239566	20.2960989
H	9.2387879	12.7457724	19.5754893
C	6.3196397	13.3941606	19.0145262
C	8.6060168	15.0544693	23.9068839
H	8.1088805	15.2866963	22.9676639
C	4.3460544	13.3522877	25.4157159
H	4.1225343	12.8251622	24.4843040
H	3.4635846	13.9259741	25.7200166
H	4.5769038	12.6138006	26.1917202
C	6.6777982	13.5191351	24.5072305
H	6.6868772	12.4613807	24.7843235
C	8.7040388	13.7869314	25.9504314
H	8.2748214	13.0355049	26.6143418
C	10.8027262	11.1081262	18.3853283
H	11.6703172	11.6742511	18.7414343
H	10.2199641	11.7280931	17.6964461
H	11.1609321	10.2186537	17.8537306
C	5.8857169	15.0411150	26.4756900
H	6.7013446	15.7438592	26.2851736
H	6.2002855	14.3457572	27.2604504
H	5.0027776	15.5896291	26.8245770
H	3.2055566	7.9027060	18.5712756
C	4.4156670	9.3846960	24.5308196
H	5.7484460	10.9897798	23.4969509
N	6.6346340	11.0167319	22.9848806
C	3.1565063	9.6943260	23.9673118
C	0.6174115	10.3500390	22.8865675
C	2.2915262	10.6273169	24.6064209
C	2.6855641	9.0959231	22.7659814
C	1.4458373	9.4249525	22.2433043
C	1.0521290	10.9447700	24.0769380
H	2.6193529	11.0921637	25.5345896
H	3.3165148	8.3914289	22.2322077
H	1.1218289	8.9671234	21.3084990
H	0.4156260	11.6677235	24.5885402
H	-0.3480292	10.6157749	22.4579259
H	4.7044104	9.9305189	25.4298898
C	7.1495038	9.6736974	22.9190588
H	7.8530325	9.6098186	22.0856595
H	7.7438472	9.4619177	23.8335865
C	6.1060906	8.5719037	22.7691084
H	6.6154410	7.6412749	22.4775605
C	5.3051747	8.2892988	24.0533789
H	6.0166139	8.0279272	24.8502048
H	4.6919886	7.3916043	23.8675444
H	5.4310709	8.8392727	21.9541719

product-quintet (E_{tot}=-4161.61739)

Fe	6.3808150	12.5840798	21.6848115
C	5.6258670	16.2060768	19.2219285
H	5.7103405	15.7498799	18.2397365
O	8.6227187	10.4438213	18.9513241
O	5.5724892	15.9940792	24.0074616
N	6.2529364	14.0958217	20.2282471
N	7.8247060	11.5748039	20.7260472
N	4.9314528	11.5424635	20.8285154
N	6.1036277	14.0145276	23.0819752
C	4.0025739	8.7414918	18.6349158
C	5.3519016	17.3796029	21.7190605
H	5.2293885	17.8267623	22.7022523
C	5.2775426	17.5308907	19.3304368
H	5.0937038	18.1085602	18.4227893
C	1.5424980	15.7504518	17.9649481
H	1.4929453	16.3744736	17.0719155
C	2.5854290	14.8669092	18.1251916
H	3.3624500	14.7811636	17.3697208
C	5.1460418	18.1433673	20.5928042
H	4.8702082	19.1933804	20.6791410
C	9.1145487	13.1961459	26.8394251
H	9.3161713	12.6398020	27.7558205
C	5.2509118	8.2598376	18.2074037
H	5.3187790	7.4014432	17.5407562
C	6.3938302	8.9077666	18.6294020
H	7.3685780	8.5698593	18.2848377
C	0.5372639	15.8626563	18.9523781
H	-0.2825708	16.5680804	18.8143090
C	3.9248750	9.8293568	19.4785711
H	2.9561881	10.2078095	19.7983916
C	5.7472486	11.6963371	15.1340376
H	6.6212036	11.6310264	14.4841016
C	4.7480481	12.4200388	17.2582982
C	5.8851295	12.3310113	16.3928193
C	5.8834288	15.3818457	20.3628282
C	3.5193789	11.8658372	16.8210148
H	2.6609539	11.9267663	17.4858048
C	11.1028399	13.1462982	21.7000058
H	11.2736816	13.7634197	20.8176212
C	11.7758162	12.4039125	23.8975926
H	12.4644618	12.4509160	24.7401947
C	3.7451862	13.1365771	19.4748544
C	0.5982023	15.0847983	20.0875274
H	-0.1699421	15.1675610	20.8580671
C	2.6734621	14.0461448	19.2782345
C	9.8349634	14.7082745	25.1032168
H	10.6111024	15.3227669	24.6500339
C	7.2152124	13.4908387	18.0532327
H	8.1420962	13.9665177	18.3639075
C	4.8795931	13.0412499	18.5295643
C	5.8147416	15.2897810	22.8755635
C	9.7958089	11.0649051	19.5296400
C	9.9961741	12.2947311	21.7411287
C	2.7949831	12.4861510	21.6179448
H	2.8433400	11.8511963	22.4977541
C	1.6576103	14.1649431	20.2826942
C	1.7489978	13.3635767	21.4457954
H	0.9683846	13.4457961	22.2037862
C	7.1107749	12.8931488	16.8200177
H	7.9710285	12.8517043	16.1507321
C	11.9952591	13.1931540	22.7692147
H	12.8611418	13.8545092	22.7249504
C	3.4151983	11.2596317	15.5898210
H	2.4617348	10.8355696	15.2728118

C 7.5954453 13.8045985 25.0533339

C 10.0939784 14.0212945 26.2898254

H 11.0686235 14.1096277 26.7697015

C 3.8081755 12.3648640 20.6379277

C 5.3641269 15.0274051 25.0595382

C 7.5820935 10.7037281 19.7736714

C 10.6460701 11.5903519 23.9630271

H 10.4459822 11.0084366 24.8614493

C 5.7093243 16.0077344 21.6481947

C 9.7652779 11.5383358 22.8883498

H 8.8930470 10.8965525 22.8999961

C 5.0779866 10.4999931 19.9640282

C 4.5379268 11.1706469 14.7363580

H 4.4440264 10.6846178 13.7647732

C 6.3480763 10.0237150 19.4968774

C 10.5785688 9.9687090 20.2318459

H 10.8517851 9.1997995 19.5003590

H 9.9651158 9.5164374 21.0176432

H 11.4843358 10.3761150 20.6916032

C 9.1661926 12.1013411 20.5085271

H 9.0647111 13.0695572 20.0003034

C 6.1151702 13.5451642 18.9408468

C 8.5958958 14.5931724 24.4813268

H 8.3903519 15.1251962 23.5548238

C 3.8840170 14.6721393 25.0815774

H 3.5930723 14.2178605 24.1278922

H 3.2884469 15.5780415 25.2407890

H 3.6888595 13.9646111 25.8936226

C 6.1867625 13.8279913 24.5301594

H 5.6790072 12.9140201 24.8363634

C 7.8686117 13.0907107 26.2230121

H 7.0893839 12.4705389 26.6625917

C 10.5933531 11.7147720 18.4210849

H 11.4544644 12.2350699 18.8552352

H 9.9705759 12.4314706 17.8784456

H 10.9613980 10.9567716 17.7195598

C 5.8267311 15.6328027 26.3631964

H 6.8692482 15.9546257 26.2897957

H 5.7487487 14.8864583 27.1608712

H 5.1939192 16.4916482 26.6159320

H 3.0844526 8.2590821 18.2968590

H 4.4611838 11.6865749 23.7040758

C 5.0095497 10.7888672 24.0112329

C 4.4160237 9.5526035 23.3323780

H 4.1222541 9.8255917 22.3160332

H 3.5499666 9.1646357 23.8779901

C 5.5994192 8.5969370 23.3009615

H 5.4924708 7.8193522 22.5367683

H 5.7184488 8.1126180 24.2780813

C 6.7767619 9.5247880 23.0104800

H 6.9906245 9.5616018 21.9403728

H 7.6872261 9.1971608 23.5302627

N 6.3641877 10.8790365 23.4488934

C 4.9634187 10.6804130 25.5171653

C 4.8114539 10.5219312 28.3209853

C 3.8454105 11.1670941 26.2031860

C 5.9976561 10.1008741 26.2565062

C 5.9239886 10.0203815 27.6471217

C 3.7673507 11.0938186 27.5914393

H 3.0330268 11.6195707 25.6348987

H 6.8799985 9.7221615 25.7445573

H 6.7447534 9.5707860 28.2068098

H 2.8928961 11.4907811 28.1080176

H 4.7578260 10.4697405 29.4083385

H 7.0063077 11.2583707 24.1357958

Triplet:

nitrene(triplet) (E_{tot}=-4161.55643)

Fe 6.9387458 13.0700063 21.9124149

C 6.2052851 16.1006591 18.7082615

H 6.2875526 15.4433347 17.8473303

O 8.1798998 9.9511679 19.5999353

O 6.3537285 16.9538293 23.4178390

N 6.7643045 14.2283072 20.1419578

N 7.8609169 11.7319028 20.9122115

N 5.2416811 12.3416021 21.4924043

N 6.6053301 14.7932884 22.8755477

C 3.3255844 9.3894406 20.4593951

C 5.9603074 17.8079608 20.8707637

H 5.8490027 18.4579993 21.7352135

C 5.8744974 17.4243080 18.5149765

H 5.7044273 17.7841060 17.4987770

C 1.9148776 16.2775302 18.2386500

H 1.7453724 16.6574053 17.2306702

C 2.8456965 15.2856860 18.4493607

H 3.4179423 14.8737393 17.6219024

C 5.7474405 18.3044255 19.6013324

H 5.4756279 19.3479832 19.4508301

C 9.9618434 14.2090647 26.3520872

H 10.2257767 13.8151690 27.3345250

C 4.3247138 8.5186871 19.9929873

H 4.0720584 7.5258651 19.6250801

C 5.6348018 8.9552702 19.9937416

H 6.4279609 8.3157909 19.6123646

C 1.1826801 16.8156570 19.3206294

H 0.4484007 17.6013485 19.1409777

C 3.6406273 10.6439416 20.9346261

H 2.8597086 11.3099639 21.2926667

C 4.8375425 10.9432223 15.9524553

H 5.5394288 10.5324797 15.2250142

C 4.4268376 12.3450674 17.9293702

C 5.3362356 11.7949052 16.9687184

C 6.4387136 15.5443779 20.0024617

C 3.0547941 12.0002953 17.8357224

H 2.3683817 12.4060699 18.5749734

C 11.4107001 12.9240892 21.0815472

H 11.4531491 13.3281595 20.0691380

C 12.4307299 12.5875310 23.2420691

H 13.2638097 12.7378623 23.9283437

C 4.0424685 13.7627324 20.0027173

C 1.4027775 16.3539126 20.6000160

H 0.8493173 16.7716797 21.4423133

C 3.0844894 14.7791238 19.7508013

C 10.6081943 15.1960682 24.2495704

H 11.3817428 15.5573631 23.5739858

C 7.1654145 12.9354988 18.0763752

H 8.2076867 13.2397477 18.1117106

C 4.9221816 13.1864026 18.9610075

C 6.4207981 16.0152732 22.4529610

C 9.5187718 10.5063501 19.7642292

C 10.2794374 12.2140897 21.4917705

C 3.5603243 13.8951601 22.3917598

H 3.7833050 13.5343401 23.3927103

C 2.3544920 15.3361876 20.8520675

C 2.6180460 14.8705826 22.1647516

H 2.0595259 15.3028473 22.9958341

C 6.7066058 12.1294687 17.0605104

H 7.3939703 11.7471518 16.3047651

C 12.4873071 13.1039109 21.9488143

H 13.3673997 13.6549192 21.6157874

C 2.6009712 11.1678503 16.8380954

H 1.5423217 10.9114994 16.7855053

C 8.2795309 14.7038538 24.6806803

C 10.9520025 14.7227232 25.5151328
H 11.9935936 14.7268080 25.8360646
C 4.2658142 13.3312686 21.3094441
C 6.2565722 16.2496970 24.6872329
C 7.3436901 10.6730841 20.3538569
C 11.2874811 11.9121503 23.6675645
H 11.2200021 11.5449769 24.6905729
C 6.2953433 16.4548353 21.0971527
C 10.2209048 11.7250261 22.7959947
H 9.3291206 11.2008547 23.1144631
C 4.9813050 11.1144391 20.9753348
C 3.4974263 10.6339518 15.8845690
H 3.1255276 9.9760032 15.0986928
C 5.9837797 10.2347835 20.4697841
C 10.2925725 9.5144311 20.6108087
H 10.3273208 8.5522148 20.0872002
H 9.8045782 9.3774522 21.5800636
H 11.3111274 9.8742297 20.7845364
C 9.2411377 11.8761118 20.4630728
H 9.2487545 12.6756821 19.7133159
C 6.2950319 13.4436519 19.0654962
C 9.2804826 15.1819578 23.8337358
H 9.0102714 15.5416727 22.8427815
C 4.7835521 16.1542277 25.0487129
H 4.2433557 15.6325245 24.2558856
H 4.3590481 17.1578216 25.1689101
H 4.6684926 15.6033177 25.9903307
C 6.8249933 14.8536622 24.3182886
H 6.2496896 14.0572353 24.8000197
C 8.6327134 14.1992082 25.9329409
H 7.8547409 13.8065390 26.5892582
C 10.1389854 10.6858177 18.3962059
H 11.1132882 11.1745774 18.5127948
H 9.4955987 11.3062440 17.7672361
H 10.2890486 9.7147438 17.9101714
C 7.0398411 17.0393404 25.7114171
H 8.0779619 17.1644970 25.3923138
H 7.0358325 16.5064457 26.6681722
H 6.5751457 18.0227786 25.8494964
H 2.2822587 9.0712566 20.4557755
H 2.9442995 9.8823571 24.0352075
C 3.5386708 9.3107472 24.7626492
H 3.4022103 9.7917271 25.7417012
C 5.0096713 9.4016949 24.3498699
H 5.1236497 8.9489354 23.3560466
H 5.6235987 8.8096597 25.0446749
C 5.4889401 10.8426272 24.3003605
H 5.4264641 11.3109850 25.2925854
H 4.8493540 11.4158366 23.6236110
C 6.9259776 10.9681621 23.7672051
H 7.0511935 10.2578526 22.9302485
H 7.6355129 10.6723834 24.5634276
N 7.1943468 12.2964559 23.3569416
C 3.0470669 7.8919064 24.8154390
C 2.1879574 5.2137138 24.8882965
C 2.8216240 7.1800031 23.6304061
C 2.8402044 7.2390000 26.0343981
C 2.4129682 5.9112871 26.0745428
C 2.3948933 5.8540563 23.6648576
H 2.9882577 7.6761646 22.6748125
H 3.0128572 7.7817608 26.9655822
H 2.2528626 5.4215509 27.0360633
H 2.2199562 5.3162955 22.7318478
H 1.8508819 4.1770805 24.9164114

TS2-proS(boat)triplet (E_{tot}=-4161.53064)

Fe 6.6416364 12.4020745 21.7546297
C 5.6225968 15.8317373 19.0873156
H 5.8937542 15.3913399 18.1322731
O 8.7821060 10.2838528 19.0204451
O 5.3897961 15.7821189 23.8564527
N 6.4347774 13.8364621 20.1970105
N 7.9527363 11.5630092 20.6569881
N 5.1956427 11.5037732 20.9580068
N 5.9817244 13.8003601 22.9904621
C 4.0933985 8.6520153 18.9346423
C 4.8984139 16.9876294 21.4933483
H 4.6048590 17.4180570 22.4474018
C 5.0538092 17.0854010 19.1119223
H 4.8896731 17.6104042 18.1694518
C 1.4425597 15.4164968 18.1598310
H 1.2667637 15.9466308 17.2232390
C 2.5149381 14.5605255 18.2712113
H 3.1924963 14.4066965 17.4351999
C 4.6776843 17.6850362 20.3249042
H 4.2144035 18.6701115 20.3448884
C 9.2775247 13.4892066 26.5821755
H 9.5889916 13.0301035 27.5213813
C 5.2971028 8.1607071 18.4022140
H 5.3024974 7.2947008 17.7428487
C 6.4737248 8.8092413 18.7191597
H 7.4190911 8.4653642 18.3053335
C 0.5722515 15.6219757 19.2534500
H -0.2734329 16.3027832 19.1534176
C 4.0805732 9.7497795 19.7682351
H 3.1435665 10.1304649 20.1675855
C 5.5134559 11.2477710 15.2397773
H 6.3413826 11.1110306 14.5425195
C 4.6801456 12.1220538 17.3802542
C 5.7576010 11.9328019 16.4556668
C 5.8653873 15.0714048 20.2722290
C 3.4048901 11.5982392 17.0490556
H 2.5929952 11.7280648 17.7604938
C 11.2356078 13.3162457 21.3600670
H 11.3189879 13.8237574 20.3987338
C 12.1125107 12.8568491 23.5601759
H 12.8732259 13.0071753 24.3259261
C 3.8666839 12.9806005 19.6302436
C 0.7997858 14.9712335 20.4468928
H 0.1415448 15.1357783 21.3012814
C 2.7642445 13.8611639 19.4781911
C 9.7370479 14.9222262 24.6989669
H 10.4175916 15.5709763 24.1498128
C 7.2430472 13.0824215 17.9867162
H 8.2085357 13.5262366 18.2081572
C 4.9201539 12.7885863 18.6106190
C 5.6224637 15.0418343 22.7594244
C 9.9413626 10.9951006 19.5425070
C 10.1552543 12.4587875 21.5950767
C 3.2497438 12.6049170 21.9625280
H 3.5033524 12.1367229 22.9062255
C 1.8944558 14.0864981 20.5964895
C 2.1739674 13.4487630 21.8321020
H 1.5241056 13.6467962 22.6850739
C 7.0318674 12.4459765 16.7851688
H 7.8464814 12.3385795 16.0678831
C 12.2138366 13.5104198 22.3328891
H 13.0549112 14.1746570 22.1316385
C 3.1991997 10.9359845 15.8607228
H 2.2118677 10.5368802 15.6260642
C 7.6040611 13.7922418 24.8572557
C 10.1320085 14.3658686 25.9146521
H 11.1166350 14.5901306 26.3243264

C	4.0805871	12.3448967	20.8540524
C	5.3175825	14.8574520	24.9781385
C	7.7317789	10.6188242	19.7808206
C	11.0036081	12.0551419	23.8217890
H	10.8790953	11.5993265	24.8018764
C	5.4776677	15.6981489	21.4973492
C	10.0273207	11.8645751	22.8493982
H	9.1413890	11.2851142	23.0702333
C	5.2733089	10.4363146	20.1191006
C	4.2610074	10.7582367	14.9445430
H	4.0853165	10.2313138	14.0063165
C	6.4909408	9.9382057	19.5632666
C	10.7485931	9.9913447	20.3442330
H	11.0217277	9.1518481	19.6951363
H	10.1587904	9.6197260	21.1877279
H	11.6538750	10.4612697	20.7398537
C	9.2887733	12.1060991	20.4215639
H	9.1581267	13.0173168	19.8252776
C	6.2113669	13.2277783	18.9385311
C	8.4841204	14.6297004	24.1692507
H	8.1759804	15.0626700	23.2199072
C	3.8708203	14.4468097	25.1516440
H	3.5001821	13.9913295	24.2323103
H	3.2585037	15.3245090	25.3890600
H	3.7896475	13.7181894	25.9607934
C	6.1626287	13.6741139	24.4413976
H	5.7403149	12.7339633	24.7959236
C	8.0201403	13.2051478	26.0534408
H	7.3339258	12.5508930	26.5884327
C	10.7211985	11.5476316	18.3713471
H	11.5782124	12.1161149	18.7474859
H	10.0883140	12.2012342	17.7646141
H	11.0939970	10.7286858	17.7449314
C	5.8535551	15.5655232	26.2003657
H	6.8717439	15.9256892	26.0317118
H	5.8645112	14.8686317	27.0445872
H	5.1995791	16.4101354	26.4469799
H	3.1494872	8.1653694	18.6856528
H	4.2166675	10.0120392	23.4306515
C	4.8287396	10.1478276	24.3267306
H	5.8192180	11.0308304	23.7216142
C	5.7122876	8.9487119	24.6366611
H	5.2984589	8.0726302	24.1146259
H	5.6830765	8.7175320	25.7096014
C	7.1625605	9.1296338	24.1768545
H	7.6657143	8.1556463	24.1026189
H	7.7219476	9.7444241	24.8958940
C	7.1765193	9.8364325	22.8300153
H	6.4674058	9.3437753	22.1446932
H	8.1564673	9.7702632	22.3437201
N	6.8132717	11.2212238	23.0156679
C	4.1246418	10.7926773	25.4200299
C	2.7224083	11.9702867	27.5881402
C	4.7571961	11.0463063	26.6586377
C	2.7667285	11.1648561	25.3061072
C	2.0775252	11.7318794	26.3716527
C	4.0715733	11.6272767	27.7184210
H	5.8039971	10.7712005	26.7784362
H	2.2422945	10.9647615	24.3720392
H	1.0244001	11.9906234	26.2544211
H	4.5911648	11.8134852	28.6595684
H	2.1839570	12.4215684	28.4209213

TS2-proR(boat)triplet (E_{tot}=-4161.53575)

Fe	6.4352134	12.3189610	21.6586331
C	5.2491321	15.8454096	19.3726007
H	5.5195816	15.5099444	18.3762665
O	8.7403909	10.1599907	18.9725457
O	5.2437915	15.3881054	24.1334962
N	6.2375842	13.8320460	20.3008485
N	7.9025045	11.5031966	20.5567688
N	5.0163618	11.3905966	20.7262604
N	6.2080293	13.6673606	23.0717461
C	4.1129642	8.5189135	18.6297155
C	4.4798355	16.7076730	21.8907479
H	4.1880167	17.0302503	22.8872483
C	4.5363391	17.0139504	19.5208280
H	4.2677847	17.5840019	18.6306273
C	1.6401654	15.2830041	17.4458410
H	1.5815696	15.8010212	16.4878554
C	2.6918362	14.4324112	17.6997647
H	3.4669030	14.2757707	16.9538068
C	4.1332118	17.4617584	20.7900045
H	3.5562484	18.3780099	20.9038432
C	9.7677895	14.0148918	26.4133171
H	10.2181816	13.6570426	27.3399083
C	5.3634634	7.9983434	18.2636375
H	5.4378413	7.1024876	17.6493935
C	6.5042393	8.6623473	18.6736307
H	7.4820430	8.3004025	18.3650043
C	0.6371294	15.4960729	18.4185501
H	-0.1911005	16.1724452	18.2054085
C	4.0250635	9.6483011	19.4161775
H	3.0583894	10.0489201	19.7064760
C	6.3485645	11.7185666	15.0493414
H	7.2639449	11.7866667	14.4595502
C	5.1257598	12.2311152	17.1163274
C	6.3224866	12.3152489	16.3341883
C	5.5988049	15.0163966	20.4793908
C	4.0046291	11.5539035	16.5759015
H	3.1034191	11.4907038	17.1812739
C	11.1971079	13.2046069	21.2793996
H	11.3602573	13.6418951	20.2941476
C	11.9069521	12.8742890	23.5606853
H	12.6156594	13.0588425	24.3670083
C	3.8712159	12.8778576	19.2444841
C	0.7136774	14.8556384	19.6354368
H	-0.0477192	15.0236156	20.3987081
C	2.7903792	13.7445904	18.9360662
C	9.8441090	15.4119136	24.4505402
H	10.3608648	16.1448072	23.8314668
C	7.3815844	13.5447620	18.1347813
H	8.2042091	14.1451428	18.5177055
C	5.0910659	12.8075772	18.4120953
C	5.5640568	14.8087177	22.9628328
C	9.9023259	10.8598706	19.4935343
C	10.0723321	12.4018165	21.4991283
C	2.9513046	12.5009216	21.4692938
H	3.0310992	12.0021431	22.4276717
C	1.7837705	13.9762139	19.9312067
C	1.9061810	13.3503558	21.1948709
H	1.1487326	13.5440673	21.9548256
C	7.4274420	13.0214270	16.8621728
H	8.3119526	13.1598218	16.2392993
C	12.1140898	13.4364017	22.3013249
H	12.9879849	14.0618033	22.1158136
C	4.0619945	10.9810961	15.3261613
H	3.1938973	10.4536974	14.9296392
C	7.9545640	13.9450697	24.8050819
C	10.4133250	14.9869953	25.6499649
H	11.3710100	15.3927829	25.9758632
C	3.9212145	12.2338827	20.4808284
C	5.4840399	14.3908767	25.1672428

C	7.6828195	10.5283121	19.7189960
C	10.7594982	12.1220604	23.7977812
H	10.5589949	11.7361491	24.7945313
C	5.2099126	15.5040412	21.7687514
C	9.8422398	11.8942985	22.7762312
H	8.9226213	11.3544677	22.9731934
C	5.1762820	10.3219639	19.9016661
C	5.2435900	11.0626259	14.5538512
H	5.2769074	10.6040702	13.5651649
C	6.4468018	9.8254300	19.4699545
C	10.6723414	9.8735116	20.3516476
H	10.9474522	9.0041252	19.7438903
H	10.0560187	9.5462764	21.1948232
H	11.5755067	10.3442508	20.7516314
C	9.2469455	12.0113092	20.3115830
H	9.1516763	12.8865556	19.6597027
C	6.2465721	13.3782997	18.9578037
C	8.6203419	14.8957854	24.0312301
H	8.1757596	15.2270875	23.0952027
C	4.1964032	13.6095806	25.3573360
H	3.9416991	13.0715211	24.4408273
H	3.3769090	14.2893156	25.6165498
H	4.3254477	12.8826283	26.1663319
C	6.5513715	13.5020063	24.4848622
H	6.4278728	12.4530635	24.7611634
C	8.5504011	13.4921264	25.9850764
H	8.0409619	12.7352894	26.5829972
C	10.7144135	11.3581534	18.3195765
H	11.5830701	11.9119634	18.6910410
H	10.1034612	12.0133529	17.6915585
H	11.0710074	10.5132172	17.7186586
C	5.8997091	15.1290763	26.4170865
H	6.7836729	15.7437484	26.2273445
H	6.1387062	14.4121774	27.2091980
H	5.0738408	15.7665651	26.7544276
H	3.1973739	8.0283258	18.2954247
C	4.7722119	10.0394977	24.4053694
H	5.5687518	10.7986305	23.6384382
C	5.5301229	8.7267222	24.4055414
H	5.0471908	8.0243566	23.7153394
H	5.4760908	8.2652272	25.4047291
C	6.9829557	8.9033516	23.9736859
H	7.4626854	7.9240422	23.8366386
H	7.5530811	9.4563923	24.7341492
C	7.0059804	9.6978573	22.6736567
H	6.3457423	9.2183937	21.9326328
H	8.0067296	9.6834505	22.2239541
N	6.6231283	11.0601353	22.9400049
C	3.4019375	10.0409922	23.8988890
C	0.7627392	10.0991348	22.8872101
C	2.3746045	10.7292122	24.5739119
C	3.0657930	9.3827538	22.6991791
C	1.7684195	9.4090266	22.2066131
C	1.0759912	10.7615831	24.0740853
H	2.6048821	11.2407781	25.5067789
H	3.8429576	8.8911718	22.1200446
H	1.5424858	8.9030144	21.2684577
H	0.3018302	11.3045581	24.6179110
H	-0.2510685	10.1300781	22.4888240
H	4.8843612	10.5981782	25.3416526

TS2-proS(chair)triplet (E_{tot}=-4161.54117)

Fe	6.6595613	12.3984215	21.6090314
C	6.1141101	16.1615428	19.4590640
H	6.3167066	15.8092524	18.4525191
O	8.7098593	9.4836366	19.6129353
O	5.0618375	15.3687703	24.0536039
N	6.4705964	13.9190129	20.3269571
N	7.9049055	11.3842005	20.4941171
N	5.1485007	11.4369807	20.7594701
N	5.7472223	13.5458830	22.9367218
C	4.0261283	8.0635178	19.8625359
C	5.4927637	17.1015174	21.9935100
H	5.2160915	17.4430775	22.9882339
C	5.8371805	17.4968575	19.6607298
H	5.8391989	18.1713348	18.8028387
C	1.9421681	15.5416887	17.5652810
H	1.9651826	16.1699557	16.6742601
C	2.9950151	14.6947589	17.8311573
H	3.8468636	14.6487067	17.1574388
C	5.5448743	17.9935472	20.9422308
H	5.3320805	19.0498765	21.0986709
C	7.9545995	11.8264753	26.9092443
H	7.9611465	11.0999435	27.7229372
C	5.2364467	7.3412434	19.8960376
H	5.2432338	6.2618493	19.7547527
C	6.4162097	8.0359120	20.0625027
H	7.3705220	7.5146388	20.0197965
C	0.8351253	15.6093758	18.4398924
H	0.0081161	16.2844114	18.2184851
C	4.0035846	9.4249795	20.0658507
H	3.0625734	9.9702328	20.0499628
C	6.4254470	12.0698575	14.9820803
H	7.3155651	12.1889743	14.3623358
C	5.2791741	12.4360766	17.1232315
C	6.4434766	12.5851690	16.3018093
C	6.1455440	15.2206439	20.5307473
C	4.1460885	11.7772231	16.5844785
H	3.2646244	11.6685137	17.2131167
C	10.9198229	13.4812775	19.6279294
H	11.0454552	13.1416702	18.6008462
C	11.4781749	15.0334852	21.3888414
H	12.0561927	15.8818590	21.7553733
C	4.0658298	12.9826039	19.2867075
C	0.8090323	14.8293265	19.5748570
H	-0.0347577	14.8819014	20.2645683
C	2.9920130	13.8667857	18.9821250
C	9.0659936	13.5110361	25.5947919
H	9.9517092	14.1089587	25.3828380
C	7.5698297	13.7053726	18.1336295
H	8.4218908	14.2385168	18.5499599
C	5.2839146	12.9515203	18.4490810
C	5.5473604	14.8414808	22.9131887
C	9.8557629	10.3890963	19.6055721
C	9.9644568	12.8596480	20.4426565
C	2.9469369	12.3451958	21.3507049
H	2.9774463	11.7764156	22.2732222
C	1.8765481	13.9494132	19.8780928
C	1.8888334	13.1726891	21.0617277
H	1.0515541	13.2561429	21.7560979
C	7.5670770	13.2646685	16.8297911
H	8.4355855	13.4293344	16.1902241
C	11.6814772	14.5480525	20.0965685
H	12.4182449	15.0164775	19.4435123
C	4.1614185	11.2830162	15.2991351
H	3.2830665	10.7726482	14.9031153
C	6.7828095	12.8952694	25.0740919
C	9.0923472	12.5906617	26.6441316
H	9.9936800	12.4657361	27.2439672
C	4.0348707	12.2313461	20.4565056
C	4.6323966	14.2461262	24.8761823
C	7.6751560	10.1316250	20.1843764
C	10.5140985	14.4362171	22.1976846

H 10.3266800 14.8217103 23.1975768

C 5.7662023 15.7280361 21.8145359

C 9.7699040 13.3531702 21.7322620

H 9.0153141 12.9189691 22.3817714

C 5.1893303 10.1546344 20.3799370

C 5.3096670 11.4302627 14.4884175

H 5.3094726 11.0359011 13.4719254

C 6.4305676 9.4314765 20.2752690

C 10.8231426 9.9633010 20.6919173

H 11.2193702 8.9673149 20.4624939

H 10.3295232 9.9267053 21.6641688

H 11.6514511 10.6781328 20.7433950

C 9.1566891 11.7530710 19.8372958

H 8.8666631 12.1074477 18.8386167

C 6.4492011 13.5034155 18.9740242

C 7.9194783 13.6680054 24.8195262

H 7.9068108 14.3698894 23.9889746

C 3.1613692 13.9978270 24.5893922

H 3.0180249 13.8141060 23.5220152

H 2.5695909 14.8717878 24.8853618

H 2.8184398 13.1205863 25.1492921

C 5.5011992 13.0977002 24.3092395

H 4.9329621 12.1646171 24.3015048

C 6.8094854 11.9871200 26.1343691

H 5.9217644 11.3940696 26.3340988

C 10.4911307 10.2846990 18.2331344

H 11.3716892 10.9338502 18.1854567

H 9.7788463 10.5793554 17.4549594

H 10.8135141 9.2530509 18.0497740

C 4.8760767 14.6089335 26.3226730

H 5.9275196 14.8577921 26.4895611

H 4.6162126 13.7568619 26.9597529

H 4.2464471 15.4631734 26.5979204

H 3.0873581 7.5379388 19.6800087

C 5.6722994 9.3151178 23.6261235

H 6.1361136 10.5474847 23.3535115

N 7.0395107 11.3527946 22.9973087

C 8.2547042 10.6617137 23.3159217

H 9.0368903 10.9061977 22.5911140

H 8.5869263 11.0357943 24.2997623

C 8.1141576 9.1368002 23.4043344

H 9.0447825 8.7225802 23.8171536

C 6.9182784 8.7708148 24.2672676

H 7.0612789 9.1946137 25.2689243

H 6.8474960 7.6751615 24.3790132

H 7.9796592 8.7295064 22.3961056

H 5.5222460 8.9442669 22.6114728

C 4.4281695 9.4867494 24.3592533

C 1.9734044 10.0186733 25.6668691

C 4.3391303 9.5079149 25.7657446

C 3.2349798 9.6841972 23.6326111

C 2.0326071 9.9549516 24.2712402

C 3.1324032 9.7742958 26.4063534

H 5.2218118 9.2945217 26.3646999

H 3.2785502 9.6153888 22.5481026

H 1.1320148 10.1172423 23.6775320

H 3.0939925 9.7859006 27.4966009

H 1.0319436 10.2347712 26.1716926

TS2-proR(chair)triplet (E_{tot}=-4161.5313)

Fe 6.4397570 12.2506124 21.7414758

C 5.1462096 15.7812624 19.5058007

H 5.4139856 15.4638293 18.5029624

O 8.6865579 10.0395554 19.0447888

O 5.2548766 15.2906737 24.2627304

N 6.2233661 13.8007137 20.4078399

N 7.8787448 11.4545704 20.5858051

N 5.0067470 11.3580160 20.8066244

N 6.2541796 13.6188124 23.1559618

C 4.0591322 8.4016311 18.8529910

C 4.3750559 16.5930386 22.0417800

H 4.0865432 16.8962034 23.0454672

C 4.3888399 16.9183307 19.6734604

H 4.0837037 17.4819616 18.7911263

C 1.7104938 15.1456479 17.3339521

H 1.6746236 15.6349187 16.3599563

C 2.7587245 14.3074937 17.6394291

H 3.5528079 14.1339455 16.9178043

C 3.9840210 17.3401813 20.9516813

H 3.3729424 18.2321181 21.0794891

C 9.8921766 14.0970093 26.3974031

H 10.3861248 13.7482252 27.3051590

C 5.3027514 7.8374524 18.5288359

H 5.3641877 6.9004777 17.9776311

C 6.4526420 8.5101453 18.8958079

H 7.4258976 8.1187331 18.6094133

C 0.6813018 15.3815845 18.2731945

H -0.1432646 16.0484754 18.0196189

C 3.9861238 9.5815237 19.5637927

H 3.0245923 10.0110211 19.8280977

C 6.5571194 11.7511135 15.1458325

H 7.4773906 11.8798312 14.5740198

C 5.2605082 12.1820095 17.1859650

C 6.4656919 12.3453957 16.4288762

C 5.5412303 14.9569702 20.6012470

C 4.1965327 11.4346187 16.6230962

H 3.2891606 11.3134938 17.2101954

C 11.1344848 13.2837476 21.1181425

H 11.2443834 13.6856803 20.1107142

C 11.9290708 13.0850492 23.3872554

H 12.6502341 13.3400751 24.1625283

C 3.9080510 12.8098665 19.2640505

C 0.7283771 14.7760424 19.5091205

H -0.0532545 14.9619408 20.2472077

C 2.8285527 13.6569398 18.8975081

C 9.8422068 15.5252959 24.4561387

H 10.3032573 16.2933442 23.8354171

C 7.4068083 13.6326860 18.2543689

H 8.1779355 14.2850807 18.6613671

C 5.1605881 12.7543097 18.4800957

C 5.5620623 14.7341779 23.0772453

C 9.8583214 10.7590295 19.5172047

C 10.0601049 12.4360803 21.4076717

C 2.9282540 12.4938823 21.4734164

H 2.9846243 12.0253001 22.4485716

C 1.7941200 13.9110384 19.8581364

C 1.8861196 13.3270568 21.1440316

H 1.1076730 13.5398709 21.8767557

C 7.5084028 13.1272659 16.9772969

H 8.3891472 13.3362154 16.3689223

C 12.0682090 13.6047311 22.1006017

H 12.9025078 14.2654404 21.8626479

C 4.3187210 10.8635336 15.3770104

H 3.4963469 10.2786764 14.9639082

C 8.0374483 13.9622920 24.8409654

C 10.4673617 15.1103569 25.6312089

H 11.4128356 15.5587265 25.9367173

C 3.9237594 12.2038382 20.5190526

C 5.5629914 14.2904898 25.2769014

C 7.6454284 10.4403327 19.7988191

C 10.8341800 12.2796852 23.6905119

H 10.6891869 11.9202341 24.7062650

C 5.1503985 15.4205281 21.8989873

C 9.9000277 11.9646781 22.7088107
H 9.0269094 11.3687075 22.9457934
C 5.1473822 10.2568831 20.0225415
C 5.5085866 11.0222579 14.6295539
H 5.5931096 10.5653146 13.6431667
C 6.4090258 9.7211002 19.6182488
C 10.6314406 9.8178406 20.4214889
H 10.8967804 8.9145332 19.8604497
H 10.0231650 9.5416735 21.2882437
H 11.5409414 10.3059727 20.7846286
C 9.2130627 11.9551571 20.2727946
H 9.0901360 12.7755733 19.5575937
C 6.2743035 13.3748200 19.0581104
C 8.6330360 14.9561965 24.0638988
H 8.1454726 15.2792477 23.1465332
C 4.3166836 13.4496400 25.4800284
H 4.0625942 12.9307451 24.5525213
H 3.4746544 14.0826998 25.7816172
H 4.5003447 12.7025634 26.2600487
C 6.6481537 13.4569462 24.5550137
H 6.5759622 12.3970424 24.8146063
C 8.6886932 13.5224706 25.9965342
H 8.2338001 12.7332408 26.5968723
C 10.6631970 11.1921876 18.3131634
H 11.5265518 11.7756460 18.6505447
H 10.0454810 11.8043256 17.6490636
H 11.0270253 10.3165103 17.7628001
C 5.9762821 15.0279045 26.5274791
H 6.8272550 15.6839978 26.3264313
H 6.2658204 14.3105605 27.3019080
H 5.1319237 15.6230828 26.8948381
H 3.1370105 7.9038373 18.5486270
C 4.6620156 9.8727352 24.3124200
H 5.5146128 10.6840858 23.6654711
N 6.6188151 11.0020308 23.0384837
C 3.2848187 9.9944583 23.8397074
C 0.6171024 10.2759924 22.9344051
C 2.3429919 10.7637364 24.5536709
C 2.8363760 9.3466486 22.6723309
C 1.5279593 9.4875830 22.2290032
C 1.0339049 10.9088590 24.1060425
H 2.6486720 11.2477479 25.4790171
H 3.5292071 8.7513955 22.0867974
H 1.2182977 8.9883294 21.3106827
H 0.3320846 11.5162093 24.6792536
H -0.4036982 10.3978476 22.5731360
H 4.7832528 10.2820747 25.3219989
C 7.2310395 9.7028290 22.9227847
H 7.8931890 9.6539478 22.0532678
H 7.8566434 9.5384382 23.8249774
C 6.2314477 8.5461619 22.8493078
H 6.7794153 7.5982171 22.7453755
C 5.3739141 8.5452048 24.1059183
H 6.0251272 8.3436390 24.9687488
H 4.6368377 7.7296187 24.0554411
H 5.6197813 8.6754150 21.9527272

Int2-proS(boat)triplet (E_{tot}=--4161.55648)

Fe 6.3128334 12.4783815 21.5863393
C 6.0623486 15.8381204 18.8416822
H 6.0603500 15.2978370 17.8999827
O 8.8320773 10.4203306 19.0013110
O 5.5070385 15.9914979 23.5708253
N 6.1510482 13.7340401 20.0586972
N 7.8732269 11.6226580 20.6242624
N 4.9837065 11.2718255 20.7212083
N 6.0097859 13.9431249 22.8151122
C 4.3534980 8.4737199 18.4363624
C 5.9730107 17.2681691 21.2172455
H 5.9083367 17.8085127 22.1589578
C 6.0315636 17.2161965 18.8288568
H 6.0195614 17.7332606 17.8677750
C 1.2194511 15.3340736 18.1343112
H 1.0824704 15.9700240 17.2591367
C 2.3027787 14.4872097 18.2026985
H 3.0257446 14.4406681 17.3912376
C 6.0042234 17.9572071 20.0229778
H 5.9819126 19.0455896 20.0078315
C 8.4338331 13.2031805 26.9677152
H 8.5006341 12.6561517 27.9084296
C 5.6517929 8.0663166 18.0839940
H 5.8104248 7.2173031 17.4211978
C 6.7241942 8.7827971 18.5735150
H 7.7353343 8.5064756 18.2837688
C 0.2828159 15.3896475 19.1917905
H -0.5717763 16.0631796 19.1239758
C 4.1565913 9.5404556 19.2835272
H 3.1495396 9.8556853 19.5475336
C 5.3749166 11.2362071 15.0342929
H 6.2252650 11.1059697 14.3633941
C 4.4783245 12.0590359 17.1675177
C 5.5842480 11.8859071 16.2755141
C 6.0984801 15.0878080 20.0566906
C 3.2085542 11.5648072 16.7781347
H 2.3731920 11.6861484 17.4636044
C 10.9345371 13.5568635 21.6200520
H 11.0451335 14.1521837 20.7129045
C 11.6608590 12.9909325 23.8519055
H 12.3278246 13.1518639 24.6984076
C 3.6128956 12.7806006 19.4379015
C 0.4548158 14.5991643 20.3073431
H -0.2584515 14.6422378 21.1319077
C 2.5018770 13.6528752 19.3326256
C 9.3847416 14.7003669 25.3348404
H 10.2131764 15.3124521 24.9835343
C 7.0260834 12.9798647 17.8836074
H 7.9913313 13.3844256 18.1671260
C 4.6834218 12.6902210 18.4247622
C 5.8628022 15.2189045 22.5308854
C 9.9256798 11.1917231 19.5622673
C 9.9361336 12.5814010 21.6856227
C 2.8791452 12.1039984 21.6573139
H 3.0631022 11.5040855 22.5433398
C 1.5609756 13.7213735 20.4134678
C 1.7801260 12.9261152 21.5648377
H 1.0655599 12.9844821 22.3869580
C 6.8549308 12.3668472 16.6657090
H 7.6992927 12.2439512 15.9866094
C 11.7985910 13.7575524 22.6955014
H 12.5795347 14.5159028 22.6303185
C 3.0356641 10.9409550 15.5636416
H 2.0514100 10.5655823 15.2816756
C 7.1726785 13.7905469 24.9861121
C 9.4829150 14.0200934 26.5487950
H 10.3835962 14.1134118 27.1562778
C 3.8068258 12.0167007 20.5921864
C 5.0354659 15.0801010 24.6094974
C 7.7332422 10.6688824 19.7418714
C 10.6339833 12.0522778 23.9398992
H 10.4865620 11.4907407 24.8610119
C 6.0156584 15.8571092 21.2622245
C 9.7768952 11.8520735 22.8634672
H 8.9772365 11.1240546 22.9159525

C	5.2379489	10.2764484	19.8438226
C	4.1271938	10.7729125	14.6817281
H	3.9784979	10.2734987	13.7239765
C	6.5594600	9.8926200	19.4333387
C	10.7749905	10.2229735	20.3654898
H	11.1396368	9.4326635	19.6995485
H	10.1759594	9.7734196	21.1634841
H	11.6241416	10.7416531	20.8206202
C	9.1744438	12.2410664	20.4385724
H	9.0337740	13.1692628	19.8655079
C	5.9531097	13.1297333	18.7896348
C	8.2441634	14.5729329	24.5488304
H	8.1817034	15.0903169	23.5945875
C	3.5498925	14.8653740	24.3693165
H	3.3826436	14.3390193	23.4245293
H	3.0440045	15.8368983	24.3329979
H	3.1276215	14.2789844	25.1896402
C	5.8472870	13.8115562	24.2685736
H	5.2770436	12.9085296	24.4951597
C	7.2804976	13.0971629	26.1927886
H	6.4475913	12.4942343	26.5389919
C	10.7053636	11.8248038	18.4313632
H	11.4939443	12.4573430	18.8545728
H	10.0493038	12.4380552	17.8066041
H	11.1729839	11.0535837	17.8079330
C	5.3129398	15.7041486	25.9552267
H	6.3675755	15.9771380	26.0452795
H	5.0748430	14.9778154	26.7385803
H	4.6878478	16.5950912	26.0869124
H	3.4874046	7.9442895	18.0366509
H	3.9522042	10.5553027	24.1934248
C	4.5939435	10.0836398	24.9302662
H	6.9732176	11.6350181	23.8200386
C	5.2872794	8.8299197	24.5494147
H	4.7278520	8.3279279	23.7474773
H	5.3177937	8.1368547	25.4037437
C	6.7223118	9.0612015	24.0463962
H	7.1889201	8.0862465	23.8386614
H	7.3156853	9.5479848	24.8341718
C	6.7506577	9.9023942	22.7803708
H	6.0138167	9.5180862	22.0680940
H	7.7279378	9.7531281	22.2832834
N	6.4791567	11.2950209	22.9971946
C	4.6652309	10.6792786	26.2077492
C	4.7951245	11.9773602	28.7303589
C	5.5270023	10.2111476	27.2395345
C	3.8548808	11.8088810	26.5106395
C	3.9223022	12.4439165	27.7382970
C	5.5853890	10.8516298	28.4682091
H	6.1694210	9.3535348	27.0514860
H	3.1668242	12.1636033	25.7456008
H	3.2913828	13.3115962	27.9339589
H	6.2668401	10.4801436	29.2348377
H	4.8564307	12.4822137	29.6937599

Int2-proR(boat)triplet (E_{tot}=-4161.55316)

Fe	6.5042612	12.4000730	21.6058932
C	5.3288948	15.8827854	19.3153290
H	5.6717733	15.5784524	18.3310071
O	8.8345175	10.1452233	18.9941519
O	5.1470827	15.3573561	24.0686519
N	6.2983249	13.8642378	20.2434364
N	7.9728160	11.5120655	20.5461196
N	5.0888701	11.4232557	20.6638551
N	6.2419907	13.7150566	23.0137476
C	4.1868822	8.5751067	18.5371820
C	4.3933519	16.6689156	21.8024384
H	4.0423392	16.9642661	22.7883411
C	4.5751222	17.0275613	19.4463025
H	4.3459836	17.6131687	18.5553566
C	1.5829115	15.4088620	17.6460008
H	1.4911926	15.9683868	16.7143331
C	2.6496679	14.5596240	17.8317961
H	3.4060379	14.4429904	17.0595503
C	4.0804061	17.4310450	20.6979025
H	3.4657382	18.3242625	20.7954363
C	9.8176106	14.4341204	26.2723734
H	10.2856015	14.1864656	27.2257525
C	5.4392422	8.0477725	18.1836019
H	5.5154307	7.1566229	17.5627071
C	6.5784003	8.6987701	18.6168874
H	7.5576219	8.3287247	18.3226088
C	0.6075316	15.5686144	18.6564465
H	-0.2326627	16.2451647	18.4976989
C	4.0958953	9.6971825	19.3321442
H	3.1270860	10.1018246	19.6105973
C	6.2101616	11.7142080	15.0067308
H	7.1217040	11.7180218	14.4071896
C	5.0505730	12.3081577	17.0889948
C	6.2416258	12.3074787	16.2930515
C	5.6306808	15.0359783	20.4214557
C	3.8803509	11.7060239	16.5625586
H	2.9841375	11.7002440	17.1786282
C	11.2794696	13.0880052	21.4528643
H	11.5300214	13.5110285	20.4797295
C	11.7995352	12.7636499	23.7854206
H	12.4483065	12.9389857	24.6425124
C	3.8854799	12.9488115	19.2662437
C	0.7246552	14.8749891	19.8405640
H	-0.0164966	15.0008529	20.6314552
C	2.7904352	13.8191259	19.0327002
C	9.8453410	15.6094030	24.1689585
H	10.3406469	16.2795332	23.4667351
C	7.4120903	13.4498699	18.0820870
H	8.2892642	13.9673773	18.4621887
C	5.0733082	12.8817851	18.3860296
C	5.5239506	14.8120264	22.8989770
C	9.9934042	10.8067830	19.5648348
C	10.1129365	12.3288687	21.5950969
C	3.0229488	12.4450993	21.4912403
H	3.1280701	11.8821359	22.4123911
C	1.8099148	13.9930969	20.0658288
C	1.9646164	13.2990343	21.2901589
H	1.2205813	13.4394467	22.0743727
C	7.4066204	12.9160396	16.8132738
H	8.2996323	12.9664611	16.1890466
C	12.1239459	13.2990459	22.5390228
H	13.0304082	13.8925998	22.4159160
C	3.8822210	11.1357407	15.3104926
H	2.9751029	10.6698579	14.9243110
C	7.9903953	14.1543150	24.7035894
C	10.4366055	15.3274919	25.3986596
H	11.3914982	15.7808689	25.6644535
C	3.9749378	12.2474791	20.4694603
C	5.4757819	14.3841792	25.1031735
C	7.7584559	10.5380702	19.7048711
C	10.6147943	12.0487643	23.9395332
H	10.3291295	11.6765803	24.9214482
C	5.1716175	15.4937329	21.6990004
C	9.7723774	11.8336218	22.8519982
H	8.8240843	11.3197004	22.9803278
C	5.2460224	10.3612755	19.8378490
C	5.0563777	11.1390541	14.5227614

H 5.0456309 10.6827825 13.5325093
C 6.5199903 9.8566720 19.4217909
C 10.6914412 9.7958261 20.4562084
H 10.9644224 8.9169528 19.8611352
H 10.0242461 9.4920572 21.2691511
H 11.5907526 10.2356111 20.8980906
C 9.3413911 11.9761622 20.3589614
H 9.3113897 12.8630270 19.7140155
C 6.2697387 13.3900320 18.9067241
C 8.6256660 15.0304026 23.8247083
H 8.1653685 15.2467247 22.8631242
C 4.2676979 13.4814217 25.2894180
H 4.0509616 12.9424958 24.3632860
H 3.3900683 14.0745110 25.5696380
H 4.4731557 12.7520238 26.0807059
C 6.6188136 13.5956924 24.4222979
H 6.5984384 12.5430302 24.7160299
C 8.6081323 13.8444002 25.9184822
H 8.1277082 13.1388211 26.5971566
C 10.8717486 11.2787895 18.4281323
H 11.7400649 11.8063368 18.8369600
H 10.3108527 11.9528867 17.7726845
H 11.2279965 10.4231144 17.8423600
C 5.8132322 15.1610799 26.3526559
H 6.6505294 15.8404043 26.1719134
H 6.0891967 14.4729559 27.1579551
H 4.9350856 15.7369045 26.6681083
H 3.2726480 8.0943944 18.1848355
C 4.5309258 9.4635936 24.7439815
H 5.6936598 11.1262785 23.4646018
C 5.5144883 8.3946369 24.4347793
H 5.1130311 7.7092616 23.6782545
H 5.6652093 7.7926667 25.3486338
C 6.8928231 8.8837546 23.9629883
H 7.5287381 7.9990065 23.8100666
H 7.3684651 9.4956770 24.7429605
C 6.8328246 9.6938968 22.6668877
H 6.0874270 9.2383177 21.9939225
H 7.7933825 9.6202505 22.1473693
N 6.5561790 11.0849010 22.9162248
C 3.3249565 9.7139414 24.0501344
C 0.8398658 10.1994859 22.7705241
C 2.3494973 10.5844606 24.6168164
C 3.0096997 9.1241586 22.7942950
C 1.7922486 9.3632034 22.1792096
C 1.1354767 10.8146171 23.9926254
H 2.5686494 11.0630042 25.5703030
H 3.7448275 8.5057944 22.2866044
H 1.5877197 8.9086034 21.2106233
H 0.4090662 11.4835628 24.4554286
H -0.1082195 10.3935455 22.2708774
H 4.7180464 10.0560117 25.6420131

Int2-proS(chair)triplet (E_{tot}=-4161.56008)

Fe 6.6270070 12.4316568 21.5679039
C 6.1105243 16.1687338 19.4073852
H 6.2363445 15.8037218 18.3930696
O 8.7477304 9.5180958 19.6578410
O 5.1642867 15.4275053 24.0135661
N 6.4472153 13.9243570 20.2763061
N 7.9014540 11.4253791 20.4811200
N 5.1304489 11.4195670 20.6993040
N 5.8405894 13.5785002 22.9321208
C 4.0930077 7.9794837 19.9467745
C 5.6727762 17.1434829 21.9703553
H 5.4654741 17.5000863 22.9766254
C 5.9046146 17.5129915 19.6193853
H 5.8838245 18.1846748 18.7595117
C 1.8856697 15.5404432 17.5723642
H 1.9051343 16.1878476 16.6949986
C 2.9467089 14.6991454 17.8241452
H 3.8013282 14.6762320 17.1525883
C 5.7094500 18.0279445 20.9152392
H 5.5553513 19.0935287 21.0768172
C 7.8219119 11.7228318 26.9556534
H 7.7673371 10.9882102 27.7598726
C 5.3257333 7.2992817 19.9849727
H 5.3667200 6.2173677 19.8700442
C 6.4857202 8.0339067 20.1265155
H 7.4533896 7.5375258 20.0915553
C 0.7745956 15.5773545 18.4436364
H -0.0587877 16.2482379 18.2337540
C 4.0325598 9.3455303 20.1149936
H 3.0752955 9.8618309 20.0910691
C 6.3679157 12.1027501 14.9227268
H 7.2543386 12.2269580 14.2987163
C 5.2352323 12.4506293 17.0741782
C 6.3941631 12.6062673 16.2467499
C 6.1711006 15.2299093 20.4832448
C 4.0992130 11.7958608 16.5369190
H 3.2219828 11.6805266 17.1702355
C 10.8436744 13.6081461 19.6587547
H 10.9557995 13.3268414 18.6121085
C 11.4002069 15.0705827 21.4951247
H 11.9621301 15.9134839 21.8975733
C 4.0310555 12.9674662 19.2463076
C 0.7523472 14.7712639 19.5605835
H -0.0954800 14.7983782 20.2469243
C 2.9486022 13.8460251 18.9567162
C 9.0688324 13.3371894 25.6723575
H 9.9992029 13.8686509 25.4758869
C 7.5302693 13.7142026 18.0788871
H 8.3804570 14.2523752 18.4925644
C 5.2471223 12.9546862 18.4054260
C 5.6558000 14.8831689 22.8853425
C 9.8680524 10.4507093 19.6131671
C 9.9281509 12.9137245 20.4585703
C 2.9045447 12.2631149 21.2850741
H 2.9368303 11.6691048 22.1920124
C 1.8274554 13.8955600 19.8483505
C 1.8402188 13.0876425 21.0115310
H 0.9968597 13.1444069 21.7015081
C 7.5213492 13.2817094 16.7729359
H 8.3853295 13.4523966 16.1288738
C 11.5842161 14.6698947 20.1713970
H 12.2898243 15.1993921 19.5306543
C 4.1060181 11.3136111 15.2467828
H 3.2250289 10.8067972 14.8520744
C 6.7594271 12.8878622 25.1163273
C 9.0105869 12.4123886 26.7161774
H 9.8910266 12.2217134 27.3297335
C 4.0034201 12.1863233 20.3970093
C 4.6705938 14.3298781 24.8270588
C 7.6940356 10.1593101 20.2078365
C 10.4777396 14.3955633 22.2922956
H 10.3126934 14.7146012 23.3191981
C 5.8787252 15.7565938 21.7837185
C 9.7530226 13.3199977 21.7814899
H 9.0320566 12.8138432 22.4189855
C 5.1969686 10.1199195 20.3937489
C 5.2487221 11.4682080 14.4300617
H 5.2420339 11.0831437 13.4100140
C 6.4623100 9.4333403 20.3092146

C 10.8639556 10.0799119 20.6939060
H 11.2354780 9.0640060 20.5173845
H 10.4068843 10.1203717 21.6835084
H 11.7049071 10.7810825 20.6704310
C 9.1450388 11.8062416 19.8254143
H 8.8644555 12.1584105 18.8237391
C 6.4149162 13.5048448 18.9247745
C 7.9501103 13.5740968 24.8764856
H 7.9987415 14.2745857 24.0466695
C 3.2056169 14.1194001 24.4822154
H 3.1006296 13.8983991 23.4162995
H 2.6325636 15.0227556 24.7203911
H 2.8093792 13.2751439 25.0574283
C 5.5224899 13.1520684 24.2991839
H 4.8936271 12.2545992 24.2739505
C 6.7036600 11.9651071 26.1615185
H 5.7774177 11.4223901 26.3340042
C 10.4865049 10.3410917 18.2334555
H 11.3153976 11.0521429 18.1476257
H 9.7404272 10.5599531 17.4618825
H 10.8791525 9.3298875 18.0748148
C 4.8731552 14.6922935 26.2791703
H 5.9253595 14.9104979 26.4819666
H 4.5647369 13.8521270 26.9102263
H 4.2606394 15.5667040 26.5277647
H 3.1695539 7.4200084 19.7887615
C 5.6064488 8.8462195 23.6918054
H 6.2407408 11.0978339 23.6071899
N 6.9211409 11.1398065 22.8604317
C 8.1444836 10.5382437 23.3067704
H 8.9328498 10.8053511 22.6026180
H 8.4373417 10.9506463 24.2870381
C 8.0827571 9.0120888 23.4108540
H 9.0371797 8.6446595 23.8182368
C 6.9287046 8.5510594 24.2976862
H 7.0283969 9.0148655 25.2875826
H 7.0238809 7.4589256 24.4521248
H 7.9603637 8.5889726 22.4082233
H 5.5142310 8.6842708 22.6215024
C 4.4543879 9.2745339 24.3873742
C 2.1248102 10.2808201 25.6684984
C 4.3894947 9.4272835 25.8010336
C 3.2769770 9.5947934 23.6539975
C 2.1476451 10.0937854 24.2791884
C 3.2521338 9.9264449 26.4188845
H 5.2434622 9.1402281 26.4109066
H 3.2943448 9.4492693 22.5760916
H 1.2710181 10.3476796 23.6817124
H 3.2366883 10.0376897 27.5043341
H 1.2370987 10.6788407 26.1593256

Int2-proR(chair)triplet (E_{tot}=-4161.55138)

Fe 6.5429728 12.3334890 21.6979720
C 5.2324431 15.8255532 19.4942026
H 5.5682060 15.5483835 18.4996837
O 8.7715326 9.9996029 19.0707368
O 5.1747984 15.2298722 24.2407094
N 6.3024475 13.8386772 20.3834783
N 7.9583461 11.4490381 20.5765384
N 5.0944201 11.3913589 20.7671801
N 6.2850803 13.6360739 23.1275923
C 4.1147463 8.4112034 18.8676505
C 4.3010627 16.5373454 22.0066410
H 3.9542947 16.8030406 23.0025802
C 4.4330067 16.9350921 19.6534316
H 4.1627742 17.5196984 18.7735541
C 1.6841431 15.2554006 17.5012787
H 1.6275183 15.7876191 16.5509744
C 2.7511488 14.4246931 17.7573076
H 3.5412142 14.2975227 17.0214422
C 3.9416125 17.3017405 20.9181151
H 3.2926891 18.1678045 21.0366734
C 9.9457687 14.4948446 26.2533890
H 10.4666640 14.2535916 27.1806621
C 5.3550341 7.8397187 18.5385649
H 5.4091658 6.8962012 17.9977415
C 6.5093748 8.5094306 18.8951921
H 7.4795292 8.1062413 18.6140809
C 0.6627831 15.4300477 18.4624638
H -0.1769032 16.0918458 18.2483777
C 4.0499645 9.5985620 19.5648514
H 3.0902461 10.0287781 19.8367108
C 6.4800091 11.7732766 15.1203405
H 7.3991325 11.8576228 14.5384851
C 5.2312815 12.2593931 17.1783011
C 6.4344419 12.3644964 16.4072545
C 5.5859936 14.9766157 20.5834679
C 4.1263756 11.5639083 16.6269592
H 3.2217586 11.4817519 17.2251254
C 11.2326555 13.2031393 21.2058567
H 11.4038117 13.5798957 20.1970662
C 11.8959566 13.0495667 23.5199473
H 12.5765624 13.3139790 24.3279815
C 3.9446948 12.8752758 19.2958738
C 0.7344290 14.7688816 19.6684395
H -0.0429621 14.9056528 20.4218197
C 2.8479679 13.7205611 18.9840377
C 9.8172080 15.7167161 24.1801438
H 10.2431544 16.4302290 23.4750065
C 7.4655796 13.5900851 18.2263118
H 8.2793530 14.1899505 18.6295817
C 5.1759976 12.8288876 18.4764059
C 5.5303679 14.7117926 23.0514832
C 9.9467266 10.6949278 19.5668746
C 10.1264485 12.3846734 21.4567539
C 2.9860483 12.4202679 21.4914828
H 3.0522868 11.8856276 22.4330137
C 1.8190282 13.9078072 19.9659741
C 1.9259057 13.2507835 21.2154225
H 1.1438096 13.4009592 21.9594750
C 7.5276498 13.0791995 16.9490575
H 8.4165788 13.2311338 16.3354898
C 12.1175041 13.5310071 22.2298068
H 12.9759221 14.1709174 22.0227717
C 4.2043461 10.9949814 15.3764710
H 3.3493733 10.4523670 14.9720412
C 8.0709998 14.1465774 24.7553992
C 10.4761016 15.4409923 25.3767411
H 11.4141664 15.9427350 25.6143312
C 3.9864652 12.2115449 20.5204082
C 5.5676468 14.2504599 25.2474192
C 7.7200042 10.4282994 19.7984071
C 10.7723017 12.2694934 23.7805775
H 10.5671996 11.9360293 24.7953919
C 5.1238328 15.3962693 21.8726139
C 9.8876582 11.9430654 22.7568212
H 8.9880684 11.3715049 22.9615230
C 5.2178861 10.2803206 20.0036801
C 5.3903321 11.1004246 14.6136650
H 5.4398148 10.6452251 13.6241085
C 6.4766214 9.7287760 19.6048560
C 10.6713419 9.7473679 20.5045281
H 10.9310395 8.8304617 19.9632965

H 10.0306132 9.4984694 21.3559773

H 11.5815607 10.2183335 20.8884011

C 9.3114598 11.9133787 20.2931185

H 9.2329220 12.7331232 19.5702351

C 6.3242957 13.4030226 19.0345846

C 8.6185811 15.0765237 23.8723080

H 8.1076636 15.2869279 22.9353161

C 4.3996628 13.2984186 25.4434279

H 4.1802315 12.7735590 24.5095232

H 3.5074641 13.8512228 25.7576570

H 4.6508383 12.5593738 26.2127063

C 6.7196023 13.5213654 24.5190934

H 6.7595521 12.4652048 24.7988583

C 8.7558206 13.8463867 25.9364253

H 8.3436151 13.1016150 26.6182708

C 10.7944264 11.0943955 18.3804031

H 11.6625145 11.6614833 18.7337006

H 10.2101396 11.7132633 17.6918752

H 11.1516365 10.2040752 17.8495244

C 5.9097157 15.0135964 26.5036723

H 6.7121342 15.7314737 26.3137116

H 6.2387708 14.3198386 27.2839108

H 5.0187140 15.5449732 26.8585910

H 3.1891590 7.9109744 18.5773318

C 4.3632247 9.3882451 24.5175608

H 5.8046730 11.0305214 23.5746113

N 6.6535589 11.0218052 23.0065181

C 3.1053703 9.6990856 23.9513512

C 0.5711583 10.3595300 22.8662370

C 2.2427401 10.6352322 24.5884346

C 2.6364754 9.1010255 22.7495802

C 1.3985701 9.4327374 22.2242106

C 1.0049087 10.9549897 24.0565062

H 2.5719563 11.1022264 25.5152587

H 3.2680522 8.3957673 22.2173803

H 1.0752818 8.9757706 21.2887412

H 0.3690173 11.6800497 24.5658157

H -0.3931102 10.6267898 22.4358651

H 4.6486539 9.9350671 25.4172009

C 7.1311244 9.6661136 22.9285350

H 7.8366276 9.5945063 22.0974899

H 7.7139180 9.4293159 23.8445024

C 6.0631367 8.5872305 22.7608273

H 6.5520390 7.6520346 22.4485344

C 5.2586865 8.2982707 24.0400862

H 5.9655242 8.0333037 24.8399645

H 4.6468389 7.4012468 23.8464544

H 5.3905186 8.8837883 21.9546425

TS3-S-triplet (E_{tot}=-4161.55485)

Fe 6.3641277 12.4493610 21.7129179

C 6.1877235 15.7833432 18.9221484

H 6.1082265 15.2397530 17.9858190

O 8.7355908 10.3848308 19.0263225

O 5.6852645 16.0109100 23.6414539

N 6.1806743 13.6869732 20.1611037

N 7.8720093 11.6140449 20.6828347

N 4.9586998 11.2687650 20.9481269

N 6.1301155 13.9341187 22.9226055

C 4.2359411 8.4079745 18.7662110

C 6.2714373 17.2355849 21.2840350

H 6.2683876 17.7861457 22.2222484

C 6.2525656 17.1600353 18.8965148

H 6.2407099 17.6689109 17.9309739

C 1.3067862 15.3525786 18.2266697

H 1.1982160 15.9704597 17.3346442

C 2.3603414 14.4714910 18.3248967

H 3.0867191 14.3814214 17.5202133

C 6.3180904 17.9115752 20.0830333

H 6.3725670 18.9986806 20.0572689

C 8.3244788 13.1044167 27.1912464

H 8.3294718 12.5313299 28.1191475

C 5.5139532 8.0036774 18.3466770

H 5.6413714 7.1432081 17.6918475

C 6.6073035 8.7379090 18.7587961

H 7.6018017 8.4625192 18.4155605

C 0.3645649 15.4674876 19.2745014

H -0.4652780 16.1687270 19.1829194

C 4.0814749 9.4922277 19.6012013

H 3.0886772 9.8048423 19.9179399

C 5.2288022 11.0501494 15.2304994

H 6.0532486 10.9020602 14.5314041

C 4.4103034 11.9434774 17.3668393

C 5.4791004 11.7499609 16.4361562

C 6.2178739 15.0399586 20.1427121

C 3.1298668 11.4287546 17.0456407

H 2.3208690 11.5700712 17.7584936

C 10.9469487 13.7381865 21.2079620

H 10.9834921 14.1924132 20.2175456

C 11.8242294 13.5372887 23.4486092

H 12.5457038 13.8293460 24.2118540

C 3.6109432 12.7624074 19.6197578

C 0.5002045 14.6988795 20.4100396

H -0.2176327 14.7852684 21.2272832

C 2.5248230 13.6623866 19.4788365

C 9.3968357 14.6256814 25.6570853

H 10.2543740 15.2324203 25.3712020

C 6.9647520 12.9292076 17.9407407

H 7.9294632 13.3779445 18.1480636

C 4.6612477 12.6159027 18.5950062

C 6.0342138 15.2108755 22.6171441

C 9.8538472 11.1705700 19.5186148

C 9.9738309 12.7723673 21.4876568

C 2.8351531 12.1592268 21.8355762

H 2.9558629 11.5691294 22.7366954

C 1.5760560 13.7881250 20.5459593

C 1.7577204 13.0054873 21.7105642

H 1.0313603 13.0893471 22.5201698

C 6.7525487 12.2758099 16.7512336

H 7.5668247 12.1579766 16.0352989

C 11.8699728 14.1174176 22.1804150

H 12.6257350 14.8682200 21.9474130

C 2.9151277 10.7570747 15.8635269

H 1.9238954 10.3638874 15.6353462

C 7.1922216 13.7716661 25.1572171

C 9.4137795 13.9081584 26.8535660

H 10.2789002 13.9676607 27.5149650

C 3.7879310 12.0293739 20.7958935

C 5.1263671 15.1289809 24.6615562

C 7.6795987 10.6474578 19.8248979

C 10.8256844 12.6119545 23.7459508

H 10.7441840 12.1993424 24.7494711

C 6.2193500 15.8251876 21.3419705

C 9.9009433 12.2434244 22.7737563

H 9.0999050 11.5548667 23.0063951

C 5.1845652 10.2495682 20.0824779

C 3.9730160 10.5612563 14.9471538

H 3.7918926 10.0216673 14.0170578

C 6.4860480 9.8665342 19.6012454

C 10.7120665 10.2476463 20.3653555

H 11.0410320 9.3999246 19.7537958

H 10.1327090 9.8759877 21.2163527

H	11.5847170	10.7846004	20.7498027
C	9.1330292	12.2647913	20.3555643
H	8.9050490	13.1259138	19.7140444
C	5.9385487	13.0718399	18.9039063
C	8.3008928	14.5428882	24.8030326
H	8.3036335	15.0831501	23.8594068
C	3.6485013	14.9659852	24.3459663
H	3.5094092	14.4388293	23.3974604
H	3.1819592	15.9552763	24.2776057
H	3.1644861	14.3995626	25.1451933
C	5.9110932	13.8311554	24.3699514
H	5.3011167	12.9542172	24.5830750
C	7.2135843	13.0479409	26.3517121
H	6.3470114	12.4555053	26.6305014
C	10.6096183	11.7214665	18.3310650
H	11.4306843	12.3514442	18.6898548
H	9.9472745	12.3166315	17.6955155
H	11.0327240	10.9029147	17.7367034
C	5.3513766	15.7537297	26.0167162
H	6.4082815	15.9886301	26.1671641
H	5.0391306	15.0439325	26.7898817
H	4.7536288	16.6683788	26.1067102
H	3.3522093	7.8634507	18.4304641
H	4.2696603	10.5369488	23.5023292
C	4.8369546	10.1348773	24.3290253
H	7.0224429	11.6072627	24.0029122
C	5.4598911	8.7968855	24.1405457
H	5.0676822	8.3284906	23.2278529
H	5.2219853	8.1320694	24.9838233
C	6.9737025	8.9498276	23.9847197
H	7.4536381	7.9876935	23.7522680
H	7.4182280	9.3369411	24.9126175
C	7.1815373	9.9339024	22.8560685
H	6.6845646	9.5491516	21.9631418
H	8.2512678	9.9884219	22.6022127
N	6.6310778	11.2380793	23.1376636
C	4.6185046	10.6740715	25.6293858
C	4.1473714	11.7862423	28.2031905
C	5.2648620	10.1670546	26.7887449
C	3.7017881	11.7391807	25.8261501
C	3.4792638	12.2866863	27.0797914
C	5.0306594	10.7130867	28.0429722
H	5.9707715	9.3453981	26.6931506
H	3.1630048	12.1244382	24.9634331
H	2.7695523	13.1068507	27.1929959
H	5.5531829	10.3075250	28.9104501
H	3.9756426	12.2194388	29.1879546

TS3-R-triplet (E_{tot}=-4161.5536)

Fe	6.2532829	12.4625005	21.6486728
C	6.1673650	15.8536828	18.9243999
H	6.0431461	15.3368261	17.9779903
O	8.6511171	10.4536224	18.9085994
O	5.6243675	16.0009109	23.6313051
N	6.0936431	13.7300859	20.1132630
N	7.7935233	11.6309412	20.6095866
N	4.9013364	11.2996766	20.8449000
N	6.0412393	13.9266612	22.8928518
C	4.1703645	8.4157260	18.7050037
C	6.3444323	17.2445288	21.3171542
H	6.3723086	17.7733152	22.2674541
C	6.2989867	17.2252491	18.9291817
H	6.2962527	17.7567465	17.9758780
C	1.1420202	15.2190878	18.0237722
H	0.9949352	15.7857880	17.1037501
C	2.2270467	14.3796191	18.1444402
H	2.9381654	14.2720179	17.3284447
C	6.4180831	17.9446738	20.1318618
H	6.5272697	19.0278317	20.1300650
C	7.9006351	13.0725944	27.3472850
H	7.7778291	12.6582821	28.3484573
C	5.4483203	8.0181600	18.2747714
H	5.5748390	7.1523675	17.6268103
C	6.5410334	8.7628287	18.6689726
H	7.5343877	8.4919747	18.3183625
C	0.2167063	15.3548718	19.0837114
H	-0.6395266	16.0209558	18.9741675
C	4.0145365	9.5070570	19.5299348
H	3.0246002	9.8158900	19.8590033
C	5.3266095	11.1847713	15.1042583
H	6.1681702	11.0924668	14.4161434
C	4.4333039	11.9826296	17.2479681
C	5.5267749	11.8578343	16.3341491
C	6.1838635	15.0790619	20.1263928
C	3.1829509	11.4203755	16.8905618
H	2.3580975	11.5074655	17.5937944
C	10.8311478	13.6581289	21.4224863
H	10.8268110	14.2822231	20.5279969
C	11.7727591	13.0912933	23.5721672
H	12.5004416	13.2713912	24.3637104
C	3.5464862	12.7612828	19.4918156
C	0.4037980	14.6528723	20.2547197
H	-0.2977487	14.7606651	21.0835574
C	2.4382321	13.6306535	19.3309119
C	9.2747410	14.1096723	25.6606567
H	10.2341355	14.4999074	25.3304047
C	6.9328475	13.0513921	17.9005691
H	7.8746733	13.5267570	18.1512656
C	4.6253960	12.6416991	18.4933624
C	5.9917448	15.2098863	22.6062227
C	9.7737283	11.2240446	19.4091481
C	9.9098144	12.6129526	21.5340984
C	2.8518714	12.2787505	21.7709103
H	3.0545622	11.7766991	22.7073925
C	1.5121178	13.7862028	20.4153608
C	1.7572484	13.0971365	21.6273716
H	1.0708659	13.2313549	22.4626528
C	6.7725074	12.4234802	16.6898776
H	7.6062855	12.3531861	15.9905137
C	11.7605886	13.8970291	22.4340156
H	12.4787421	14.7110590	22.3304702
C	3.0168841	10.7752109	15.6861304
H	2.0478796	10.3459742	15.4292990
C	6.9521915	13.6326590	25.1827922
C	9.1355167	13.5827350	26.9429131
H	9.9847993	13.5684536	27.6264900
C	3.7419406	12.0761038	20.6925412
C	4.9898207	15.1222468	24.6055201
C	7.6021329	10.6864908	19.7256567
C	10.8244260	12.0778991	23.7095642
H	10.7982635	11.4755066	24.6169414
C	6.2241299	15.8369633	21.3447872
C	9.8955054	11.8470838	22.7002792
H	9.1604009	11.0579016	22.7980881
C	5.1201202	10.2729715	19.9926069
C	4.0971174	10.6536131	14.7832230
H	3.9544304	10.1371464	13.8335698
C	6.4190084	9.8934558	19.5083182
C	10.6745291	10.2490306	20.1464824
H	11.0030433	9.4714697	19.4474862
H	10.1264707	9.7804028	20.9697082
H	11.5475025	10.7643913	20.5585534
C	9.0723716	12.2612765	20.3406213

H 8.8844859 13.1894185 19.7836811

C 5.8795504 13.1384193 18.8406431

C 8.1949683 14.1200101 24.7794427

H 8.3164466 14.5059443 23.7704448

C 3.5229221 15.0040870 24.2279570

H 3.4196658 14.5652526 23.2317438

H 3.0558293 15.9952497 24.2375577

H 3.0079526 14.3533320 24.9412826

C 5.7283388 13.7911944 24.3195238

H 5.0602817 12.9382590 24.4448201

C 6.8172672 13.1108141 26.4747793

H 5.8428980 12.7609272 26.8121972

C 10.4846603 11.8737392 18.2427581

H 11.2841769 12.5164304 18.6290535

H 9.7880211 12.4780811 17.6560174

H 10.9313135 11.1127422 17.5919462

C 5.1845464 15.7398985 25.9692215

H 6.2463262 15.8844178 26.1855590

H 4.7574301 15.0865535 26.7366294

H 4.6679352 16.7058698 26.0043375

H 3.2887284 7.8583309 18.3848702

H 5.6946331 11.0929287 25.5201403

C 5.2499223 10.3347884 24.8836593

H 7.1521700 11.6266796 23.7751772

C 5.9731049 9.0347457 24.8474790

H 5.3424342 8.2502114 24.4138931

H 6.1971698 8.7189473 25.8811959

C 7.2611539 9.1360375 24.0371619

H 7.6711431 8.1399440 23.8194298

H 8.0272028 9.6945284 24.5976119

C 6.9258742 9.8838355 22.7663723

H 6.0958046 9.4016962 22.2386805

H 7.7771171 9.8514807 22.0722938

N 6.5369802 11.2417796 23.0581237

C 3.8453920 10.4326998 24.6545051

C 1.0424878 10.6557476 24.2675807

C 3.1393610 9.5303356 23.8183685

C 3.0858892 11.4532220 25.2811256

C 1.7159703 11.5624229 25.0907188

C 1.7695308 9.6426371 23.6342343

H 3.6857025 8.7671112 23.2694402

H 3.5937648 12.1497027 25.9469453

H 1.1633372 12.3595008 25.5895378

H 1.2608757 8.9480561 22.9650545

H -0.0310650 10.7472044 24.1072560

Product-S-triplet (E_{tot}=-4161.60396)

Fe 6.2366549 12.5475855 21.7122304

C 5.8525691 16.0054464 19.2216513

H 5.9199235 15.5211879 18.2526549

O 8.6282829 10.5390023 19.0268102

O 5.6450441 15.9067285 24.0017276

N 6.1825323 13.8637918 20.3142600

N 7.7703102 11.5948514 20.8223963

N 4.8366992 11.4598534 20.8516065

N 6.1155257 13.8948372 23.0916833

C 4.0719563 8.7519279 18.4914189

C 5.6021595 17.2813276 21.6732145

H 5.4927328 17.7628015 22.6418456

C 5.6361611 17.3632767 19.2822257

H 5.5472500 17.9255101 18.3511799

C 1.5353238 15.7645616 18.0267223

H 1.5122450 16.4140559 17.1510197

C 2.5534833 14.8500220 18.1730620

H 3.3365209 14.7662273 17.4235877

C 5.5125717 18.0257761 20.5188762

H 5.3357633 19.0993633 20.5634535

C 9.1669909 13.3528742 26.8846563

H 9.3861723 12.8597987 27.8327078

C 5.3488125 8.3168708 18.0955984

H 5.4648267 7.4886112 17.3982151

C 6.4561353 8.9739011 18.5898620

H 7.4511461 8.6723102 18.2706090

C 0.5214122 15.8756162 19.0055186

H -0.2792783 16.6043754 18.8769797

C 3.9340491 9.7981527 19.3767563

H 2.9442321 10.1420014 19.6704347

C 5.6226158 11.6594293 15.1242857

H 6.5005802 11.5671612 14.4832564

C 4.6350681 12.3603627 17.2604923

C 5.7783957 12.2375012 16.4082498

C 5.9768006 15.1994775 20.3949397

C 3.3829803 11.8940348 16.7892735

H 2.5201575 11.9802936 17.4454163

C 11.0121118 13.2292052 21.8367400

H 11.1586142 13.8685702 20.9659263

C 11.7333872 12.4500988 24.0055240

H 12.4342999 12.4903221 24.8382373

C 3.6506339 13.0555354 19.4929057

C 0.5516726 15.0686792 20.1217531

H -0.2213951 15.1522394 20.8872949

C 2.6080012 13.9977200 19.3056925

C 9.8288254 14.7976412 25.0709180

H 10.5776179 15.4233269 24.5884246

C 7.1441675 13.2514410 18.1310028

H 8.0890133 13.6619860 18.4697929

C 4.7837541 12.9268876 18.5554160

C 5.8757717 15.1829903 22.8803932

C 9.7727741 11.1632750 19.6522050

C 9.9206257 12.3577021 21.8736727

C 2.6853072 12.3973631 21.6208585

H 2.7059911 11.7507072 22.4923275

C 1.5871665 14.1201715 20.3049559

C 1.6535399 13.2925499 21.4509997

H 0.8660128 13.3714399 22.2022081

C 7.0265697 12.7112110 16.8727247

H 7.8955671 12.6448181 16.2174554

C 11.9220805 13.2662085 22.8908768

H 12.7756861 13.9433724 22.8476924

C 3.2622586 11.3404527 15.5350424

H 2.2912526 10.9836432 15.1900991

C 7.6342516 13.7893012 25.0596955

C 10.1108367 14.1918592 26.2956623

H 11.0757264 14.3546680 26.7757914

C 3.7109717 12.2773676 20.6520663

C 5.3874715 14.9470912 25.0502840

C 7.5631029 10.7532575 19.8346041

C 10.6169323 11.6188022 24.0709810

H 10.4403267 11.0138575 24.9591863

C 5.8336929 15.8828124 21.6469423

C 9.7212011 11.5747924 23.0082203

H 8.8672976 10.9095036 23.0165295

C 5.0497018 10.4728309 19.9427670

C 4.3910029 11.2196848 14.6931106

H 4.2835416 10.7764286 13.7026677

C 6.3521036 10.0534398 19.4993127

C 10.5475436 10.0606220 20.3554774

H 10.8490760 9.3106170 19.6155235

H 9.9147181 9.5851154 21.1115696

H 11.4350387 10.4659448 20.8509720

C 9.0980445 12.1668776 20.6358006

H 8.9821717 13.1448710 20.1489502

C 6.0389157 13.3273698 19.0048918

C 8.6027652 14.5883329 24.4486826

H 8.3826477 15.0564597 23.4921743

C 3.9049443 14.6010853 25.0216721

H 3.6416175 14.1474027 24.0600190

H 3.3111159 15.5117461 25.1585597

H 3.6770947 13.8979008 25.8287451

C 6.2210833 13.7470068 24.5504824

H 5.7368749 12.8378727 24.9016746

C 7.9320162 13.1567376 26.2692599

H 7.1765337 12.5338065 26.7430803

C 10.6047075 11.8385903 18.5847745

H 11.4506230 12.3483424 19.0588706

H 10.0059357 12.5676273 18.0323263

H 10.9969868 11.0947922 17.8810208

C 5.8057805 15.5565831 26.3664108

H 6.8504725 15.8766050 26.3278042

H 5.6974194 14.8147560 27.1647104

H 5.1656960 16.4177919 26.5915875

H 3.1799116 8.2641721 18.0959409

H 4.3490035 11.7992888 23.6758972

C 4.9204231 10.8987710 23.9182540

C 4.2946591 9.6513029 23.2963161

H 3.9507948 9.8991105 22.2900966

H 3.4639950 9.2713140 23.8996556

C 5.4826819 8.7067580 23.2222672

H 5.3434133 7.9137870 22.4796194

H 5.6610317 8.2435038 24.2002836

C 6.6294668 9.6356922 22.8383141

H 6.7876328 9.6307000 21.7641976

H 7.5686348 9.3445175 23.3231449

N 6.2332690 11.0190779 23.2405385

C 5.0059704 10.7501022 25.4191684

C 5.0504105 10.4758574 28.2180470

C 3.9072108 11.1368866 26.1956191

C 6.1203953 10.2108036 26.0658403

C 6.1434239 10.0725450 27.4539991

C 3.9268786 11.0074104 27.5810485

H 3.0307564 11.5529820 25.6995229

H 6.9905998 9.9052749 25.4893217

H 7.0268151 9.6570441 27.9394401

H 3.0651788 11.3264184 28.1681499

H 5.0718372 10.3776567 29.3032675

H 6.9125069 11.3860048 23.9021708

Product-R-triplet (E_{tot}=-4161.59607)

Fe 6.1325316 12.8443456 21.5097089

C 5.6436698 16.0180922 18.6107465

H 5.0393511 15.4812426 17.8853367

O 9.3264774 11.7343667 19.2718847

O 7.3076491 16.5800023 22.9893490

N 5.8520114 13.9786603 19.9106299

N 7.9801648 12.1677439 21.0269056

N 5.3331802 11.3588068 20.6160937

N 6.7583322 14.4129911 22.5876234

C 5.5450962 8.7397828 18.0475852

C 7.0945977 17.4445622 20.4819918

H 7.6374180 18.0034211 21.2400614

C 5.9748740 17.3343267 18.3726009

H 5.6228245 17.8135026 17.4576945

C 0.2253180 14.1266851 18.7447989

H -0.1563680 14.8672641 18.0411369

C 1.5481416 13.7486658 18.6930432

H 2.2244093 14.1710120 17.9518297

C 6.7478702 18.0583673 19.2979428

H 7.0332291 19.0915715 19.1054205

C 7.1639103 12.5931598 27.2108382

H 6.7805033 12.5700894 28.2312154

C 6.8836237 8.8461416 17.6267908

H 7.2666058 8.2156779 16.8257963

C 7.6999046 9.7753782 18.2365789

H 8.7368327 9.8810232 17.9251233

C -0.6461792 13.5564905 19.7019352

H -1.6927718 13.8614036 19.7301660

C 5.0536598 9.5474564 19.0485632

H 4.0044883 9.4935132 19.3279431

C 4.8628612 11.4352036 14.9464521

H 5.6296095 11.5011988 14.1729716

C 4.1026741 12.0271039 17.2096440

C 5.0882237 12.0984113 16.1752424

C 6.0811911 15.3110912 19.7730397

C 2.9374993 11.2579512 16.9624235

H 2.1906485 11.1671070 17.7458054

C 11.0875027 12.2681173 23.1022762

H 11.2783634 13.3412140 23.1044985

C 11.6103361 10.0502566 23.9004777

H 12.2111802 9.3859460 24.5218289

C 3.4262274 12.4000507 19.5802824

C -0.1725651 12.6244451 20.5977643

H -0.8335200 12.1904327 21.3487085

C 2.0661600 12.7985691 19.6086436

C 8.5957154 11.6847085 25.4905399

H 9.3306962 10.9640450 25.1403935

C 6.5007251 13.4027472 17.6411829

H 7.4194400 13.9452907 17.8277897

C 4.3412510 12.6783460 18.4621757

C 6.9663142 15.6208715 22.0896065

C 9.9850202 12.8088200 19.9983144

C 10.0617260 11.7586618 22.3005985

C 3.0417116 10.9595489 21.4864300

H 3.4253522 10.2176763 22.1748687

C 1.1859206 12.2283911 20.5827581

C 1.7025042 11.2697887 21.4826980

H 1.0269391 10.7961736 22.1944926

C 6.2882743 12.8033136 16.4293897

H 7.0543848 12.8422168 15.6540382

C 11.8587125 11.4239980 23.8964818

H 12.6512005 11.8385108 24.5202145

C 2.7445653 10.6205966 15.7558462

H 1.8385602 10.0349799 15.5949341

C 7.1631339 13.5789099 24.9987824

C 8.1240681 11.6633092 26.8029606

H 8.4905916 10.9147087 27.5055575

C 3.9353432 11.5714416 20.5817267

C 7.5015923 15.9350660 24.2556762

C 8.1378989 11.5286780 19.8838972

C 10.5768246 9.5371214 23.1164199

H 10.3668739 8.4671133 23.1222481

C 6.7482892 16.0990413 20.7649932

C 9.8042460 10.3871910 22.3247836

H 8.9922775 9.9888147 21.7201197

C 5.8639534 10.5200728 19.6965657

C 3.7112195 10.7097852 14.7320713

H 3.5498795 10.1992800 13.7822328

C 7.2316271 10.6062582 19.2790969

C 11.4718902 12.5468793 19.9373480

H 11.8123810 12.6267146 18.8981564

H 11.7064914 11.5501745 20.3213100

H 12.0058017 13.2889420 20.5401255

C 9.3014694 12.6862338 21.3798516

H 9.1965499 13.6764050 21.8343351

C 5.5384381 13.3460928 18.6848660

C 8.1199454 12.6453149 24.6062201

H 8.4250561 12.6468304 23.5704944

C 6.9495721 16.8327872 25.3415184

H 5.8887566 17.0412406 25.1634684

H 7.5009891 17.7801677 25.3592959

H 7.0666642 16.3449119 26.3147108

C 6.6830276 14.6412967 24.0578579

H 5.6269364 14.8573532 24.2864180

C 6.6806025 13.5428074 26.3136669

H 5.9192810 14.2564971 26.6271692

C 9.6054768 14.1415752 19.3768287

H 10.1822276 14.9435596 19.8525206

H 8.5433591 14.3381297 19.5426030

H 9.8230362 14.1370429 18.3032065

C 8.9913246 15.6834375 24.4302516

H 9.3488622 15.0031931 23.6525817

H 9.1982655 15.2326063 25.4054859

H 9.5231272 16.6378352 24.3478125

H 4.8742218 8.0322844 17.5591522

C 4.2651888 12.3320598 24.2167824

C 3.8448403 10.9846819 24.8156317

H 3.0539814 10.5325966 24.2092985

H 3.4580501 11.1069224 25.8331017

C 5.1269706 10.1611265 24.7708717

H 4.9470836 9.0810914 24.8280296

H 5.7900167 10.4533352 25.5947846

C 5.7431143 10.5891001 23.4500094

H 5.3273552 10.0694031 22.5867916

H 6.8308064 10.4717240 23.3990011

N 5.4416535 12.0315788 23.3379602

H 4.6798876 12.9164187 25.0532019

C 3.1680296 13.1929625 23.6467362

C 1.1189019 14.9053869 22.7561530

C 1.8879569 13.1340147 24.2105521

C 3.4054801 14.1381918 22.6495681

C 2.3902665 14.9748177 22.1934649

C 0.8740643 13.9829135 23.7718025

H 1.6720428 12.4078248 24.9925874

H 4.3901235 14.2220084 22.1961269

H 2.5980445 15.6699078 21.3811869

H -0.1193749 13.9092557 24.2155909

H 0.3182013 15.5444239 22.3866524

H 6.2079811 12.4426198 23.8735811

1,2-HAT

TS1,2-HAT-quintet (E_{tot}=-4161.50085)

Fe 6.8624971 13.0873623 21.8802878

C 6.0011241 16.4491098 19.0948458

H 6.0127413 15.8915347 18.1628222

O 7.9836162 9.4819764 20.0136104

O 6.4829127 16.7553801 23.8596797

N 6.7026946 14.4474562 20.2623235

N 7.7583425 11.5963011 20.7310324

N 5.1081477 12.3262421 21.3417480

N 7.1096636 14.7697227 23.0233445

C 3.1407860 9.2650933 20.8307699

C 5.9049016 17.8852973 21.4611611

H 5.8663034 18.4337502 22.3993959

C 5.6074125 17.7672475 19.0918700

H 5.3224776 18.2319987 18.1467168

C 2.0296314 16.2938392 17.8749212

H 1.9435740 16.6894956 16.8623384

C 2.9618185 15.3202993 18.1525944

H 3.6169627 14.9398977 17.3727696

C 5.5520475 18.5102553 20.2844514

H 5.2309593 19.5506169 20.2827530

C 10.7624359 14.4897446 26.2249289

H 11.1914632 13.9709190 27.0827318

C 4.1458924 8.2970053 20.6590775

H 3.8920334 7.2473728 20.5233302

C 5.4648453 8.7056029 20.6455676

H 6.2555266 7.9807939 20.4665005

C 1.1861839 16.7918881 18.8938829

H 0.4542456 17.5659830 18.6619894

C 3.4623077 10.5883173 21.0365080

H 2.6779970 11.3279614 21.1787391

C 5.5569652 11.3780194 15.6643635

H 6.3613921 11.1329237 14.9689474

C 4.8005507 12.5355262 17.6943429

C 5.8503873 12.2061041 16.7756948

C 6.3689468 15.7565724 20.2890326

C 3.5025674 12.0162688 17.4585293

H 2.7114779 12.2656365 18.1627047

C 11.2224299 12.8018291 20.0244735

H 11.2142031 12.6791987 18.9408178

C 12.2907752 13.6148885 22.0315948

H 13.1164703 14.1305081 22.5219724

C 4.0490334 13.7901847 19.7788900

C 1.2926509 16.3019214 20.1763748

H 0.6499022 16.6843043 20.9706723

C 3.0903202 14.7887474 19.4600933

C 10.9765366 16.0453993 24.3946093

H 11.5770521 16.7437307 23.8117516

C 7.4070759 13.4918022 18.1202856

H 8.3956290 13.9104654 18.3027186

C 5.0861838 13.3518215 18.8190491

C 6.6510605 15.9736404 22.7699336

C 9.3408031 10.0095757 20.0160074

C 10.1542203 12.3102563 20.7797450

C 3.2987628 13.8127910 22.0967745

H 3.4021870 13.4079337 23.1011705

C 2.2379945 15.2957799 20.4950961

C 2.3747611 14.7915658 21.8112216

H 1.7320350 15.1905794 22.5971466

C 7.1455974 12.7290782 17.0051218

H 7.9349136 12.5187471 16.2814823

C 12.2896161 13.4484916 20.6462955

H 13.1157911 13.8321460 20.0466146

C 3.2510689 11.2104584 16.3708567

H 2.2481435 10.8141081 16.2091638

C 8.8816201 14.8832046 24.7432291

C 11.5283687 15.4087721 25.5067662

H 12.5576685 15.6128660 25.8020741

C 4.1305466 13.2980994 21.0771488

C 6.5533001 15.8705323 25.0118250

C 7.1959754 10.4317546 20.5649249

C 11.2186001 13.1418826 22.7869340

H 11.1968856 13.2983893 23.8617683

C 6.3225076 16.5344233 21.4976525

C 10.1564643 12.4957257 22.1630910

H 9.3068932 12.1395546 22.7428614

C 4.8136082 11.0374206 21.0867793

C 4.2853555 10.8875377 15.4630165

H 4.0739489 10.2486925 14.6051491

C 5.8289654 10.0574923 20.8264757

C 10.0386986 9.4451384 21.2392656

H 10.0404976 8.3506468 21.1833301

H 9.5135713 9.7607367 22.1464821

H 11.0684359 9.8121595 21.2915143

C 9.0725855 11.5333741 20.1002038

H 8.9586612 11.9088992 19.0745232

C 6.3963314 13.7683847 19.0688583

C 9.6648860 15.7785373 24.0113896

H 9.2405813 16.2641302 23.1352206

C 5.1432268 15.3760297 25.2974990

H 4.7642194 14.8242641 24.4323391
H 4.4811788 16.2254586 25.5002469
H 5.1522631 14.7123280 26.1705954
C 7.4142885 14.7136901 24.4498165
H 7.0625785 13.7555004 24.8427872
C 9.4460319 14.2324645 25.8425923
H 8.8417299 13.5258839 26.4140407
C 10.0177741 9.6085254 18.7254349
H 11.0088844 10.0749159 18.6856587
H 9.4266924 9.9465205 17.8669245
H 10.1395867 8.5201805 18.6722266
C 7.1358914 16.6487881 26.1673893
H 8.1103401 17.0664463 25.9001258
H 7.2683382 15.9831112 27.0271999
H 6.4542294 17.4599319 26.4486370
H 2.0908122 8.9688441 20.8141968
H 2.4346586 9.6592796 24.1623661
C 3.0730331 9.0954263 24.8580135
H 2.9777545 9.5725261 25.8439604
C 4.5202635 9.2031576 24.3760847
H 4.5896257 8.7927732 23.3617618
H 5.1651066 8.5872112 25.0198734
C 5.0149373 10.6412831 24.3644640
H 4.9788017 11.0709428 25.3783372
H 4.3640548 11.2661936 23.7390299
C 6.4024682 10.7764440 23.7956219
H 6.9011412 9.8364837 23.4913170
H 7.4057846 11.4604415 24.2654259
N 6.8635441 11.9547897 23.2890075
C 2.6096211 7.6676371 24.9350644
C 1.8207661 4.9689231 25.0496687
C 2.3502285 6.9463874 23.7634409
C 2.4711853 7.0134317 26.1629675
C 2.0787887 5.6757178 26.2235344
C 1.9586312 5.6106555 23.8175419
H 2.4677406 7.4433500 22.8025386
H 2.6711657 7.5617669 27.0852303
H 1.9726496 5.1847711 27.1917302
H 1.7579289 5.0664632 22.8936295
H 1.5120468 3.9242366 25.0939564

TS1,2-HAT-triplet (E_{tot}=-4161.50499)

Fe 6.7780229 13.0883363 21.6793564
C 7.4922230 15.9947969 18.5688011
H 7.1990302 15.4384823 17.6840830
O 7.9835824 9.3734723 20.3912634
O 6.9317030 16.9083103 23.1803786
N 6.8007265 14.1515219 20.0157502
N 7.6157815 11.5258153 20.8992977
N 4.9897194 12.1735678 21.2142652
N 6.5940303 14.7511513 22.6537463
C 3.1770054 8.9944778 21.5278234
C 8.1268440 17.5383016 20.7887610
H 8.3169027 18.1468250 21.6706534
C 8.0648620 17.2392554 18.4181569
H 8.2276681 17.6253858 17.4102606
C 2.3621064 16.9338861 18.5114219
H 2.3704725 17.5357204 17.6020497
C 3.1600539 15.8145186 18.5991236
H 3.8006777 15.5205136 17.7699379
C 8.4287023 18.0183656 19.5327972
H 8.8921158 18.9955420 19.4058191
C 7.6666439 13.2759645 27.1488332
H 7.3216369 12.8657869 28.0984817
C 4.2334204 8.0601456 21.5084613
H 4.0343709 6.9998916 21.6430429
C 5.5176234 8.5211745 21.3249396
H 6.3487758 7.8206430 21.2717603
C 1.5351778 17.3130076 19.5925730
H 0.9101744 18.2027882 19.5118943
C 3.4107283 10.3450249 21.4111666
H 2.5828973 11.0507117 21.4159409
C 4.5379563 11.5358045 15.5394288
H 5.1906430 11.1648354 14.7476081
C 4.2798118 12.6998748 17.6886546
C 5.1231371 12.2060441 16.6413818
C 7.2825774 15.4046418 19.8521990
C 2.8813356 12.4913829 17.5846389
H 2.2409693 12.8628130 18.3816821
C 10.6569497 13.0908524 19.4777153
H 10.5353023 12.7527507 18.4483401
C 11.7296704 14.5250083 21.0930210
H 12.4516151 15.3052259 21.3339716
C 3.9956419 13.8741107 19.9001269
C 1.5232395 16.5596389 20.7471818
H 0.8895924 16.8463903 21.5880914
C 3.1727713 15.0216636 19.7746218
C 9.4526457 13.7634606 25.6031891
H 10.5109232 13.7367268 25.3445285
C 7.0560081 13.0607468 17.8238803
H 8.1215870 13.2694845 17.8816002
C 4.8565613 13.3781644 18.8007945
C 7.0380532 15.9253350 22.2609393
C 9.2772793 10.0296563 20.2157595
C 9.8470164 12.5445718 20.4758646
C 3.1818666 13.5389473 22.1734597
H 3.2333443 12.9553178 23.0909815
C 2.3321387 15.4049424 20.8717994
C 2.3489815 14.6249653 22.0558817
H 1.7078496 14.9221323 22.8871358
C 6.5196727 12.4157133 16.7357773
H 7.1631839 12.0672320 15.9263652
C 11.5958889 14.0745314 19.7802915
H 12.2093705 14.5034432 18.9879888
C 2.3417941 11.8364191 16.4993411
H 1.2633023 11.6870229 16.4380026
C 7.1672744 14.3156650 25.0158736
C 9.0249857 13.2462050 26.8266149
H 9.7447499 12.8156734 27.5228131
C 4.0227433 13.1591647 21.0959082
C 5.9951202 16.4197034 24.1804249
C 7.1280043 10.3061465 20.8712396
C 10.9226059 13.9848240 22.0938151
H 11.0235038 14.3433030 23.1161783
C 7.5436001 16.2648226 20.9732634
C 9.9838838 13.0013219 21.7888035
H 9.3341589 12.5953004 22.5625735
C 4.7351519 10.8712569 21.2992964
C 3.1743941 11.3539664 15.4651998
H 2.7356858 10.8368815 14.6113885
C 5.8045725 9.9004098 21.2148110
C 10.1346839 9.7362300 21.4313594
H 10.2362998 8.6524862 21.5574689
H 9.6830554 10.1640994 22.3302104
H 11.1259900 10.1837198 21.3033943
C 8.8405884 11.5051425 20.1089378
H 8.5453744 11.6705123 19.0650684
C 6.2370961 13.5270397 18.8826035
C 8.5314685 14.3066915 24.7093342
H 8.8449486 14.6858801 23.7418254
C 4.5996511 16.8322222 23.7406962
H 4.3557809 16.3806898 22.7749394
H 4.5428282 17.9235441 23.6563806

H 3.8641870 16.4899818 24.4781061
C 6.1676483 14.8864530 24.0476889
H 5.2118201 14.3716655 24.1755535
C 6.7469920 13.8057414 26.2487886
H 5.6829860 13.8001612 26.4868071
C 9.8996385 9.5102636 18.9394480
H 10.8220606 10.0679484 18.7394252
H 9.2092271 9.6445026 18.0993969
H 10.1453781 8.4460008 19.0338133
C 6.3816433 17.0200244 25.5121041
H 7.4210694 16.7821983 25.7573283
H 5.7378487 16.6148068 26.3003768
H 6.2535908 18.1080296 25.4759221
H 2.1527374 8.6403165 21.6483294
H 4.1710225 9.4506659 26.2211971
C 4.8318672 8.6988392 25.7636727
H 5.5589952 8.3991326 26.5311794
C 5.5660962 9.3377665 24.5832968
H 4.8403305 9.6783431 23.8372565
H 6.1770439 8.5783056 24.0806605
C 6.4466163 10.5077852 25.0238372
H 7.2157841 10.1468048 25.7227877
H 5.8418313 11.2623030 25.5402746
C 7.0985647 11.1454656 23.8294682
H 7.8368516 10.5314186 23.2904194
H 7.4210604 12.3411216 24.1750482
N 6.5604294 12.2600387 23.2717039
C 4.0294008 7.5079450 25.3228221
C 2.5788807 5.2930319 24.3743943
C 2.8084343 7.6811379 24.6624560
C 4.5118394 6.2076766 25.4999196
C 3.7947808 5.1066093 25.0323446
C 2.0883008 6.5864201 24.1913059
H 2.4341472 8.6907206 24.4948900
H 5.4642067 6.0575450 26.0121696
H 4.1857868 4.0997354 25.1845615
H 1.1420609 6.7424395 23.6718774
H 2.0162815 4.4345168 24.0068621

After1,2-HAT-quintet (E_{tot}=-4161.59818)

Fe 6.6277118 13.1586962 21.9649312
C 6.2291183 15.9874894 18.5115009
H 6.0288877 15.2498590 17.7393293
O 7.8291551 9.8321353 19.7250881
O 6.7754709 17.2682466 23.0876516
N 6.5050194 14.1972948 20.1217554
N 7.6103126 11.5628666 21.1461760
N 4.8831438 12.2707100 21.6770851
N 6.8299882 15.0391257 22.7988783
C 2.9390962 9.4656300 20.3089008
C 6.6834547 17.9005074 20.4650860
H 6.8390934 18.6420476 21.2450204
C 6.2394716 17.3233855 18.1863338
H 6.0502895 17.6192814 17.1527218
C 1.8597791 16.4949481 18.4476925
H 1.7206873 16.8985880 17.4442755
C 2.6955165 15.4191884 18.6429700
H 3.2211013 14.9626160 17.8077265
C 6.4831316 18.3069397 19.1648168
H 6.4926437 19.3646688 18.9062263
C 9.8506386 14.4454943 26.5625714
H 9.9953812 14.1197652 27.5933096
C 3.9368020 8.6238466 19.7957279
H 3.6771277 7.6891986 19.3016936
C 5.2588195 8.9967629 19.9338711
H 6.0443910 8.3602971 19.5339705
C 1.1851518 17.0882640 19.5391829
H 0.5264864 17.9407003 19.3710386
C 3.2769585 10.6498081 20.9288098
H 2.5003460 11.3132680 21.3030403
C 4.2810627 10.7945874 16.1936378
H 4.9416081 10.2943088 15.4837362
C 4.0018134 12.3087625 18.1078651
C 4.8555558 11.6501256 17.1653847
C 6.4809242 15.5142776 19.8392494
C 2.6056867 12.0773042 18.0324440
H 1.9615278 12.5706775 18.7565865
C 11.1783120 12.5716675 21.4502213
H 11.2861095 13.0357089 20.4689228
C 12.0591818 12.0970728 23.6479775
H 12.8500870 12.1965639 24.3906435
C 3.7566366 13.7766908 20.1669210
C 1.3672846 16.5944700 20.8122383
H 0.8582028 17.0519418 21.6620717
C 2.8943888 14.8787043 19.9388374
C 10.7563574 15.1030761 24.4266256
H 11.6125149 15.2792648 23.7781797
C 6.7754024 12.7234913 18.1800593
H 7.8385841 12.9454506 18.2077637
C 4.5701117 13.1569621 19.0961605
C 6.7714028 16.2224928 22.2264308
C 9.1890309 10.2922522 19.9069847
C 10.0118141 11.8642215 21.7489206
C 3.3043722 13.9272397 22.5591368
H 3.5000414 13.5498800 23.5610827
C 2.2221414 15.4900832 21.0485875
C 2.4544290 14.9893716 22.3522559
H 1.9521709 15.4656846 23.1953128
C 6.2498607 11.8791077 17.2316323
H 6.9006604 11.3829436 16.5105495
C 12.2026498 12.6787792 22.3895722
H 13.1095284 13.2316430 22.1429787
C 2.0763509 11.2476657 17.0704728
H 0.9990582 11.0830853 17.0291956
C 8.3593201 15.0132330 24.7360402
C 10.9527653 14.7087638 25.7491816
H 11.9629398 14.5855190 26.1393567
C 3.9527108 13.3010586 21.4688960
C 6.4959811 16.7287187 24.4008996
C 7.0282694 10.5677711 20.5288483
C 10.8864055 11.4105685 23.9615818
H 10.7625034 10.9664905 24.9495302
C 6.6801663 16.5329304 20.8366931
C 9.8755408 11.2870331 23.0131458
H 8.9655238 10.7375530 23.2362745
C 4.6227834 11.0739528 21.0786720
C 2.9192987 10.5960770 16.1421592
H 2.4891282 9.9370094 15.3875166
C 5.6374536 10.2022662 20.5657090
C 9.9186889 9.2162368 20.6918520
H 9.9141402 8.2877467 20.1094411
H 9.4201055 9.0351445 21.6482570
H 10.9525275 9.5168748 20.8891918
C 8.9858918 11.6374201 20.6768043
H 9.0451898 12.4741147 19.9658954
C 5.9514413 13.3631631 19.1334336
C 9.4672139 15.2468974 23.9199624
H 9.3090613 15.5382266 22.8836242
C 4.9896838 16.7787140 24.6098803
H 4.4877086 16.1728533 23.8490336
H 4.6358544 17.8133473 24.5349702
H 4.7393080 16.3871305 25.6037795
C 6.9590185 15.2588897 24.2366252

H 6.2702694 14.5952131 24.7690316
C 8.5620216 14.5976345 26.0550341
H 7.6968318 14.3913019 26.6866069
C 9.8201481 10.5061475 18.5464612
H 10.8216991 10.9322573 18.6800021
H 9.2138319 11.1909849 17.9485574
H 9.9140099 9.5539363 18.0112231
C 7.2463574 17.5575233 25.4177457
H 8.3171852 17.5650190 25.1952188
H 7.1034424 17.1342402 26.4177642
H 6.8635845 18.5847540 25.4098315
H 1.8883711 9.1909691 20.2131147
H 3.3111231 9.4618774 23.4845128
C 3.8118023 8.9903671 24.3405483
H 3.2642526 9.2817581 25.2484693
C 5.2403494 9.5336316 24.4185832
H 5.7619545 9.3040734 23.4813118
H 5.7818007 9.0231312 25.2287182
C 5.2508436 11.0473302 24.6430409
H 4.7069296 11.2669181 25.5775292
H 4.7547989 11.5540515 23.8091351
C 6.6299729 11.5806386 24.7617184
H 7.2364501 11.2104569 25.6046112
H 8.0767460 12.6790608 24.1762940
N 7.1254384 12.4137732 23.9221022
C 3.7942337 7.4965663 24.1870381
C 3.8456294 4.7017172 23.8869226
C 4.0862968 6.9175422 22.9468684
C 3.5324642 6.6562781 25.2737667
C 3.5551819 5.2688184 25.1276304
C 4.1109784 5.5325891 22.7968830
H 4.2840060 7.5631609 22.0938155
H 3.3014413 7.0955565 26.2459868
H 3.3414847 4.6288712 25.9847345
H 4.3337846 5.0992340 21.8208441
H 3.8596219 3.6177244 23.7690678

After1,2-HAT-triplet (E_{tot}=-4161.59348)

Fe 6.6674266 13.2462398 21.9083529
C 6.4219511 15.8503108 18.4936672
H 6.1664646 15.1121185 17.7395090
O 7.8875893 9.9397743 19.6941289
O 6.8983253 17.1870905 23.0636464
N 6.4624910 14.1093853 20.1851421
N 7.6533093 11.6932019 21.0738378
N 4.8965507 12.2919634 21.7073082
N 6.7672790 14.9628300 22.7606630
C 3.0267504 9.4442998 20.3390507
C 7.0002726 17.7917049 20.3955743
H 7.1957432 18.5404923 21.1599656
C 6.5794769 17.1670405 18.1244384
H 6.4531339 17.4388034 17.0751114
C 1.7558178 16.4619720 18.5213433
H 1.5940503 16.8598985 17.5189513
C 2.6015516 15.3909717 18.7037384
H 3.1096221 14.9340225 17.8576769
C 6.8881481 18.1611347 19.0742458
H 7.0106280 19.2010129 18.7754954
C 9.5490196 14.1848566 26.6739215
H 9.6224743 13.8341817 27.7040679
C 4.0465963 8.6196290 19.8326390
H 3.8108331 7.6707391 19.3543234
C 5.3558321 9.0335915 19.9592452
H 6.1566869 8.4142670 19.5624565
C 1.0982665 17.0553080 19.6228538
H 0.4298003 17.9018890 19.4643325
C 3.3319894 10.6393367 20.9482811
H 2.5380249 11.2829060 21.3211668
C 4.1526525 10.8130038 16.2107017
H 4.7997193 10.3131995 15.4883469
C 3.9097323 12.3022177 18.1480717
C 4.7475252 11.6443345 17.1913513
C 6.5876888 15.4125001 19.8442555
C 2.5104513 12.0903552 18.0835663
H 1.8804485 12.5806242 18.8218691
C 11.2155586 12.6849760 21.4350562
H 11.3282211 13.1871381 20.4731003
C 12.0987474 12.0994762 23.6052658
H 12.8943783 12.1534373 24.3477821
C 3.7030596 13.7628135 20.2204757
C 1.3108828 16.5681076 20.8943791
H 0.8165657 17.0260448 21.7527367
C 2.8312906 14.8575043 19.9980720
C 10.6000366 14.8516533 24.6077278
H 11.4971799 15.0082243 24.0130266
C 6.6888677 12.6583535 18.2196860
H 7.7541725 12.8593420 18.2554332
C 4.4959283 13.1279829 19.1462874
C 6.8452292 16.1535889 22.1950306
C 9.2377929 10.4374257 19.8317868
C 10.0433528 11.9753353 21.7037224
C 3.3259099 13.9426399 22.6189652
H 3.5525484 13.5902744 23.6228114
C 2.1795607 15.4727597 21.1181290
C 2.4515349 14.9858897 22.4199118
H 1.9642784 15.4645744 23.2704789
C 6.1459064 11.8468886 17.2528799
H 6.7856339 11.3524865 16.5212228
C 12.2440393 12.7379445 22.3747249
H 13.1572658 13.2895025 22.1493409
C 1.9613917 11.2814745 17.1148857
H 0.8820286 11.1295815 17.0821973
C 8.1879834 14.8411130 24.7785800
C 10.7049964 14.4255389 25.9308999
H 11.6861649 14.2598271 26.3759029
C 3.9515355 13.3050467 21.5204546
C 6.4708491 16.6671785 24.3486940
C 7.0800970 10.6691568 20.4974043
C 10.9174247 11.4165737 23.8930987
H 10.7895704 10.9324893 24.8612972
C 6.8489086 16.4451962 20.8070239
C 9.9025868 11.3479200 22.9440176
H 8.9907591 10.7909535 23.1434654
C 4.6695583 11.1059522 21.0965951
C 2.7879135 10.6344273 16.1689356
H 2.3423004 9.9925307 15.4084083
C 5.7054863 10.2585941 20.5741939
C 10.0222466 9.3737032 20.5813788
H 10.0319897 8.4558113 19.9823823
H 9.5544199 9.1585034 21.5456605
H 11.0509392 9.7032306 20.7582081
C 9.0267154 11.7789421 20.6138431
H 9.1044269 12.6275795 19.9180500
C 5.8792250 13.2890556 19.1866231
C 9.3483638 15.0485081 24.0304030
H 9.2607895 15.3618509 22.9922470
C 4.9625955 16.8369071 24.4386026
H 4.4737577 16.2763455 23.6366246
H 4.7008534 17.8974001 24.3490124
H 4.6039122 16.4617455 25.4051975
C 6.8328841 15.1674133 24.2085244
H 6.0725118 14.5511874 24.6972681
C 8.2978346 14.3928017 26.0970641

H 7.3906570 14.2075644 26.6738028

C 9.8252696 10.6676392 18.4535253

H 10.8243286 11.1070993 18.5607241

H 9.1967106 11.3447471 17.8708950

H 9.9177805 9.7183441 17.9128957

C 7.2005117 17.4329494 25.4277074

H 8.2829363 17.3638439 25.2882184

H 6.9497308 17.0165272 26.4092743

H 6.8938688 18.4850233 25.4006768

H 1.9833227 9.1399315 20.2501526

H 3.2232413 9.4982455 23.5464005

C 3.7874189 9.0660483 24.3837708

H 3.2781038 9.3538094 25.3146888

C 5.1951217 9.6649071 24.3690467

H 5.6702368 9.4358640 23.4076917

H 5.8013605 9.1930146 25.1564519

C 5.1662643 11.1854164 24.5579135

H 4.6886822 11.4138903 25.5249221

H 4.5986890 11.6415245 23.7439179

C 6.5400352 11.7349421 24.5481497

H 7.1844229 11.4825863 25.4012147

H 8.0189879 12.6799861 23.8289889

N 7.0514716 12.4437379 23.6004919

C 3.8294012 7.5699968 24.2590394

C 4.0059573 4.7752535 24.0095699

C 4.1146774 6.9807645 23.0217656

C 3.6379046 6.7401901 25.3681782

C 3.7230125 5.3526792 25.2472364

C 4.2010452 5.5959761 22.8970907

H 4.2629953 7.6175924 22.1518765

H 3.4147012 7.1873627 26.3386722

H 3.5651308 4.7206149 26.1222766

H 4.4188581 5.1547336 21.9234127

H 4.0698791 3.6910842 23.9118858

N2 (E_{tot}=-109.51915)

N 0.0000000 0.0000000 0.9861683

N 0.0000000 0.0000000 -0.1161683

References

(S1) Ito, Y.; Kusakabe, T.; Dhage, Y. D.; Takahashi, K.; Sakata, K.; Sasai, H.; Kato, K. Total Synthesis of (-)-Graminin A Based on Asymmetric Cyclization Carbonylation of Propargyl Acetate. *J. Org. Chem.* **2019**, *84*, 16268-16277. DOI: 10.1021/acs.joc.9b02886.

(S2) Doherty, S.; Knight, J. G.; Smyth, C. H.; Sore, N. T.; Rath, R. K.; McFarlane, W.; Harrington, R. W.; Clegg, W. Modular Synthesis of a New Class of Bis(amino-oxazoline) Using Palladium-Catalyzed Buchwald−Hartwig Amination Methodology. *Organometallics* **2006**, *25*, 4341-4350. DOI: 10.1021/om0602714.

(S3) a) Evans, D. F. The determination of the paramagnetic susceptibility of substances in solution by nuclear magnetic resonance. *Journal of the Chemical Society (Resumed)* **1959**. DOI: 10.1039/jr9590002003. b) Sur, S. K. Measurement of magnetic susceptibility and magnetic moment of paramagnetic molecules in solution by high-field fourier transform NMR spectroscopy. *J. Magn. Reson.* **1989**, *82*, 169-173. DOI: 10.1016/0022-2364(89)90178-9.

(S4) Kuijpers, P. F.; Tiekink, M. J.; Breukelaar, W. B.; Broere, D. L. J.; van Leest, N. P.; van der Vlugt, J. I.; Reek, J: N. H.; de Bruin, B. *Chem. Eur. J.* **2017**, *23*, 7945-7952. DOI: 10.1002/chem.201700358

(S5) Qin, J.; Zhou, Z.; Cui, T.; Hemming, M.; Meggers, E. Enantioselective intramolecular C-H amination of aliphatic azides by dual ruthenium and phosphine catalysis. *Chem. Sci.* **2019**, *10*, 3202-3207. DOI: 10.1039/c9sc00054b.

(S6) You, T.; Zeng, S. H.; Fan, J.; Wu, L.; Kang, F.; Liu, Y.; Che, C. M. A soluble iron(II)-phthalocyanine-catalyzed intramolecular C(sp(3))-H amination with alkyl azides. *Chem. Commun,* **2021**, *57*, 10711-10714. DOI: 10.1039/d1cc04573c.

(S7) Shing, K. P.; Liu, Y.; Cao, B.; Chang, X. Y.; You, T.; Che, C. M. N-Heterocyclic Carbene Iron(III) Porphyrin-Catalyzed Intramolecular C(sp(3))-H Amination of Alkyl Azides. *Angew. Chem. Int. Ed.* **2018**, *57*, 11947-11951. DOI: 10.1002/anie.201806059 .

(S8) Bruker, SAINT V8.40B, Bruker AXS Inc., Madison, Wisconsin, USA, 2001

(S9) Krause, L.; Herbst-Irmer, R.; Sheldrick, G. M.; Stalke, D. Comparison of silver and molybdenum microfocus X-ray sources for single-crystal structure determination. *J. Appl. Cryst.* **2015**, *48*, 3-10. DOI: 10.1107/S1600576714022985.

(S10) Sheldrick, G. M. SHELXT - integrated space-group and crystal-structure determination. *Act. Cryst. A* **2015**, *71*, 3-8. DOI: 10.1107/S2053273314026370.

(S11) Spek, A. L. PLATON SQUEEZE: a tool for the calculation of the disordered solvent contribution to the calculated structure factors. *Act Cryst. C* **2015**, *71*, 9-18. DOI: 10.1107/S2053229614024929.

(S12) Spek, A. L. (2001). PLATON. Utrecht University, The Netherlands.

(S13) Sheldrick, G. M. Crystal structure refinement with SHELXL. *Act Cryst. C* **2015**, *71*, 3-8. DOI: 10.1107/S2053229614024218.

(S14) TURBOMOLE, Version 7.8.1 (TURBOMOLE Gmbh, Karlsruhe, Germany).

(S15) PQS version 2.4, 2001, Parallel Quantum Solutions, Fayetteville, Arkansas, USA (the Baker optimizer is available separately from PQS upon request

(S16) Baker, J. An algorithm for the location of transition states. *J. Comp. Chem.* **1986**, *7*, 385-395. DOI: 10.1002/jcc.540070402.

(S17) Budzelaar, P. H. Geometry optimization using generalized, chemically meaningful constraints. *J. Comp. Chem.* **2007**, *28*, 2226-2236. DOI: 10.1002/jcc.20740.

(S18) Swart, M.; Ehlers, A. W.; Lammertsma, K. Performance of the OPBE exchange-correlation functional. *Mol. Phys.* **2004**, *102*, 2467-2474. DOI: 10.1080/0026897042000275017.
(S19) a) Weigend, F.; Ahlrichs, R. Balanced basis sets of split valence, triple zeta valence and quadruple zeta valence quality for H to Rn: Design and assessment of accuracy. *Phys. Chem. Chem. Phys.* **2005**, *7,* 3297-3305. DOI: 10.1039/b508541a. b) Weigend, F.; Häser, M.; Patzelt, H.; Ahlrichs, R. RI-MP2: optimized auxiliary basis sets and demonstration of efficiency. *Chem. Phys. Lett.* **1998**, *294*, 143-152. DOI: 10.1016/s0009-2614(98)00862-8.
(S20) Grimme, S.; Antony, J.; Ehrlich, S.; Krieg, H. A consistent and accurate ab initio parametrization of density functional dispersion correction (DFT-D) for the 94 elements H-Pu. *J. Chem. Phys.* **2010**, *132*, 154104. DOI: 10.1063/1.3382344.
